# Dipyrromethane–diphosphines: nickel and palladium complexes as catalysts for the selective hydroboration of CO_2_ to methoxyborane under mild conditions with mechanistic study

**DOI:** 10.1039/d6ra04348h

**Published:** 2026-08-05

**Authors:** Rohit Gupta, Ashok Kumar, Ganesan Mani

**Affiliations:** a Department of Chemistry, Indian Institute of Technology Kharagpur Kharagpur 721 302 India gmani@chem.iitkgp.ac.in +91 3222 282252 +91 3222 282320

## Abstract

The dipyrromethane–diphosphine ligands [R_2_C(C_4_H_3_N)_2_-1,9-(CH_2_PPh_2_)_2_] represented as L1H2, L2H2 and L3H2 containing phenyl, ethyl and cyclohexyl groups, react with [Pd(COD)Cl_2_] to give complexes of the type [PdCl_2_(L1H2-κ^2^-*P*,*P*)] 3, [Pd(L1-κ^4^-*P*,*N*,*N*,*P*)] 4a, [Pd(L2-κ^4^-*P*,*N*,*N*,*P*)] 4b and [Pd(L3-κ^4^-*P*,*N*,*N*,*P*)] 4c which were characterized by both spectroscopic and single crystal X-ray diffraction methods. The coordination mode of ligand κ^2^ or κ^4^ can readily be identified by UV-vis spectrum. The catalytic applications of these palladium complexes and the reported analogous nickel complexes were investigated in the hydroboration of CO_2_ using 9-BBN. Methoxyborane was selectively obtained in a quantitative yield with a TON of 200 from the reduction of CO_2_ (1 atm) in the presence of 0.5 mol% of palladium complex 4a at 25 °C. Mechanistic studies established the formation of Ni and Pd hydrides by the cleavage of the B–H bond across the palladium atom and ligand framework, and CO_2_ insertion followed by further reductions to give methoxyborane *via* its acetal derivative.

## Abstract 2

The selective hydroboration of CO_2_ (1 atm) to methoxyborane catalyzed by Pd and Ni complexes of dipyrromethane-based diphosphines at 25 °C operates *via* the *in situ* generated metal hydrides, and their catalytic activity depends on the *meso* groups.

## Graphical abstract

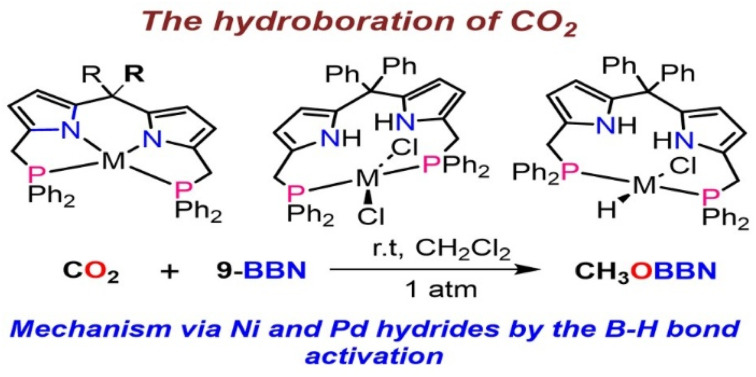

## Introduction

The chemical transformation of carbon dioxide into useful products such as formic acid and methanol is a promising way to mitigate the effect of its rising concentration in the atmosphere.^1^ However, developing an efficient, economically viable, and sustainable catalyst remains a challenge owing to its inherent thermodynamic and kinetic stability, and hence, it continues to be an active area of research. The direct hydrogenation of CO_2_ with H_2_ is the most atom-efficient method. However, H_2_ is a flammable gas and requires harsh reaction conditions.^2^ Alternatively, hydrosilanes^3^ and hydroboranes^4^ are safer to handle and can be tuned to selectively give a particular reduced product of CO_2_ in high yield under mild conditions.^5–8^ Of particular interest is the functionalization of organic molecules using formoxyborane, bis(boryl)acetal, and methoxyborane that are formed when CO_2_ is reduced with hydroboranes.^9,10^ Methoxyboranes are easily hydrolyzable to give methanol. In addition, the controlled hydroboration of CO_2_ paves the way to understanding the mechanism of transformation of CO_2_ that will be useful to optimize industrial-scale reactions for producing methanol.^11^

In homogeneous catalysis, since the pioneering work of Guan's group on the hydroboration of CO_2_ catalyzed by pincer nickel complex,^12^ a plethora of organometallic catalysts based on noble metals (Ru,^13^ Pd^14^) as well as earth-abundant metals (Zn,^15^ Ni,^16^ Fe,^17^ Mn^18^) have been described. In particular, group 10 metal catalysts have been extensively studied for this transformation of CO_2_.^19,20^ Very recently, we reported the superior catalytic activity of NHC pincer palladium thiolate complex over its hydride and nickel counterparts in the selective hydroboration of CO_2_ to methoxyborane under mild conditions with TON >25 000.^21^ However, the number of group 10 metal catalysts for the selective reduction of CO_2_ to methoxyborane still remains very limited, especially with palladium.^5,6,14^ In addition, to the best of our knowledge all the nickel and palladium complexes reported so far contain pincer backbones. Non-pincer metal complexes catalyzed selective hydroboration of CO_2_ to methoxyborane^18*d*^ and formate^22,23^ have also been described.

**Chart 1:**
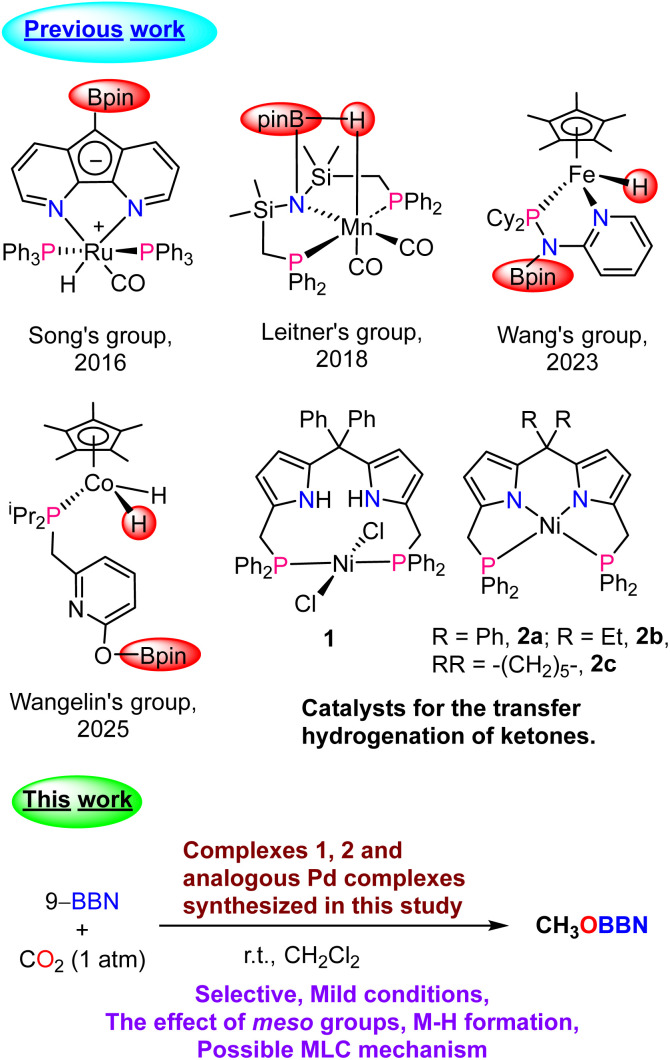
The hydroboration of CO_2_ catalyzed by metal complexes *via* MLC mechanisms involving the B–H activation of boranes and the present work.

In homogeneous catalysis, a variety of metal complexes that work *via* the mechanism of metal–ligand cooperation (MLC), an alternative to traditional metal atom-centered reactions, have been reported. MLC *via* amido/amine and aromatization–dearomatization are common in catalytic (de)hydrogenation reactions, in addition to the MLC involving metal–borane complexes.^24^ In particular, MLC mechanisms with the cleavage of B–H bond across several bonds, such as benzylic C–Ru,^25^ S–Ru,^26^ S–Os,^27^ amide N–Ru,^17*d*,28,29^ have been described.^30^ Of these, MLC mechanisms for the catalytic hydroboration of CO_2_ have scarcely been reported (Chart 1).^31,18*a*,32,33^ No pyrrolide N–M assisted B–H bond activation has been reported to date as far as we know.

The dipyrromethane-based diphosphines display versatility in forming a variety of main group compounds and homo- and heterometallic complexes in which they adopt both the pincer and non-pincer modes of coordination.^34–36^ Ligands L1H2, L2H2 and L3H2 having phenyl, ethyl and cyclohexyl substituents at the *meso* position form κ^2^ and κ^4^ non-pincer type nickel complexes. Interestingly, the type of nickel complex isolated and their catalytic efficiency in the transfer hydrogenation of ketones depend on the nature of substituents at the *meso* position.^37^ Motivated by these findings, herein we report analogous palladium complexes bearing these ligands and the selective hydroboration of CO_2_ into methoxyborane using these new non-pincer palladium complexes as well as using the reported nickel complexes under mild conditions with mechanistic study involving B–H bond cleavage of 9-BBN.

## Results and discussion

### Synthesis of palladium complexes

**Scheme 1:**
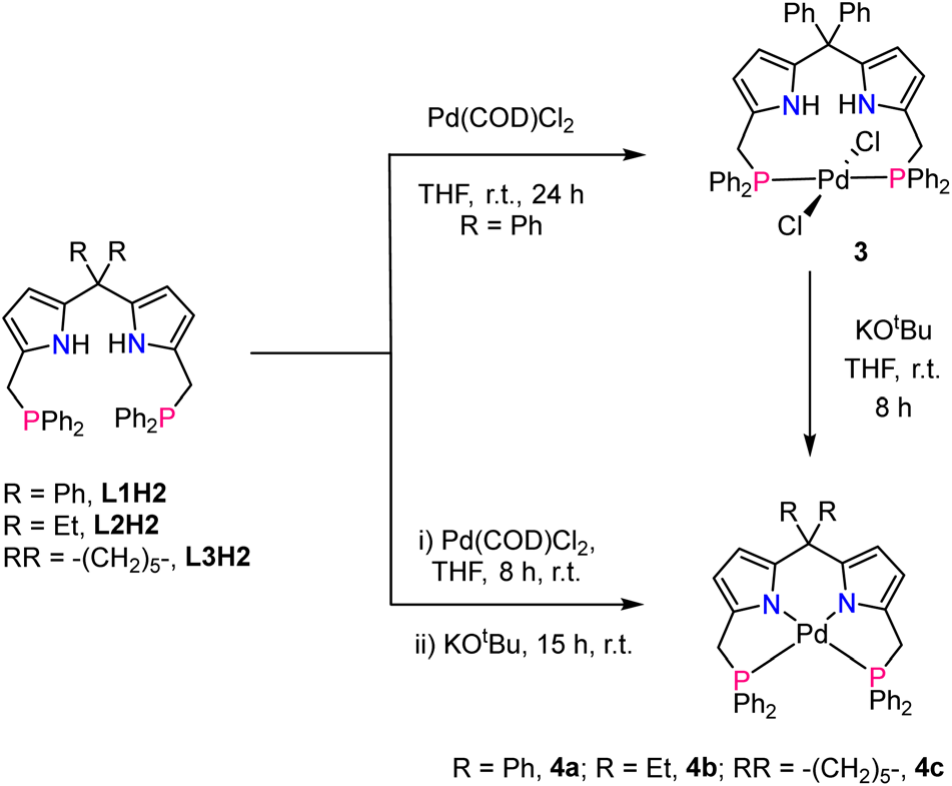
The synthesis of two types of palladium(ii) complexes bearing dipyrromethane-based diphosphines containing different *meso* groups which influence the metalation and hydroboration of CO_2_ properties.

**Fig. 1:**
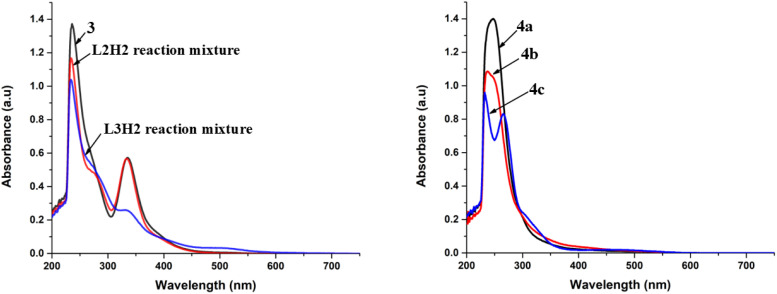
(left side) The UV-vis spectra of complex 3, and of solids obtained from the reactions of L2H2 and L3H2 with [Pd(COD)Cl_2_] in dichloromethane (0.01 mM). (right side) The UV-vis spectra of complexes 4a, 4b and 4c in dichloromethane (0.01 mM).

**Fig. 2:**
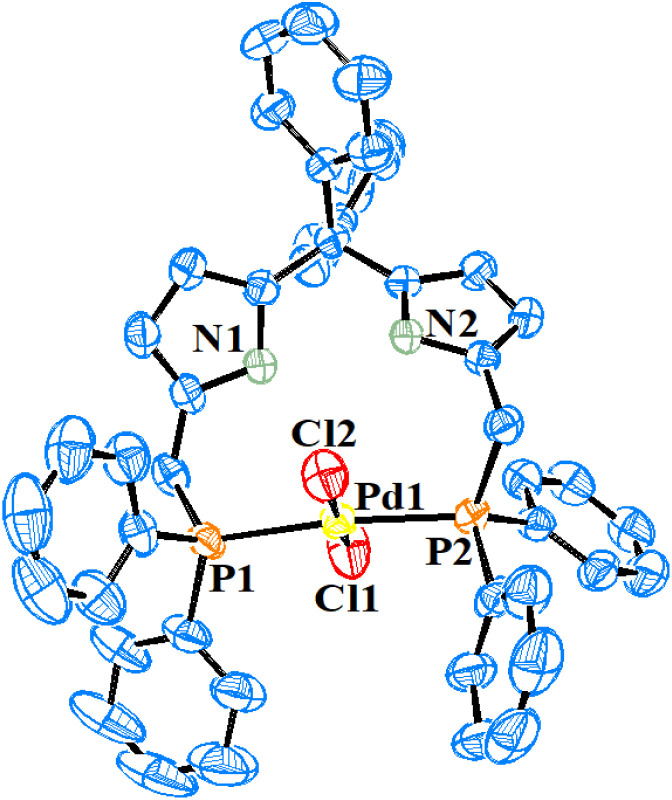
The X-ray structure of complex 3 (50% ellipsoids). All hydrogen atoms are omitted for clarity. Selected bond distances (Å) and bond angles (°): Pd1–P1 2.3257(9), Pd1–P2 2.3321(9), Pd1–Cl1 2.2903(9), Pd1–Cl2 2.2938(9), Cl1–Pd1–Cl2 172.79(4), Cl1–Pd1–P1 87.05(3), Cl2–Pd1–P1 93.00(3), Cl1–Pd1–P2 94.42(3), Cl2–Pd1–P2 86.36(3), P1–Pd1–P2 173.28(3).

The reaction between L1H2 and [Pd(COD)Cl_2_] in THF at room temperature gave the chelated complex 3 in a 89% yield as a yellow solid (Scheme 1). Complex 3 is well soluble in solvents such as CH_2_Cl_2_, CHCl_3_, CH_3_CN, toluene and THF and its structure was confirmed by the single crystal X-ray diffraction method. The X-ray structure of complex 3 is given in Fig. 2 along with important bond distances and angles. Complex 3 crystallizes in the monoclinic space group *P*2_1_/*n* with one molecule in the asymmetric unit. The ligand adopts the chelate κ^2^-*P*,*P*-coordination mode with the *trans* disposition of the phosphorus atoms in a distorted square planar geometry around the palladium(ii) atom containing two chlorine atoms *trans* to each other. The metal center is slightly out of the square plane by 0.005 Å. The whole dipyrromethane moiety lies above the palladium square plane with the P1–Pd1–P2 angle of 173.28(3)°. The structure is similar to the analogous nickel complex^37^ [NiCl_2_(L1H2)] 1 containing the same ligand. This solid-state structure of complex 3 is supported by spectroscopic and HRMS methods. Its ^31^P{^1^H} NMR spectrum displayed a singlet at *δ* 15.3 ppm, which is shifted downfield by 30.8 ppm with respect to the ligand and is also shifted downfield compared to the analogous nickel complex 1 (*δ* 2.65 ppm). The UV-vis spectrum of complex 3 in CH_2_Cl_2_ exhibits an absorption band at 334 nm together with a weak d–d transition around 495 nm (Fig. 1). This spectrum is characteristic of the presence of neutral ligand coordinated to a ‘PdCl_2_’ unit, like the analogous nickel complex 1.

Although our attempts to isolate chelate complexes containing ligands L2H2 and L3H2 similar to complex 3 have failed, the analysis of the ^31^P{^1^H} NMR spectra of solids obtained from the 1 : 1 mole ratio reactions of L2H2 and L3H2 with [Pd(COD)Cl_2_] in THF at room temperature showed the formation of the corresponding chelate complexes resonating at *δ* 15.0 or 16.3 ppm, in addition to minor signals at 29.4 or 29.7 ppm, respectively. In addition, the solid obtained from the reaction of ligand L1H2 with [Pd(COD)Cl_2_] also showed a similar minor signal at *δ* 29.0 ppm along with the major product complex 3. A plausible structure could not be proposed to this minor peak based on HRMS data of these solids. However, to follow the transformation that the minor products would undergo, these reactions were carried out in THF in NMR tubes. Like the isolated solids from the reactions of ligands L1-L3H2, similar sets of signals were observed for these NMR tube reactions in their ^31^P{^1^H} NMR spectra. Subsequently, the addition of two equiv. of KO^*t*^Bu to each reaction mixture in NMR tube led to observing only one signal at *δ* 49.5, 44.5 and 47.0 ppm for reactions with L1-L3H2, respectively. These signals are assigned to the pyrrole nitrogen-coordinated complexes of the type [Pd(κ^4^-*P*,*N*,*N*,*P*-L)] 4 and then they were confirmed by their direct synthesis (see below). These transformations indicate the presence of pyrrole NH groups in the compounds that give minor signals in the ^31^P{^1^H} NMR spectra.

The addition of 2.2 equiv. of KO^*t*^Bu to the reaction mixture of L1-L3H2 and [Pd(COD)Cl_2_] in THF at room temperature afforded complexes 4a–c in very good crystalline yields. In addition, when complex 3 was treated with KO^*t*^Bu in THF, complex 4a was obtained. The ^31^P{^1^H} NMR spectra of 4a–c in CDCl_3_ showed resonances at *δ* 50.3, 45.2 and 47.2 ppm, respectively, for their one phosphorus environments. The characteristic structural feature of complexes 4a–c was indicated by their UV-vis spectra, which gave an intense absorption band in the region of 240–280 nm without an intense absorption around 335 nm which appeared for the palladium chloride complex 3 (Fig. 1).

**Fig. 3:**
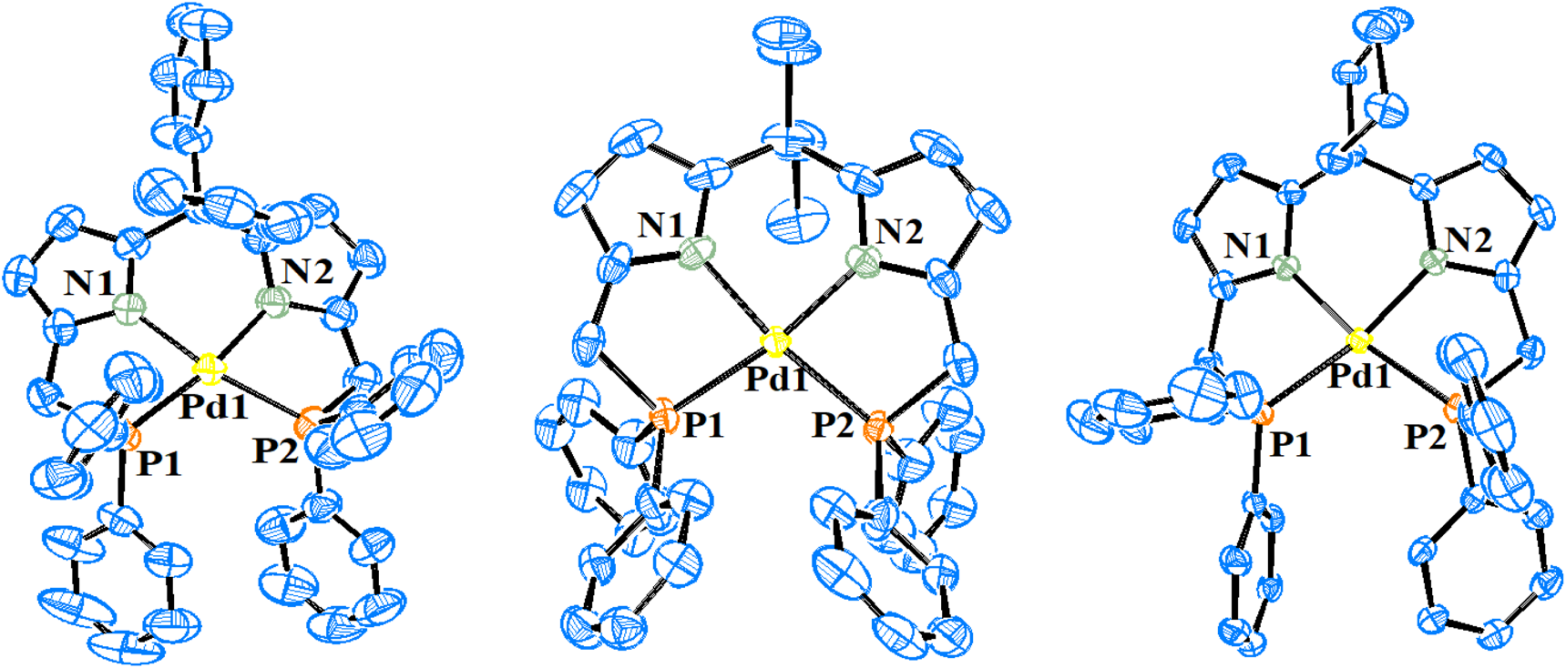
The X-ray structures of complexes 4a–c (50% ellipsoids). All hydrogen and distorted phenyl atoms are omitted for clarity. Selected bond distances (Å) and bond angles (°): for 4a: Pd1–N1 2.0140(16), Pd1–N2 2.0158(16), P1–Pd1 2.2601(5), P2–Pd1 2.2617(5), N1–Pd1–N2 89.91(7), N1–Pd1–P2 170.41(5), N2–Pd1–P2 80.61(5), N1–Pd1–P1 80.79(5), N2–Pd1–P1 170.70(5), P1–Pd1–P2 108.69(2). For 4b: Pd1–N1 2.011(3), Pd1–N2 2.012(4), P1–Pd1 2.2551(11), P2–Pd1 2.2541(11), N1–Pd1–N2 89.36(15), N1–Pd1–P2 171.78(11), N2–Pd1–P2 82.43(11), N1–Pd1–P1 82.03(11), N2–Pd1–P1 171.39(11), P1–Pd1–P2 106.17(4). For 4c: Pd1–N1 2.021(2), Pd1–N2 2.021(3), P1–Pd1 2.2861(8), P2–Pd1 2.2736(8), N1–Pd1–N2 88.88(10), N1–Pd1–P2 169.83(7), N2–Pd1–P2 81.11(7), N1–Pd1–P1 80.52(7), N2–Pd1–P1 169.26(7), P1–Pd1–P2 109.53(3).

The X-ray structures of 4a–c are given in Fig. 3. Complexes 4a and 4c crystallize in the monoclinic space group *P*2_1_/*c*, while complex 4b crystallizes in the orthorhombic space group *Pbca*. The structure revealed that ligands adopt the κ^4^-*P*,*N*,*N*,*P* tetradentate mode in which both the pyrrole nitrogen and phosphorus atoms are coordinated to the palladium atom. This coordination results in the formation of two five-membered and one six-membered chelate rings, giving extra stability to the complex. In all cases, the central six-membered ring is puckered as ligand forms a distorted square planar geometry around the palladium atom. The Pd–P bond distances (2.254(1) to 2.2861(8) Å) in 4a–c are considerably shorter than the distances (2.3321(9) Å and 2.326(1) Å) in the palladium chloride structure 3. This is attributed to the presence of the pyrrolide nitrogens trans to the phosphorus atoms in the former structures, in contrast to the latter structure in which the two phosphorus atoms are trans to each other. The Pd–N distances range from 2.011(3) to 2.021(3) Å, and are slightly longer than the reported Pd–N_pyrrolide_ distances: 1.958(7) Å in the [(NNN)PdCl] complex,^38^ 1.983(5) Å in the [(PNN)PdCl] complex^39^ and 1.981(4) Å in the [(PNP)PdCl] complex.^40^ In addition, the observed Pd–N distances are similar to those (2.046(3) and 2.012(2) Å) observed in the palladium complexes of tetradentate pyrrole Schiff base ligand.^41^

### The hydroboration of CO_2_

The catalytic application of the palladium and nickel complexes 1–4 in the hydroboration of CO_2_ with 9-BBN to selectively give methoxyborane was investigated. At first, a 2 M solution of 9-BBN in toluene containing 0.5 mol% of nickel complex 2a was stirred under 1 atm of CO_2_ for 16 h to observe the formation of bis(boryl)acetal A and methoxyborane B in 14% and 66% NMR yields, respectively, with a TON of 132 for B (Table 1, entry 1). The catalytic reaction in CH_2_Cl_2_ afforded a slightly lower yield of 62% with a TON of 124 for B (Table 1, entry 2). In coordinating solvent THF, the yield of B was decreased to 42% together with 22% of A (Table 1, entry 3). The yield of B (52%) did not improve with 1 mol% of complex 2a in CH_2_Cl_2_ (Table 1, entry 4). The yield of B was slightly improved to 65% when a 1 M solution of 9-BBN in CH_2_Cl_2_ with 0.5 mol% of 2a was used (Table 1, entry 5). This yield was decreased to 34% with a 0.5 M solution of 9-BBN (Table 1, entry 6). Approximately 50% yield of B was obtained in the presence of the other two nickel complexes 2b and 2c (0.5 mol%) containing ethyl and cyclohexyl substituents, indicating the effect of *meso* substituents (Table 1, entries 7 and 8). The catalytic hydroboration reactions using HBPin, HBCat, or H_3_B^.^SMe_2_ in toluene or CH_2_Cl_2_ did not give either A or B, as shown by their ^1^H NMR spectra (Table 1, entries 9–13). This can be due to the hydridic nature of these boranes.^33^ Among these, the hydridic character of H_3_B^.^SMe_3_ is the highest, and as a result, a dark brown solution with precipitate is formed when this borane is used (Table 1, entries 13 and 21), suggesting that the catalyst is decomposed.

**Table 1:** The hydroboration of CO_2_ catalyzed by nickel and palladium complexes 1–4^a^

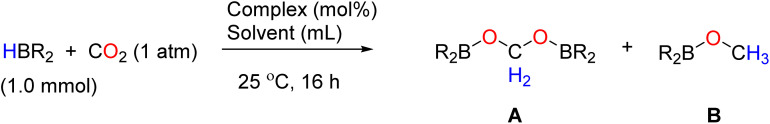						
Entry	Catalyst (mol%)	Solvent (mL)	Conc. of borane (M)	Yield^b^		TON for B^e^
				A	B	
1	2a (0.5)	Toluene (0.5)	9-BBN (2)	14	66	132
2	2a (0.5)	DCM (0.5)	9-BBN (2)	13	62	124
3	2a (0.5)	THF (0.5)	9-BBN (2)	22	42	85
4	2a (1.0)	DCM (0.5)	9-BBN (2)	21	52	52
5	2a (0.5)	DCM (1.0)	9-BBN (1)	9	65	130
6	2a (0.5)	DCM (2.0)	9-BBN (0.5)	28	34	68
7	2b (0.5)	DCM (0.5)	9-BBN (2)	—	47^c^	94
8	2c (0.5)	DCM (0.5)	9-BBN (2)	—	50	100
9	2a (0.5)	Toluene (0.5)	HBpin (2)	—	—	—
10	2a (0.5)	DCM (0.5)	HBpin (2)	—	—	—
11	2a (0.5)	Toluene (0.5)	HBcat (2)	—	—	—
12	2a (0.5)	DCM (0.5)	HBcat (2)	—	—	—
13	2a (0.5)	Toluene (0.5)	BH_3_·SMe_2_ (4)	—	—	—
14	4a (0.5)	Toluene (0.5)	9-BBN (2)	27	55	110
15	4a (0.5)	THF (0.5)	9-BBN (2)	15	60	120
16	4a (0.5)	DCM (0.5)	9-BBN (2)	—	100	200
17	4a (0.5)	Toluene (0.5)	HBpin (2)	—	8	15
18	4a (0.5)	DCM (0.5)	HBpin (2)	—	—	—
19	4a (0.5)	Toluene (0.5)	HBcat (2)	—	4	8
20	4a (0.5)	DCM (0.5)	HBcat (2)	—	—	—
21	4a (0.5)	Toluene (0.5)	BH_3_·SMe_2_ (4)	—	—	—
22	4b (0.5)	DCM (0.5)	9-BBN (2)	—	45^d^	90
23	4c (0.5)	DCM (0.5)	9-BBN (2)	—	48	96
24	2a (0.25)	DCM (0.5)	9-BBN (2)	—	44	177
25	4a (0.25)	DCM (0.5)	9-BBN (2)	23	71	285
26	2a (0.125)	DCM (0.5)	9-BBN (2)	—	35	278
27	4a (0.125)	DCM (0.5)	9-BBN (2)	—	22	174
28	2a (0.5) at 60 °C	Toluene (0.5)	9-BBN (2)	0	39	78
29	2a (0.125) at 60 °C	Toluene (0.5)	9-BBN (2)	0	5	20
30	4a (0.125) at 60 °C	Toluene (0.5)	9-BBN (2)	0	11	44
31	1	DCM (0.5)	9-BBN (2)	—	NR	—
32	1	Toluene (0.5)	9-BBN (2)	—	NR	—
33	1	THF (0.5)	9-BBN (2)	—	3	—
34	3	DCM (0.5)	9-BBN (2)	—	NR	—
35	3	Toluene (0.5)	9-BBN (2)	—	NR	—
36	3	THF (0.5)	9-BBN (2)	15	72	143
37	L1H2	DCM (0.5)	9-BBN (2)	—	2	4
38	L2H2	DCM (0.5)	9-BBN (2)	—	4	8
39	L3H2	DCM (0.5)	9-BBN (2)	—	NR	—
40	[Ni(DME)Cl_2_]	DCM (0.5)	9-BBN (2)	—	NR	—
41	[Pd(COD)Cl_2_]	DCM (0.5)	9-BBN (2)	—	NR	—

a Complex (mol% with respect to HBR_2_) and HBR_2_ (1.00 mmol as a monomer) in solvent (mL) under CO_2_ (1 atm) at 25 °C for 16 h.

b Yields are based on 9-BBN and determined by ^1^H NMR method using 1,1,2,2-tetrachloroethane (36 µL, 0.341 mmol) as an internal standard.

c After 36 h, 18% of A and 52% of B were obtained.

d After 36 h, only B in a 64% NMR yield was obtained.

e TON = [(mmol of methoxy × 3)]/mmol of catalyst.

Next, the catalytic reactions in the presence of analogous palladium complex 4a and different boranes (2 M) in toluene, CH_2_Cl_2_ or THF were carried out. Of these, the hydroboration using 4a (0.5 mol%) and 9-BBN (2 M) in CH_2_Cl_2_ gave B in a quantitative yield with a TON of 200 at 25 °C for 16 h (Table 1, entry 16). Like complex 2a, no or poor catalytic activity was observed in the presence of HBpin, HBcat, or BH_3_·SMe_2_ (Table 1, entries 17–21). In the case of ethyl and cyclohexyl substituted palladium complexes 4b and 4c, an approximately 50% yield of B was obtained in both cases (Table 1, entries 22 and 23). This is similar to nickel complexes 2b and 2c. Since better yields were obtained using complexes 2a and 4a in CH_2_Cl_2_, the loading of these complexes was decreased to 0.25 mol% and it was found that the yield of B was also decreased (Table 1, entries 24 and 25). When the loading of complex 2a or 4a was further decreased (0.125 mol%), the yield of B was also further decreased (Table 1, entries 26 and 27) with unreacted 9-BBN in the reaction mixture. At an elevated temperature of 60 °C, the catalytic activity did not improve (Table 1, entries 28–30), suggesting decomposition of the catalyst at a higher temperature. As a result, unreacted 9-BBN is present in their reaction mixtures after a 16 h reaction. In addition, the catalytic activities of the free ligands and metal precursors were examined under the optimized conditions, and they showed very poor performances (Table 1, entries 37–41), suggesting that the metal complex structure plays a key role in the catalytic activity.

### Mechanism

**Scheme 2:**
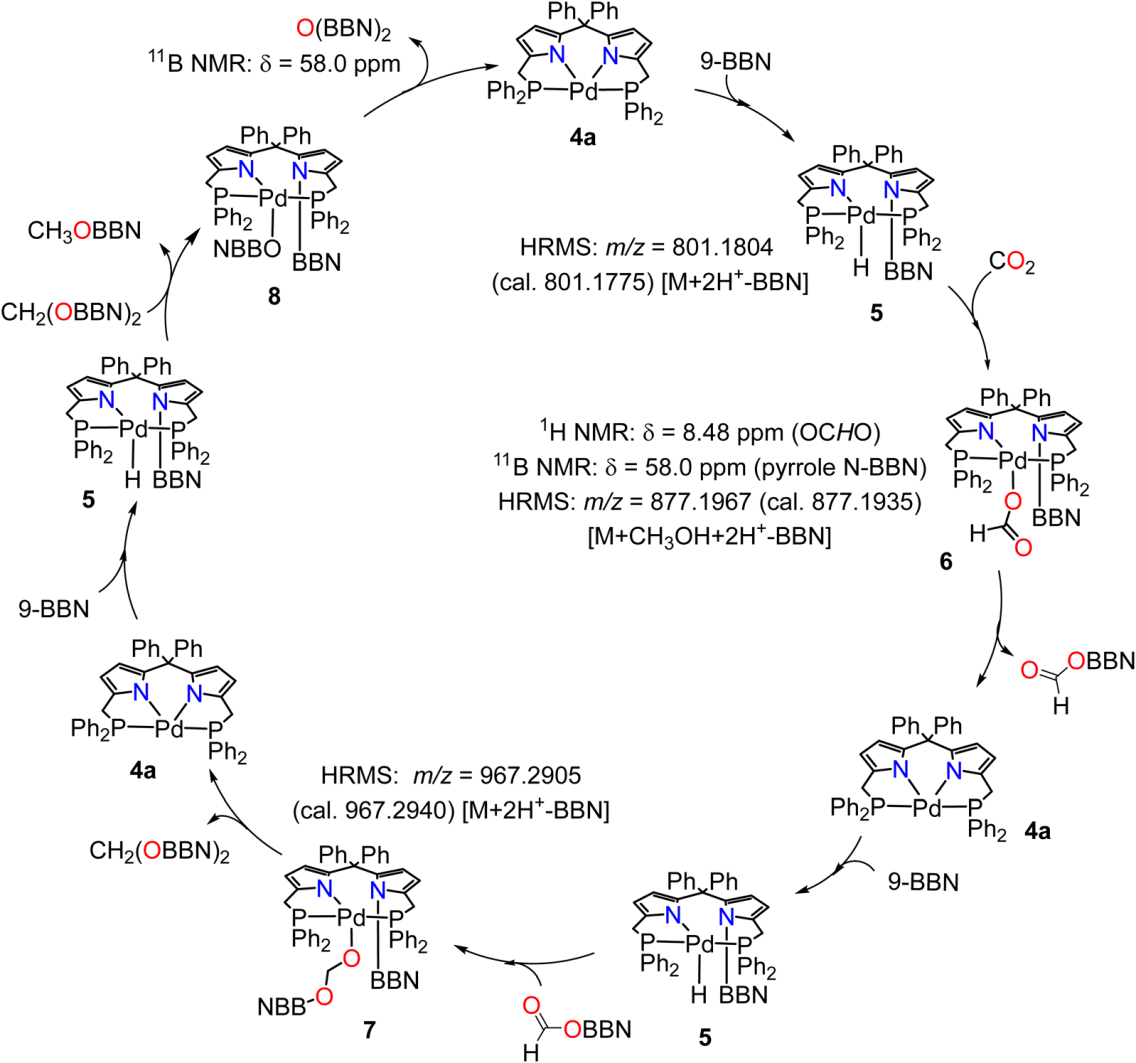
Plausible mechanism for the hydroboration of CO_2_with 9-BBN to methoxyborane catalyzed by palladium complex 4a.

The ^31^P{^1^H} NMR spectrum of the reaction mixture of complex 4a and 2 or 5 equiv. of 9-BBN in toluene or DCM showed only one peak around *δ* 49 ppm due to unreacted complex 4a, suggesting no reaction (see SI, Fig. S127). Its ^11^B NMR spectrum showed a major signal at 27.9 ppm due to unreacted 9-BBN together with a less intense broad signal at *δ* 58.8 ppm, which can be assigned to 9-BBN-OH formation owing to adventitious water (SI, Fig. S128). It is noted this is also the chemical shift position for a compound having a pyrrole N-BBN-9 bond.^42^ Conversely, when this mixture was exposed to CO_2_ (1 atm), a noticeable reaction slowly takes place, as shown by NMR method. It indicates that there can be a synergistic interaction between complex 4a, 9-BBN and CO_2_ by which the B–H bond is activated to reduce CO_2_. The reaction mixture of complex 4a, 9-BBN (5 equiv.) and CO_2_ in DCM was monitored by the NMR method (see SI, Fig. S141–143). After 1 h, a less intense doublet appears at *δ* 49.2 ppm in its ^31^P{^1^H} NMR spectrum. Its ^11^B NMR spectrum shows a broad singlet at *δ* 58.7 ppm, which can be assigned to the hydroborated products A, B, O(BBN)_2_ and also to a pyrrole N-BBN-9 bonded compound, in addition to the signal due to 9-BBN at *δ* 27.6 ppm. After 24 h, its ^1^H NMR spectrum shows a new signal at *δ* 8.48 ppm (see SI, Fig. S138), suggesting the formation of a palladium formate, complex which in turn implies a palladium hydride formation and CO_2_ insertion. In addition, the signal due to the ligand methylene protons at *δ* 3.70 ppm has disappeared and merged with the CH_2_Cl_2_ signal, suggesting that the reaction occurred on the ligand framework, preferably at one of the pyrrole N or methylene carbon atom sites, which can render complex 4a to have two phosphorus environments. As a result, importantly, its ^31^P{^1^H} NMR spectrum (see SI, Fig. S139) shows two new signals: one centered at *δ* 49.1 ppm as a doublet of doublets with *J*_(P,P)_ = 113.4 and 4.9 Hz and another signal at *δ* 48.4 ppm as a broad triplet with *J*_(P,P)_ = 18.6 Hz, in addition to the starting complex 4a at *δ* 49.6 ppm. This suggests the formation of two new complexes, each likely having two phosphorus environments, and since these signals appear around 49 ppm, these newly formed species can be pyrrole N-coordinated palladium complexes of the type 5 shown in Scheme 2. Its ^11^B NMR spectrum shows only one signal at *δ* 58.0 ppm. Its HRMS (^+^ESI) spectrum gives peaks *m*/*z* at 801.1804 (calc. 801.1775) for a palladium hydride [(L1H-κ^3^-*P*,*N*,*P*)Pd(H) + H]^+^ and *m*/*z* at 877.1967 (calc. 877.1935) for a palladium formate [(L1H-κ^3^-*P*,*N*,*P*)Pd(OCHO) + H + CH_3_OH]^+^, both of them formed after the B–N bond hydrolysis when the HRMS sample was prepared.

Based on the above analysis, the mechanism could first involve the formation of palladium hydride complex 5 by the σ-bond metathesis reaction between 9-BBN and complex 4a (Scheme 2). This type of addition of a B–H bond across a Pd-amide bond has been reported by Ozerov^43^ as well as in other complexes.^25,44^ Next, the palladium hydride reacts with CO_2_ to form the formate complex 6, which transfers its formyl group to the 9-BBN unit present in the same molecule to form boryl formate and to regenerate the catalyst 4a, which is present after CO_2_ exposure for 24 h. Next, palladium hydride 5 reduces boryl formate to the palladium bound acetal derivative 7, which then transfers the acetal to the BBN group present in the same molecule to give the acetal CH_2_(OBBN)_2_. This acetal gets reduced by palladium hydride 5 to generate the final product methoxyborane together with boryloxy–palladium complex 8 from which O(BBN)_2_ can be eliminated to regenerate catalyst 4a. This mechanism can be regarded as metal–ligand cooperation, as the B–H bond is cleaved across the metal and pyrrolide nitrogen bond, and the catalyst is regenerated by eliminating the hydroborated products. This mechanism is similar to those reported by Leitner,^18*a*^ Liao,^45^ Wang,^17*d*^ and co-workers.

**Fig. 4:**
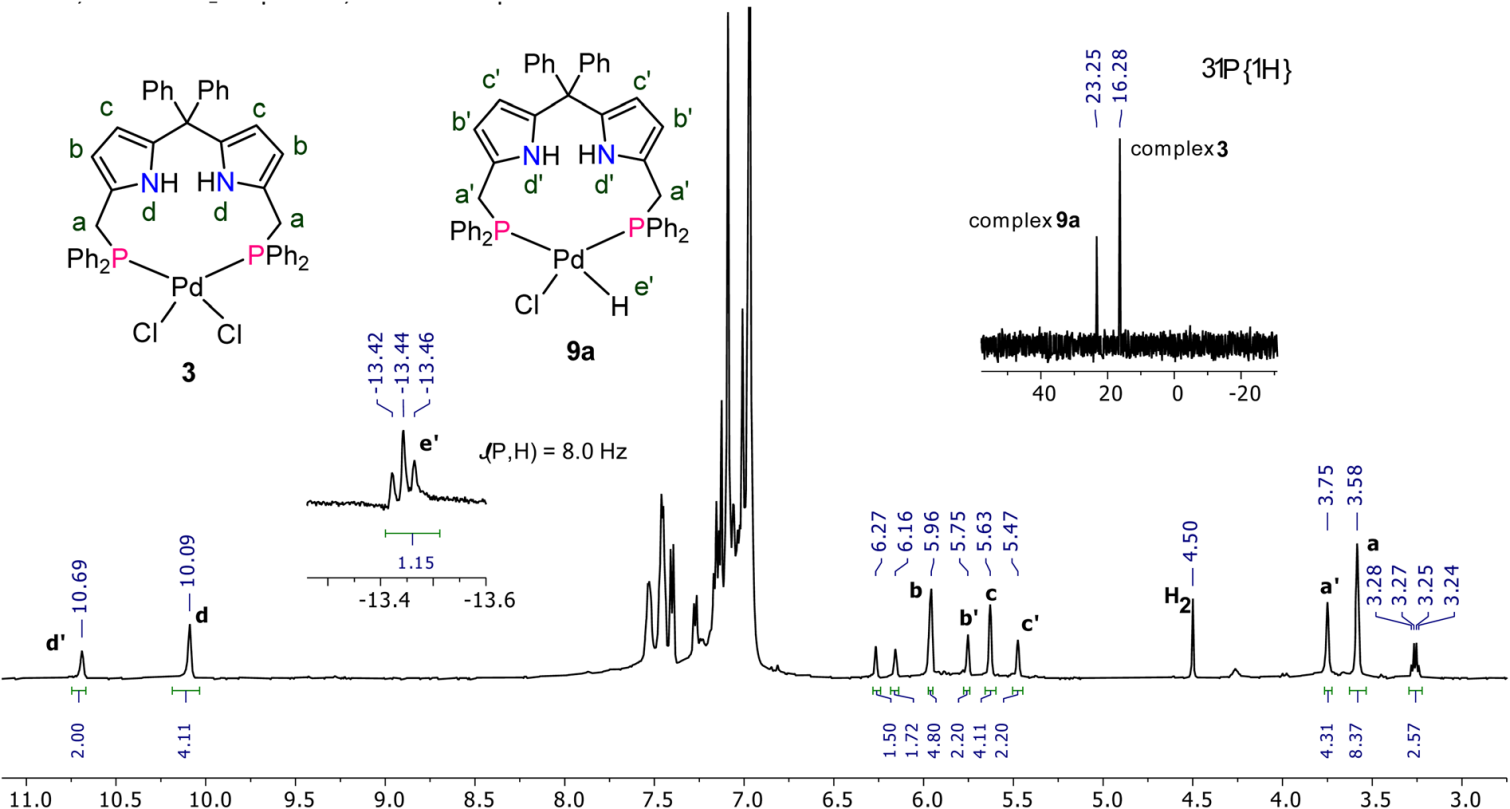
Partial ^1^H NMR (400 MHz, 25 °C.) spectrum of the reaction between complex 3 and 9-BBN (5 equiv. as a monomer) in toluene-*d*_8_ without CO_2_ exposure, indicating the formation of palladium hydride 9a. Signals are tentatively assigned based on the value of integrated intensities and peak positions. Inlet shows its ^31^P{^1^H} NMR spectrum.

**Scheme 3:**
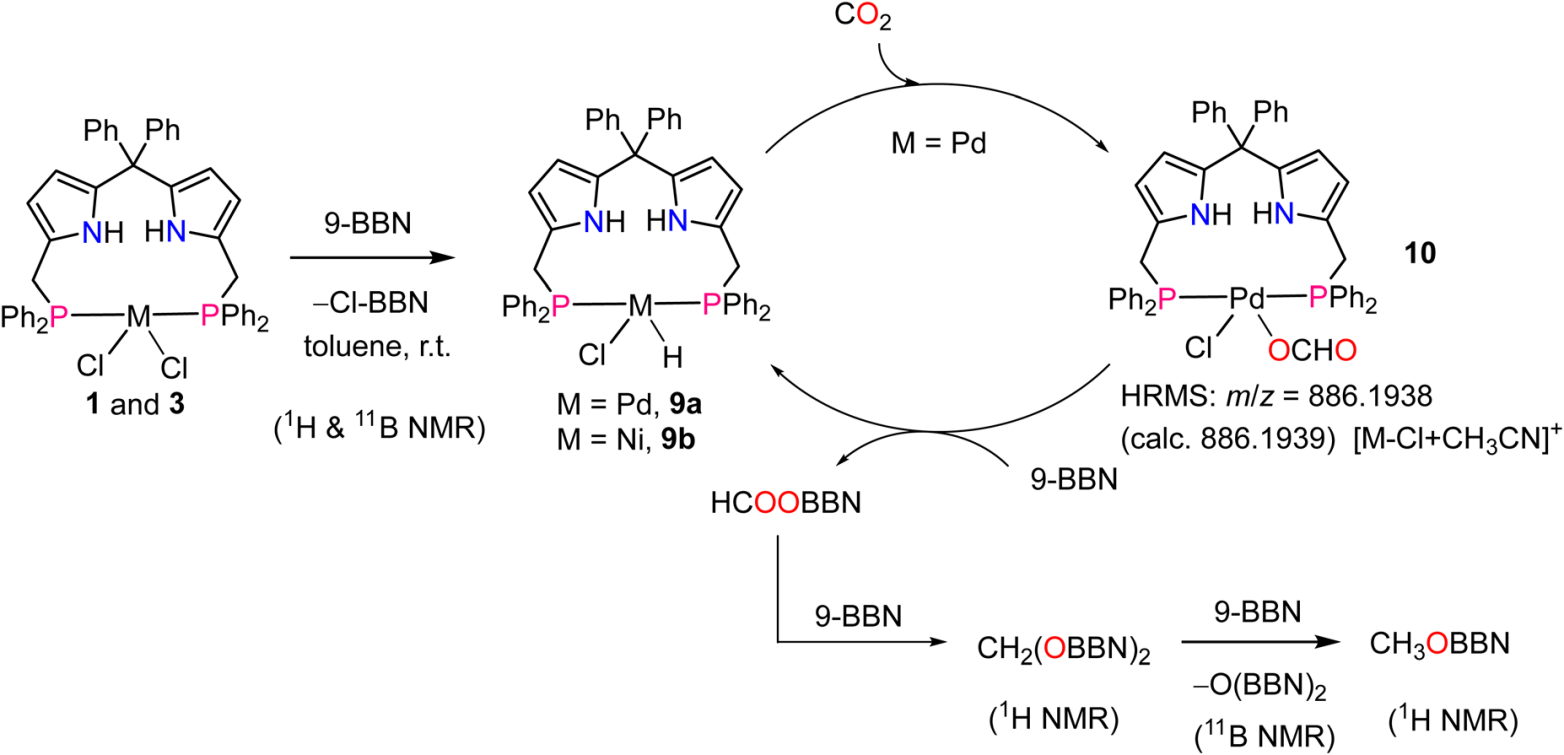
The formation of nickel and palladium hydrides in the reactions of complexes 1 and 3 with 9-BBN and their proposed mechanism for the hydroboration of CO_2_ (1 atm) catalyzed by the palladium hydride 9a at 25 °C.

Further, the catalytic hydroboration in the presence of nickel chloride complex 1 did not afford the desired product A or B (Table 1, entries 31–33). However, the palladium chloride complex 3 gave a better yield of 72% of B in THF compared to in other solvents (Table 1, entries 34–36). To understand the course of this catalytic reaction, the reaction between complex 3 and 5 equiv. of 9-BBN was carried out in toluene-*d*_8_ in an NMR tube. As given in Fig. 4, its ^1^H NMR spectrum features a triplet at *δ* −13.4 ppm (*J*_(P,H)_ = 8.0 Hz), a singlet at *δ* 10.69 ppm together with a signal at *δ* 4.50 ppm for H_2_ gas. Its ^31^P{^1^H} NMR spectrum shows a new singlet at *δ* 23.2 ppm along with the signal due to the starting complex 3. Its ^11^B NMR spectrum displays a broad signal at *δ* 81.6 ppm for the formation of 9-BBN-Cl as a by-product.^46^ The integrated intensities and chemical shift positions suggests the formation of palladium hydride 9a containing two pyrrole NH groups in solution by the equimolar reaction between complex 3 and 9-BBN with the elimination of one equiv. of 9-BBN-Cl (Scheme 3). After the CO_2_ exposure, the reaction mixture contains the hydride species 9a among other peaks, as shown by its ^1^H and ^31^P NMR spectra (see SI, Fig. S156 and S158). Gratifyingly, its HRMS (ESI^+^) spectrum showed a peak at *m*/*z* 886.1938 (calc. 886.1939) for the formate complex 10 (Fig. S160).

**Fig. 5:**
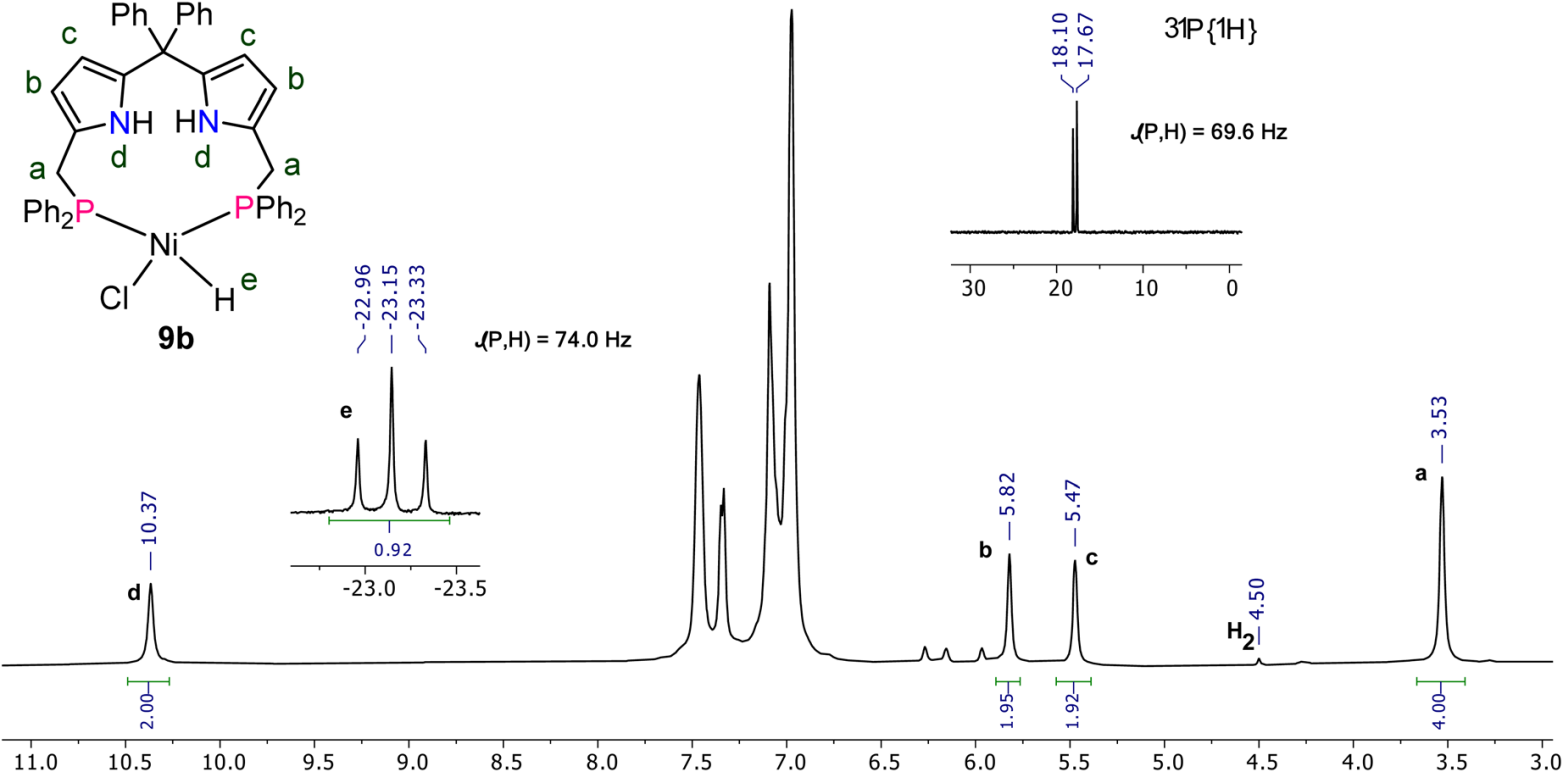
Partial ^1^H NMR (400 MHz, 25 °C) spectrum of the reaction between nickel chloride complex 1 and 9-BBN (5 equiv. as a monomer) in toluene-*d*_8_ without CO_2_ exposure, indicating the formation of nickel hydride 9b. Signals are tentatively assigned based on the value of integrated intensities and peak positions. Inlet shows its ^31^P{^1^H} NMR spectrum.

Like the PdCl_2_ complex, the NiCl_2_ complex 1 undergoes a similar reaction with 9-BBN. The ^1^H NMR spectrum of the reaction between complex 1 and 5 equiv. of 9-BBN in toluene-*d*_8_ features a triplet at *δ* −23.1 ppm (*J*_(P,H)_ = 74.0 Hz), and a singlet at *δ* 10.37 ppm together with predominant signals of the pyrrole CH and methylene protons (Fig. 5). Its ^31^P{^1^H} NMR spectrum shows a doublet at *δ* 17.9 ppm, (*J*_(P,H)_ = 69.6 Hz) (Fig. 5), suggesting the presence of only one compound. Its ^11^B NMR spectrum displays a broad signal at *δ* 81.3 ppm for the formation of 9-BBN-Cl as a by-product (see SI, Fig. 163).^46^ However, after CO_2_ exposure, ^1^H NMR spectrum shows no product (A or B) formation, which can be due to decomposition caused by an excess 9-BBN and is consistent with the poor activity observed with the nickel chloride complex 1.

From the above analysis of NMR spectra, the formation of Pd and Ni hydrides from complexes 3 and 1 and their mechanisms for the hydroboration of CO_2_ are given in Scheme 3. The palladium hydride 9a and nickel hydride 9b are formed by the substitution of chloride with hydride led to the elimination of 9-BBN-Cl when complexes 3 and 1 react with 9-BBN. As ^1^H and ^31^P{^1^H} NMR spectra showed the presence of palladium hydride before and after CO_2_ exposure, complex 9a can be the resting state of the catalysis. Complex 9a undergoes the CO_2_ insertion to give the formate complex 10. The formyl group is transferred to 9-BBN to form formoxyborane, which is reduced further by 9-BBN to eventually give methoxyborane B.

## Conclusions

Following the report of the dipyrromethane–diphosphine supported nickel complexes 1 and 2, the palladium metal also gives similar complexes whose synthesis and characterizations by both spectroscopic and X-ray methods were established in this study. In addition, upon examining the activities of both nickel and palladium complexes in the hydroboration of CO_2_ (1 atm), the palladium complex 4a containing the phenyl groups performed better than the corresponding palladium complexes with ethyl and cyclohexyl groups, suggesting that substituents at the *meso* carbon of the dipyrromethane have a key role of steric influence on catalytic activities. A similar *meso* group effect was observed in the transfer hydrogenation of various ketones catalyzed by nickel complexes 1 and 2.^37^ In the case of palladium complex 4 containing the tetradentate mode of ligand, the proposed mechanism involves a B–H bond activation across the metal and ligand framework followed by CO_2_ insertion to yield the formate complex which is further reduced to the final product methoxyborane *via* an acetal product, as supported by ^1^H NMR and HRMS methods. Furthermore, the dipyrromethane–diphosphine supported new nickel and palladium hydrides 9 formed by the reactions of 9-BBN were characterized by NMR methods. The hydroboration property of complex 3 containing the bidentate mode of ligand can be due to the *in situ* generation of its palladium hydride species. Our ongoing research involves isolation of B–H bond activated metal complexes containing dipyrromethane–diphosphine ligands for catalysis.

## Experimental section

### Synthesis of [PdCl_2_{Ph_2_C(C_4_H_3_N)_2_-1,9-(CH_2_PPh_2_)_2_-κ^2^-*P*,*P*}], 3

To a solution of ligand L1H2 (0.200 g, 0.287 mmol) in THF (20 mL) was added [Pd(COD)Cl_2_] (0.082 g, 0.287 mmol). The reaction mixture was stirred for 24 h at room temperature. The solvent was removed under vacuum to give a yellow-colored precipitate, which was washed with diethyl ether (2 × 10 mL) and dried under vacuum to give complex 3 as a yellow solid (0.223 g, 0.255 mmol, yield: 89%). The suitable single crystals of 3 were obtained by layering a solution of yellow solid in CH_2_Cl_2_ with hexanes at room temperature. ^1^H NMR (CDCl_3_, 400 MHz): *δ* = 9.63 (br s, 2H, NH), 7.55–7.28 (m, 26H, phenyl CH), 7.17–7.14 (m, 4H, phenyl CH), 5.77 (t, ^3^*J*_HH_ = 4.0, 2H, pyrrole β-CH), 5.72 (m, 2H, pyrrole β-CH), 3.92 (t, ^3^*J*_HH_ = 4.0, 4H, CH_2_). ^13^C{^1^H} NMR (125.75 MHz, CDCl_3_): 146.2, 136.5 (s, phenyl C), 133.9 (t, *J*_CP_ = 5.7, phenyl C), 130.9, 129.8, 129.7, 129.6, 129.5, 128.2 (t, *J*_CP_ = 5.0, pyrrole α-C), 127.7, 126.9, 110.8 (s, pyrrole β-C), 110.0 (t, *J*_CP_ = 3.0, pyrrole β-C), 56.3 (s, *meso* C), 26.6 (t, *J*_CP_ = 13.4, CH_2_). ^31^P{^1^H} NMR (162.04 MHz, CDCl_3_): *δ* = 15.3 (s). HRMS (^+^ESI): *m*/*z* calc. for [M − 2Cl + H]^+^ [C_47_H_41_N_2_PdP_2_]^+^ 801.1775, found: 801.1756. Anal. calcd for C_47_H_40_Cl_2_N_2_PdP_2_: C, 64.73; H, 4.62; N, 3.21. Found = C, 64.69; H, 4.80; N, 3.07.

### Synthesis of [Pd{Ph_2_C(C_4_H_2_N)_2_-1,9-(CH_2_PPh_2_)_2_-κ^4^-*P*,*N*,*N*,*P*}], 4a

#### Method a

To a solution of ligand L1H2 (0.100 g, 0.144 mmol) in THF (15 mL) was added [Pd(COD)Cl_2_] (0.041 g, 0.144 mmol) and stirred at room temperature for 8 h. Then KO^*t*^Bu (0.036 g, 0.316 mmol) was added, resulting in the formation of an orange-colored solution after 1 h. The stirring was continued for an additional 16 h. All volatilities were removed under vacuum and the resulting residue was washed with degassed H_2_O (3 × 10 mL) to give an orange-red solid. The solid was dissolved in dichloromethane (20 mL), the resulting solution was dried over anhydrous magnesium sulfate for 30 min, and then filtered through a frit. All volatilities were removed again under vacuum to give a yellow solid which was washed with diethyl ether (2 × 5 mL) and dried under vacuum to give complex 4a (0.101 g, 0.126 mmol, yield: 88%). Complex 4a was crystallized by layering its THF solution with petroleum ether at 25 °C.

### From complex 3

#### Method b

A Schlenk flask was charged with complex 3 (0.600 g, 0.387 mmol) and KO^*t*^Bu (0.117 g, 1.582 mmol) in THF (50 mL). The resulting solution was stirred at room temperature for 8 h. The solvent was removed under vacuum to give solid residue, which was washed with degassed water (3 × 20 mL). The residue was dissolved in CH_2_Cl_2_ (50 mL), and the solution was dried over anhydrous magnesium sulfate for 2 h and then filtered using a frit. The solvent was again removed under vacuum to give a yellow solid, which was washed with diethyl ether (1 × 15 mL) and dried under vacuum to give complex 4a (0.504 g, 0.632 mmol, yield: 92%). ^1^H NMR (CDCl_3_, 400 MHz): 7.31 (t, ^3^*J*_HH_ = 5.0, 4H, phenyl CH), 7.26–7.07 (m, 22H, phenyl CH), 6.93 (d, ^3^*J*_HH_ = 5.0, 4H, phenyl CH), 5.99 (d, 2H, ^3^*J*_HH_ = 3.0, pyrrole β-CH), 5.62 (t, 2H, ^3^*J*_HH_ = 2.2 pyrrole β-CH), 3.80 (m, 4H, CH_2_). ^13^C{^1^H} NMR (125.75 MHz, CDCl_3_): 149.7, 139.6 (s, phenyl C), 132.8 (t, *J*_CP_ = 6.0, phenyl C), 132.7 (t, *J*_CP_ = 4.4, phenyl C), 130.9, 130.1, 129.4, 129.1, 128.9 (t, *J*_CP_ = 5.3, pyrrole α-C), 126.9, 125.1 (s, pyrrole α-C), 110.5 (s, pyrrole β-C), 102.2 (t, *J*_CP_ = 8.1, pyrrole β-C), 60.8 (s, *meso* C), 34.6 (m, CH_2_). ^31^P{^1^H} NMR (202.45 MHz, CDCl_3_): *δ* = 50.3 (s). HRMS (^+^ESI): *m*/*z* calc. for [M + H]^+^ [C_47_H_39_N_2_PdP_2_]^+^ 799.1618, found: 799.1631. Anal. calcd for C_47_H_39_N_2_PdP_2_: C, 70.64; H, 4.79; N, 3.51. Found = C, 70.85; H, 4.66; N, 3.59.

### Synthesis of [Pd{Et_2_C(C_4_H_2_N)_2_-1,9-(CH_2_PPh_2_)_2_-κ^4^-*P*,*N*,*N*,*P*}], 4b

It was synthesized by following the procedure of synthesis of 4a (method a) using ligand L2H2 (0.200 g, 0.334 mmol), [Pd(COD)Cl_2_] (0.0953 g, 0.334 mmol) and KO^*t*^Bu (0.0825 g, 0.735 mmol). Complex 4b was obtained as a red solid (0.205 g, 0.291 mmol, yield: 87%). A solution of complex 4b in THF (10 mL) was layered with petroleum ether (25 mL) to give suitable single crystals. ^1^H NMR (CDCl_3_, 500 MHz): *δ* = 7.34–7.27 (m, 12H, phenyl CH), 7.16–7.12 (m, 8H, phenyl CH) 6.15 (d, *J*_HH_ = 4.0, 2H, pyrrole β-CH), 6.09 (d, *J*_HH_ = 4.0, 2H, pyrrole β-CH), 3.81 (t, ^2^*J*_PH_ = 6.6, 4H, CH_2_), 1.93 (q, 4H, ^3^*J*_HH_ = 7.4, CH_2_CH_3_), 0.74 (t, 6H, ^3^*J*_HH_ = 4.0, CH_2_CH_3_). ^13^C{^1^H} NMR (125.75 MHz, CDCl_3_): 138.4 (s, phenyl C), 132.7 (t, *J*_CP_ = 6.3, phenyl C), 131.8 (t, *J*_CP_ = 4.4, phenyl C), 131.0, 130.5, 130.4, 130.2, 130.0, 128.8 (t, *J*_CP_ = 5.0, pyrrole α-C), 103.8 (t, *J*_CP_ = 3.1, pyrrole β-C), 102.8 (t, *J*_CP_ = 3.8, pyrrole β-C), 50.8 (s, *meso* C), 39.7 (s, CH_2_CH_3_), 10.8 (s, CH_2_CH_3_). ^31^P{^1^H} NMR (202.45 MHz, CDCl_3_): *δ* = 45.2 (s). HRMS (^+^ESI): *m*/*z* calc. for [M + H]^+^ [C_39_H_39_N_2_PdP_2_]^+^ 703.1618, found: 703.1604. Anal. calcd for C_39_H_39_N_2_PdP_2_: C, 66.62; H, 5.45; N, 3.98. Found = C, 66.47; H, 5.65; N, 3.95.

### Synthesis of [Pd{-(CH_2_)_5_-C(C_4_H_2_N)_2_-1,9-(CH_2_PPh_2_)_2_-κ^4^-*P*,*N*,*N*,*P*}], 4c

This complex was synthesized by following the procedure of synthesis of 4a (method a) using L3H2 (0.200 g, 0.327 mmol), [Pd(COD)Cl_2_] (0.0935 g, 0.327 mmol) and KO^*t*^Bu (0.081 g, 0.721 mmol). Complex 4c was obtained as a red solid (0.180 g, 0.251 mmol, yield: 77%). A solution of complex 4c in THF (10 mL) was layered with petroleum ether (25 mL) to give suitable single crystals. ^1^H NMR (CDCl_3_, 400 MHz): *δ* = 7.39–7.33 (m, 12H, phenyl CH), 7.20–7.15 (m, 8H, phenyl CH), 6.16–6.14 (m, 2H, pyrrole β-CH), 5.96 (d, *J*_HH_ = 3.03, 2H, pyrrole β-CH), 3.83–3.80 (m, ^2^*J*_PH_ = 10.9, 4H, CH_2_), 2.23 (t, *J*_HH_ = 5.9, 4H, cyclohexyl CH), 1.62–1.59 (m, 4H, cyclohexyl CH), 1.44–1.40 (m, 2H, cyclohexyl CH). ^13^C{^1^H} NMR (125.75 MHz, CDCl_3_): 143.3, 132.8 (t, *J*_CP_ = 6.3, phenyl C), 132.4, 130.9, 130.2 (s, pyrrole α-C), 129.8 (s, pyrrole α-C), 128.9 (t, *J*_CP_ = 5.0, phenyl C), 104.7 (s, pyrrole β-C), 101.3 (t, *J*_CP_ = 7.5, pyrrole β-C), 68.1, 43.2 (s, *meso* C), 27.0(d, *J*_CP_ = 3.8, cyclohexyl C), 25.7, 23.4 25.8 (s, cyclohexyl C). ^31^P{^1^H} NMR (162.04 MHz, CDCl_3_): *δ* = 47.2 (s). HRMS (^+^ESI): *m*/*z* calc. for [M + H]^+^ [C_40_H_39_N_2_NiP_2_]^+^ 715.1618, found: 715.1606. Anal. calcd for C_40_H_39_N_2_NiP_2_: C, 67.18; H, 5.36; N, 3.92. Found = C, 66.99; H, 5.37; N, 3.83.

### Typical procedure for CO_2_ hydroboration

Inside a glovebox, 9-BBN (0.122 g, 1.00 mmol as a monomer) and complex 2a (0.0037 mg, 0.005 mmol) were taken in a 25 mL Schlenk flask equipped with a magnetic stirrer and then toluene or CH_2_Cl_2_ (0.5 mL) was added. The flask was closed, taken out of the glove box, and then connected to a carbon dioxide balloon (1 atm) *via* a condenser, adapter, and a three-way stopcock. The reaction mixture was subjected to the freeze–pump–thaw method to remove dissolved gases, and the flask was filled with carbon dioxide. The reaction mixture was stirred for 16 h under the CO_2_ atmosphere. 1,1,2,2-tetrachloroethane (0.036 mL, 0.341 mmol) as an internal standard was added, and the reaction mixture was analysed by NMR method.

## Author contributions

The manuscript was written through contributions of all authors. All authors have given approval to the final version of the manuscript. G. M. conceived the whole work and supervised and directed R. G. to carry out all experiments. R. G. and A. K. collected all experimental data.

## Conflicts of interest

There are no conflicts to declare.

## Supplementary Material

RA-OLF-D6RA04348H-s001

RA-OLF-D6RA04348H-s002

## Data Availability

CCDC 2548265–2548268 contain the supplementary crystallographic data for this paper.^47*a*–*d*^ Supplementary information (SI): general experimental section, NMR, ATR-FTIR, HRMS, and crystal structure refinement data. See DOI: https://doi.org/10.1039/d6ra04348h.
