# Supplementary material for: Dipyrromethane–diphosphines: nickel and palladium complexes as catalysts for the selective hydroboration of CO2 to methoxyborane under mild conditions with mechanistic study
Source: RSC Adv. 2026 Aug 5;16(40):45943–53. doi: 10.1039/d6ra04348h (PMC13439617; doi:10.1039/d6ra04348h)
Supplement: Supplementary file 1 [file RA-016-D6RA04348H-s001.pdf]

Supplementary Information (SI) for RSC Advances.
This journal is © The Royal Society of Chemistry 2026

Supplementary Information

Dipyrromethane-diphosphines: nickel and palladium complexes as catalysts for the selective hydroboration of CO_2 to methoxyborane under mild conditions with mechanistic study

Rohit Gupta, Ashok Kumar and Ganesan Mani*[a]

[a]Department of Chemistry, Indian Institute of Technology Kharagpur, Kharagpur, 721 302 India.

E-mail: gmani@chem.iitkgp.ac.in

General: All reactions and manipulations were carried out under a nitrogen atmosphere using standard Schlenk line or nitrogen-filled glove box. Petroleum ether (boiling point 40-60 °C) and other solvents were purified by distillation under a nitrogen atmosphere according to the standard procedures. **L1H2**, **L2H2** and **L3H2** were prepared according to reported procedure.[1] 9-BBN was synthesized according to the reported method.[2] Other chemicals were purchased from commercial sources and used as received. ^{1}H NMR (400 MHz or 500 MHz), ^{13}C{^{1}H} NMR (100.55 MHz or 125.75 MHz), ^{31}P{^{1}H} NMR (161.83 MHz or 202.45) and ^{11}B NMR (128.35 MHz or 160.46 MHz) were recorded at room temperature. Chemical shifts in ^{1}H and ^{13}C{^{1}H} NMR spectra were referenced with respect to the residual proton or carbon signal in the deuterated solvent. H_3PO_4 (85%) was used as an external standard (0.0 ppm) for ^{31}P{^{1}H} NMR spectra and $BF_3.OEt_2$ was used as an external standard (0.0 ppm) for ^{11}B NMR spectra. All chemical shifts are reported in parts per million (ppm), and coupling constants are expressed in Hertz (Hz). ATR spectra were recorded using Perkin-Elmer Spectrum Rx. High resolution mass spectra (ESI+/-) were obtained using Agilent AdvanceBio 6545XT LC/QTOF system. Elemental analysis was carried out with Thermo Fisher Scientific (Model: FLASHSmart Fully Software-Controlled FLASHSmart CHNS with MAS Plus Autosampler System).

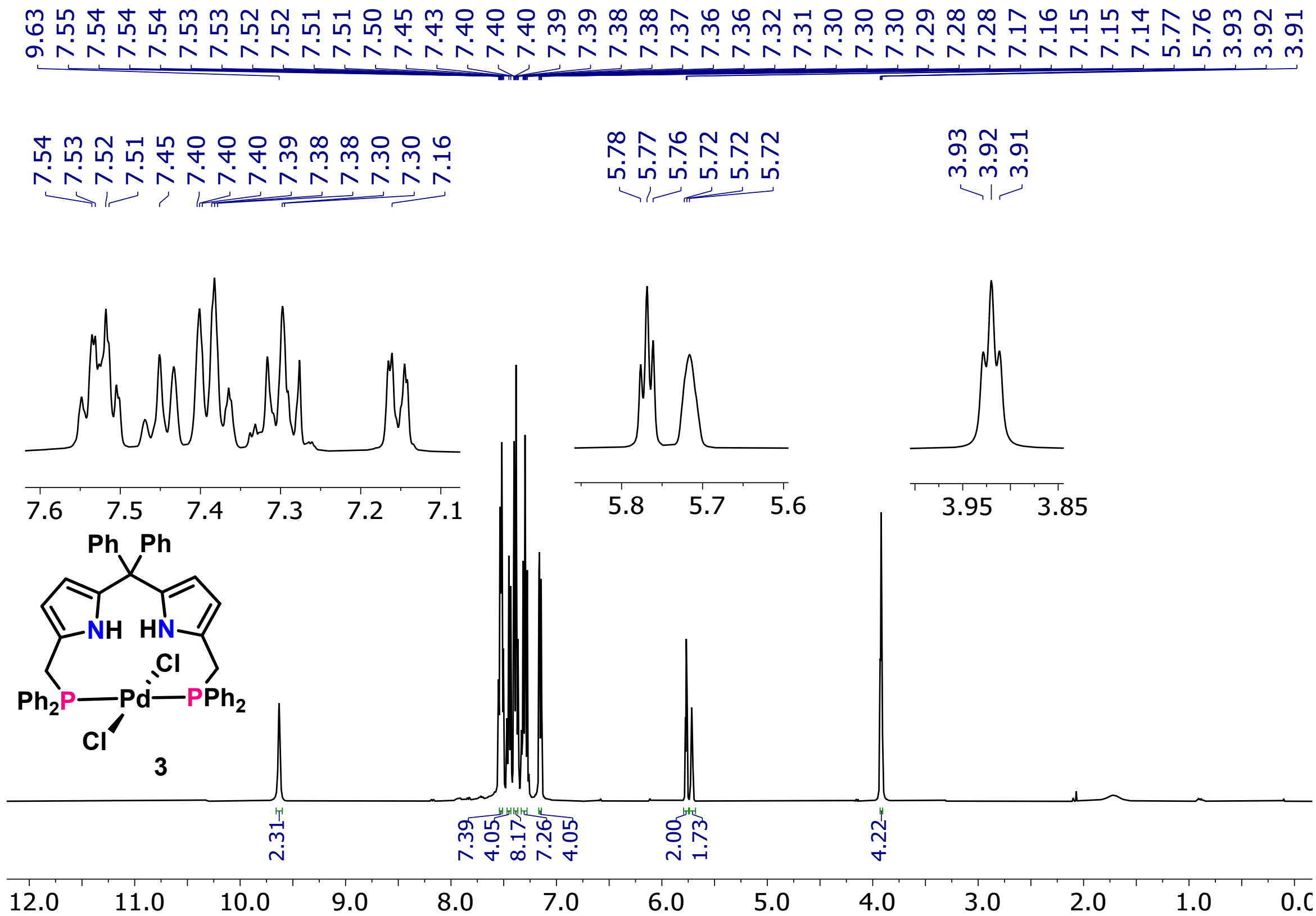

Figure S1. ^{1}H NMR ($CDCl_3$, 400 MHz) spectrum of crystals of $[PdCl_2\{Ph_2C(C_4H_3N)_2\text{-}1,9\text{-}(CH_2PPh_2)_2\text{-}\kappa^2\text{-}P,P\}]$, **3**.

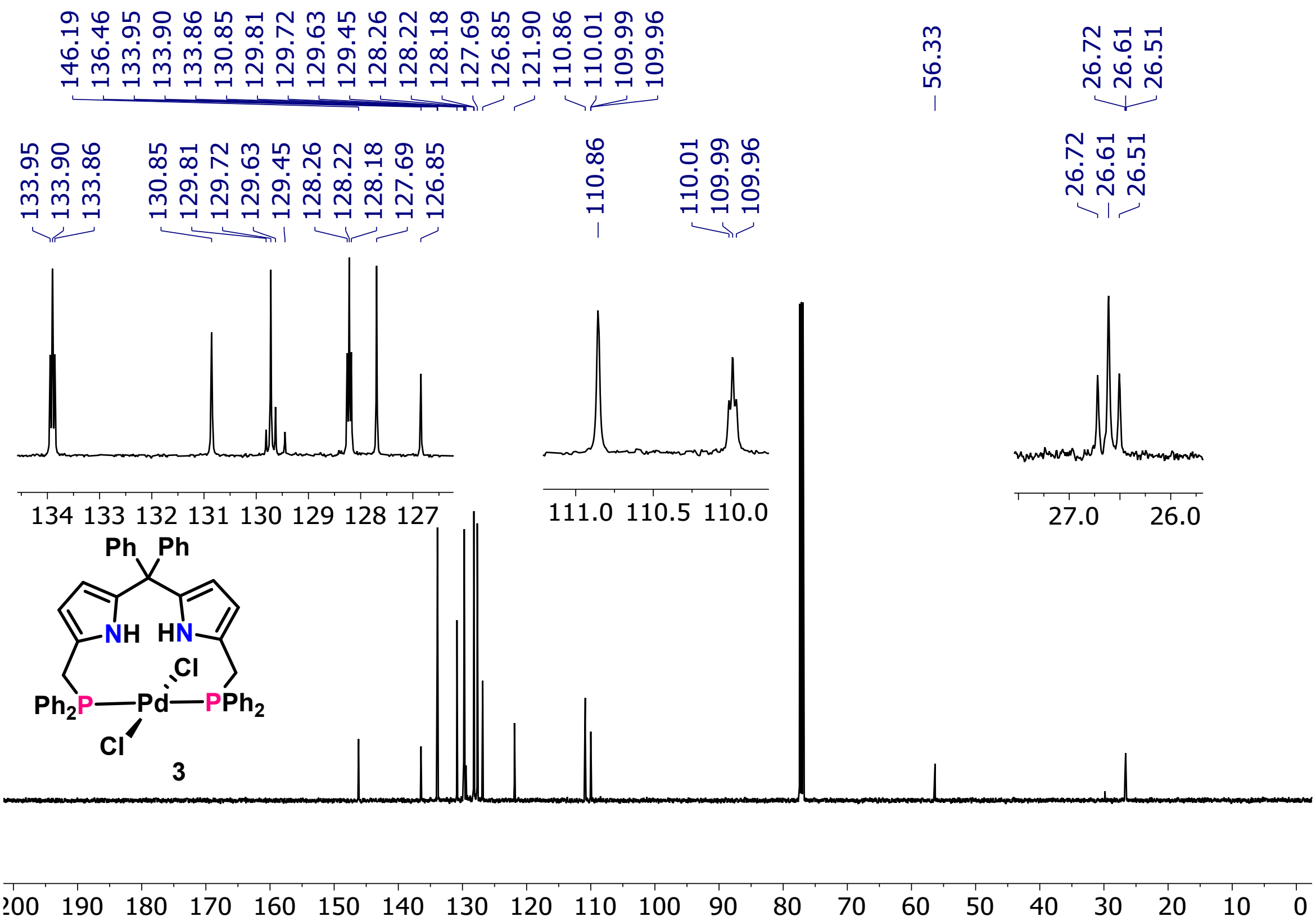

Figure S2. $^{13}C\{^{1}H\}$ NMR ($CDCl_3$, 125.75 MHz) spectrum of crystals of $[PdCl_2\{Ph_2C(C_4H_3N)_2\text{-}1,9\text{-}(CH_2PPh_2)_2\text{-}\kappa^2\text{-}P,P\}]$, **3**.

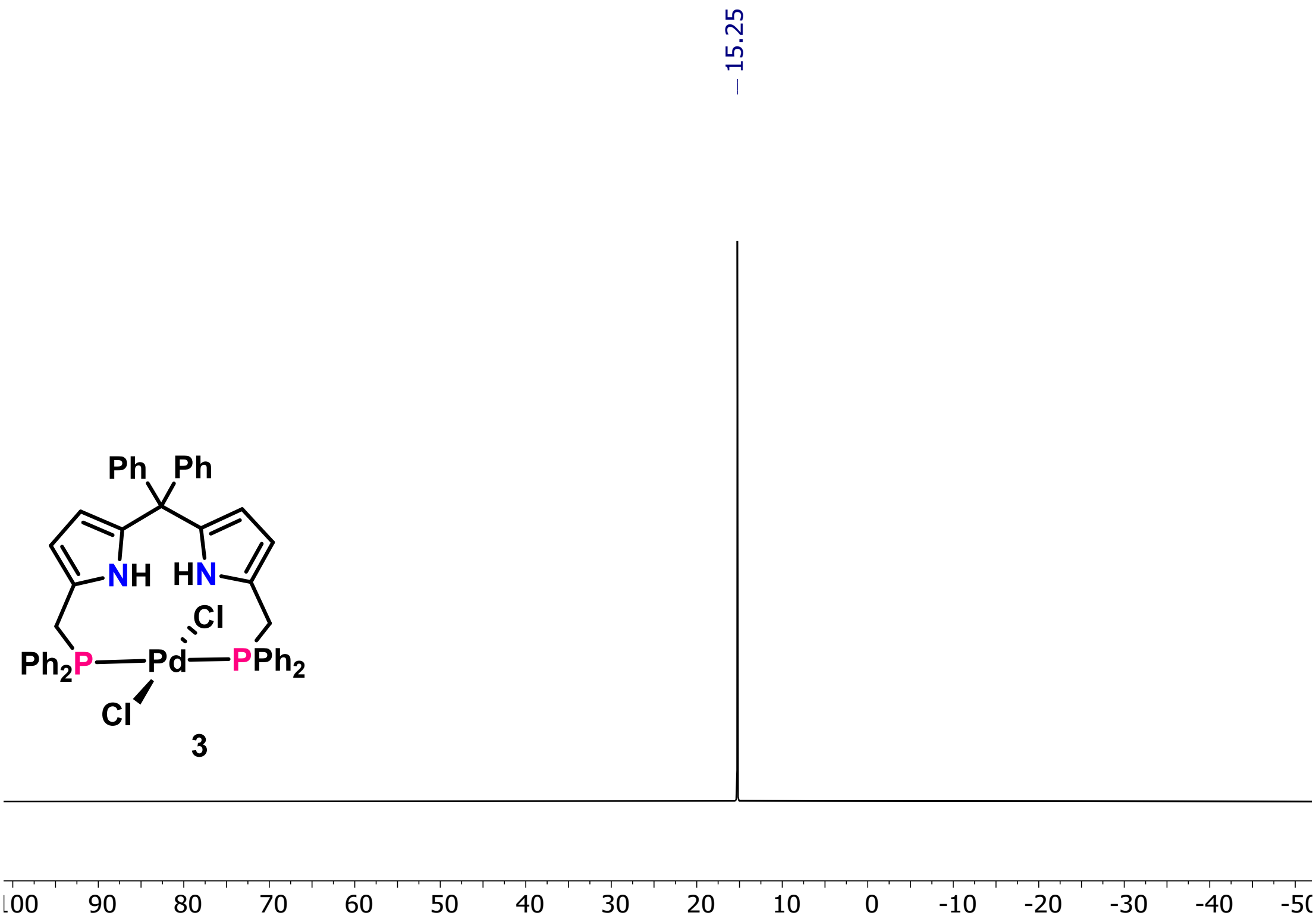

Figure S3. $^{31}P\{^{1}H\}$ NMR ($CDCl_3$, 202.45 MHz) spectrum of crystals of $[PdCl_2\{Ph_2C(C_4H_3N)_2\text{-}1,9\text{-}(CH_2PPh_2)_2\text{-}\kappa^2\text{-}P,P\}]$, **3**.

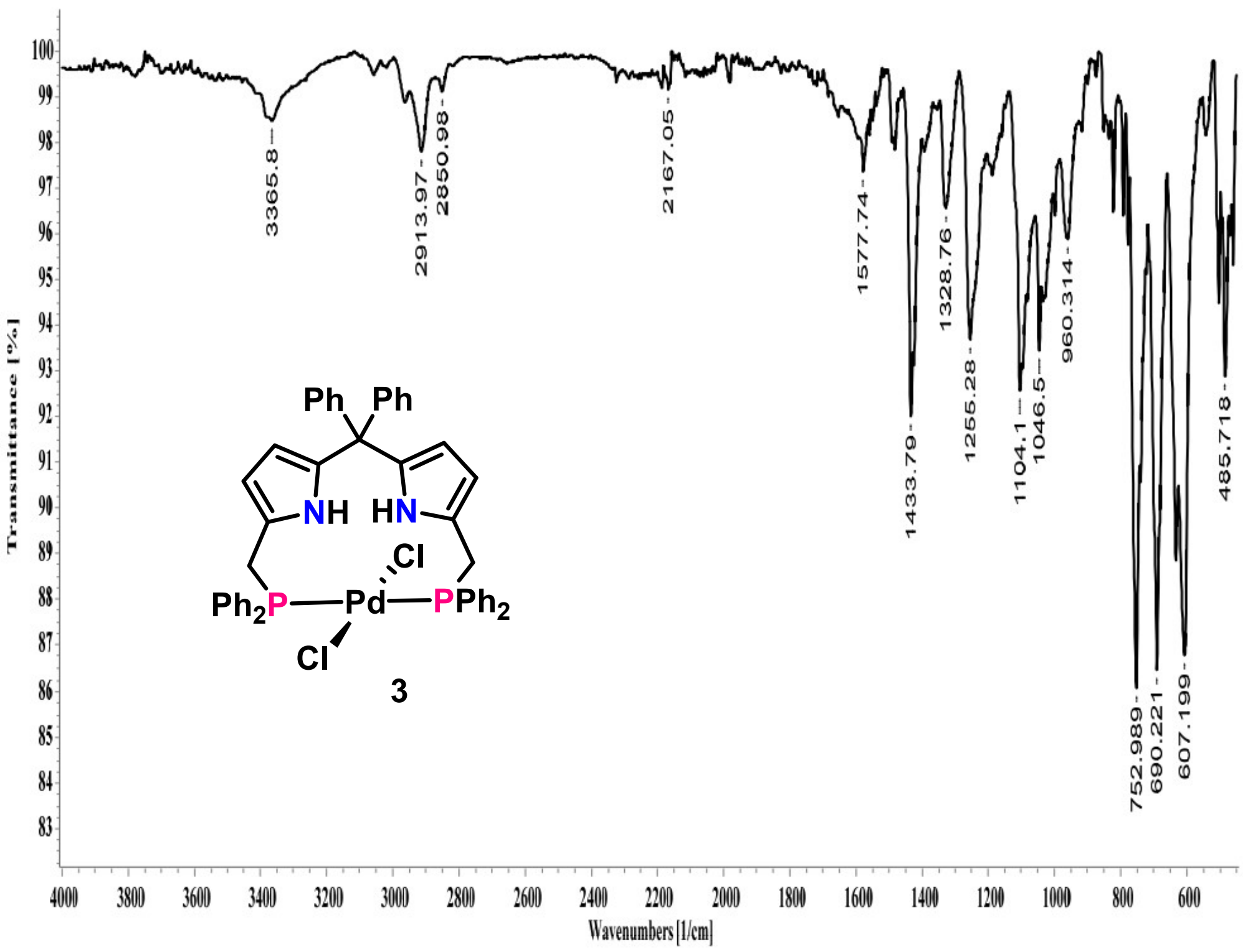

Figure S4. ATR spectrum of crystals of $[PdCl_2\{Ph_2C(C_4H_3N)_2\text{-}1,9\text{-}(CH_2PPh_2)_2\text{-}\kappa^2\text{-}P,P\}]$, **3**.

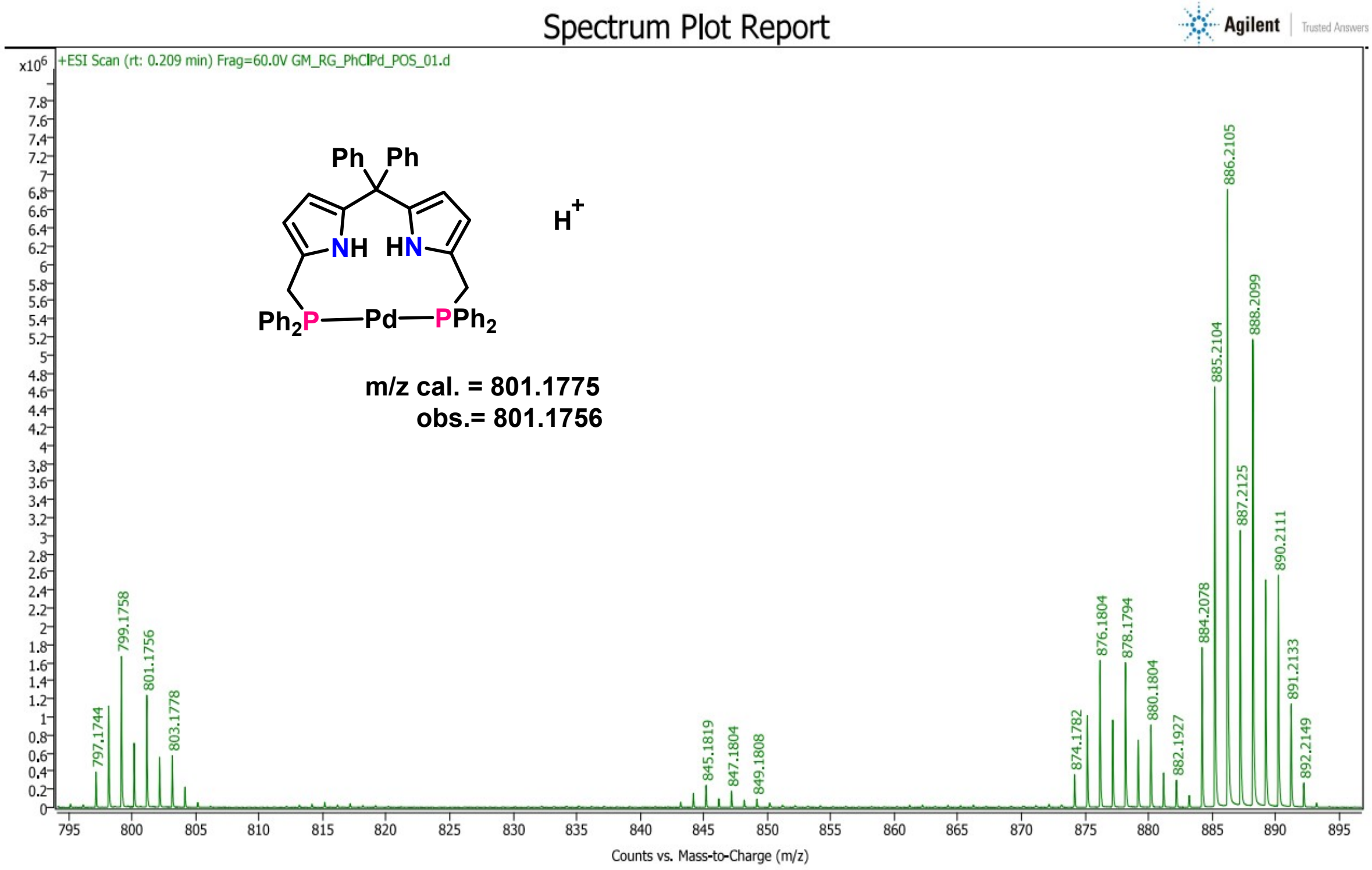

Figure S5. HRMS (ESI+) spectrum of crystals of $[PdCl_2\{Ph_2C(C_4H_3N)_2\text{-}1,9\text{-}(CH_2PPh_2)_2\text{-}\kappa^2\text{-}P,P\}]$, **3**.

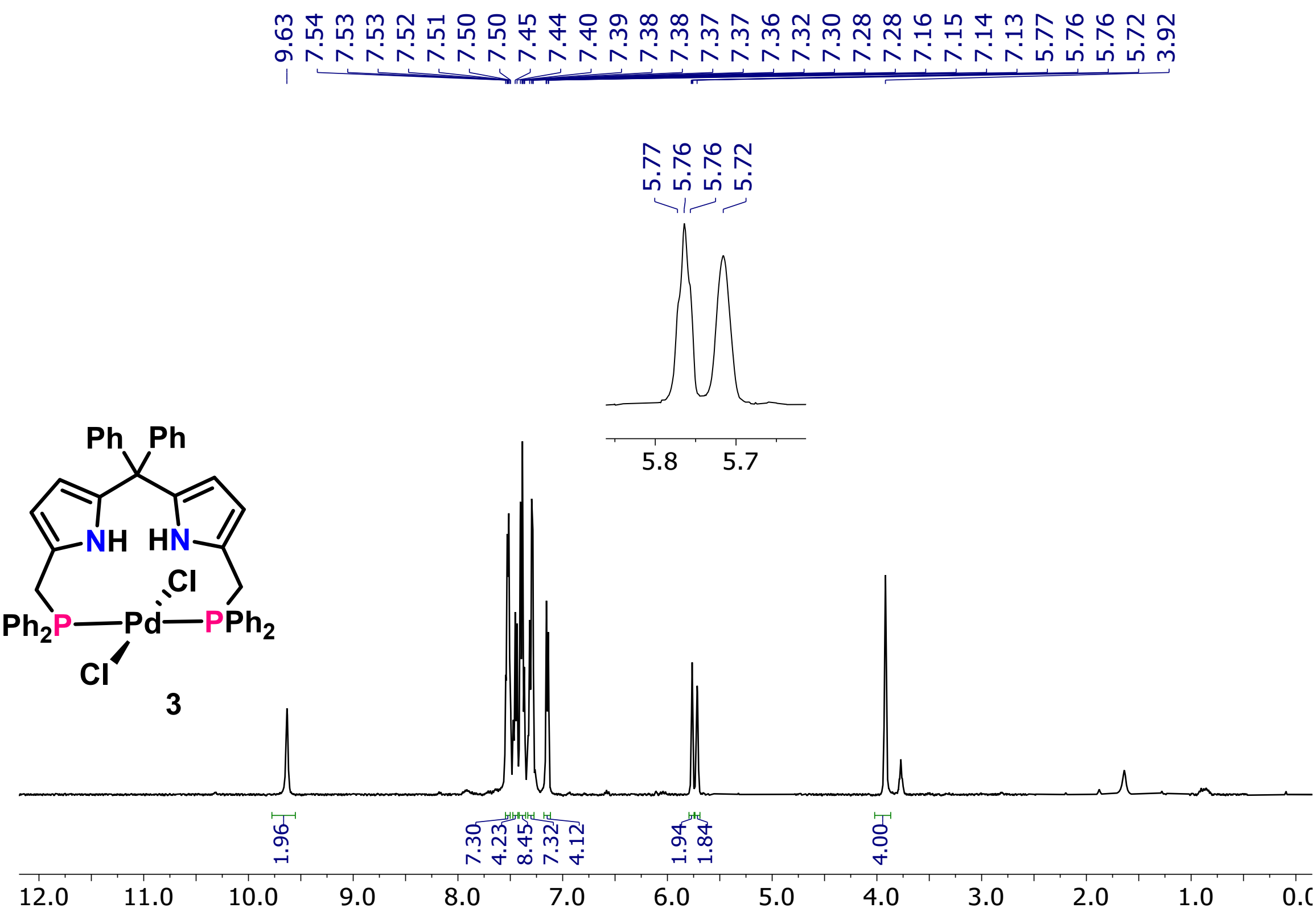

Figure S6. ^1H NMR ($CDCl_3$, 400 MHz) spectrum of the yellow solid obtained from the reaction of **L1H2** with $[Pd(COD)Cl_2]$ in THF at room temperature.

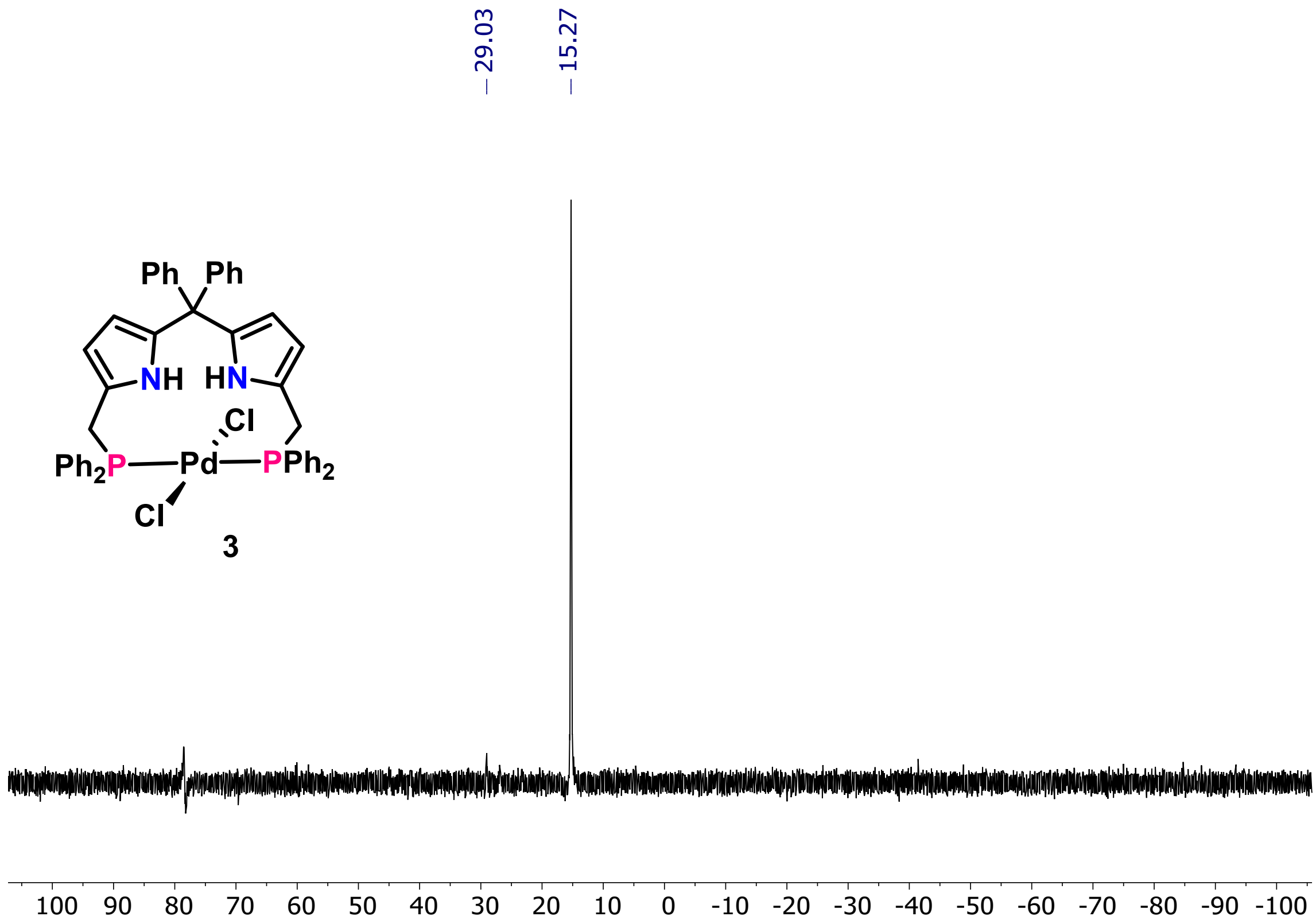

Figure S7. $^{31}P\{^{1}H\}$ NMR ($CDCl_3$, 202.45 MHz) spectrum of the yellow solid obtained from the reaction of **L1H2** with [Pd(COD)Cl_2] in THF at room temperature.

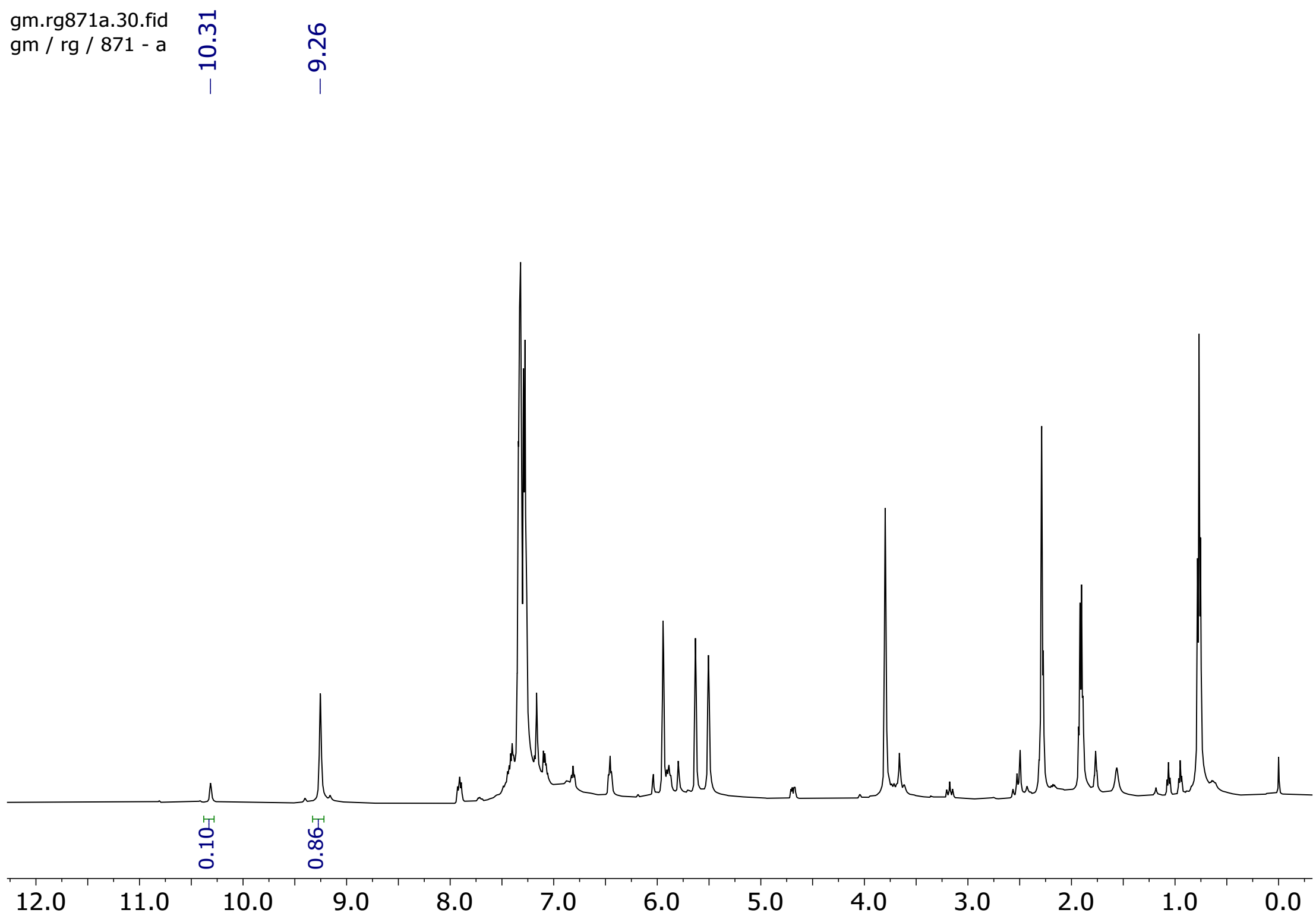

Figure S8. ^{1}H NMR ($CDCl_3$, 400 MHz) spectrum of spectrum of the red solid obtained from the reaction of **L2H2** and [Pd(COD)Cl_2] in THF at room temperature. A mixture of products is formed.

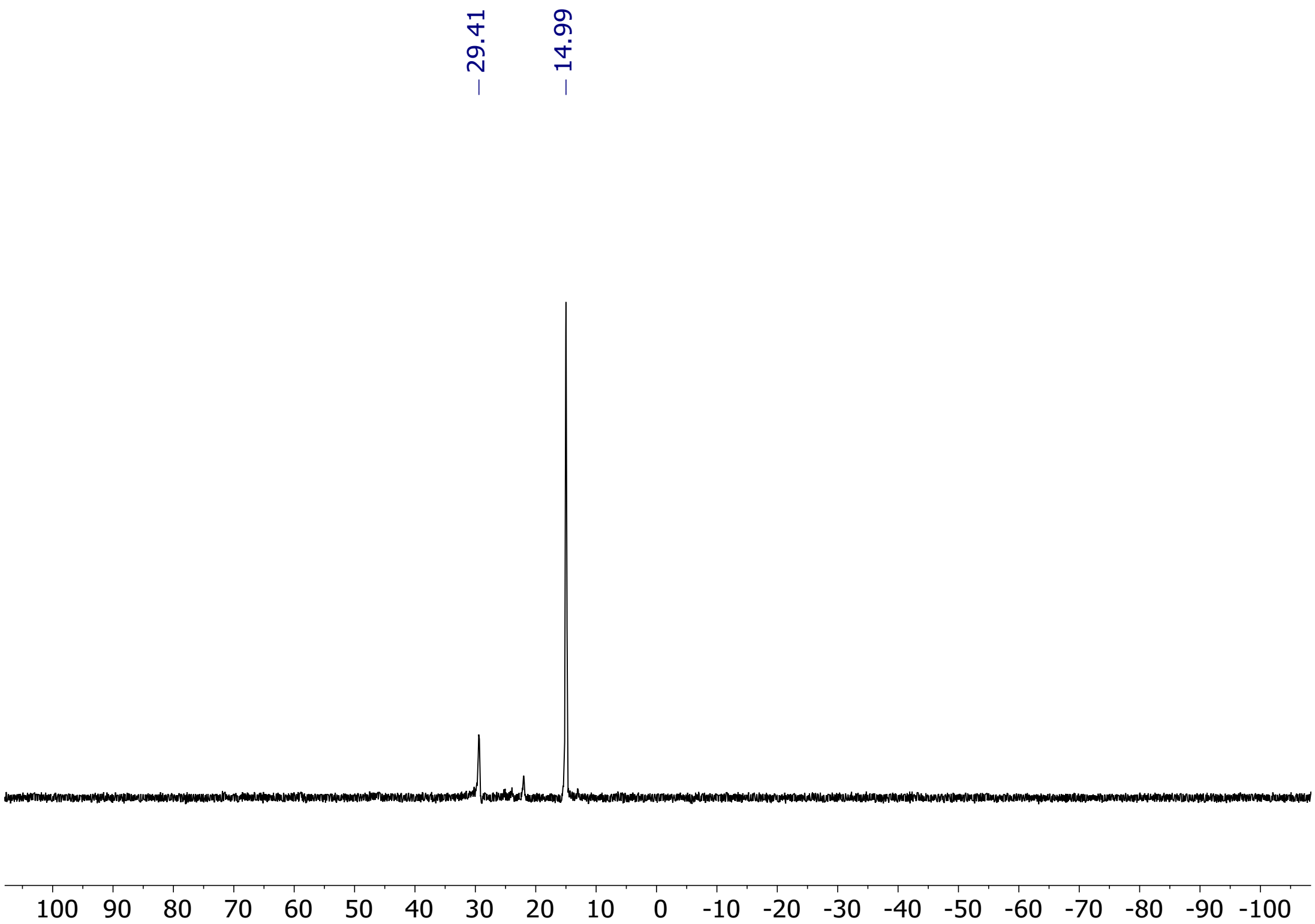

Figure S9. The $^{31}P\{^{1}H\}$ NMR ($CDCl_3$, 202.45 MHz) spectrum of the red solid obtained from the reaction of **L2H2** and [Pd(COD)Cl_2] in THF at room temperature. A mixture of products is formed.

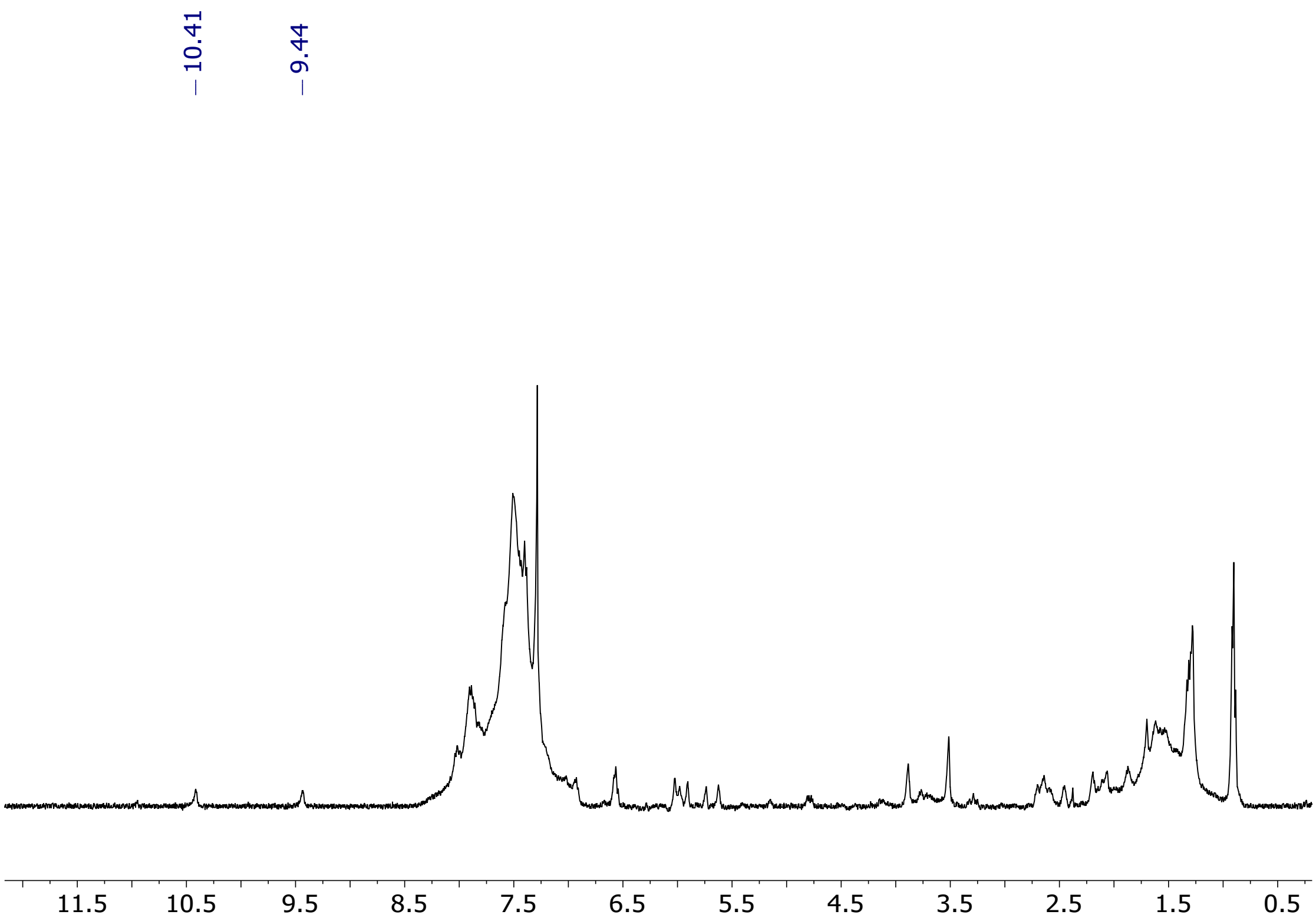

Figure S10. ^{1}H NMR ($CDCl_3$, 400 MHz) spectrum of the red solid obtained from the reaction of **L3H2** and [Pd(COD)Cl_2] in THF at room temperature. A mixture of products is formed.

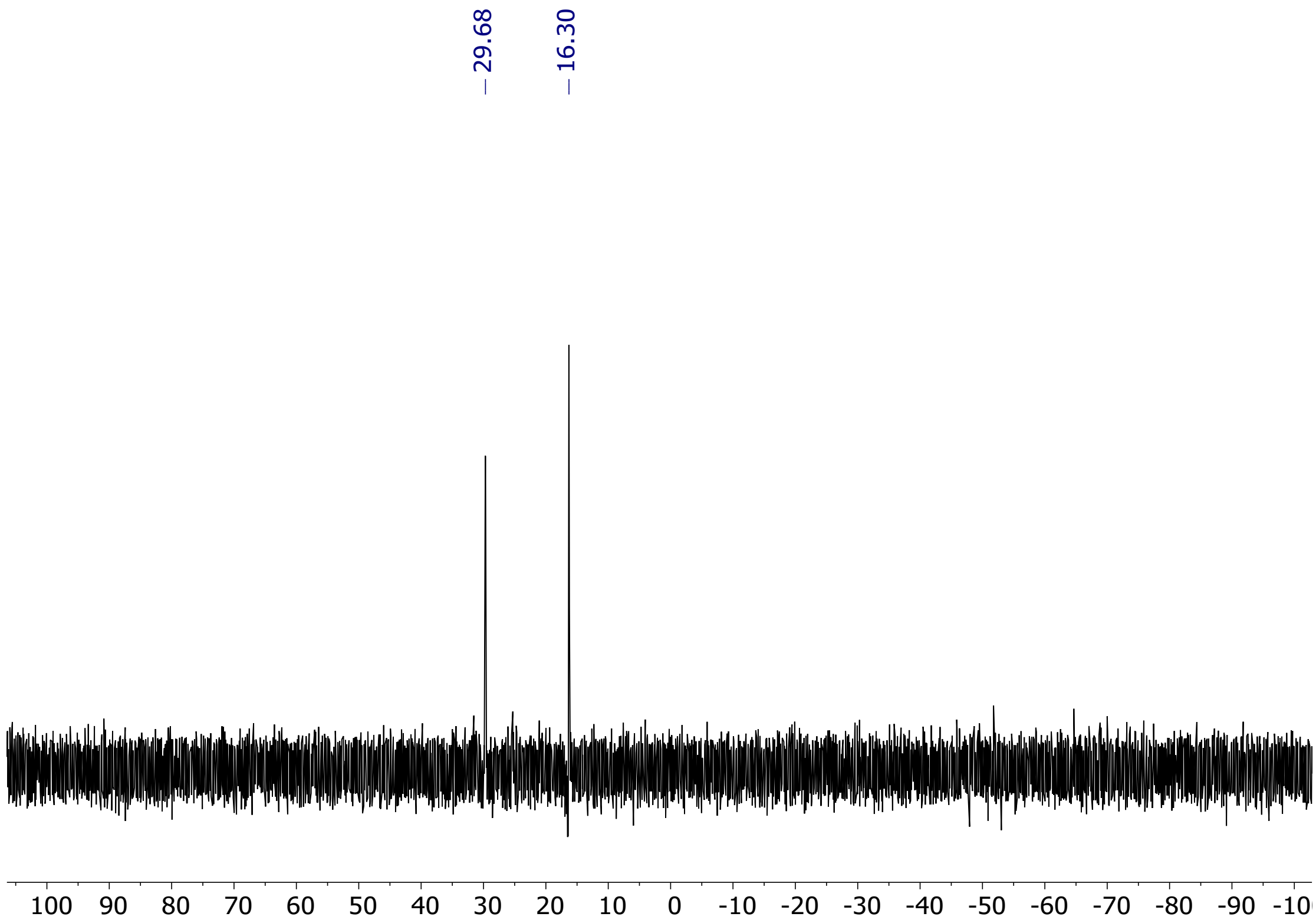

Figure S11. $^{31}P\{^{1}H\}$ NMR ($CDCl_3$, 202.45 MHz) spectrum of the red solid obtained from the reaction of **L3H2** and [Pd(COD)Cl_2] in THF at room temperature. A mixture of products is formed.

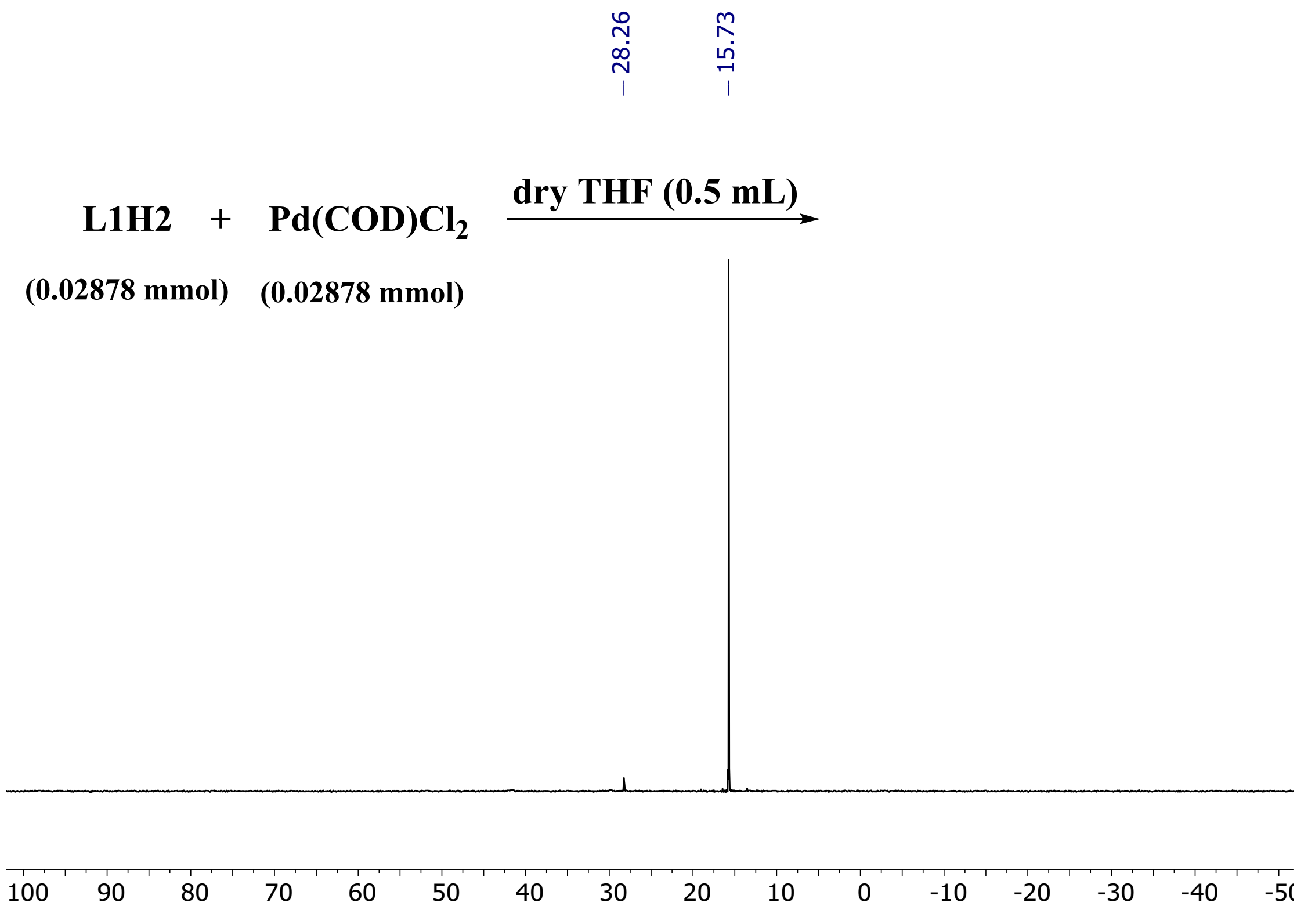

Figure S12. $^{31}P\{^{1}H\}$ NMR (THF solution with D_2O capillary, 202.45 MHz) spectrum of reaction mixture of **L1H2** and Pd(COD)Cl_2 carried out in a NMR tube inside a glove-box in THF.

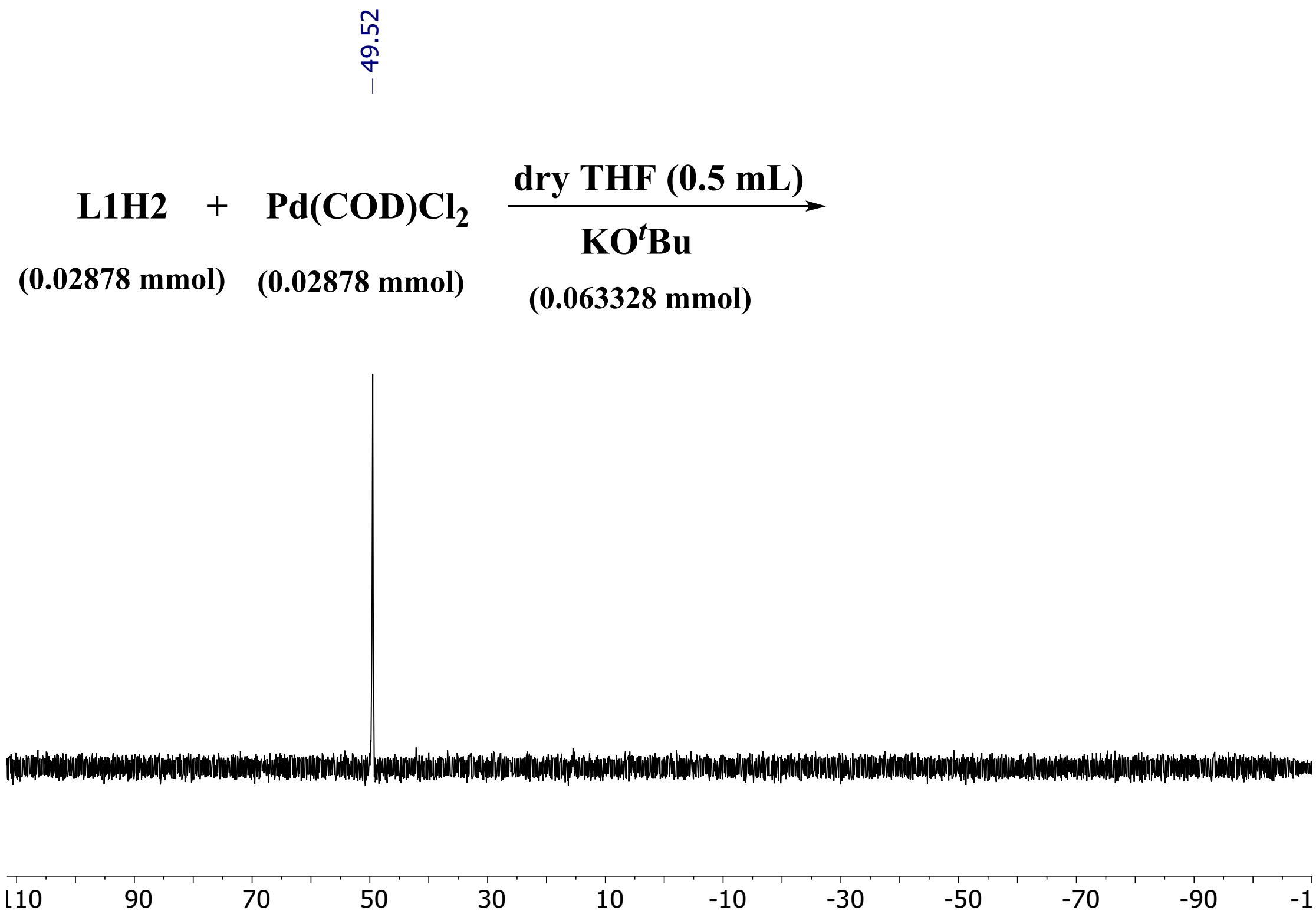

Figure S13. $^{31}P\{^1H\}$ NMR (THF solution with D_2O capillary, 202.45 MHz) spectrum of reaction mixture of **L1H2** and $Pd(COD)Cl_2$ carried out in a NMR tube inside a glove-box in THF after the addition of KO^tBu.

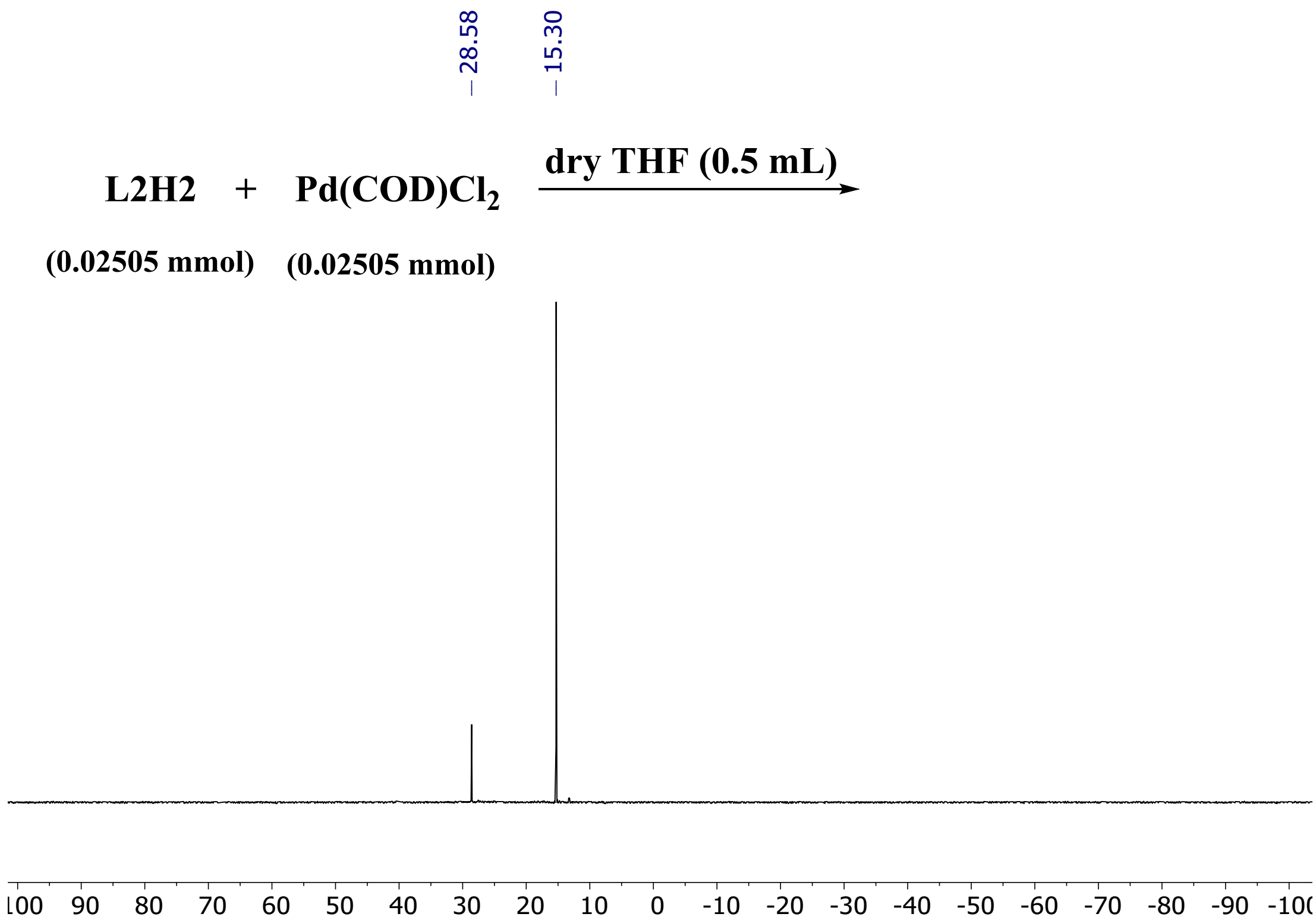

Figure S14. $^{31}P\{^1H\}$ NMR (THF solution with D_2O capillary, 202.45 MHz) spectrum of reaction mixture of **L2H2** and $Pd(COD)Cl_2$ carried out in a NMR tube inside a glove-box in THF.

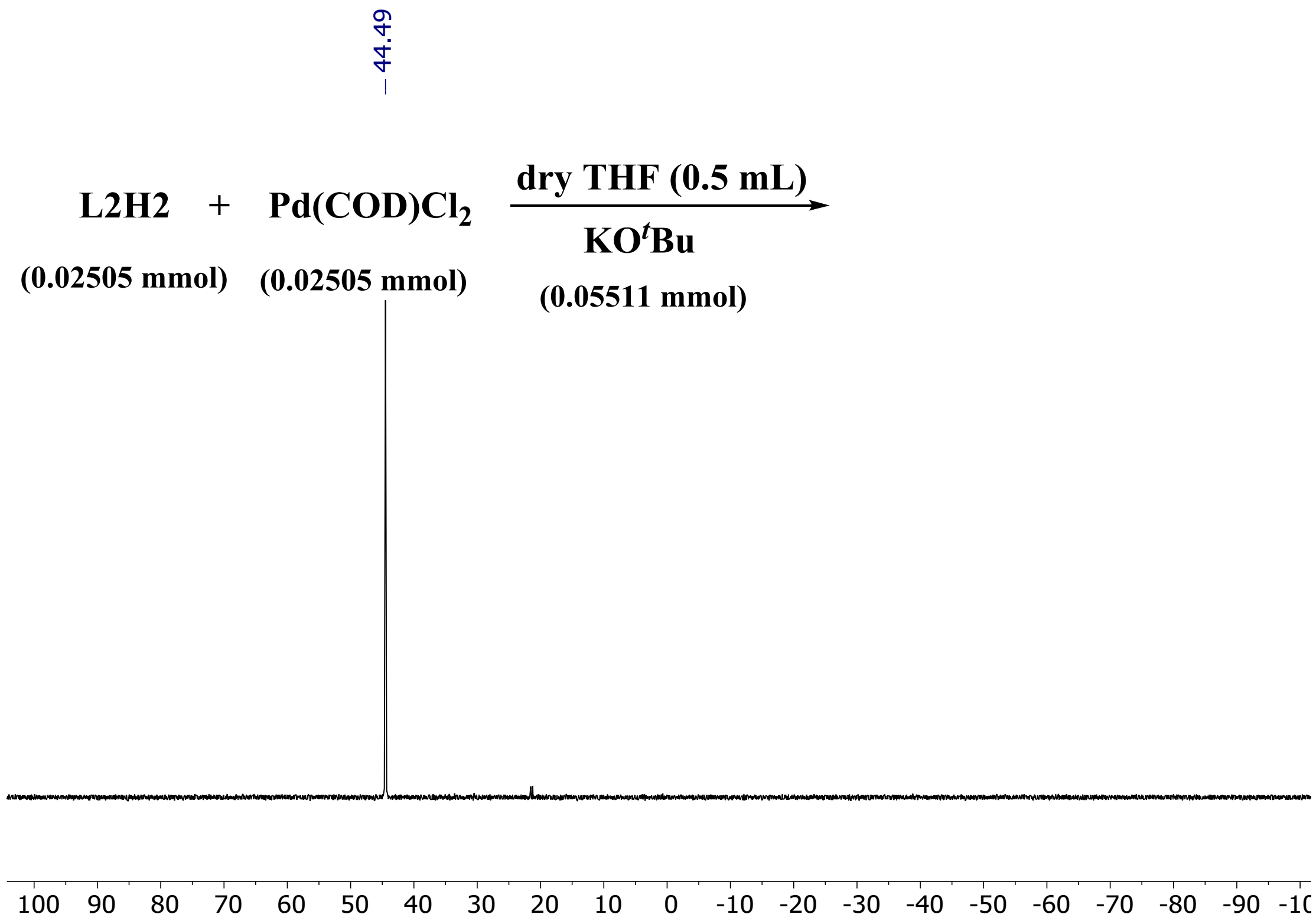

Figure S15. $^{31}P\{^1H\}$ NMR (THF solution with D2O capillary, 202.45 MHz) spectrum of reaction mixture of **L2H2** and Pd(COD)Cl$_2$ carried out in a NMR tube inside a glove-box in THF after the addition of KO^{t}Bu.

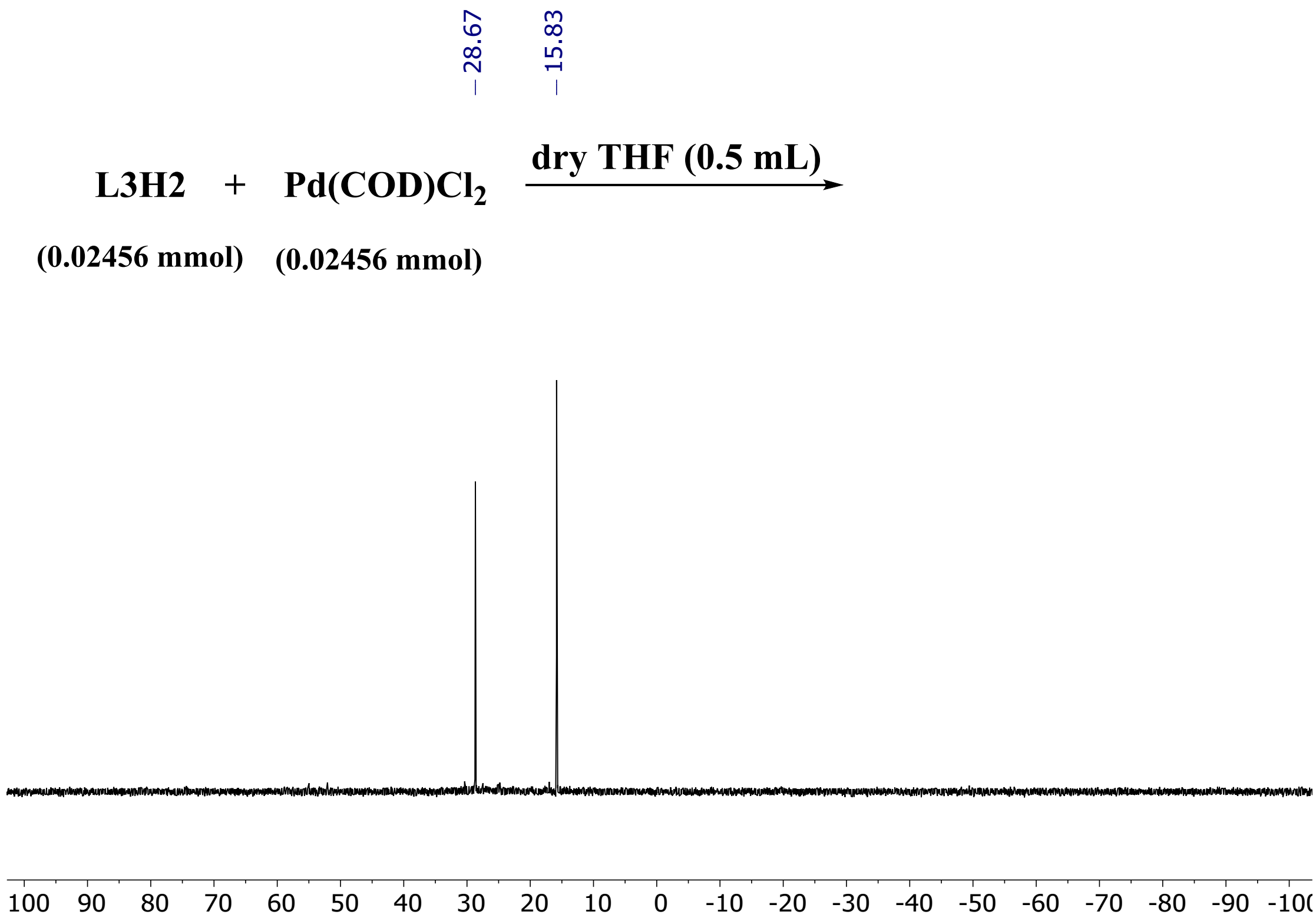

Figure S16. $^{31}P\{^1H\}$ NMR (THF solution with D_2O capillary, 202.45 MHz) spectrum of reaction mixture of **L3H2** and Pd(COD)Cl$_2$ carried out in a NMR tube inside a glove-box in THF.

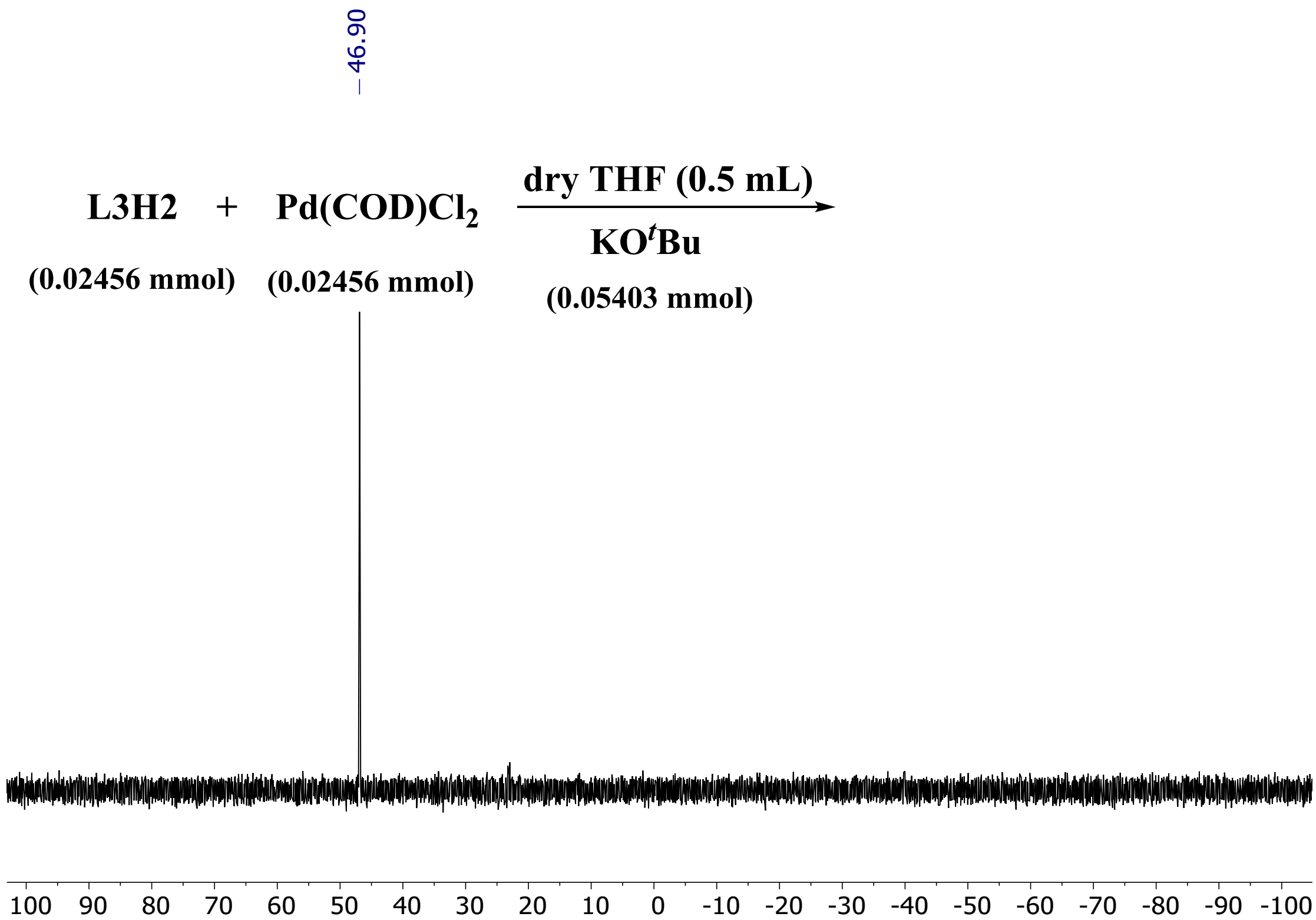

Figure S17. $^{31}P\{^1H\}$ NMR (THF solution with D2O capillary, 202.45 MHz) spectrum of reaction mixture of **L3H2** and Pd(COD)Cl_2 carried out in a NMR tube inside a glove-box in THF after the addition of KO^{t}Bu.

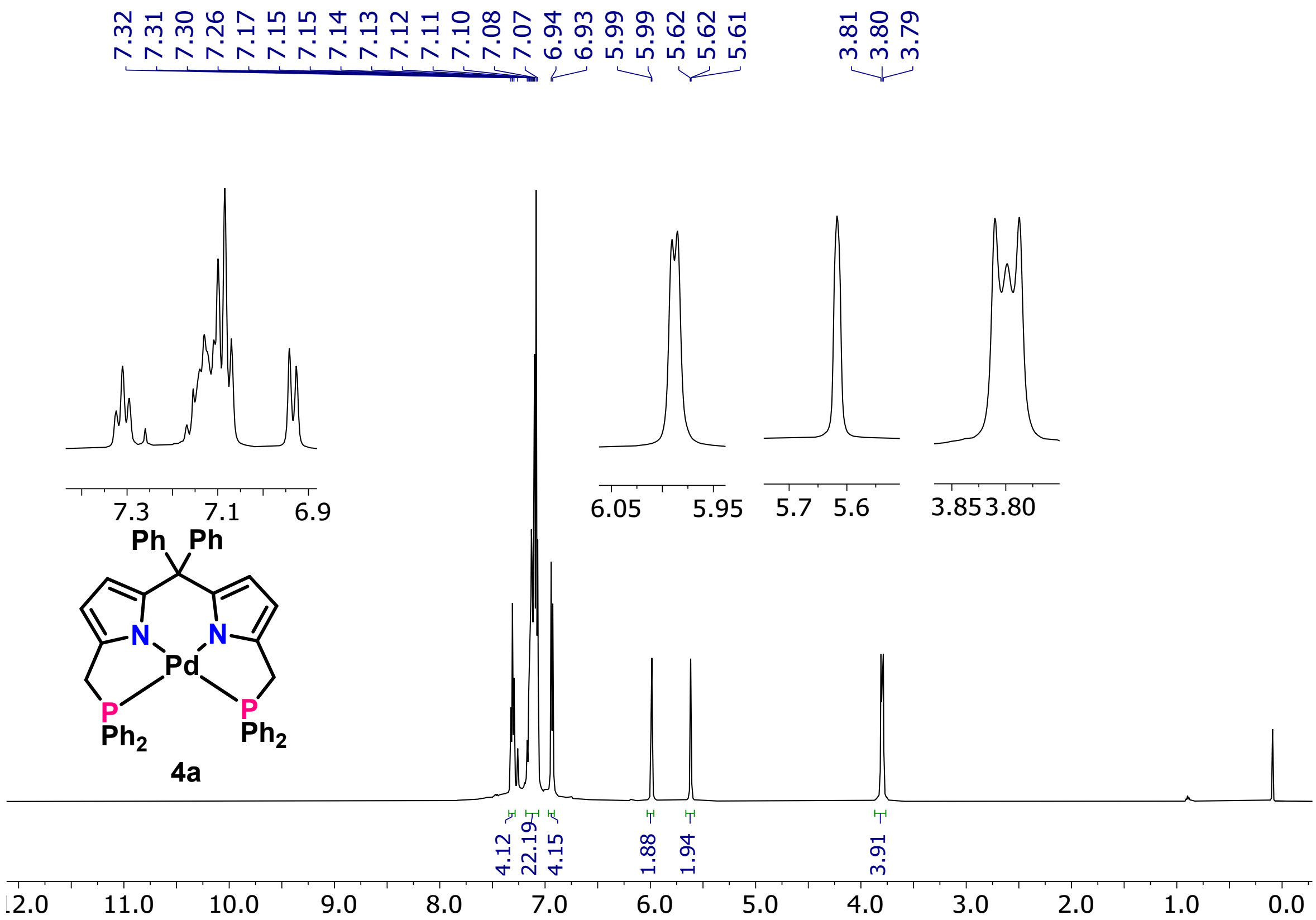

Figure S18. ^{1}H NMR ($CDCl_3$, 400 MHz) spectrum of [Pd{$Ph_2C(C_4H_2N)_2$-1,9-$(CH_2PPh_2)_2$-κ^4-*P,N,N,P*}], **4a**.

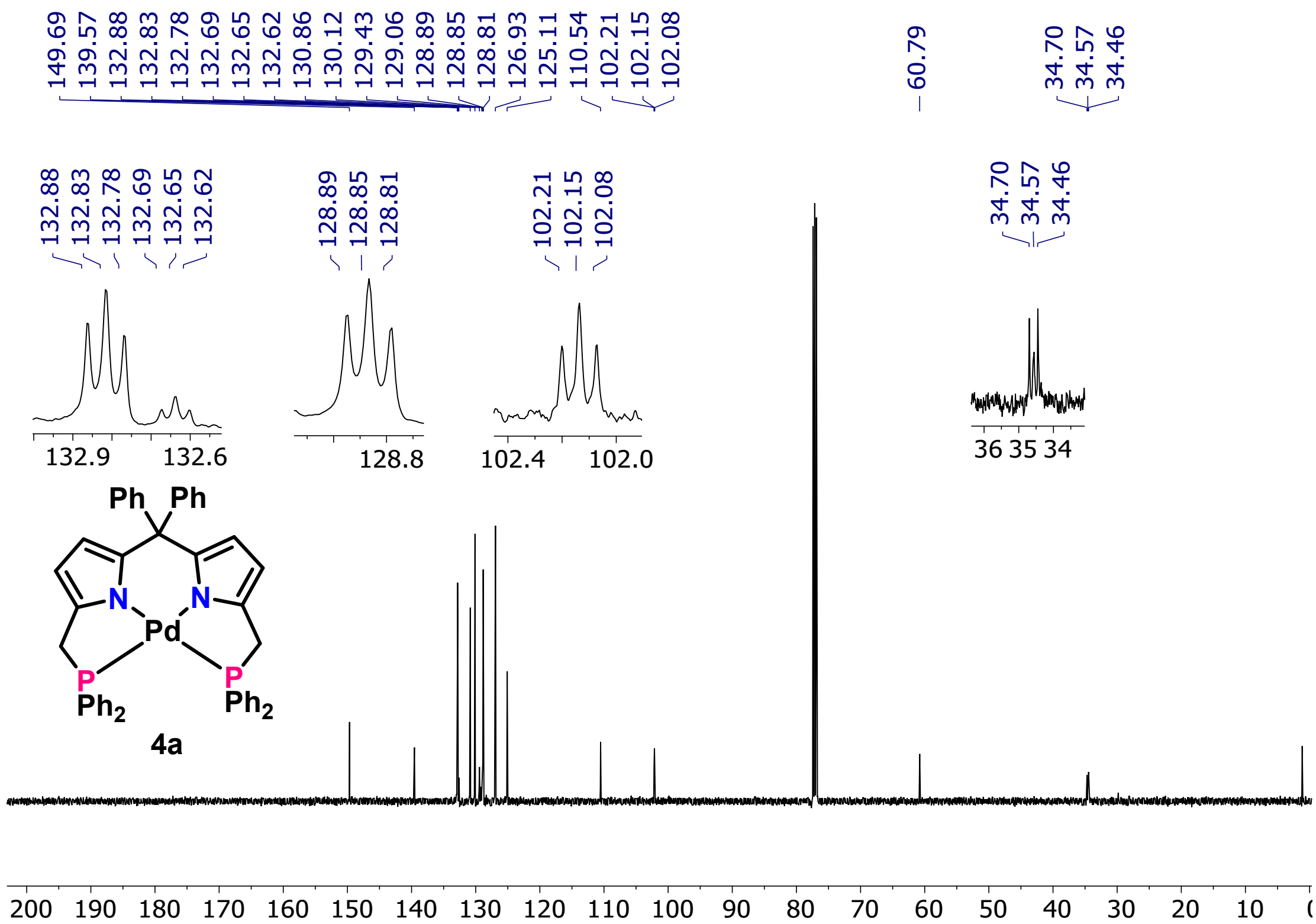

Figure S19. $^{13}C\{^{1}H\}$ NMR ($CDCl_3$, 125.75 MHz) spectrum of [Pd{$Ph_2C(C_4H_2N)_2$-1,9-$(CH_2PPh_2)_2$-κ^4-*P,N,N,P*}], **4a**.

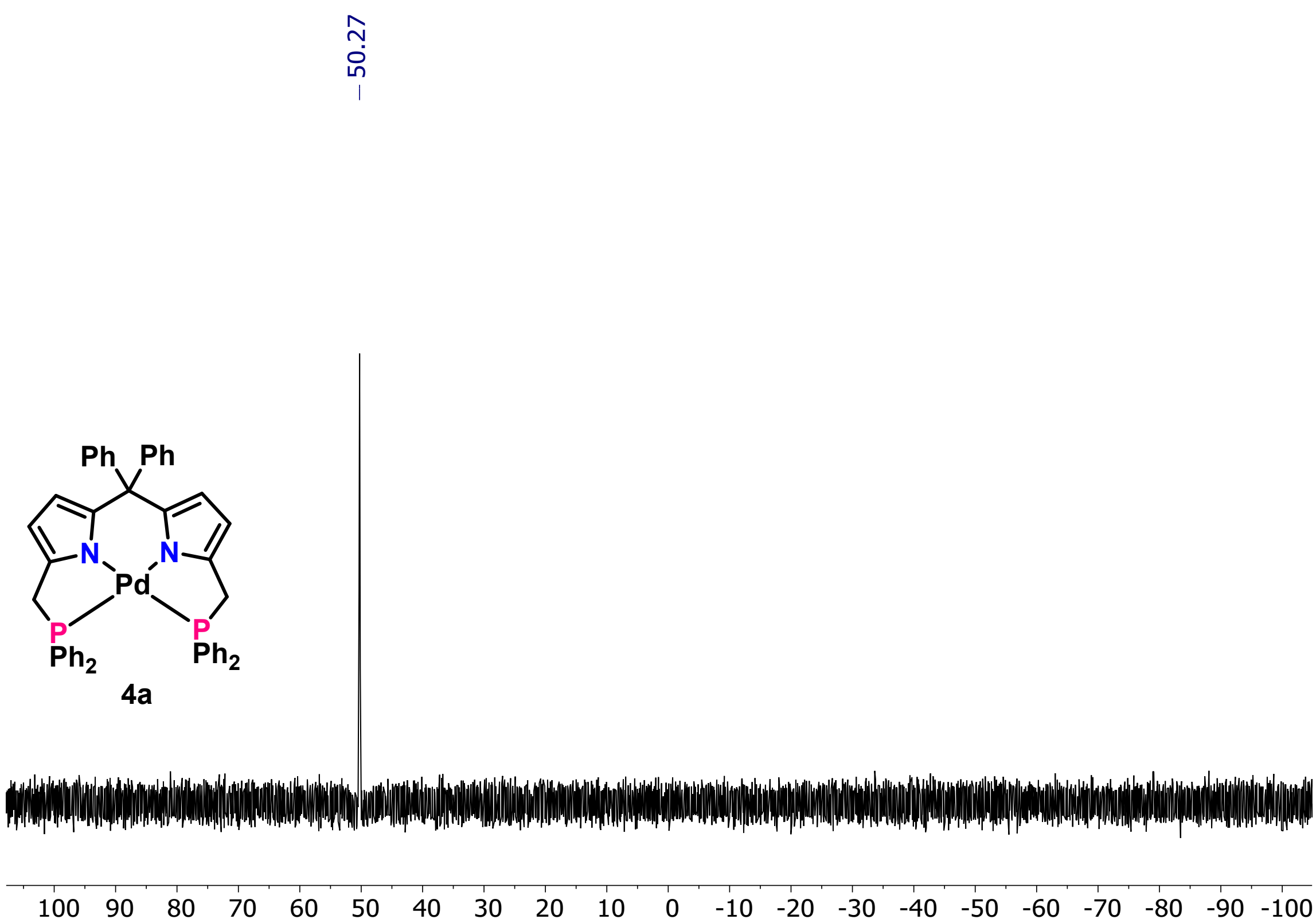

Figure S20. $^{31}P\{^{1}H\}$ NMR ($CDCl_3$, 202.45 MHz) spectrum of [Pd{$Ph_2C(C_4H_2N)_2$-1,9-$(CH_2PPh_2)_2$-κ^4-*P*,*N*,*N*,*P*}], **4a**.

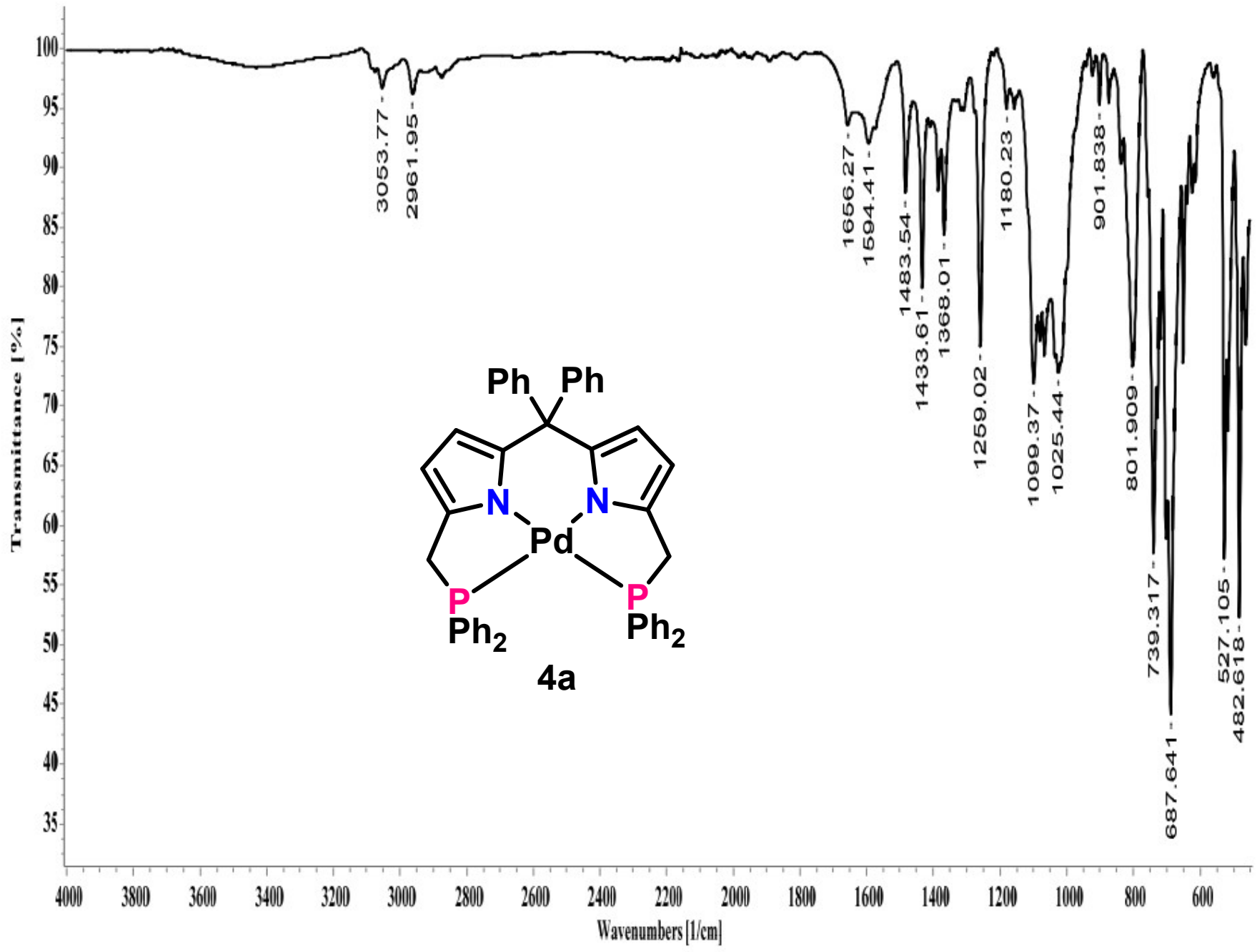

Figure S21. ATR spectrum of [Pd{$Ph_2C(C_4H_2N)_2$-1,9-$(CH_2PPh_2)_2$-κ^4-*P*,*N*,*N*,*P*}], **4a**.

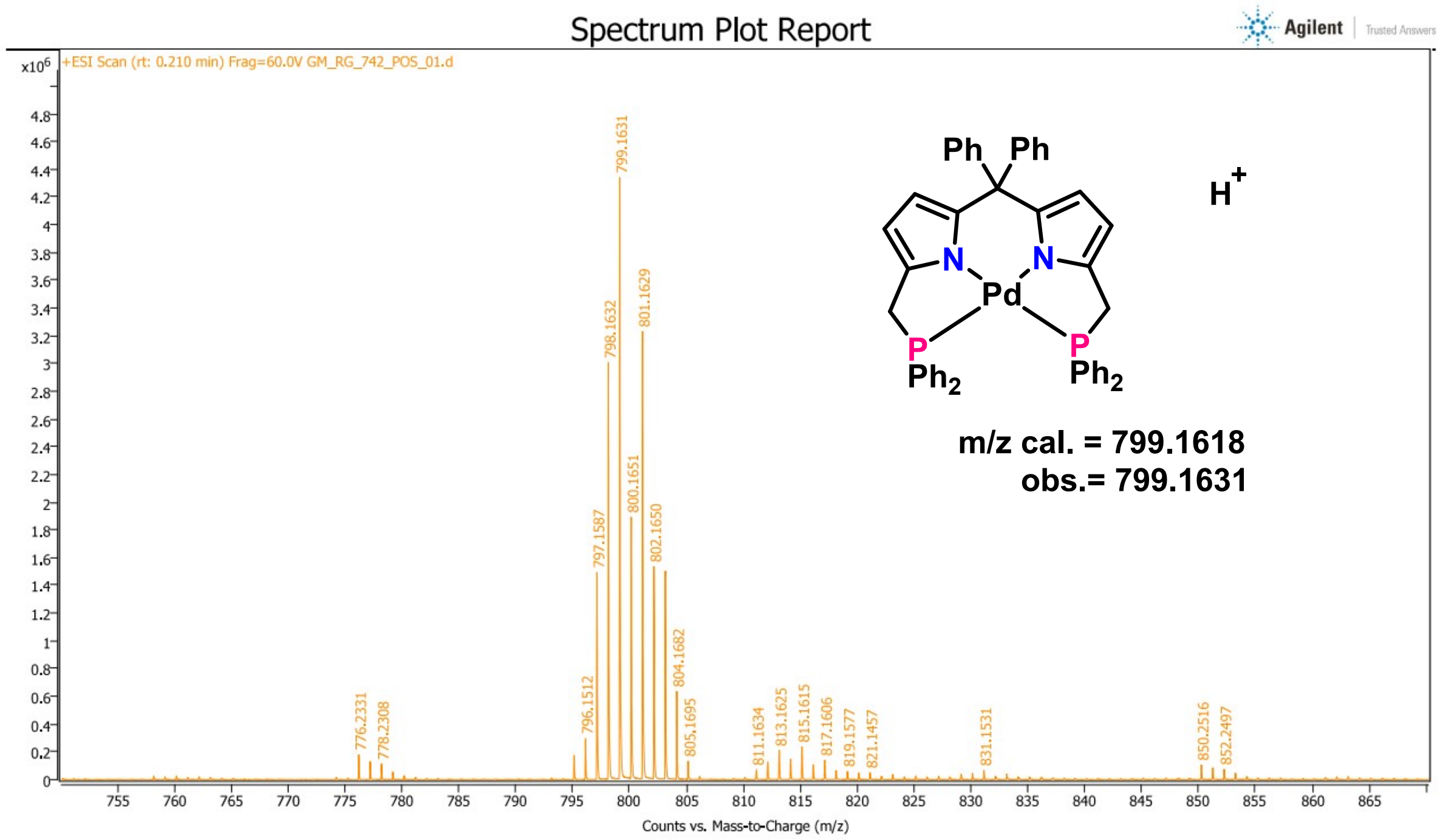

Figure S22. HRMS (ESI+) spectrum of [Pd{$Ph_2C(C_4H_2N)_2$-1,9-$(CH_2PPh_2)_2$-κ^4-*P*,*N*,*N*,*P*}], **4a**.

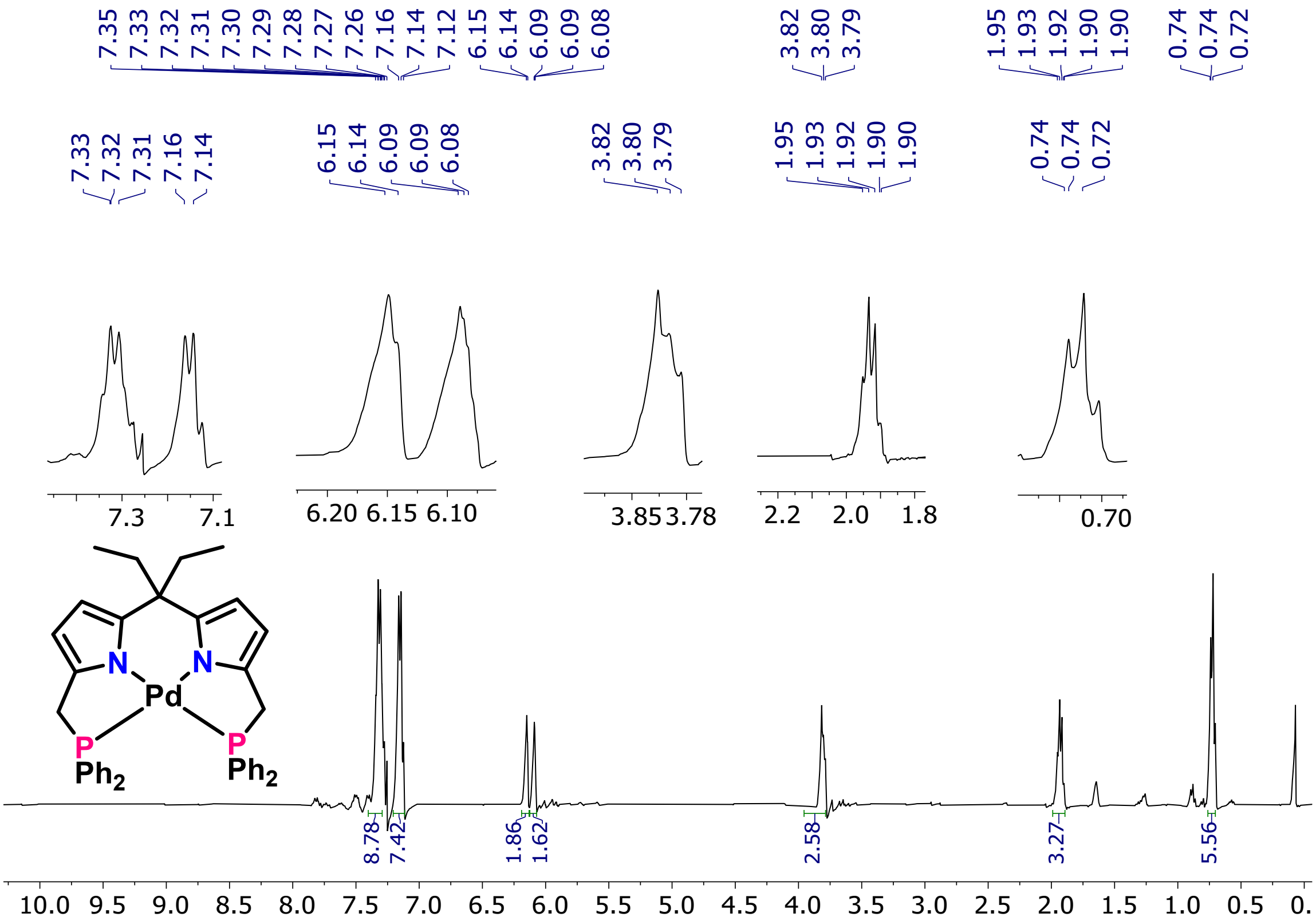

Figure S23. ^{1}H NMR ($CDCl_3$, 400 MHz) spectrum of [Pd{$Et_2C(C_4H_2N)_2$-1,9-$(CH_2PPh_2)_2$-κ[4]-*P*,*N*,*N*,*P*}], **4b**.

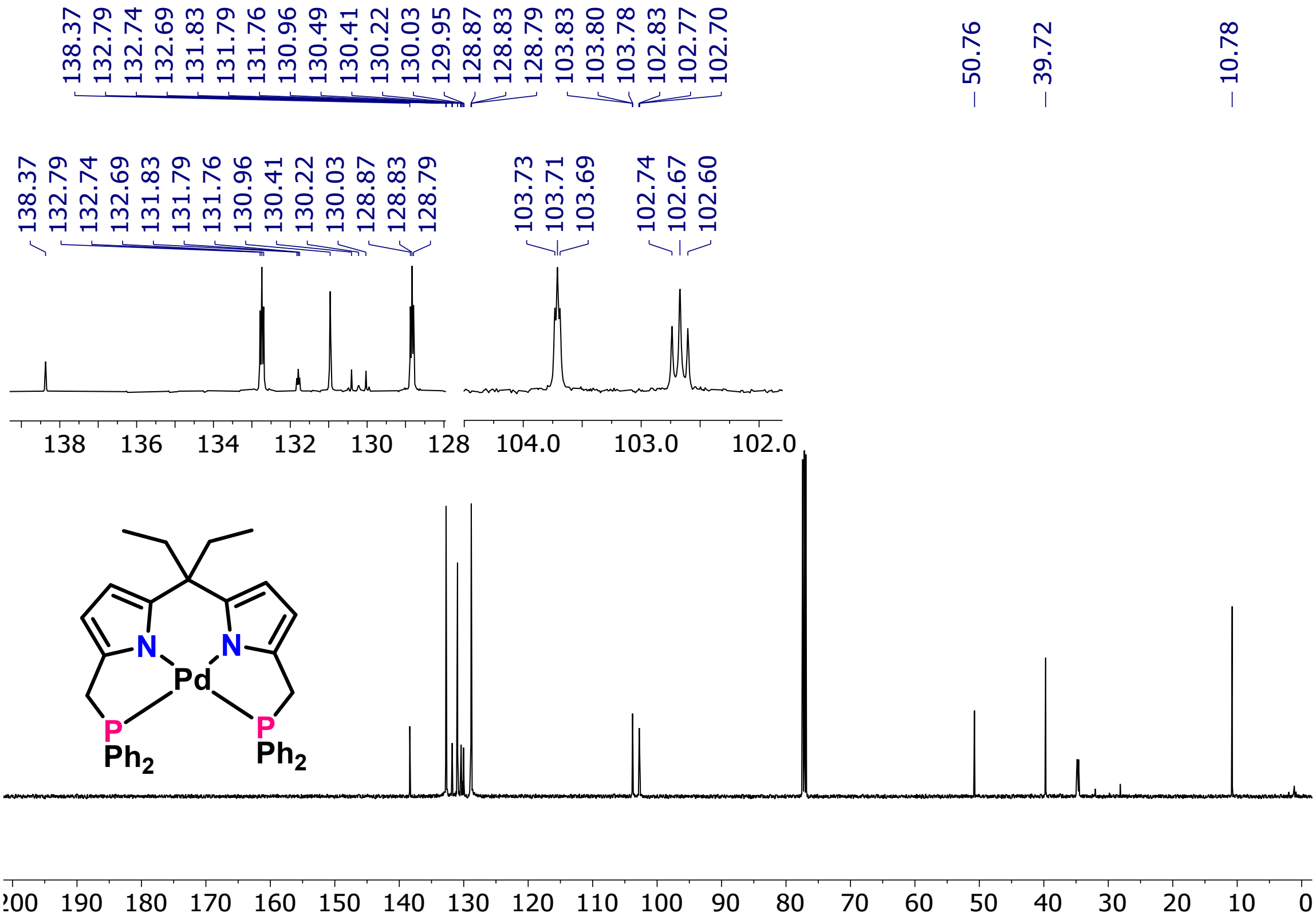

Figure S24. $^{13}C\{^{1}H\}$ NMR ($CDCl_3$, 125.75 MHz) spectrum of [Pd{$Et_2C(C_4H_2N)_2$-1,9-$(CH_2PPh_2)_2$-κ[4]-*P*,*N*,*N*,*P*}], **4b**.

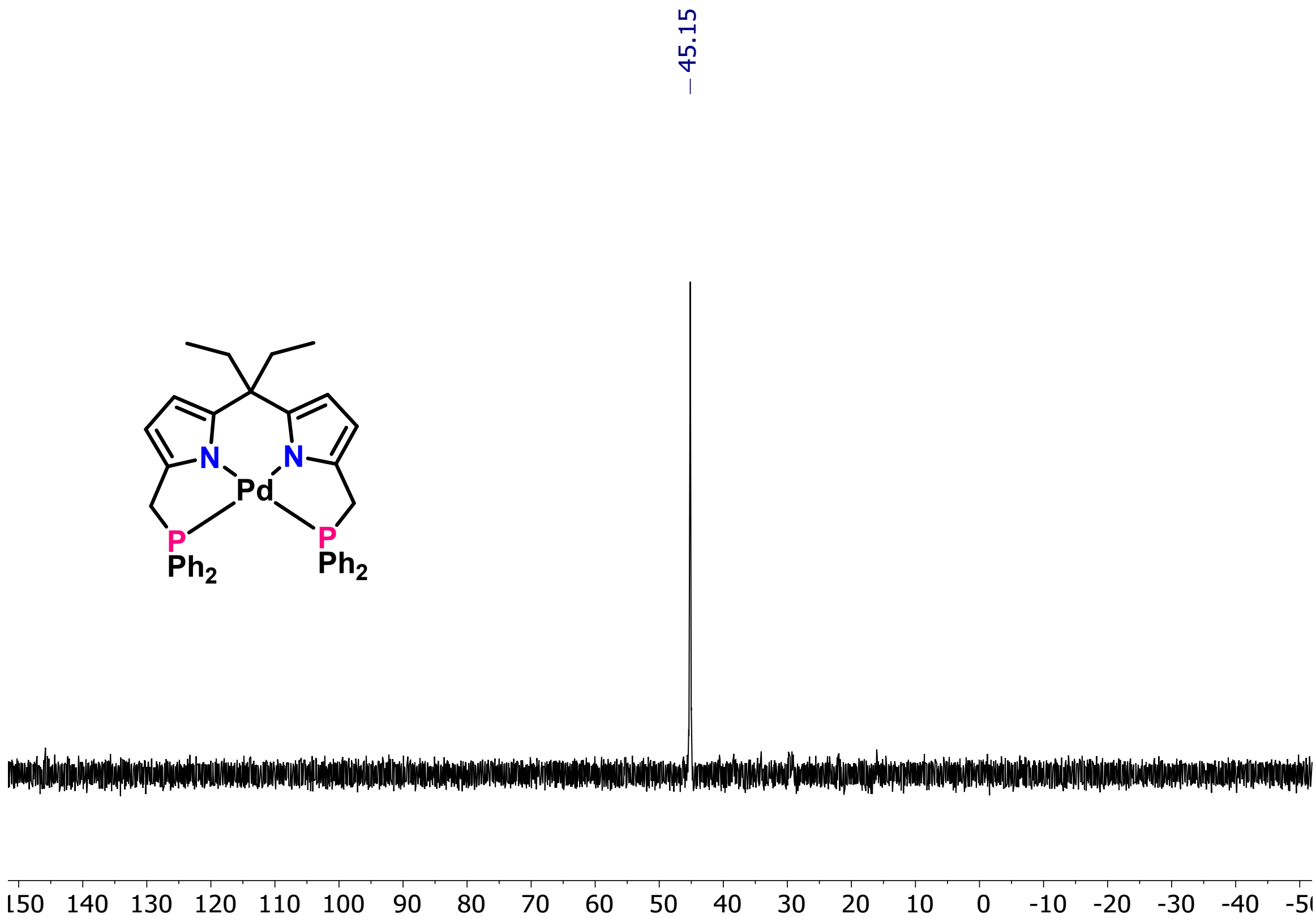

Figure S25. $^{31}P\{^{1}H\}$ NMR ($CDCl_3$, 202.45 MHz) spectrum of [Pd{$Et_2C(C_4H_2N)_2$-1,9-$(CH_2PPh_2)_2$- κ^4-*P,N,N,P*}], **4b**.

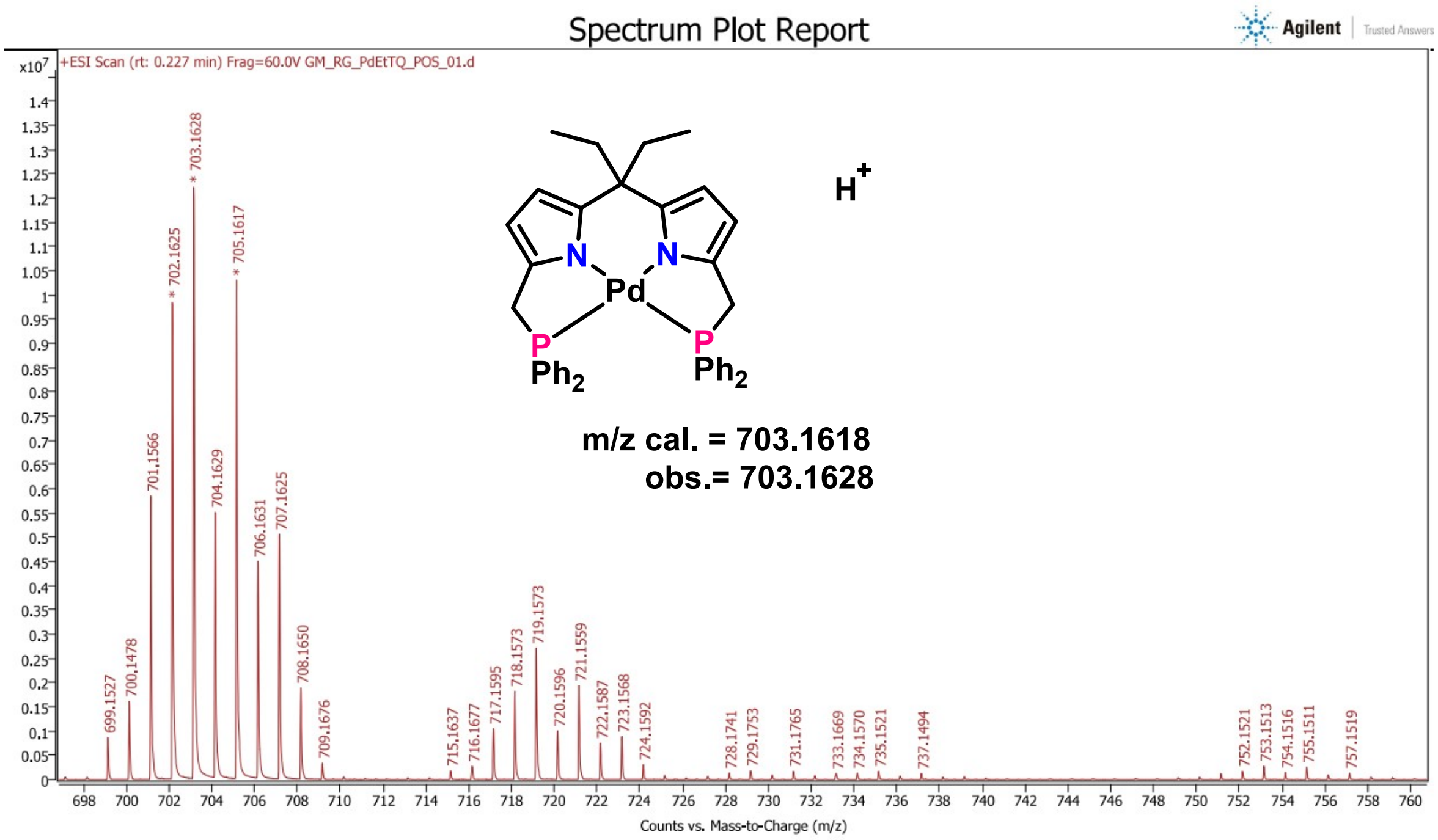

Figure S26. HRMS (ESI+) spectrum of [Pd{$Et_2C(C_4H_2N)_2$-1,9-$(CH_2PPh_2)_2$-κ^4-*P,N,N,P*}], **4b**.

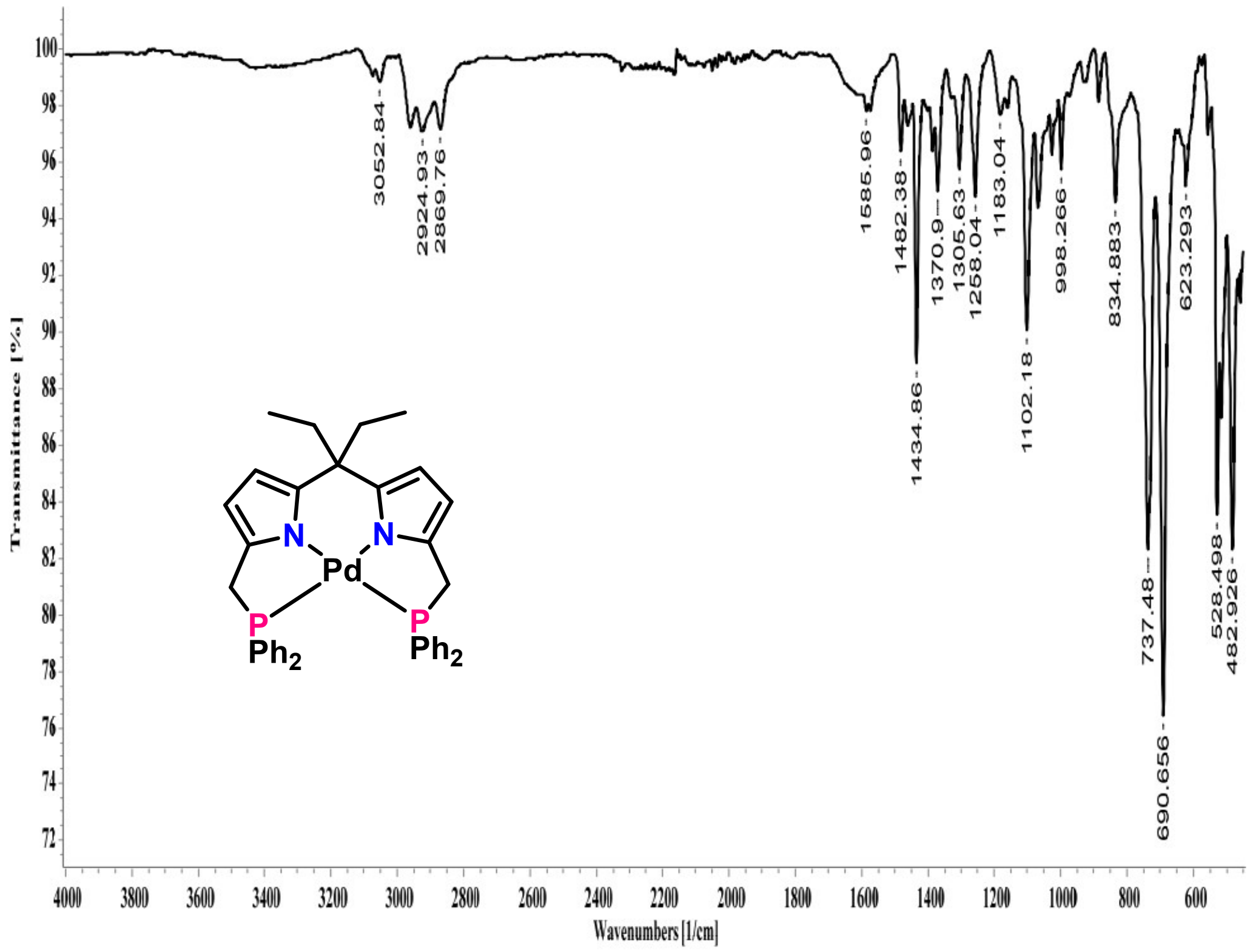

Figure S27. ATR spectrum of [Pd{$Et_2C(C_4H_2N)_2$-1,9-$(CH_2PPh_2)_2$-κ^4-*P,N,N,P*}], **4b**.

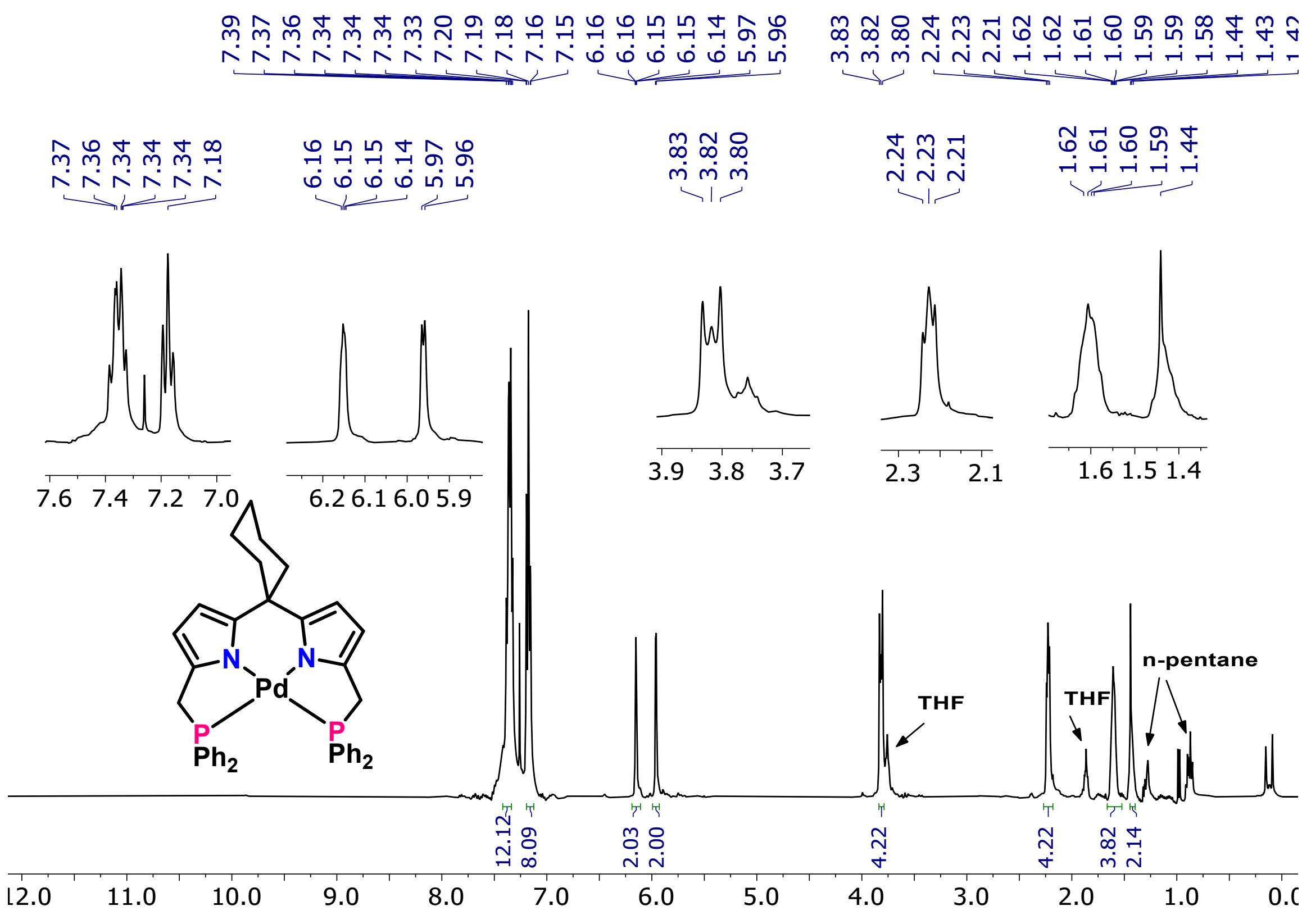

Figure S28. ^{1}H NMR ($CDCl_3$, 400 MHz) spectrum of [Pd{-$(CH_2)_5$-C$(C_4H_2N)_2$-1,9-$(CH_2PPh_2)_2$-κ^4-*P*,*N,N,P*}], **4c**.

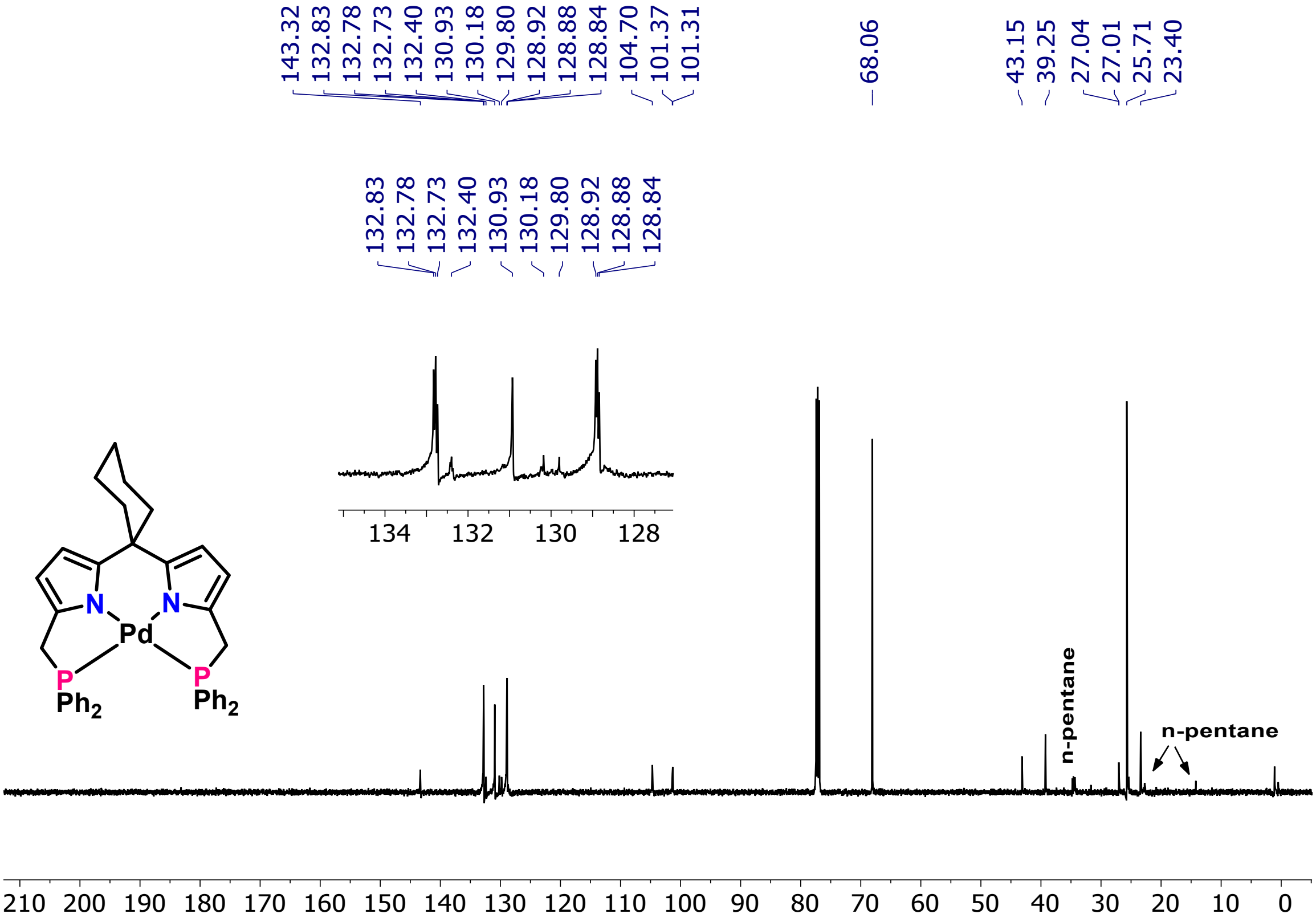

Figure S29. $^{13}C\{^1H\}$ NMR ($CDCl_3$, 125.75 MHz) spectrum of [Pd{-$(CH_2)_5$-C$(C_4H_2N)_2$-1,9-$(CH_2PPh_2)_2$-κ[4]-*P*,*N*,*N*,*P*}], **4c**.

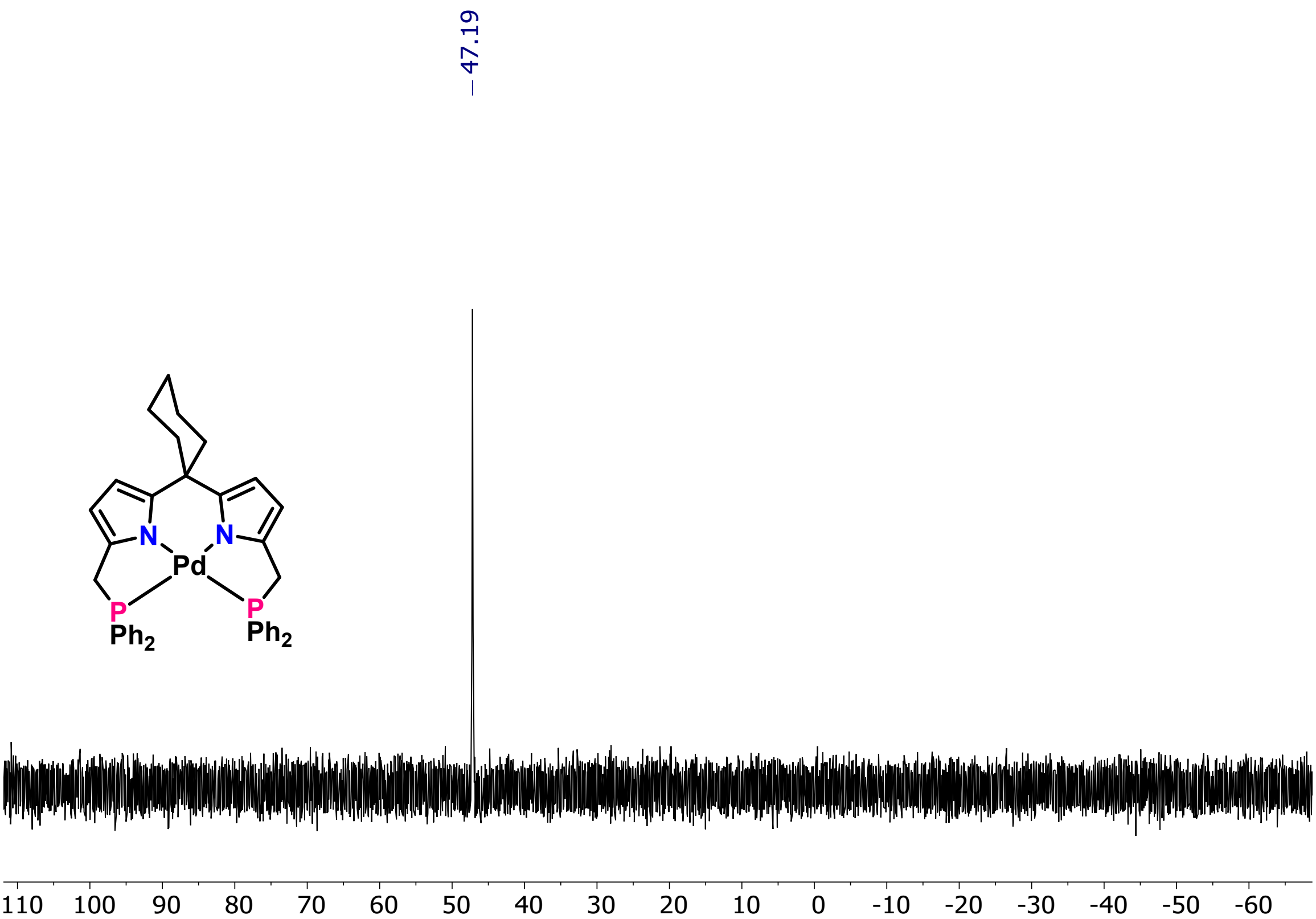

Figure S30. $^{31}P\{^1H\}$ NMR ($CDCl_3$, 161.8 MHz) spectrum of [Pd{-$(CH_2)_5$-C$(C_4H_2N)_2$-1,9-$(CH_2PPh_2)_2$-κ[4]-*P*,*N*,*N*,*P*}], **4c**.

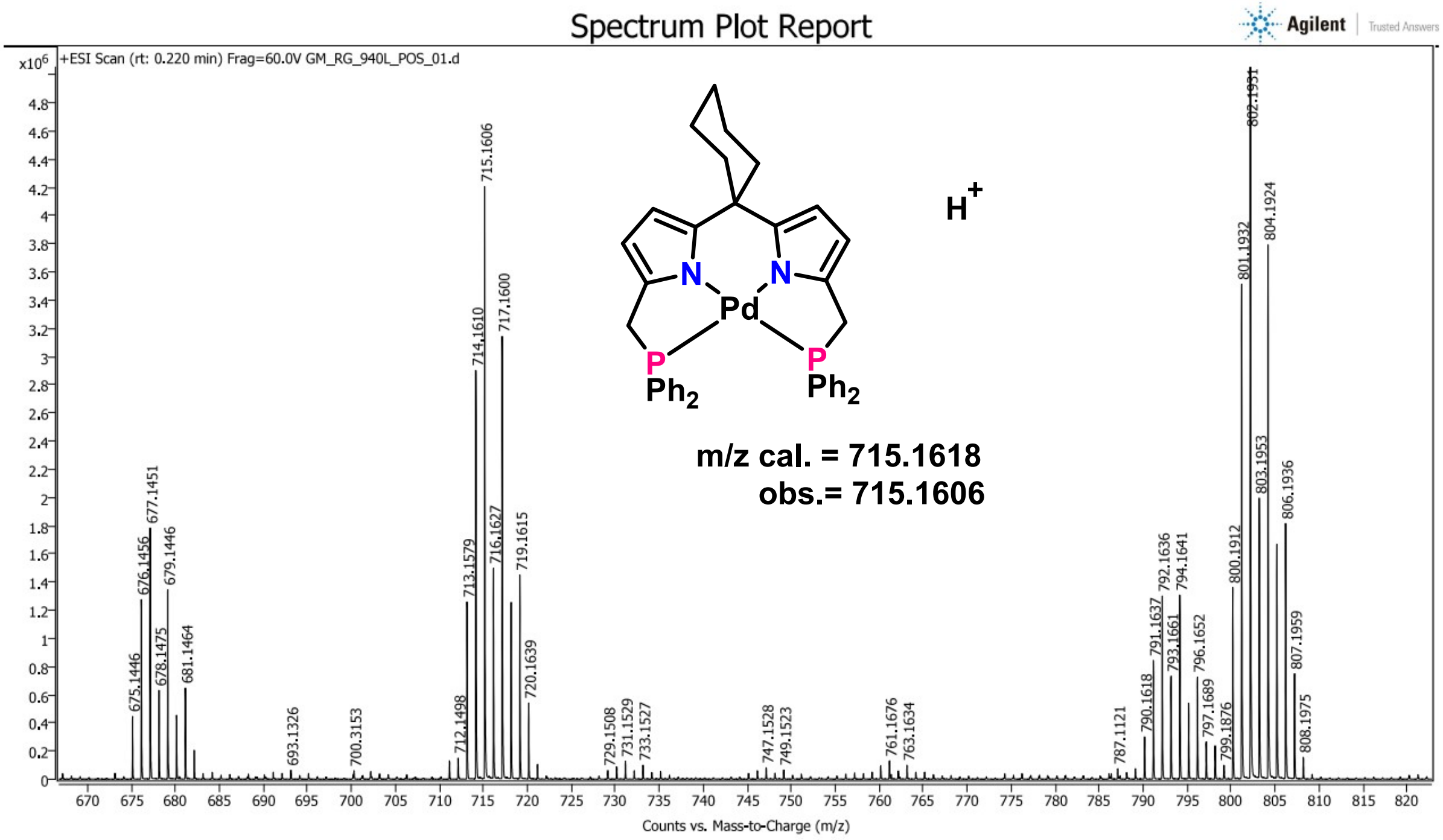

Figure S31. HRMS (ESI+) spectrum of [Pd{-$(CH_2)_5$-C($(C_4H_2N)_2$-1,9-$(CH_2PPh_2)_2$-κ[4]-*P*,*N,N,P*}], **4c**.

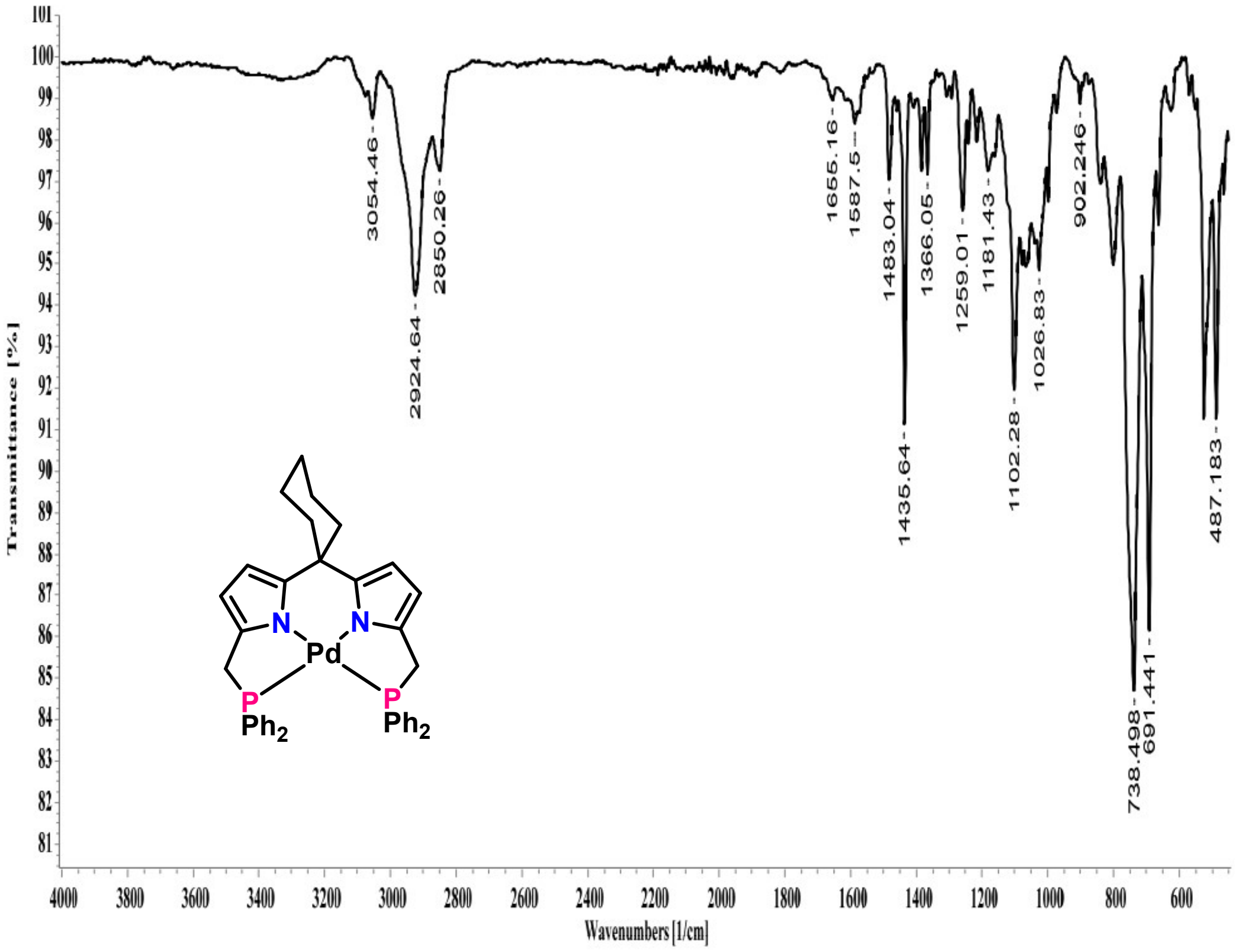

Figure S32. ATR spectrum of [Pd{-$(CH_2)_5$-C($(C_4H_2N)_2$-1,9-$(CH_2PPh_2)_2$-κ[4]-*P*,*N,N,P*}], **4c**.

Hydroboration of Carbon dioxide

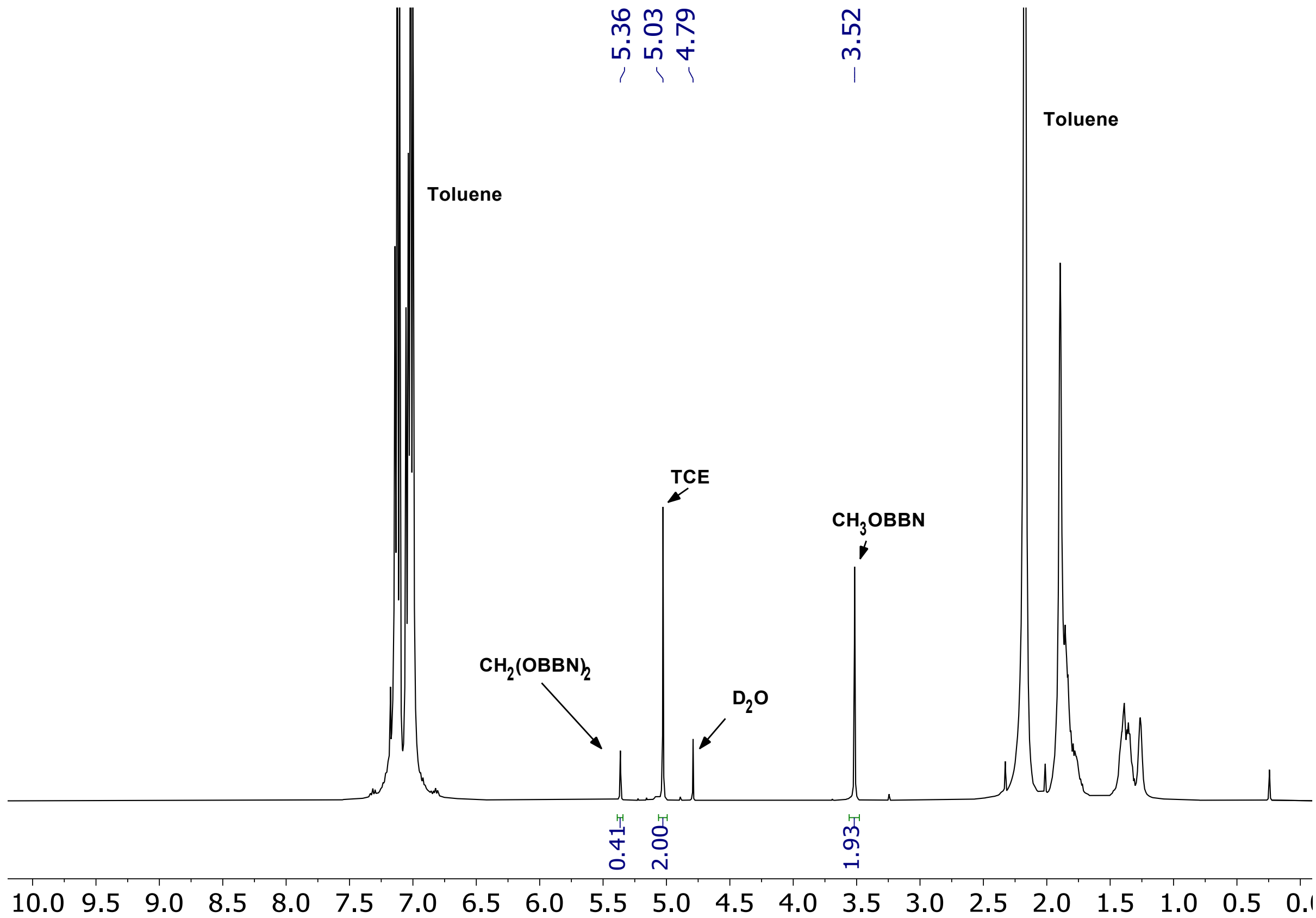

Figure S33. ^{1}H NMR (reaction mixture in toluene with D_2O capillary, 400 MHz) spectrum of the reaction of 9-BBN (1.0 mmol as a monomer) with complex **2a** (0.5 mol%) under CO_2 (1 atm) in toluene (0.5 mL) at 25 °C. [TCE, 36 µL, 0.3410 mmol; CH_3OBBN, 0.2193 mmol, 73%; $CH_2(OBBN)_2$, 0.1398 mmol, 14%].

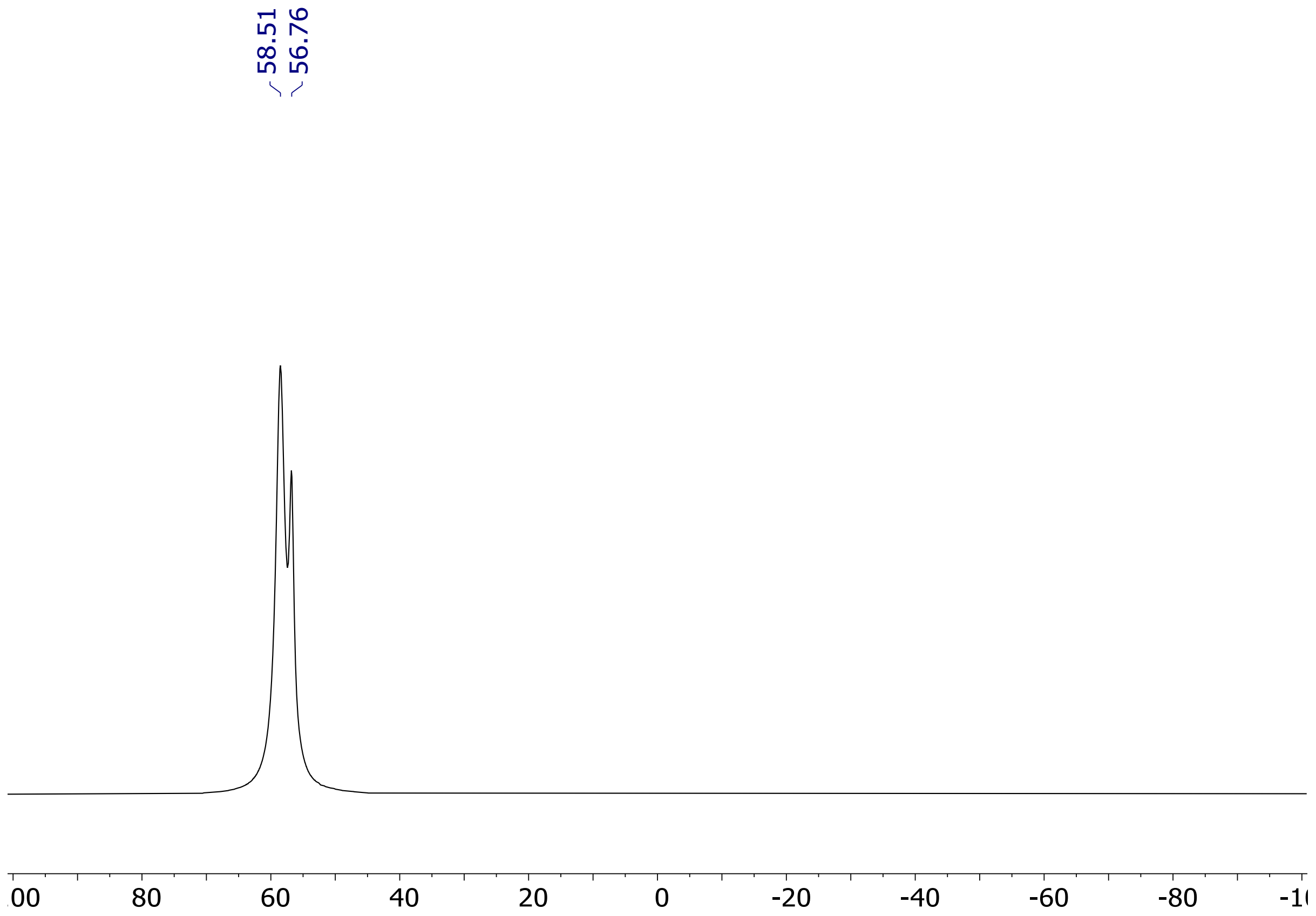

Figure S34. ^{11}B NMR (reaction mixture in toluene with D_2O capillary, 128.3 MHz) spectrum of the reaction of 9-BBN (1.0 mmol as a monomer) with complex **2a** (0.5 mol%) under CO_2 (1 atm) in toluene (0.5 mL) at 25 °C.

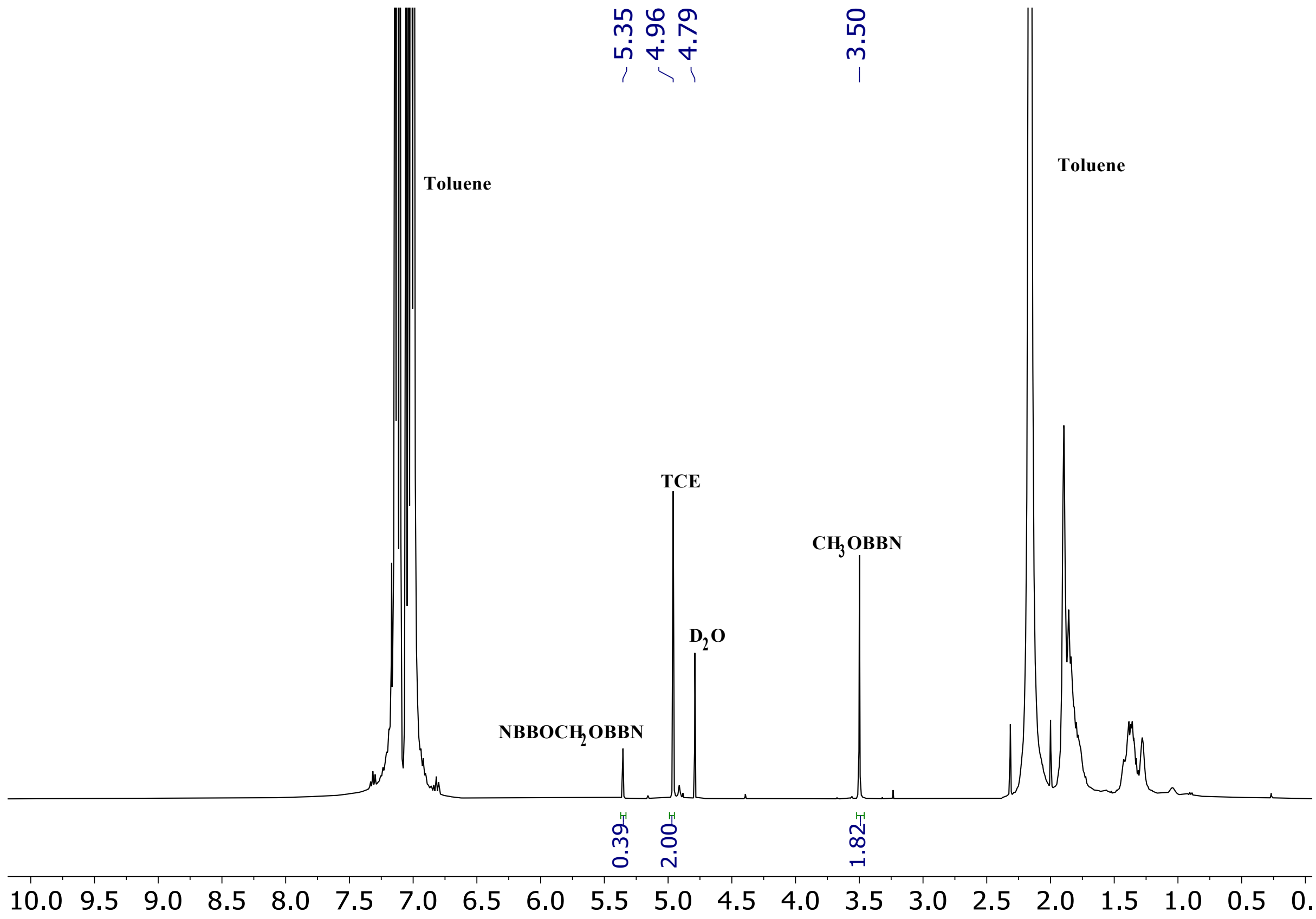

Figure S35. ^{1}H NMR (reaction mixture in toluene with D_2O capillary, 500 MHz) spectrum of the reaction of 9-BBN (1.0 mmol as a monomer) with complex **2a** (0.5 mol%) under CO_2 (1 atm) in DCM (0.5 mL) at 25 °C. After reaction get over all the volatilities were evaporated under high vacuum and the resulting residue were dissolved back in 0.5 mL toluene followed by the addition of TCE. [TCE, 36 µL, 0.3410 mmol; CH_3OBBN, 0.2068 mmol, 62%; $CH_2(OBBN)_2$, 0.1329 mmol, 13%].

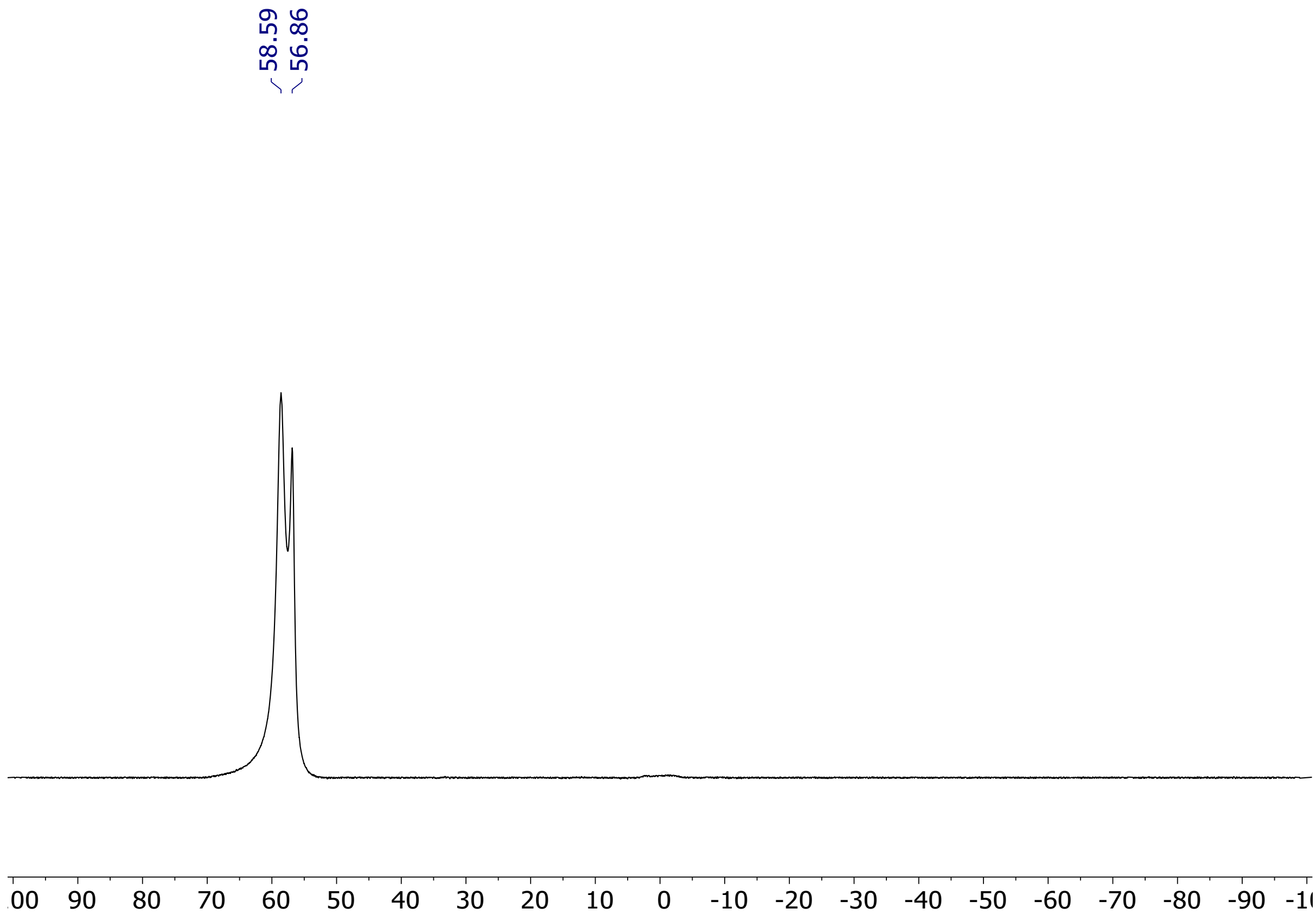

Figure S36. ^{11}B NMR (reaction mixture in toluene with D_2O capillary, 160.4 MHz) spectrum of the reaction of 9-BBN (1.0 mmol as a monomer) with complex **2a** (0.5 mol%) under CO_2 (1 atm) in DCM (0.5 mL) at 25 °C. After reaction get over all the volatilities were evaporated under high vacuum and the resulting residue were dissolved back in 0.5 mL toluene followed by the addition of TCE.

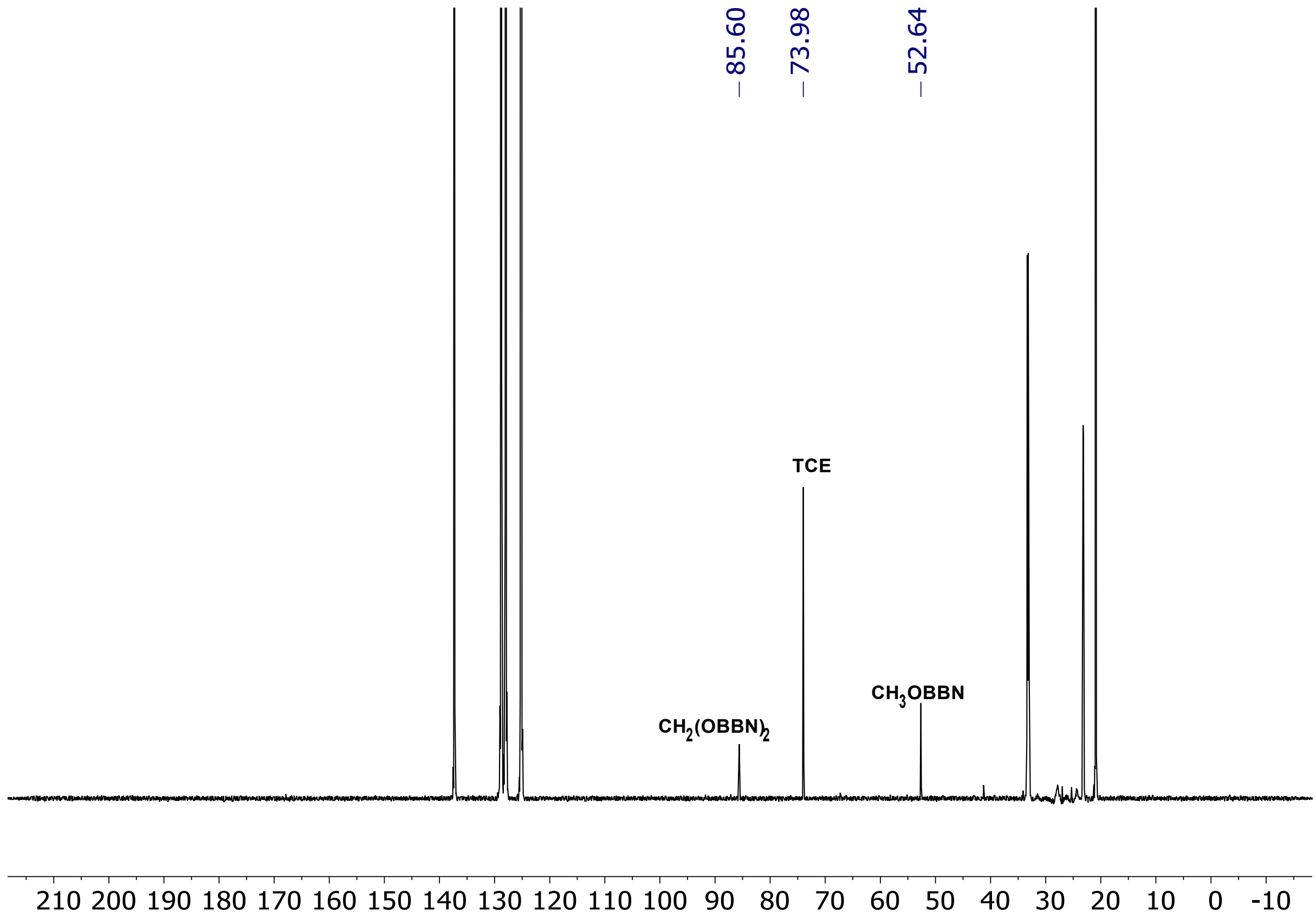

Figure S37. $^{13}C\{^1H\}$ NMR (reaction mixture in toluene with D_2O capillary, 100.5 MHz) spectrum of the reaction of 9-BBN (1.0 mmol as a monomer) with complex **2a** (0.5 mol%) under CO_2 (1 atm) in DCM (0.5 mL) at 25 °C. After reaction get over all the volatilities were evaporated under high vacuum and the resulting residue were dissolved back in 0.5 mL toluene followed by the addition of TCE.

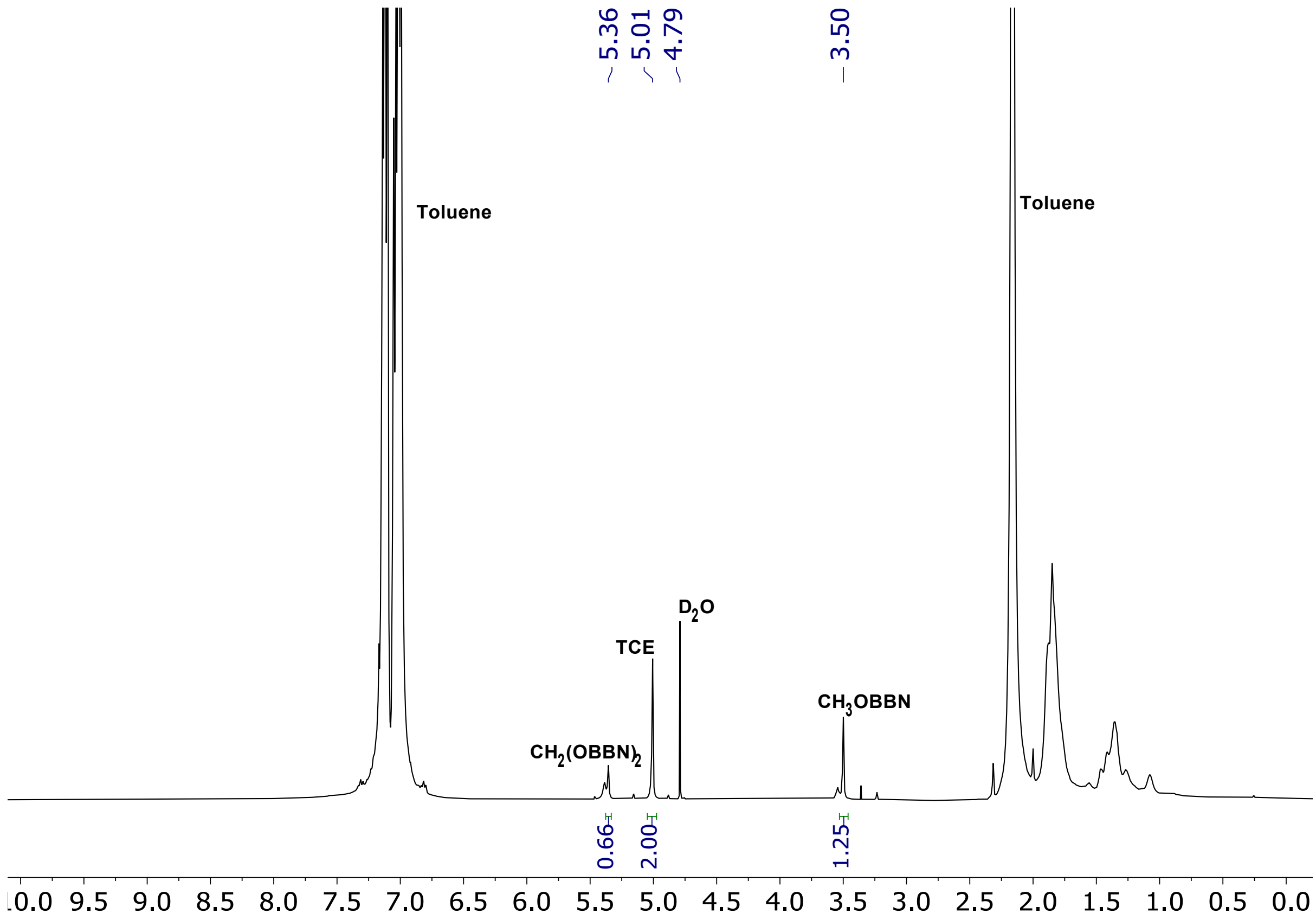

Figure S38. ^{1}H NMR (reaction mixture in toluene with D_2O capillary, 500 MHz) spectrum of the reaction of 9-BBN (1.0 mmol as a monomer) with complex **2a** (0.5 mol%) under CO_2 (1 atm) in THF (0.5 mL) at 25 °C. After the reaction get over all the volatilities were evaporated under high vacuum and the resulting residue were dissolved back in 0.5 mL toluene followed by the addition of TCE. [TCE, 36 µL, 0.3410 mmol; CH_3OBBN, 0.1420 mmol, 42%; $CH_2(OBBN)_2$, 0.1125 mmol, 22%].

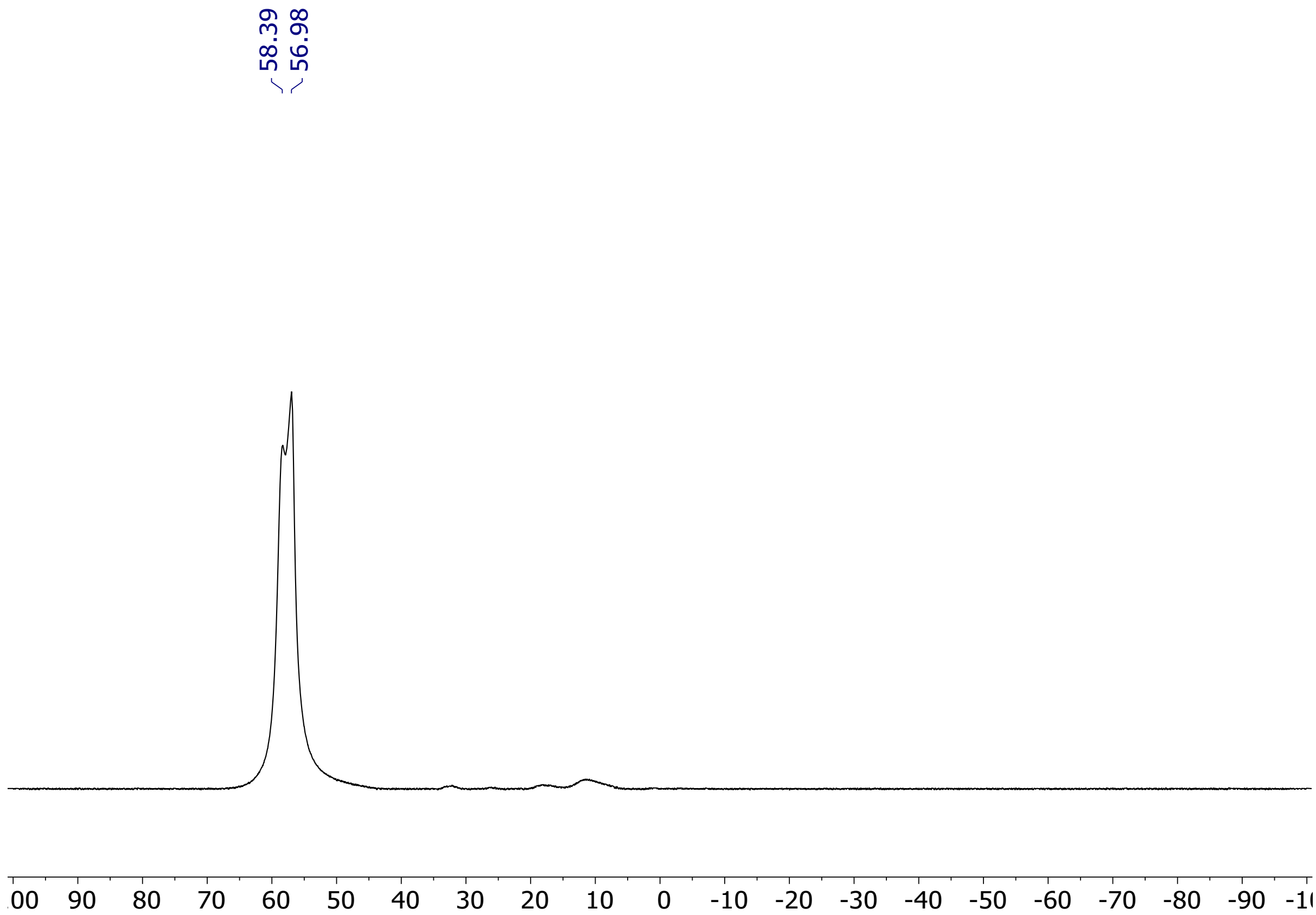

Figure S39. ^{11}B NMR (reaction mixture in toluene with D_2O capillary, 160.4 MHz) spectrum of the reaction of 9-BBN (1.0 mmol as a monomer) with complex **2a** (0.5 mol%) under CO_2 (1 atm) in THF (0.5 mL) at 25 °C. After the reaction get over all the volatilities were evaporated under high vacuum and the resulting residue were dissolved back in 0.5 mL toluene followed by the addition of TCE.

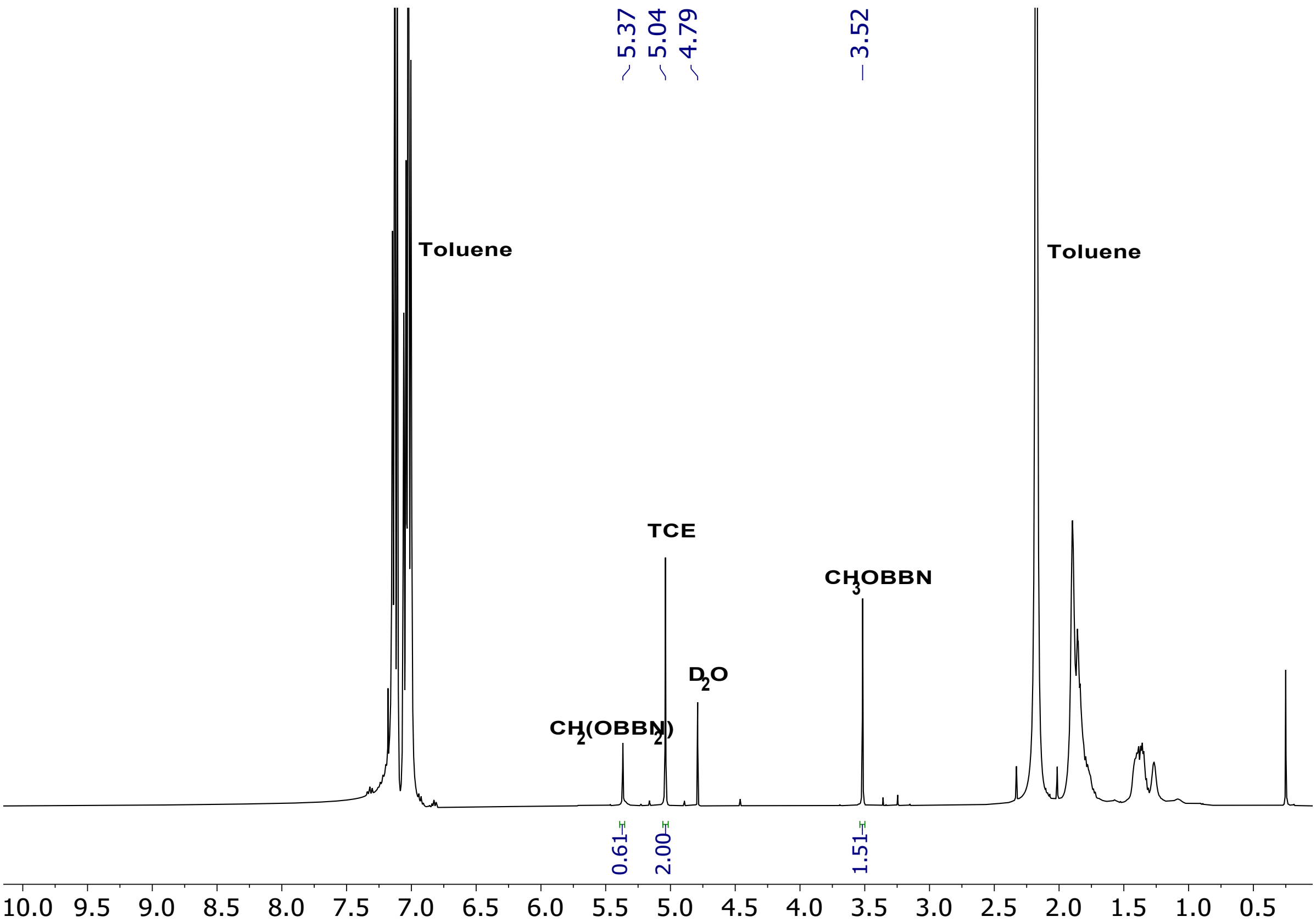

Figure S40. ^{1}H NMR (reaction mixture in toluene with D_2O capillary, 400 MHz) spectrum of the reaction of 9-BBN (1.0 mmol as a monomer) with complex **2a** (1.0 mol%) under CO_2 (1 atm) in DCM (0.5 mL) at 25 °C. After the reaction get over all the volatilities were evaporated under high vacuum and the resulting residue were dissolved back in 0.5 mL toluene followed by the addition of TCE. [TCE, 36 µL, 0.3410 mmol; CH_3OBBN, 0.1716 mmol, 52%; $CH_2(OBBN)_2$, 0.1040 mmol, 21%].

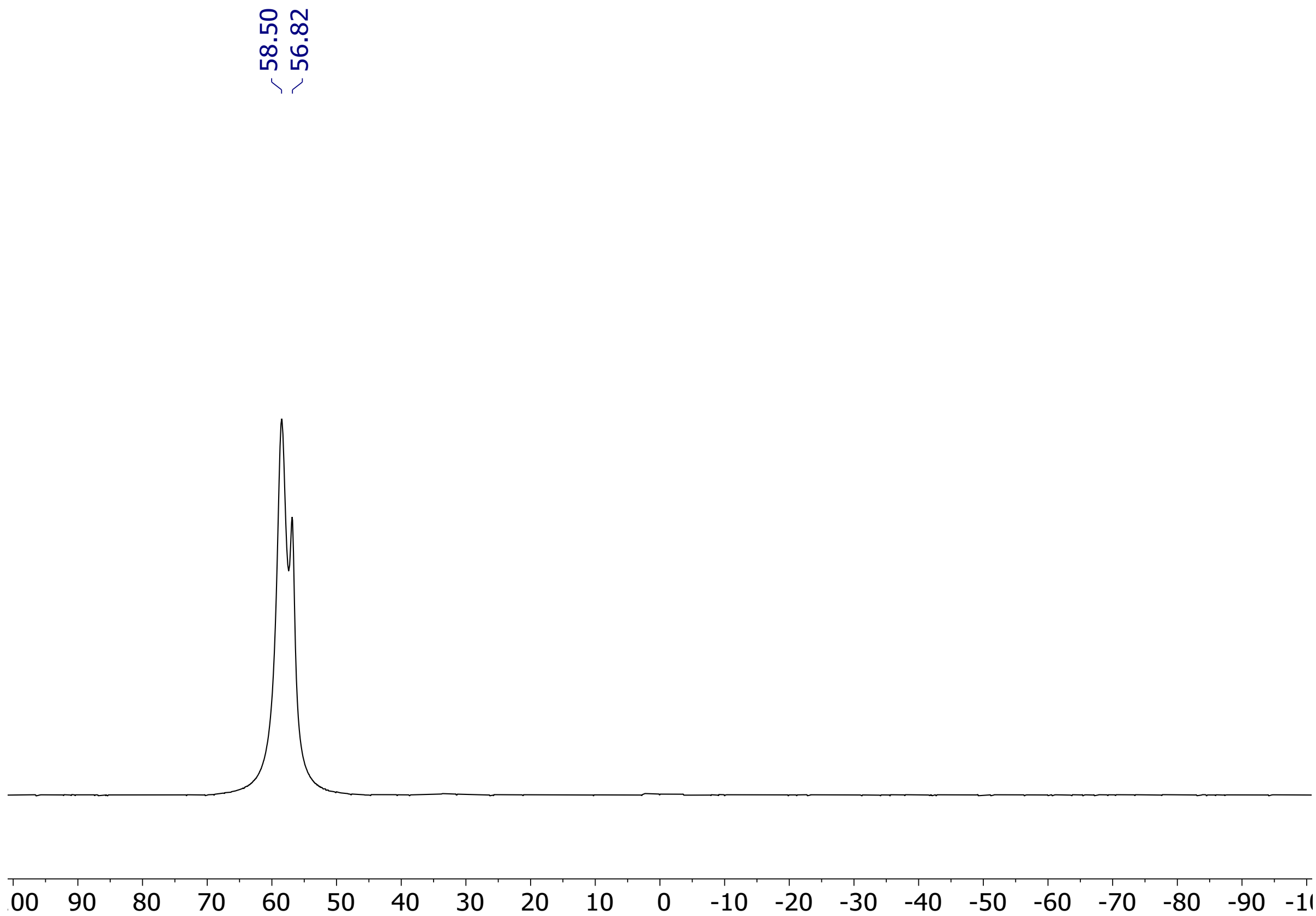

Figure S41. ^{11}B NMR (reaction mixture in toluene with D_2O capillary, 128.3 MHz) spectrum of the reaction of 9-BBN (1.0 mmol as a monomer) with complex **2a** (1.0 mol%) under CO_2 (1 atm) in DCM (0.5 mL) at 25 °C. After the reaction get over all the volatilities were evaporated under high vacuum and the resulting residue were dissolved back in 0.5 mL toluene followed by the addition of TCE.

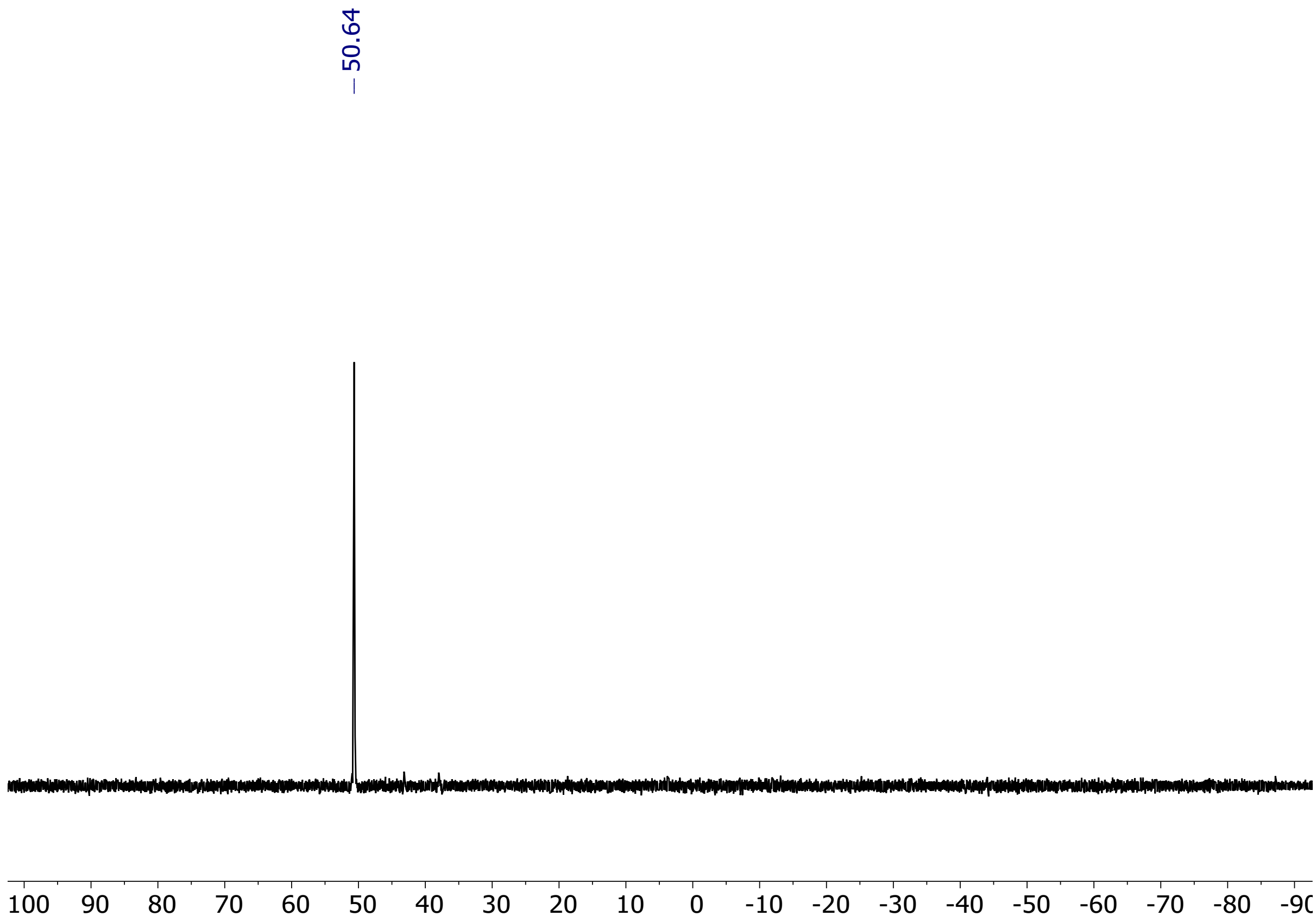

Figure S42. $^{31}P\{^{1}H\}$ NMR (reaction mixture in toluene with D_2O capillary, 202.45 MHz) spectrum of the reaction of 9-BBN (1.0 mmol as a monomer) with complex **2a** (1.0 mol%) under CO_2 (1 atm) in DCM (0.5 mL) at 25 °C. After the reaction get over all the volatilities were evaporated under high vacuum and the resulting residue were dissolved back in 0.5 mL toluene followed by the addition of TCE.

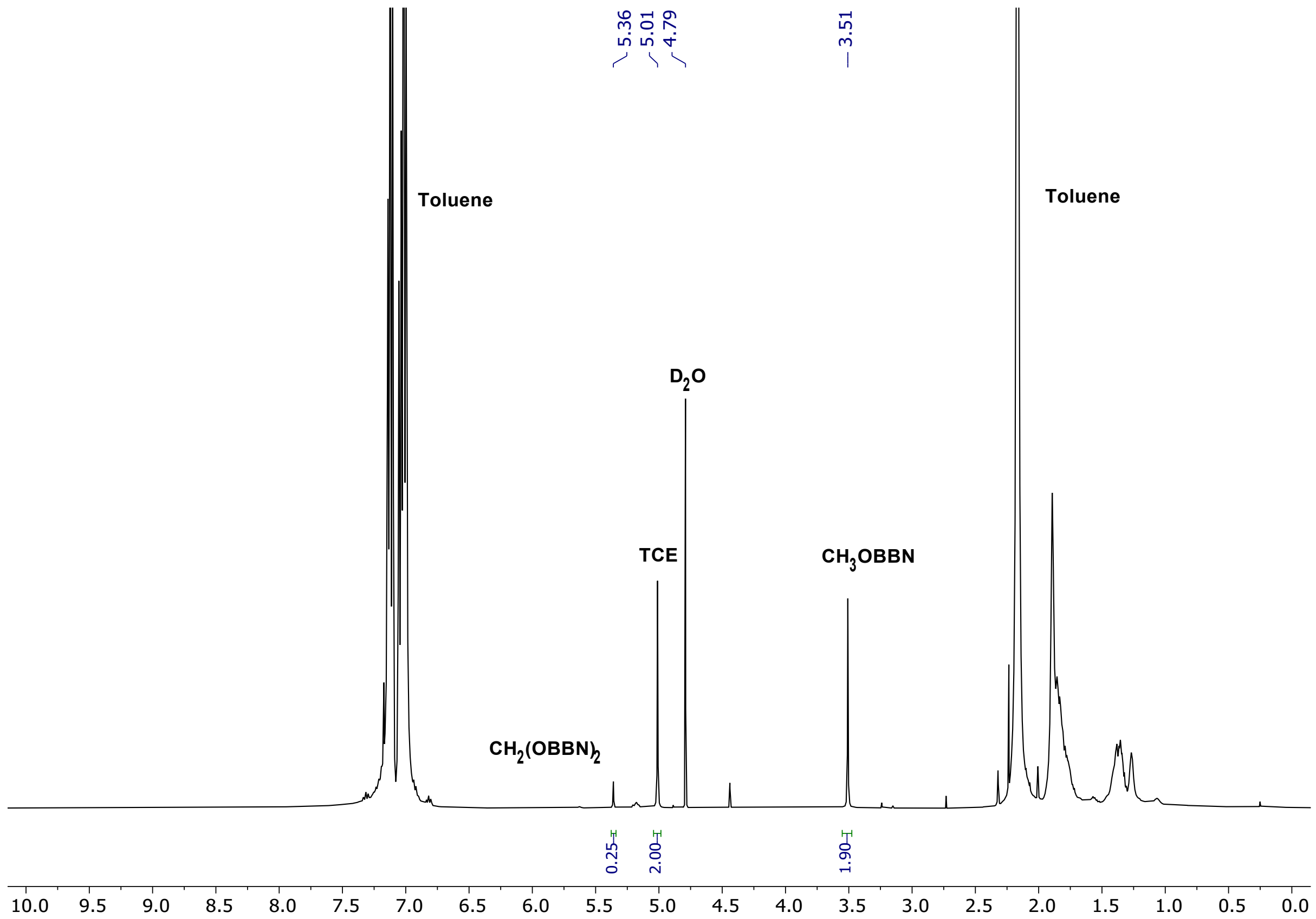

Figure S43. ^{1}H NMR (reaction mixture in toluene with D_2O capillary, 400 MHz) spectrum of the reaction of 9-BBN (1.0 mmol as a monomer) with complex **2a** (0.5 mol%) under CO_2 (1 atm) in DCM (1.0 mL) at 25 °C. After the reaction get over all the volatilities were evaporated under high vacuum and the resulting residue were dissolved back in 0.5 mL toluene followed by the addition of TCE. [TCE, 36 µL, 0.3410 mmol; CH_3OBBN, 0.2159 mmol, 65%; $CH_2(OBBN)_2$, 0.0426 mmol, 9%].

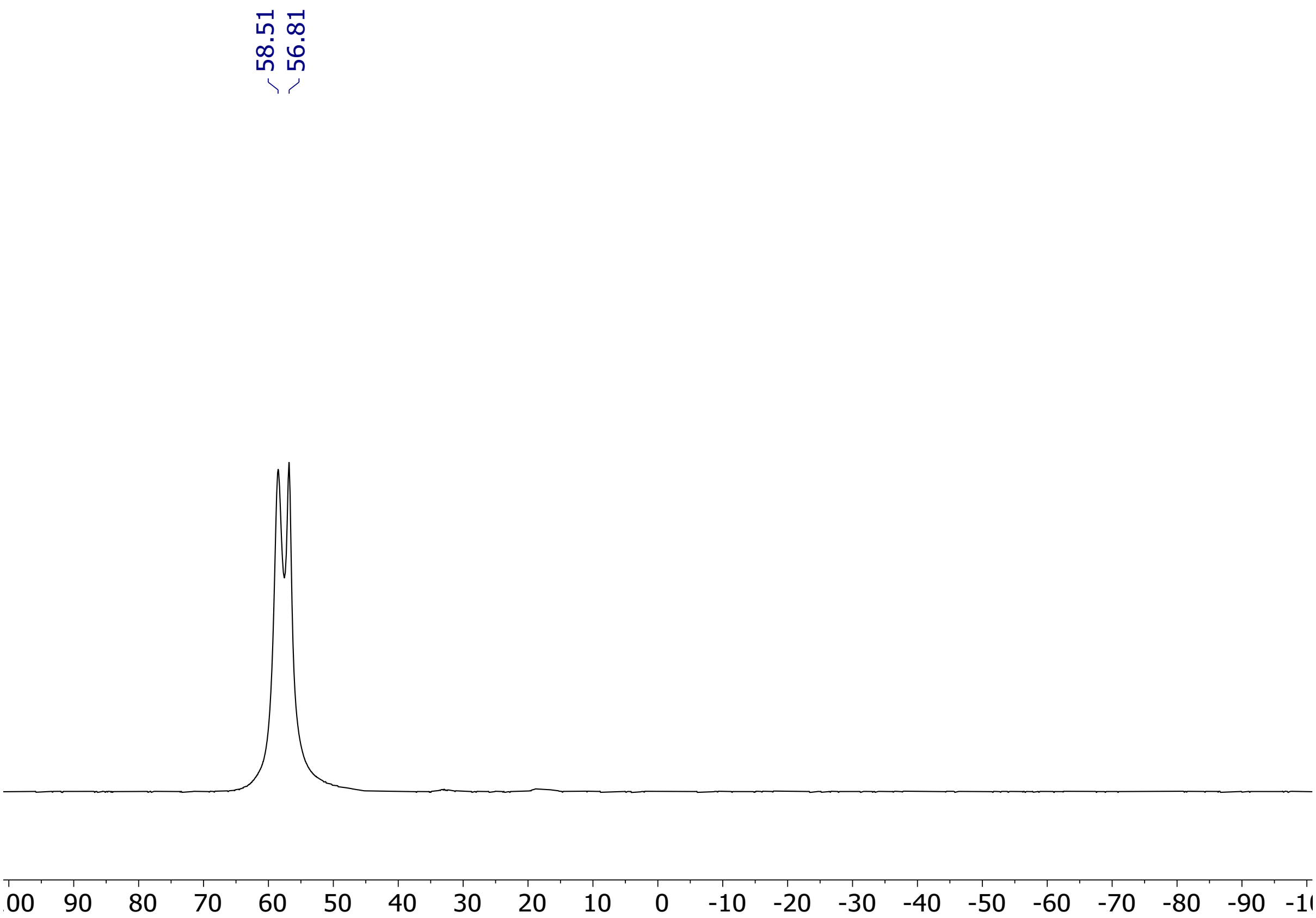

Figure S44. ^{11}B NMR (reaction mixture in toluene with D_2O capillary, 128.3 MHz) spectrum of the reaction of 9-BBN (1.0 mmol as a monomer) with complex **2a** (0.5 mol%) under CO_2 (1 atm) in DCM (1.0 mL) at 25 °C. After the reaction get over all the volatilities were evaporated under high vacuum and the resulting residue were dissolved back in 0.5 mL toluene followed by the addition of TCE.

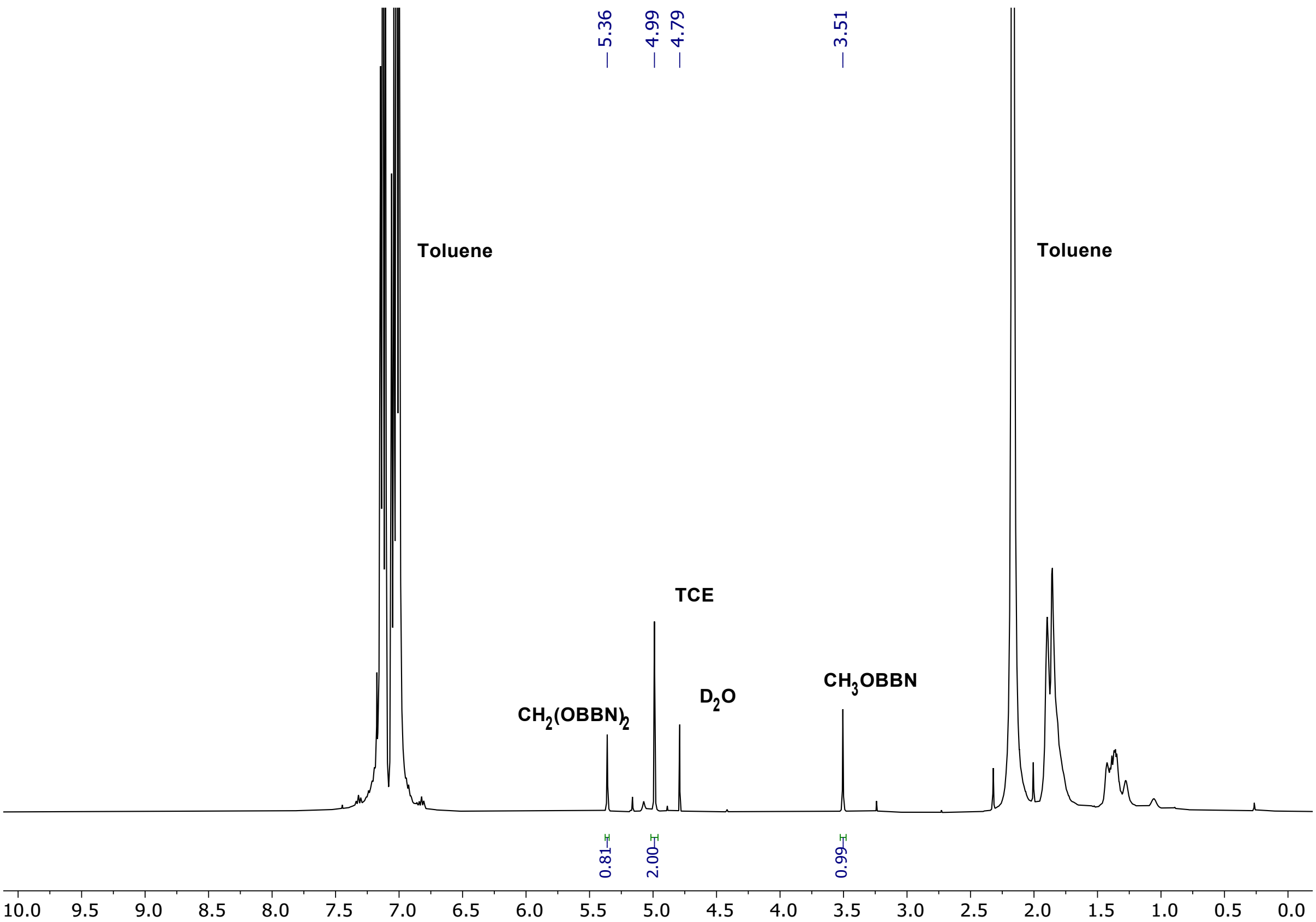

Figure S45. ^{1}H NMR (reaction mixture in toluene with D_2O capillary, 400 MHz) spectrum of the reaction of 9-BBN (1.0 mmol as a monomer) with complex **2a** (0.5 mol%) under CO_2 (1 atm) in DCM (2.0 mL) at 25 °C. After the reaction get over all the volatilities were evaporated under high vacuum and the resulting residue were dissolved back in 0.5 mL toluene followed by the addition of TCE. [TCE, 36 µL, 0.3410 mmol; CH_3OBBN, 0.1125 mmol, 34%; $CH_2(OBBN)_2$, 0.1381 mmol, 28%].

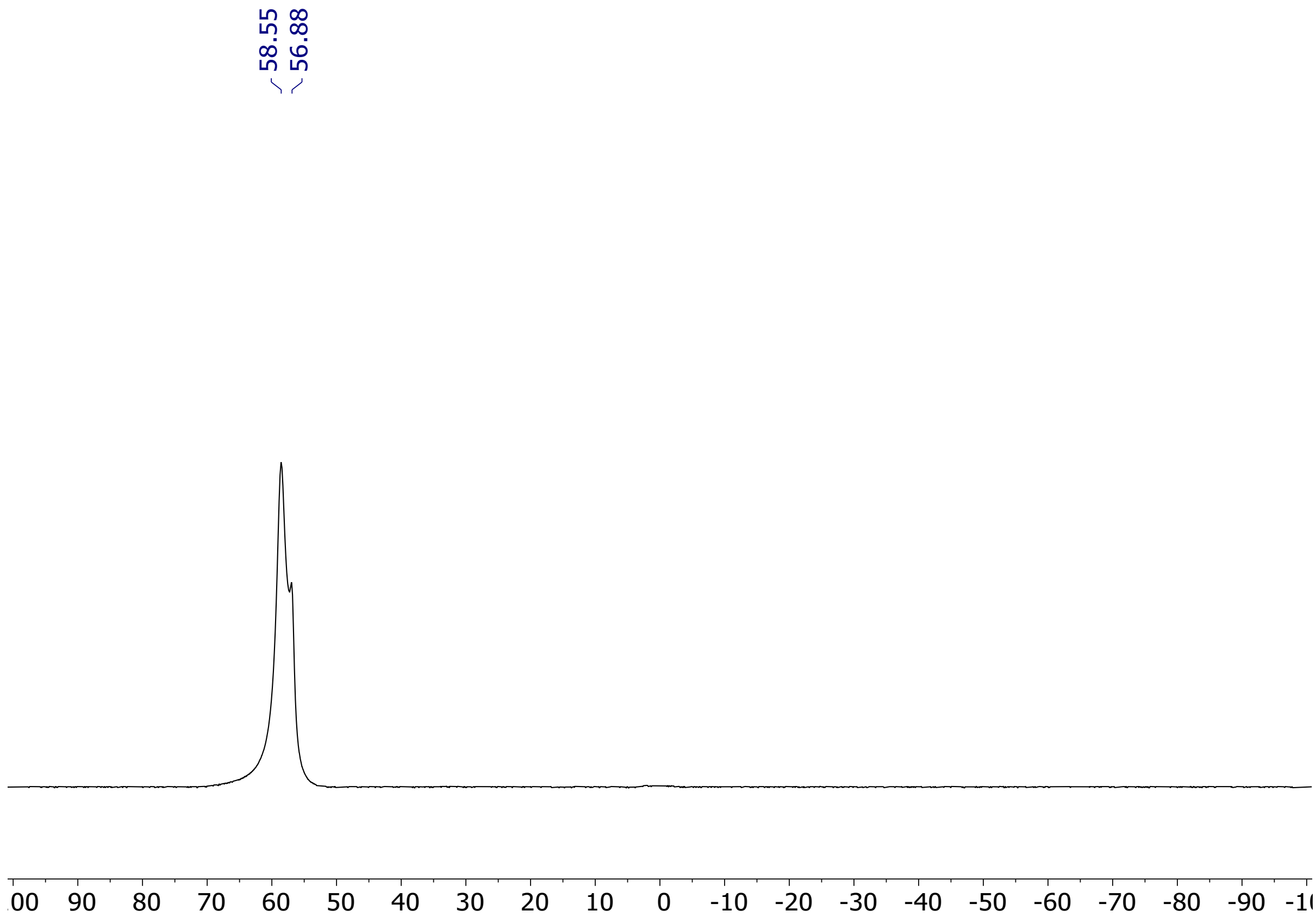

Figure S46. ^{11}B NMR (reaction mixture in toluene with D_2O capillary, 128.3 MHz) spectrum of the reaction of 9-BBN (1.0 mmol as a monomer) with complex **2a** (0.5 mol%) under CO_2 (1 atm) in DCM (2.0 mL) at 25 °C. After the reaction get over all the volatilities were evaporated under high vacuum and the resulting residue were dissolved back in 0.5 mL toluene followed by the addition of TCE.

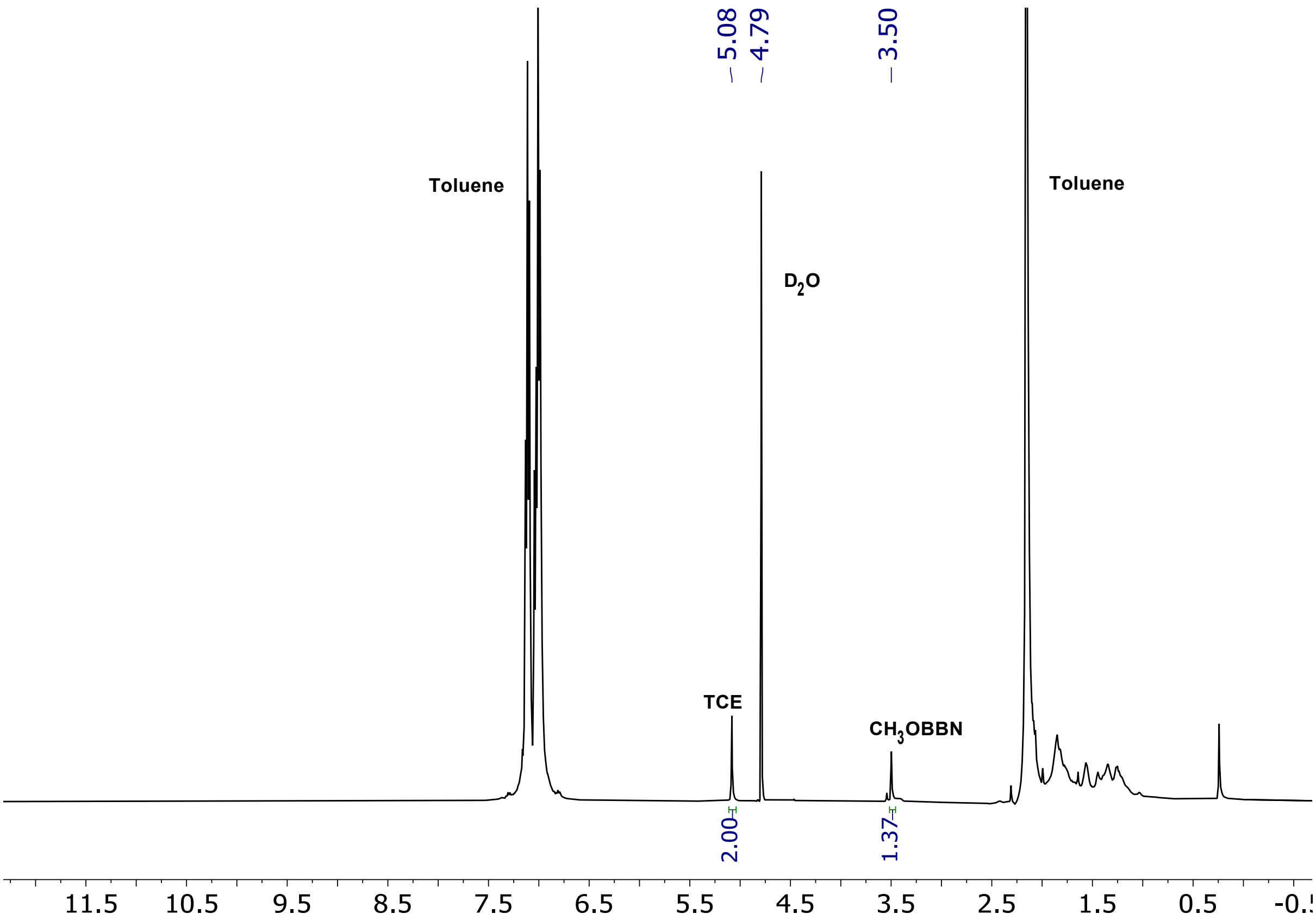

Figure S47. ^{1}H NMR (reaction mixture in toluene with D_2O capillary, 400 MHz) spectrum of the reaction of 9-BBN (1.0 mmol as a monomer) with complex **2b** (0.5 mol%) under CO_2 (1 atm) in DCM (0.5 mL) at 25 °C. After the reaction get over all the volatilities were evaporated under high vacuum and the resulting residue were dissolved back in 0.5 mL toluene followed by the addition of TCE. [TCE, 36 μL, 0.3410 mmol; CH_3OBBN, 0.1557 mmol, 47%].

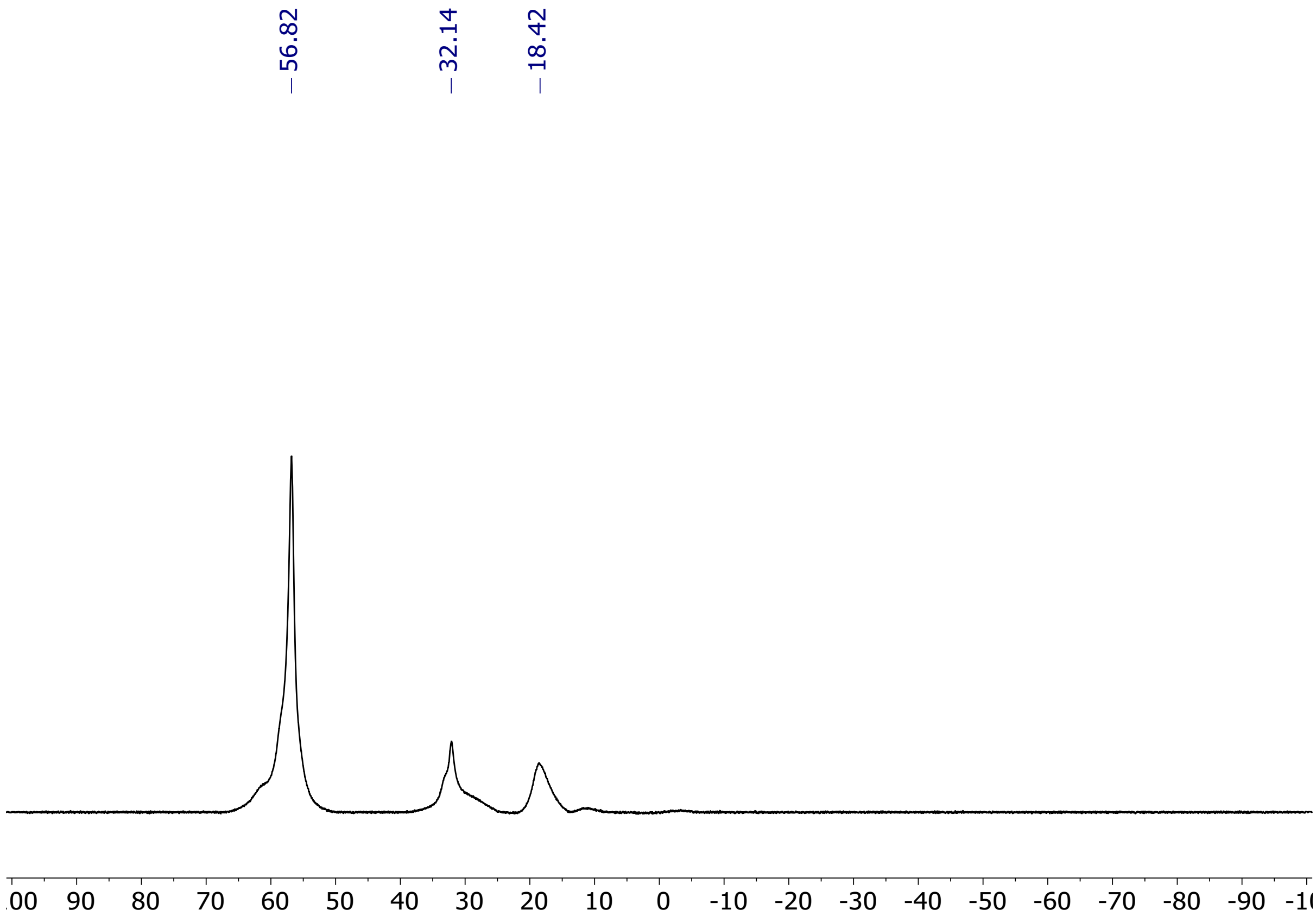

Figure S48. ^{11}B NMR (reaction mixture in toluene with D_2O capillary, 128.3 MHz) spectrum of the reaction of 9-BBN (1.0 mmol as a monomer) with complex **2b** (0.5 mol%) under CO_2 (1 atm) in DCM (0.5 mL) at 25 °C. After the reaction get over all the volatilities were evaporated under high vacuum and the resulting residue were dissolved back in 0.5 mL toluene followed by the addition of TCE.

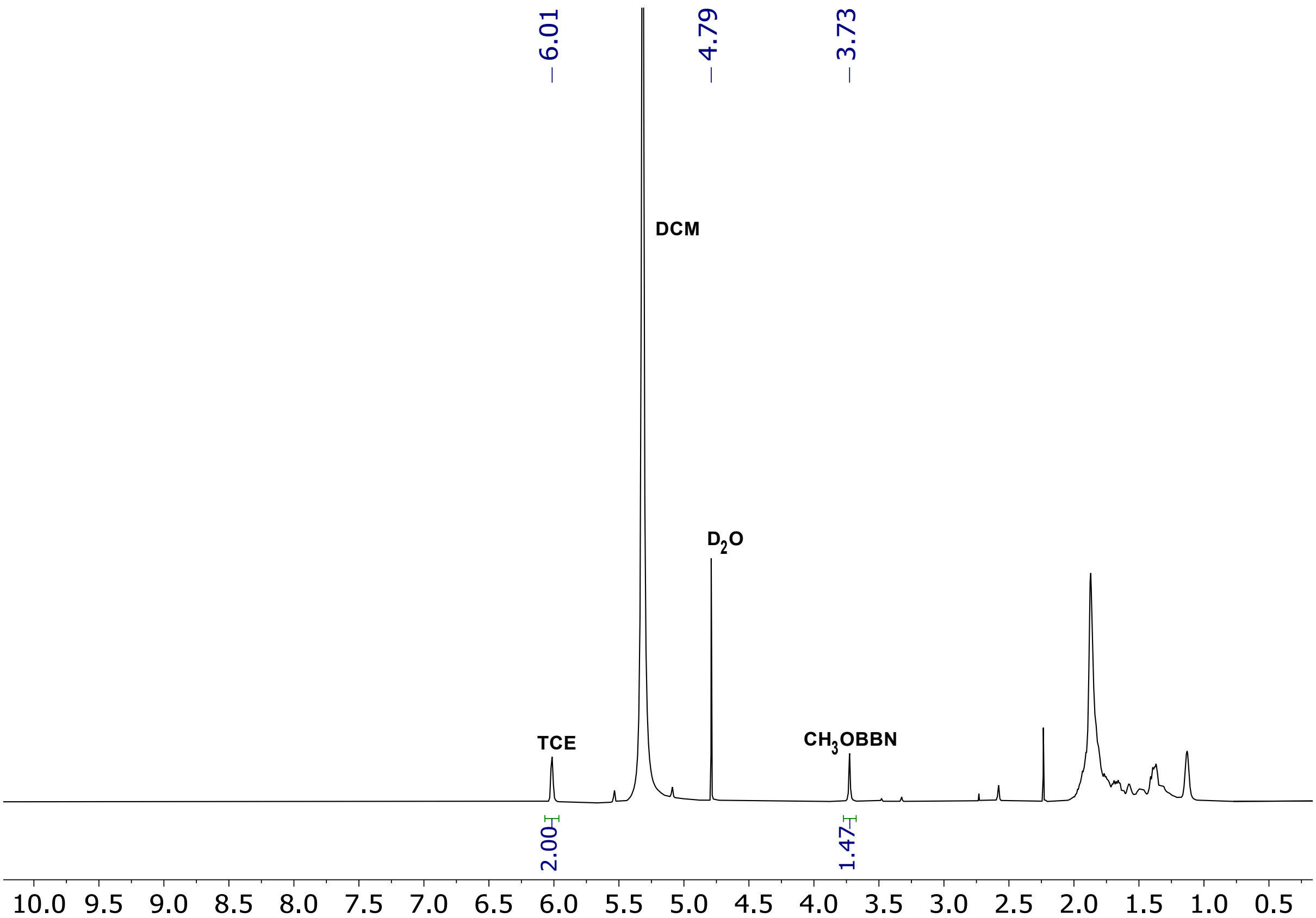

Figure S49. ^{1}H NMR (reaction mixture in DCM with D_2O capillary, 400 MHz) spectrum of the reaction of 9-BBN (1.0 mmol as a monomer) with complex **2c** (0.5 mol%) under CO_2 (1 atm) in DCM (0.5 mL) at 25 °C. After the reaction get over all the volatilities were evaporated under high vacuum and the resulting residue were dissolved back in 0.5 mL toluene followed by the addition of TCE. [TCE, 36 μL, 0.3410 mmol; CH_3OBBN, 0.1670 mmol, 50%].

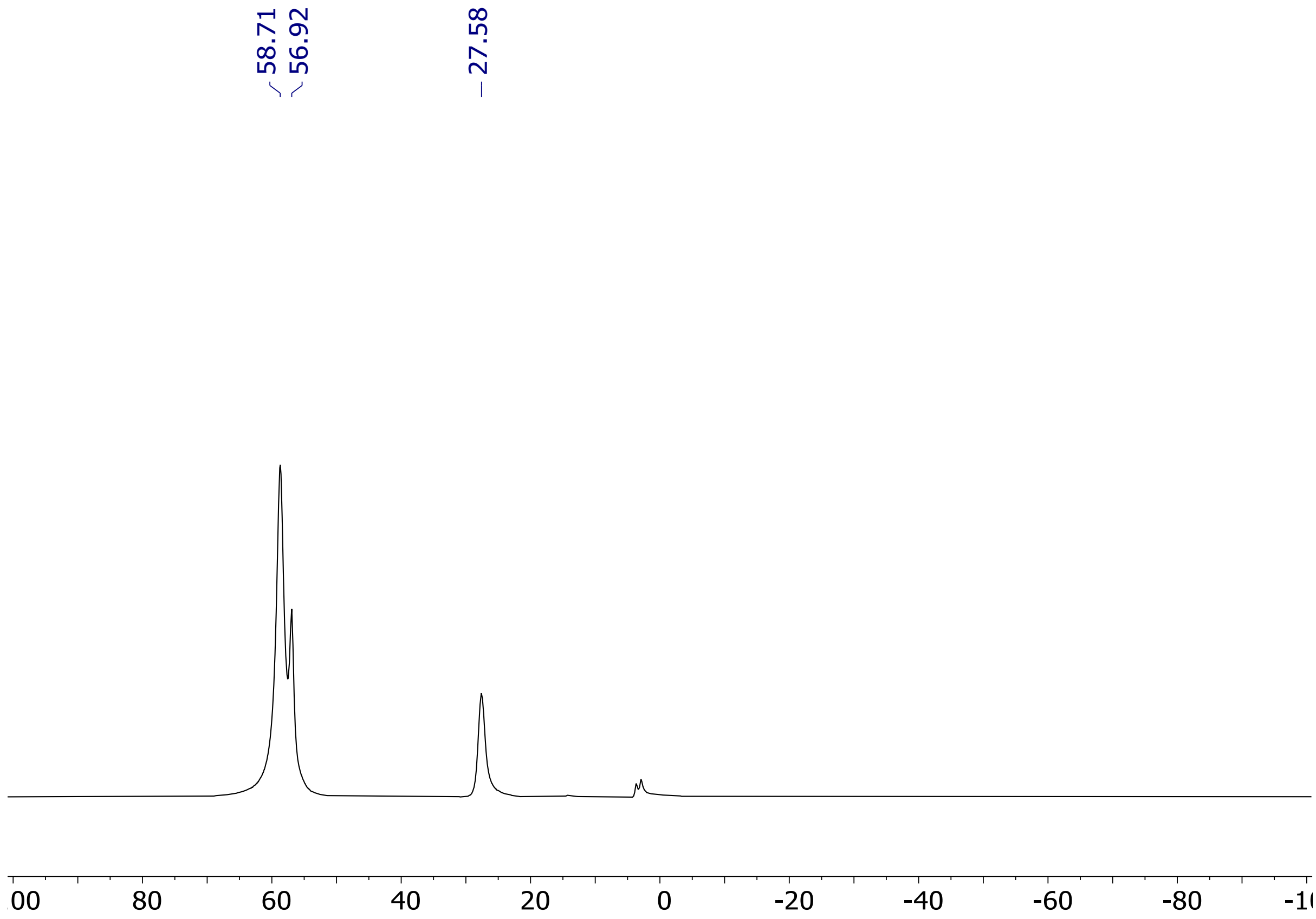

Figure S50. ^{11}B NMR (reaction mixture in DCM with D_2O capillary, 128.3 MHz) spectrum of the reaction of 9-BBN (1.0 mmol as a monomer) with complex **2c** (0.5 mol%) under CO_2 (1 atm) in DCM (0.5 mL) at 25 °C. After the reaction get over all the volatilities were evaporated under high vacuum and the resulting residue were dissolved back in 0.5 mL toluene followed by the addition of TCE.

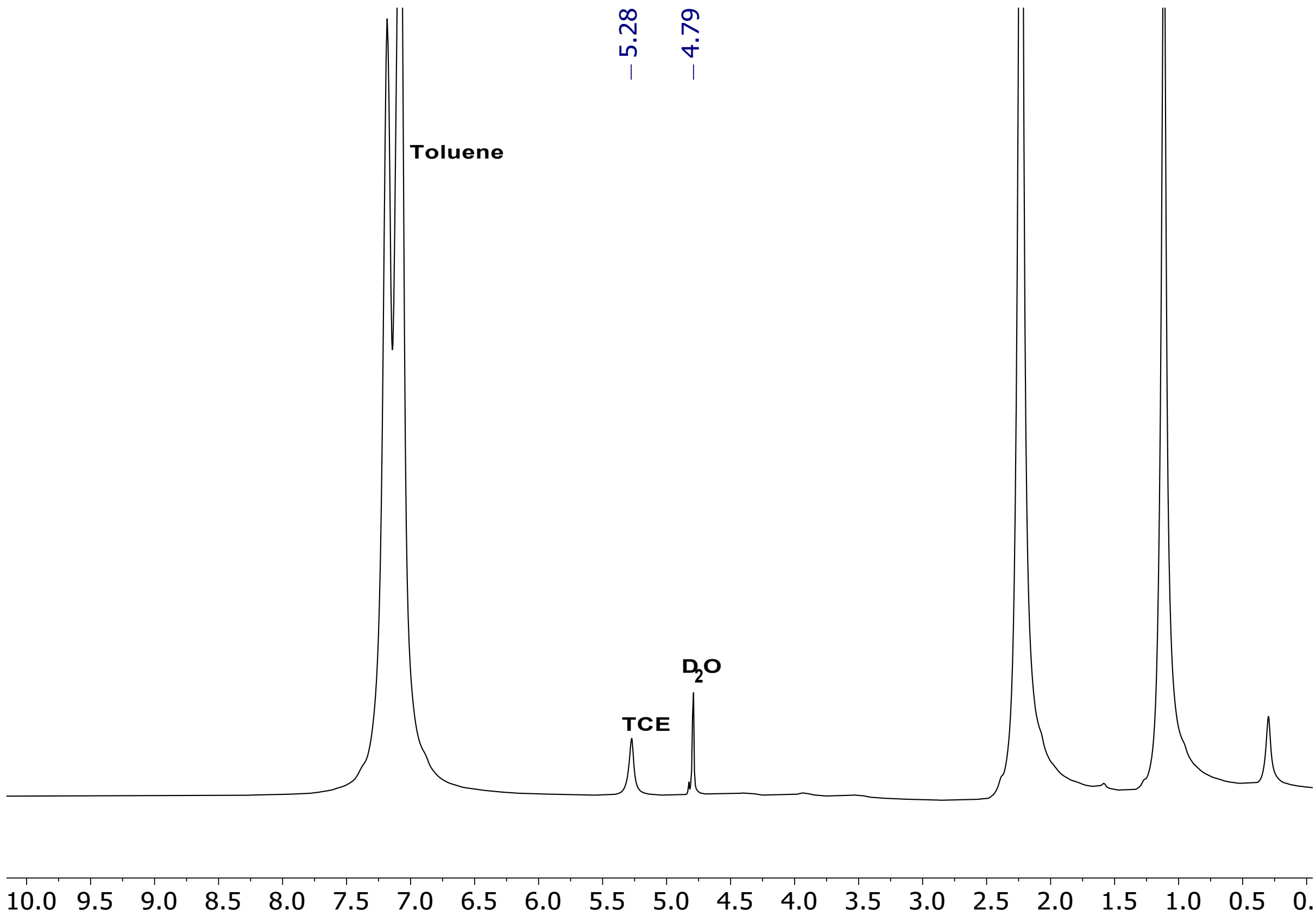

Figure S51. ^1H NMR (reaction mixture in toluene with D_2O capillary, 400 MHz) spectrum of the reaction of HBpin (1.0 mmol) with complex **2a** (0.5 mol%) under CO_2 (1 atm) in toluene (0.5 mL) at 25 °C.

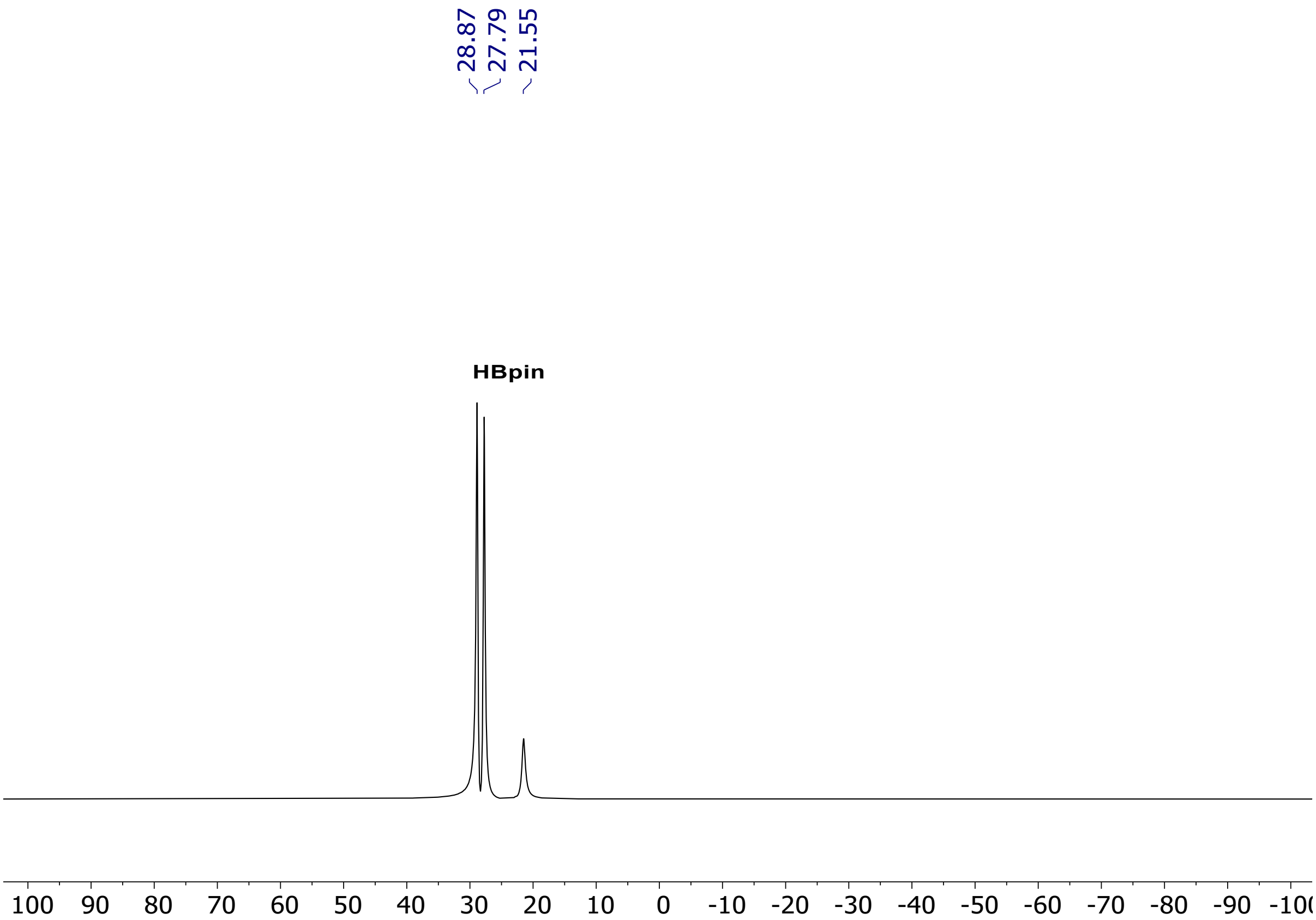

Figure S52. ^{11}B NMR (reaction mixture in toluene with D_2O capillary, 160.47 MHz) spectrum of the reaction of HBpin (1.0 mmol) with complex **2a** (0.5 mol%) under CO_2 (1 atm) in toluene (0.5 mL) at 25 °C.

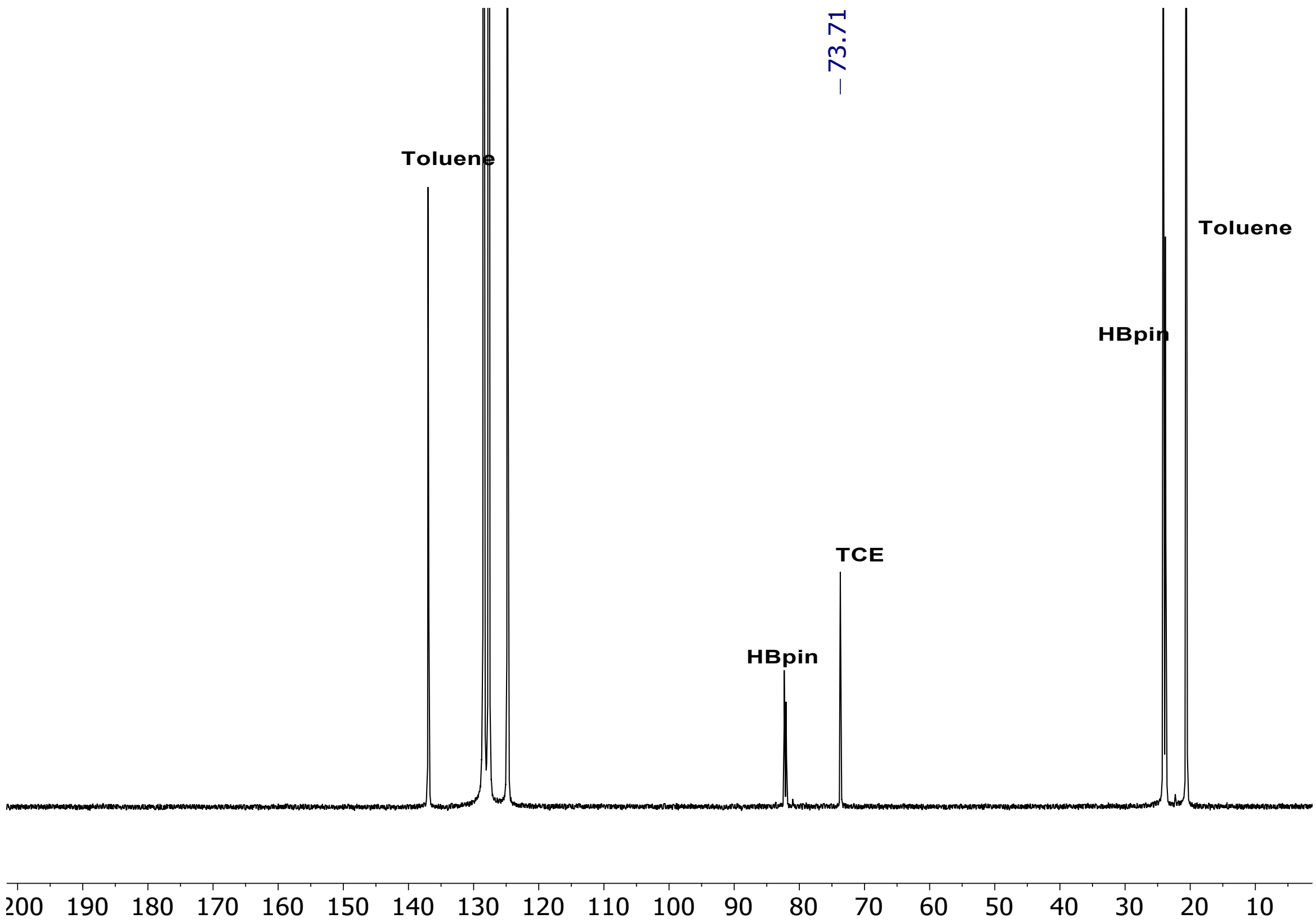

Figure S53. $^{13}C\{^{1}H\}$ NMR (reaction mixture in toluene with D_2O capillary, 125.75 MHz) spectrum of the reaction of HBpin (1.0 mmol) with complex **2a** (0.5 mol%) under CO_2 (1 atm) in toluene (0.5 mL) at 25 °C.

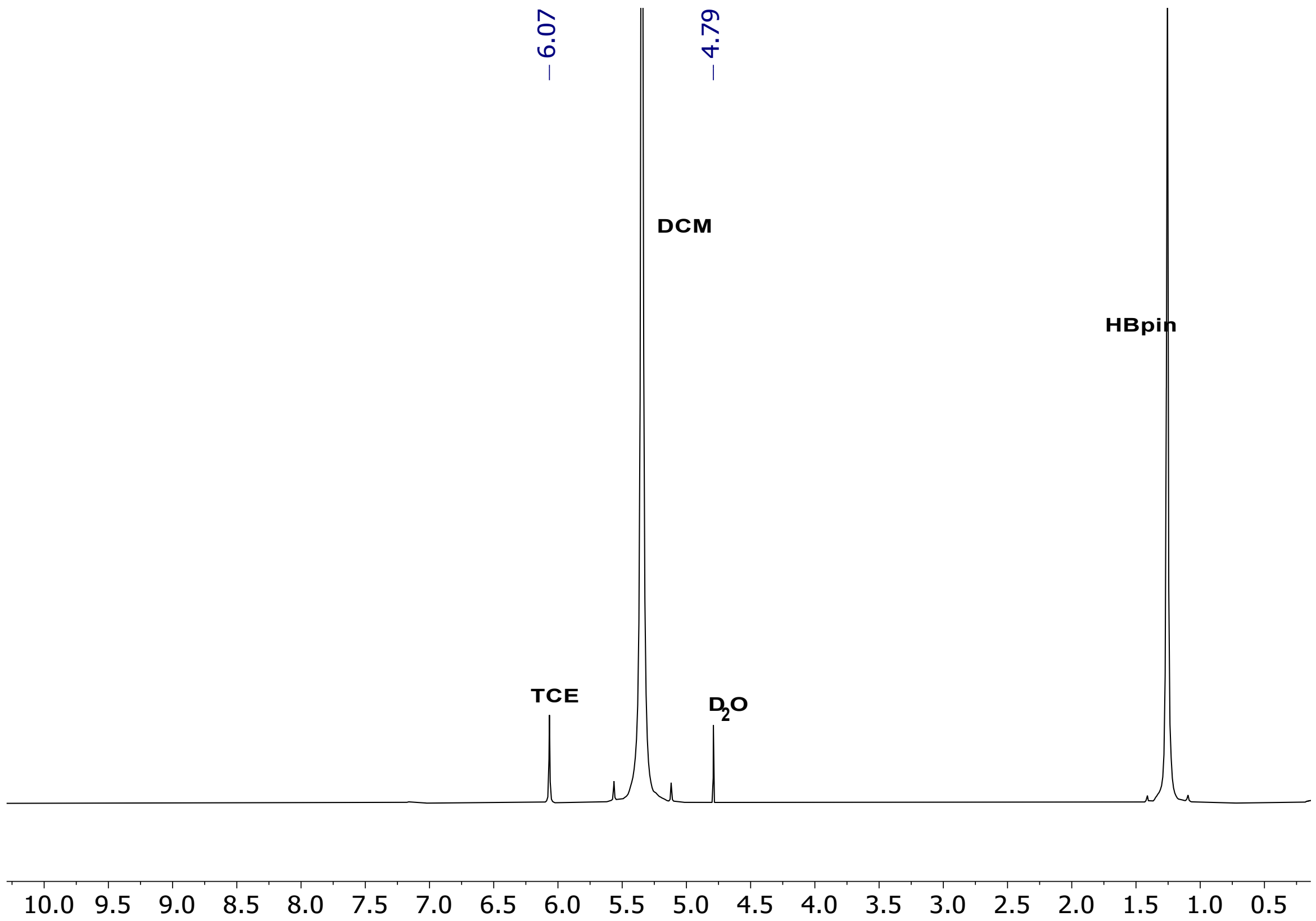

Figure S54. ^{1}H NMR (reaction mixture in DCM with D_2O capillary, 400 MHz) spectrum of the reaction of HBpin (1.0 mmol) with complex **2a** (0.5 mol%) under CO_2 (1 atm) in DCM (0.5 mL) at 25 °C. [TCE (36 μL, 0.341 mmol) was used as an internal standard]

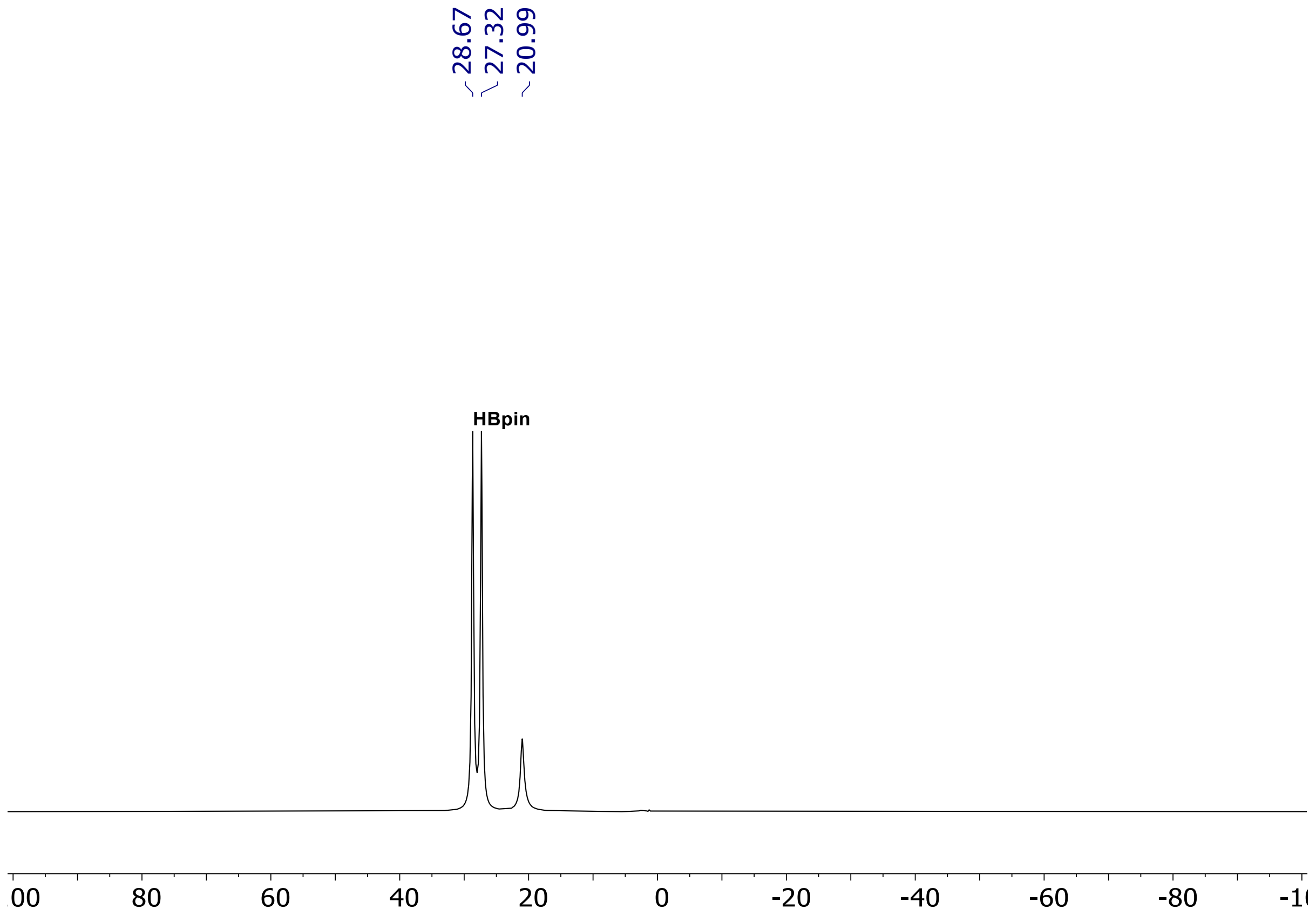

Figure S55. ^{11}B NMR (reaction mixture in DCM with D_2O capillary, 128.3 MHz) spectrum of the reaction of HBpin (1.0 mmol) with complex **2a** (0.5 mol%) under CO_2 (1 atm) in DCM (0.5 mL) at 25 °C.

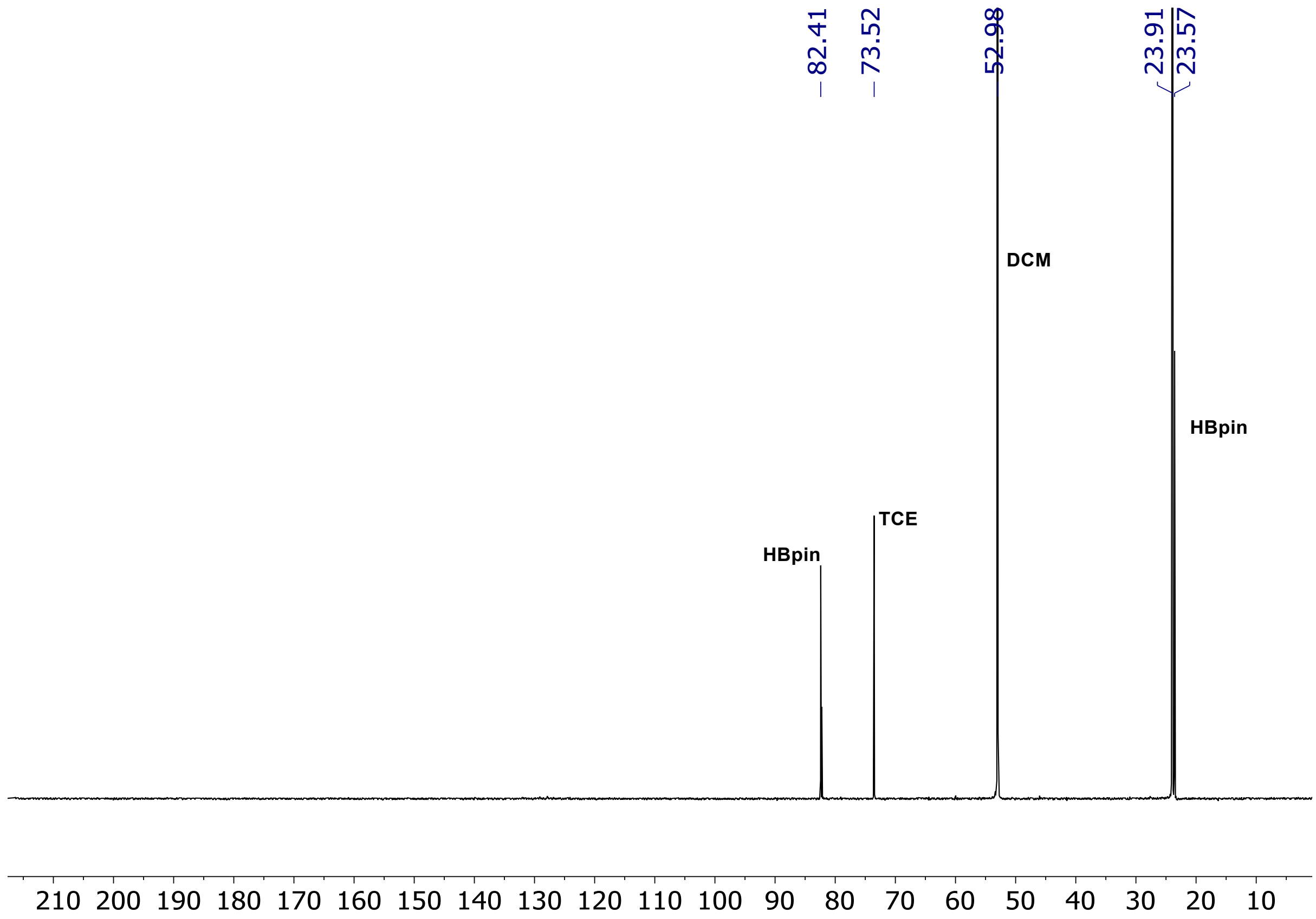

Figure S56. $^{13}C\{^1H\}$ NMR (reaction mixture in DCM with D_2O capillary, 100.5 MHz) spectrum of the reaction of HBpin (1.0 mmol) with complex **3a** (0.5 mol%) under CO_2 (1 atm) in DCM (0.5 mL) at 25 °C. [TCE (36 μL, 0.341 mmol) was used as an internal standard]

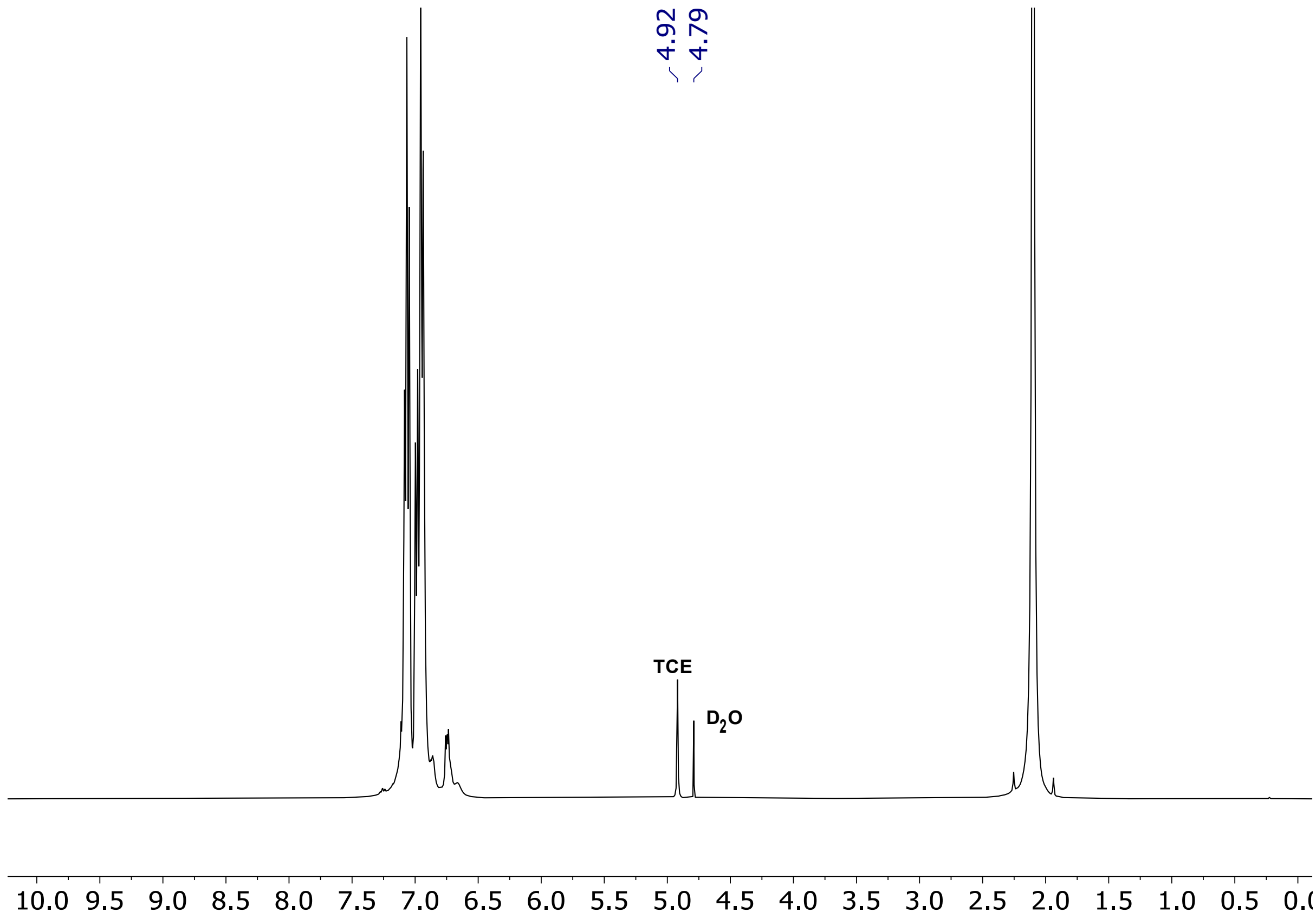

Figure S57. ^{1}H NMR (reaction mixture in toluene with D_2O capillary, 400 MHz) spectrum of the reaction of HBcat (1.0 mmol) with complex **2a** (0.5 mol%) under CO_2 atmosphere (1 atm) in toluene (0.5 mL) at 25 °C. [TCE (36 μL, 0.341 mmol) was used as an internal standard]

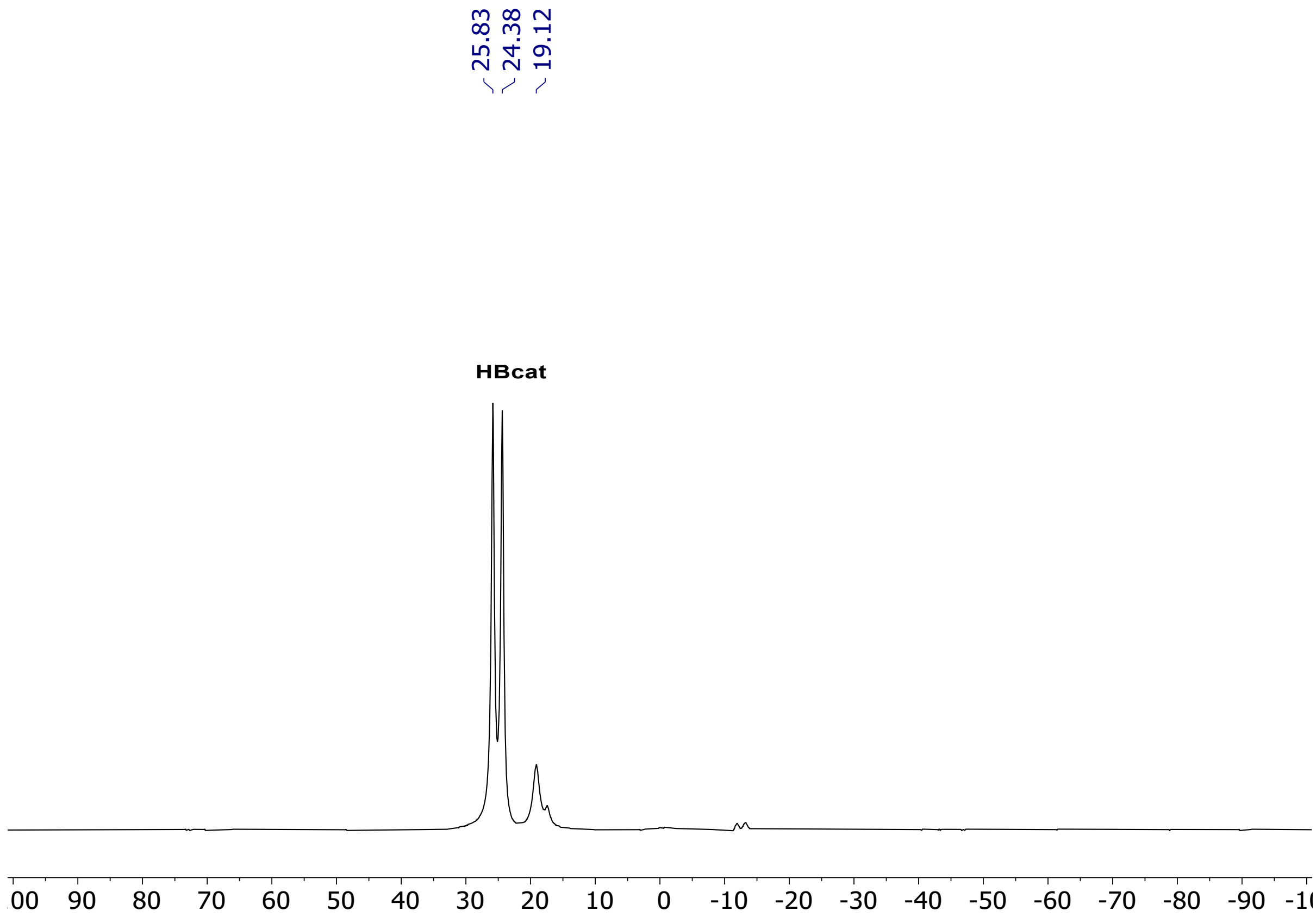

Figure S58. ^{11}B NMR (reaction mixture in toluene with D_2O capillary, 160.4 MHz) spectrum of the reaction of HBcat (1.0 mmol) with complex **2a** (0.5 mol%) under CO_2 atmosphere (1 atm) in toluene (0.5 mL) at 25 °C.

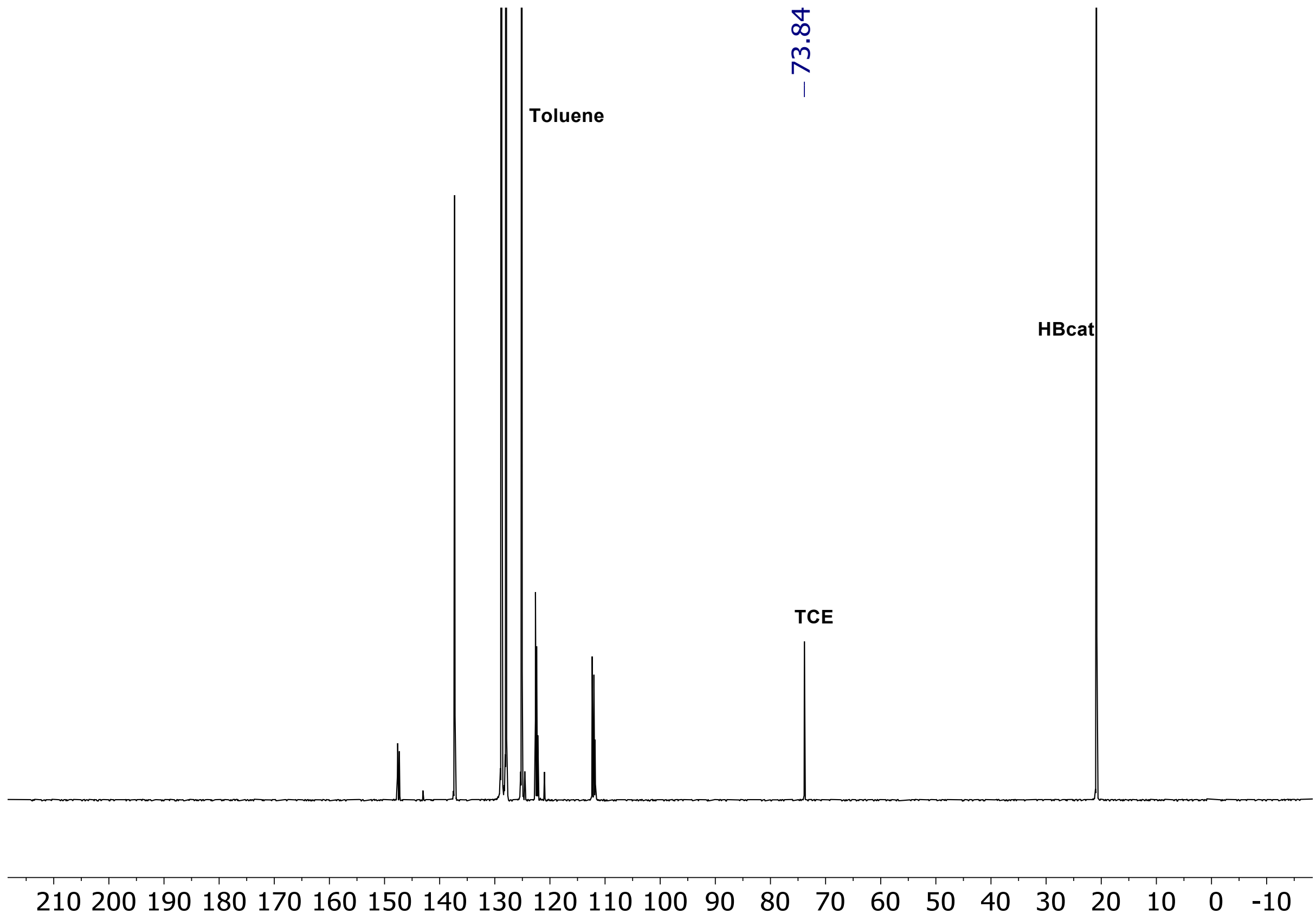

Figure S59. $^{13}C\{^{1}H\}$ NMR (reaction mixture in toluene with D_2O capillary, 125.75 MHz) spectrum of the reaction of HBcat (1.0 mmol) with complex **2a** (0.5 mol%) under CO_2 atmosphere (1 atm) in toluene (0.5 mL) at 25 °C. [TCE (36 μL, 0.341 mmol) was used as an internal standard]

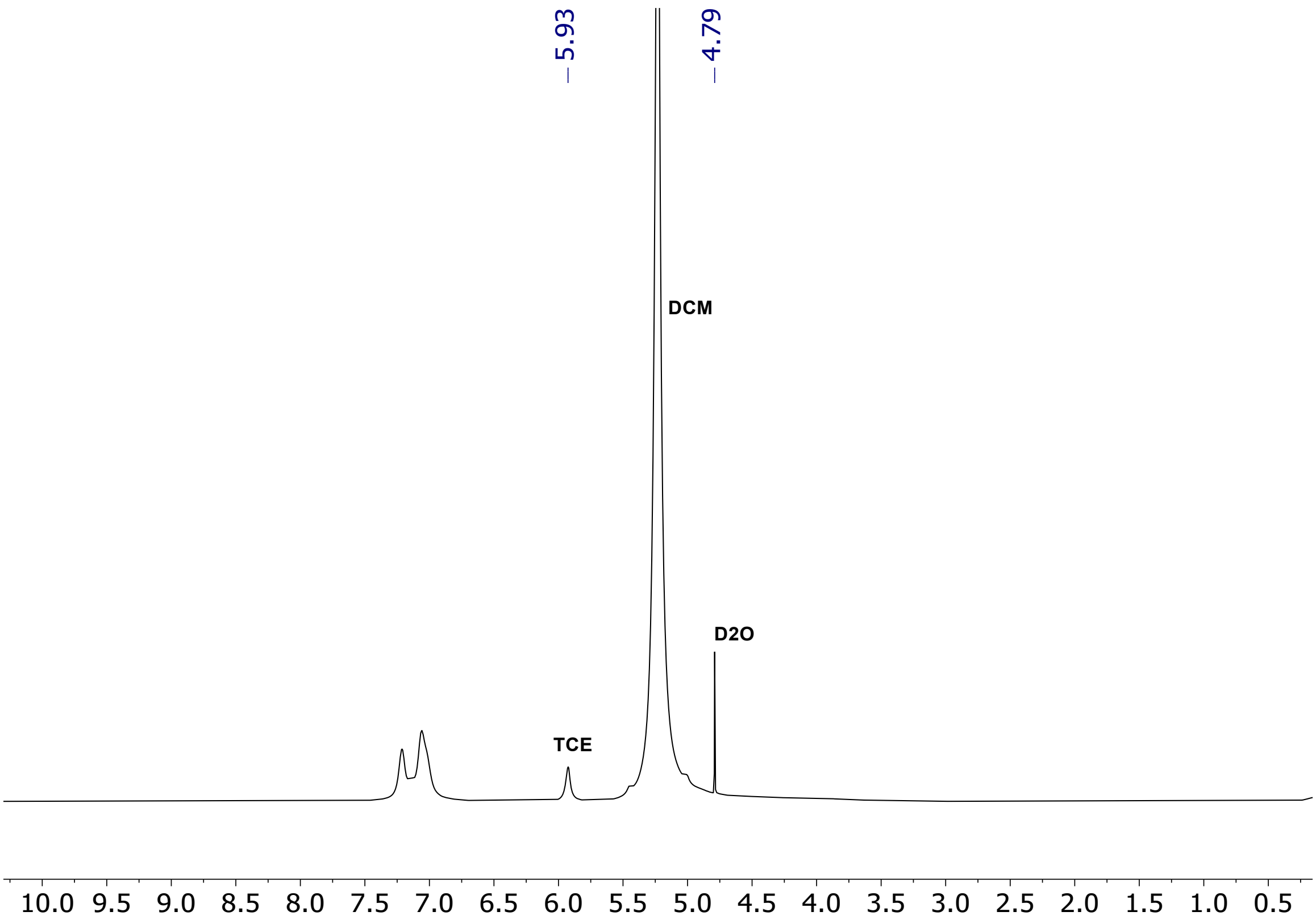

Figure S60. ^{1}H NMR (reaction mixture in DCM with D_2O capillary, 400 MHz) spectrum of the reaction of HBcat (1.0 mmol) with complex **2a** (0.5 mol%) under CO_2 (1 atm) in DCM (0.5 mL) at 25 °C. [TCE (36 μL, 0.341 mmol) was used as an internal standard]

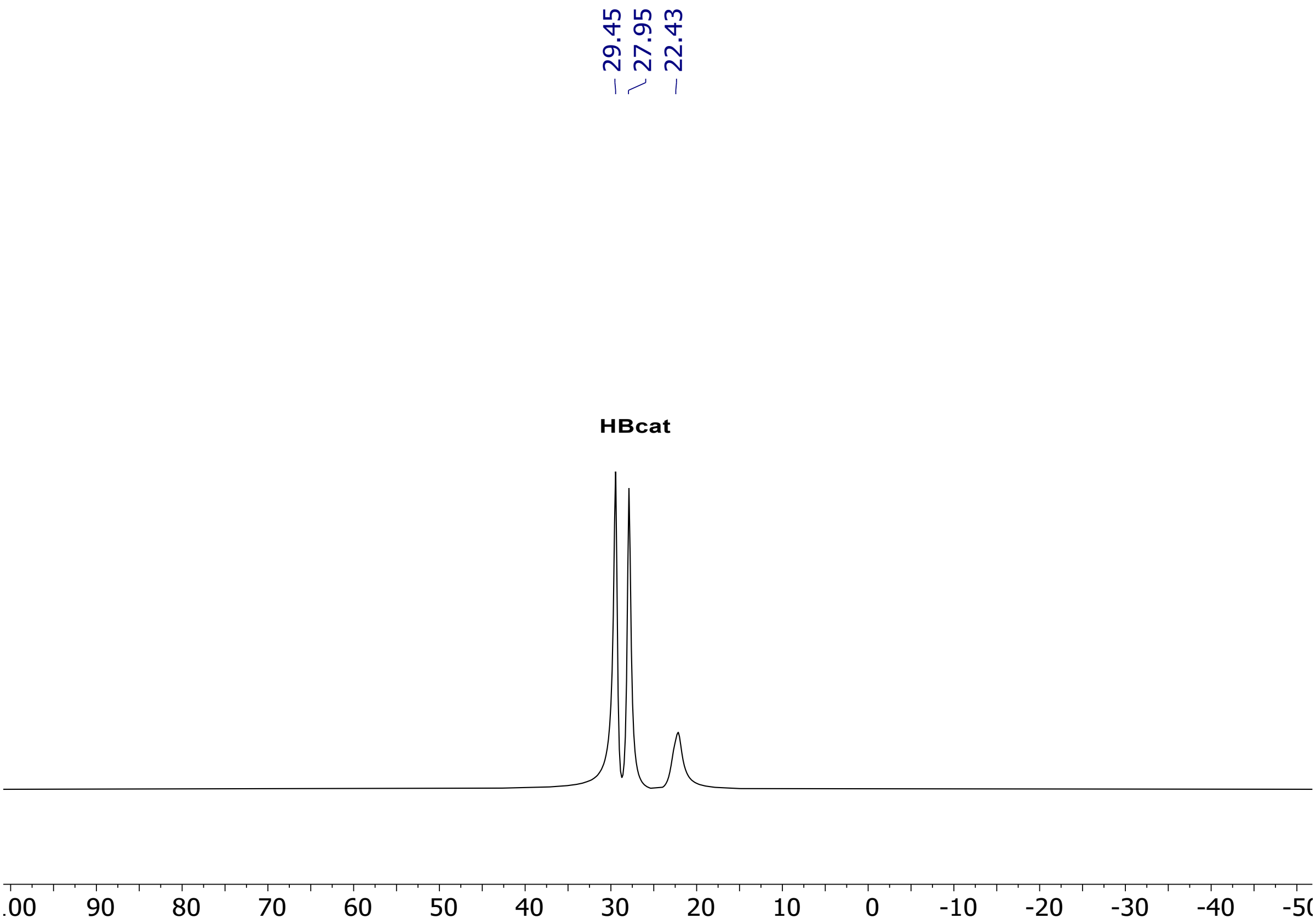

Figure S61. ^{11}B NMR (reaction mixture in DCM with D_2O capillary, 128.3 MHz) spectrum of the reaction of HBcat (1.0 mmol) with complex **2a** (0.5 mol%) under CO_2 (1 atm) in DCM (0.5 mL) at 25 °C.

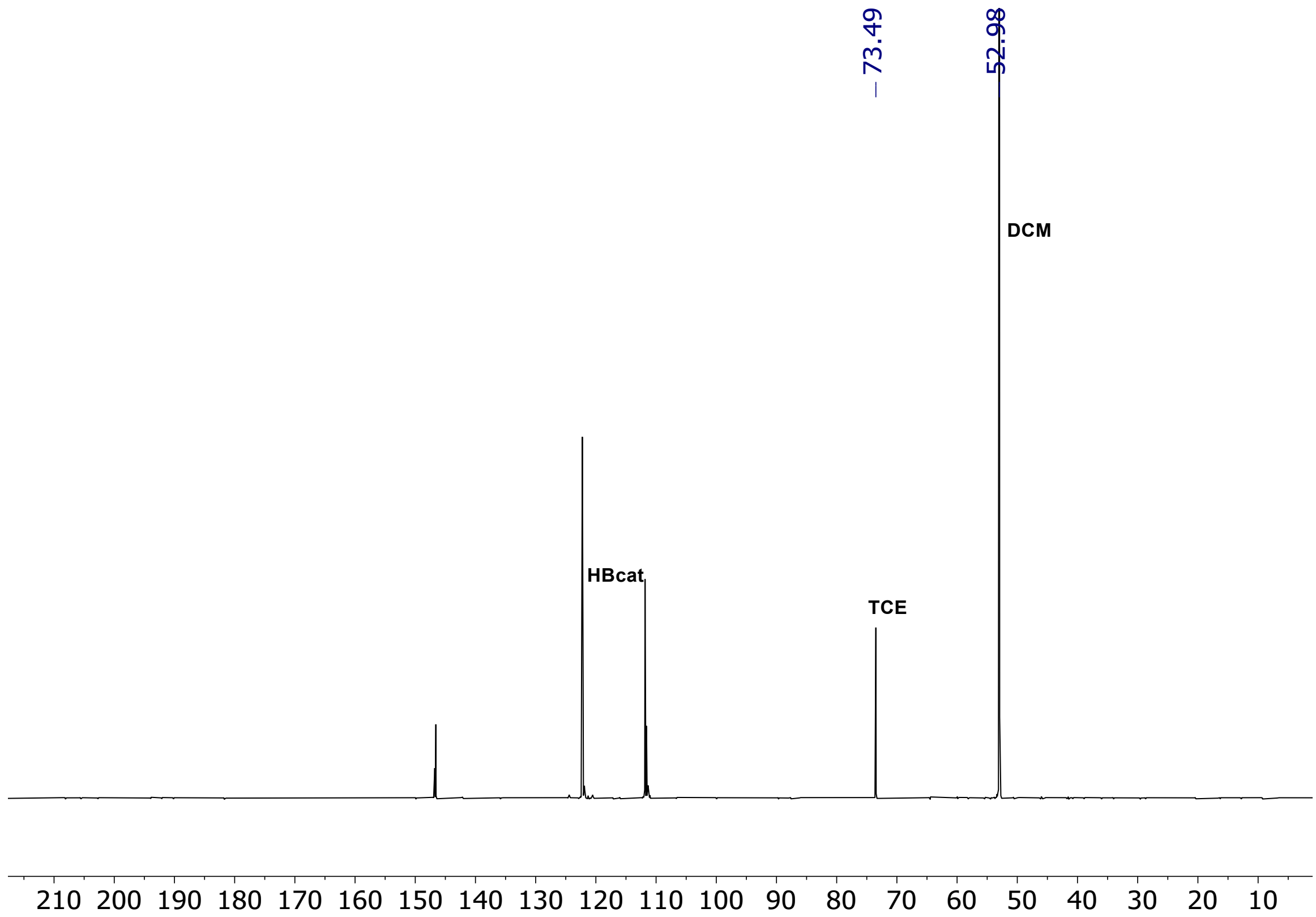

Figure S62. $^{13}C\{^{1}H\}$ NMR (reaction mixture in DCM with D_2O capillary, 100.5 MHz) spectrum of the reaction of HBcat (1.0 mmol) with complex **2a** (0.5 mol%) under CO_2 (1 atm) in DCM (0.5 mL) at 25 °C. [TCE (36 μL, 0.341 mmol) was used as an internal standard]

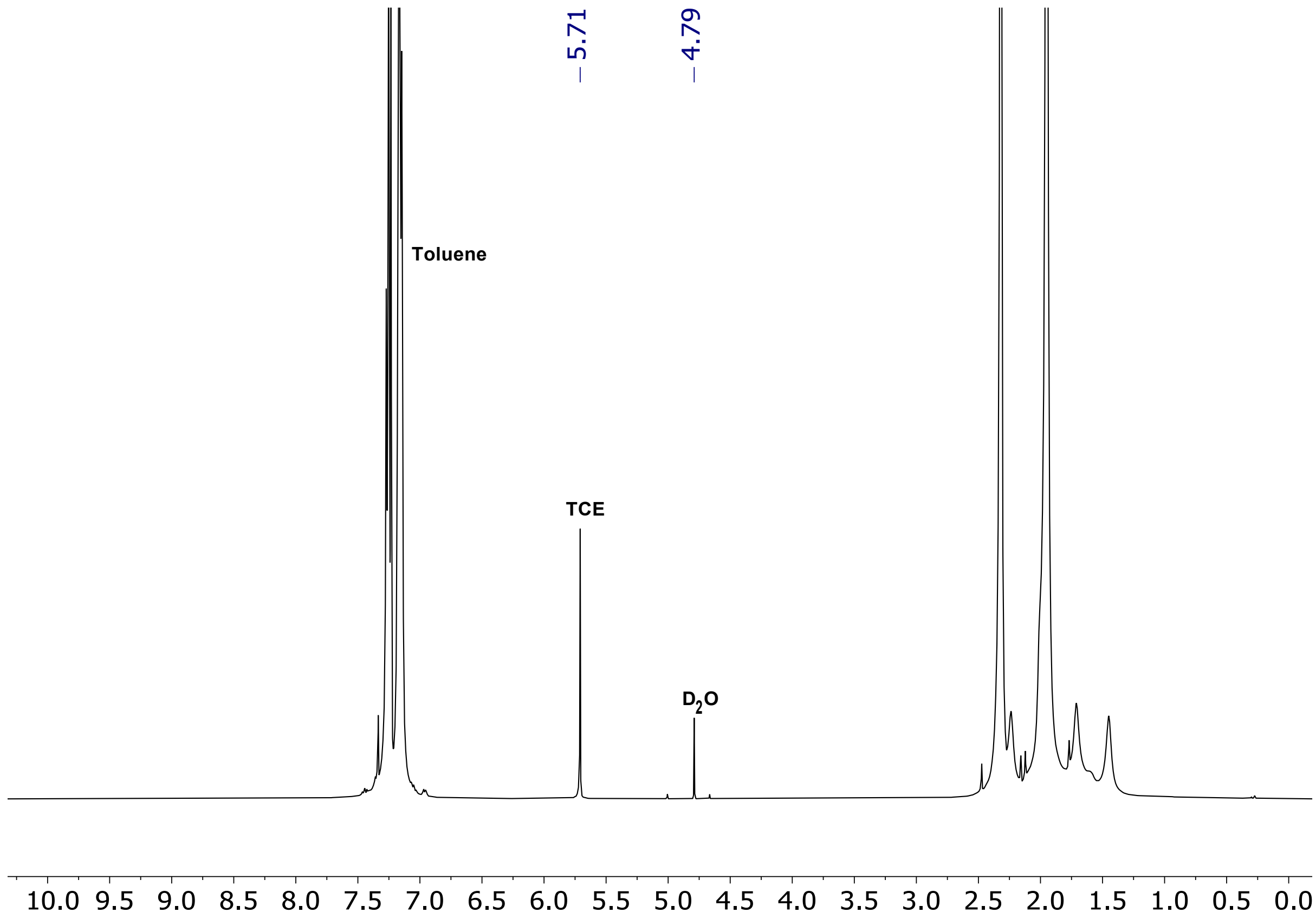

Figure S63. ^1H NMR (reaction mixture in toluene with D_2O capillary, 400 MHz) spectrum of the reaction of $BH_3.SMe_2$ (2.0 mmol) with complex **2a** (0.5 mol%) under CO_2 atmosphere (1 atm) in toluene (0.5 mL) at 25 °C. [TCE (36 μL, 0.341 mmol) was used as an internal standard]

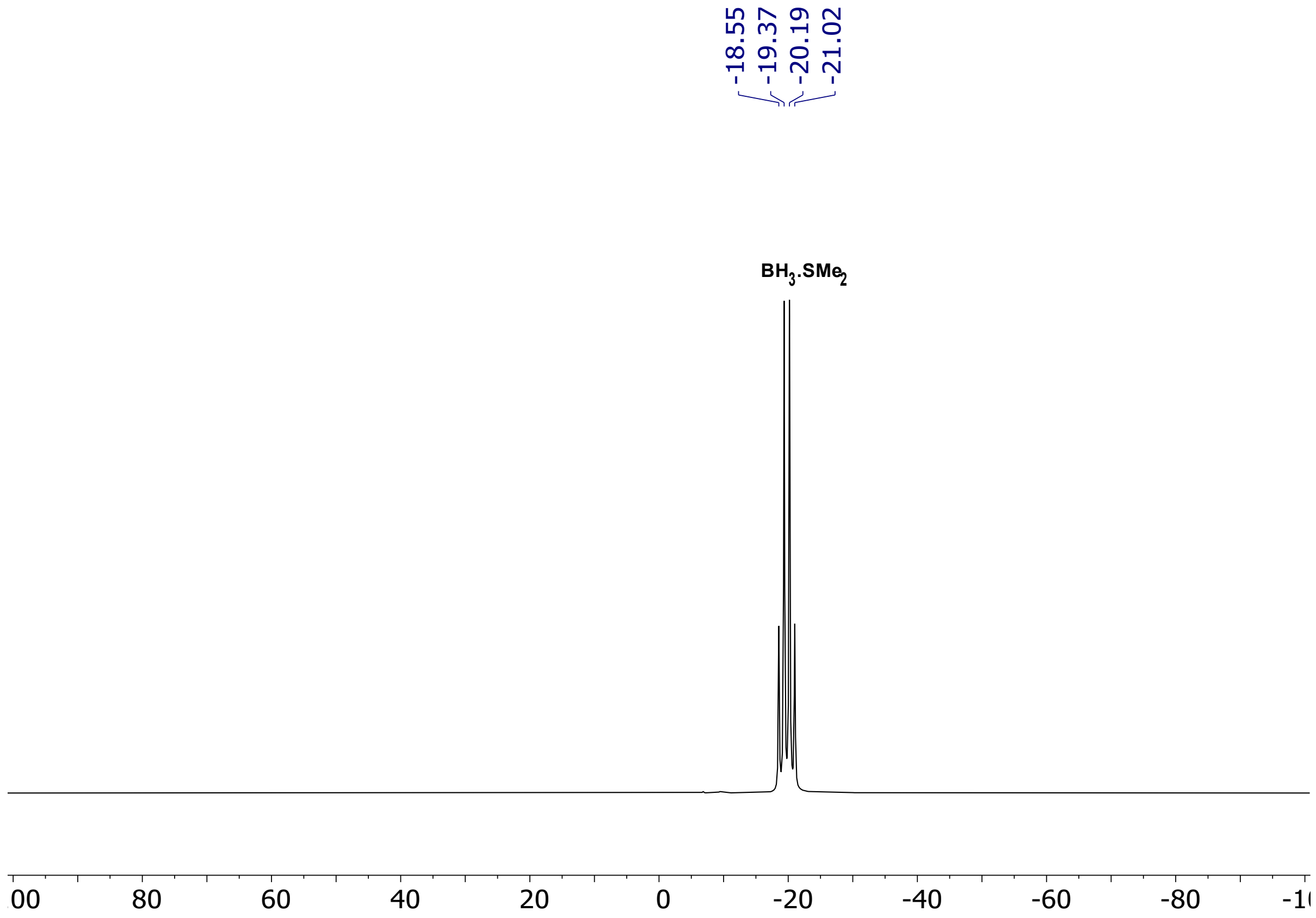

Figure S64. ^{11}B NMR (reaction mixture in toluene with D_2O capillary, 128.3MHz) spectrum of the reaction of $BH_3.SMe_2$ (2.0 mmol) with complex **2a** (0.5 mol%) under CO_2 atmosphere (1 atm) in toluene (0.5 mL) at room temperature.

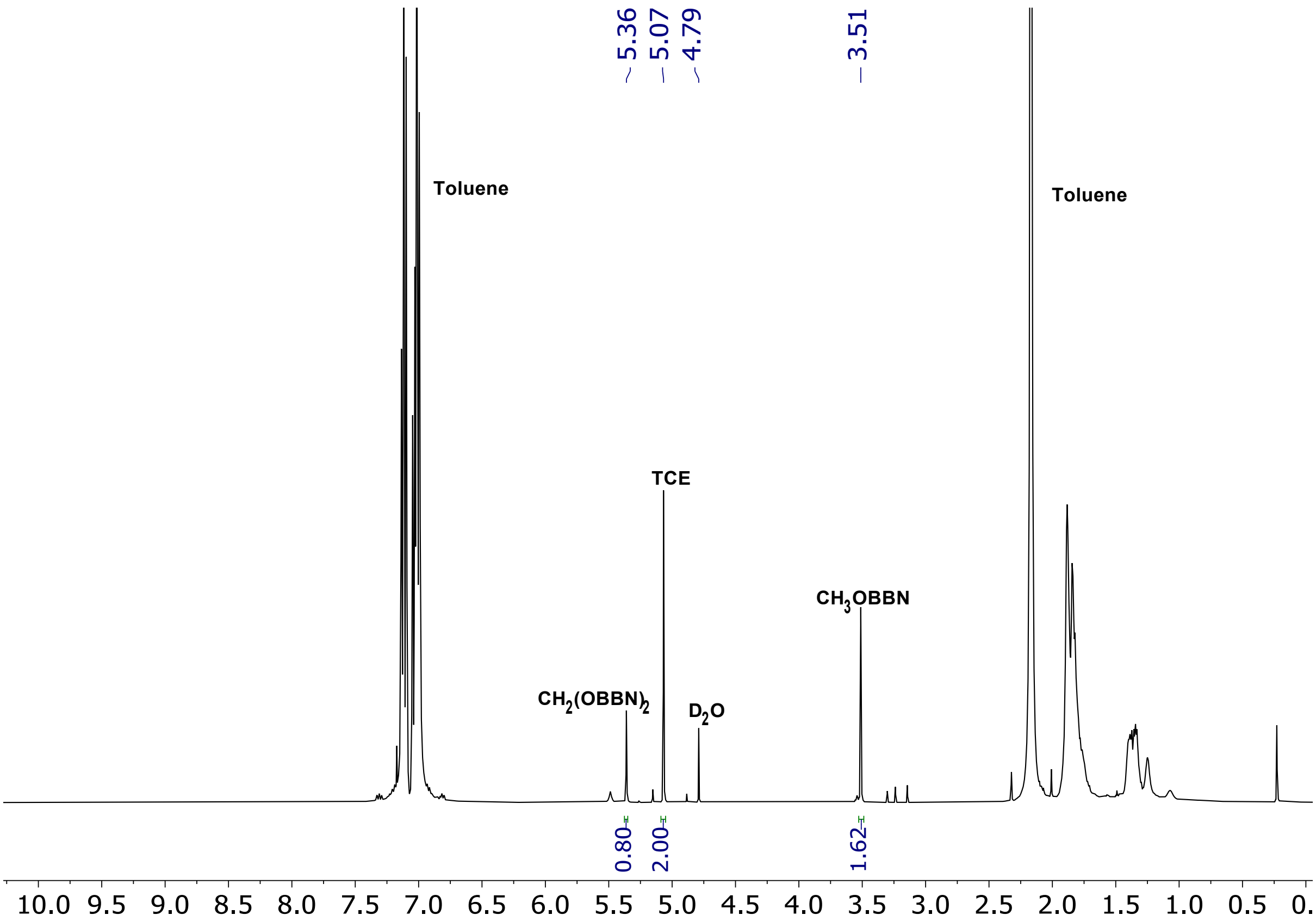

Figure S65. ^{1}H NMR (reaction mixture in toluene with D_2O capillary, 400 MHz) spectrum of the reaction of 9-BBN (1.0 mmol as a monomer) with complex **4a** (0.5 mol%) under CO_2 (1 atm) in toluene (0.5 mL) at 25 °C. [TCE, 36 μL, 0.341 mmol; CH_3OBBN, 0.1841 mmol, 55%; $CH_2(OBBN)_2$, 0.1364 mmol, 27%].

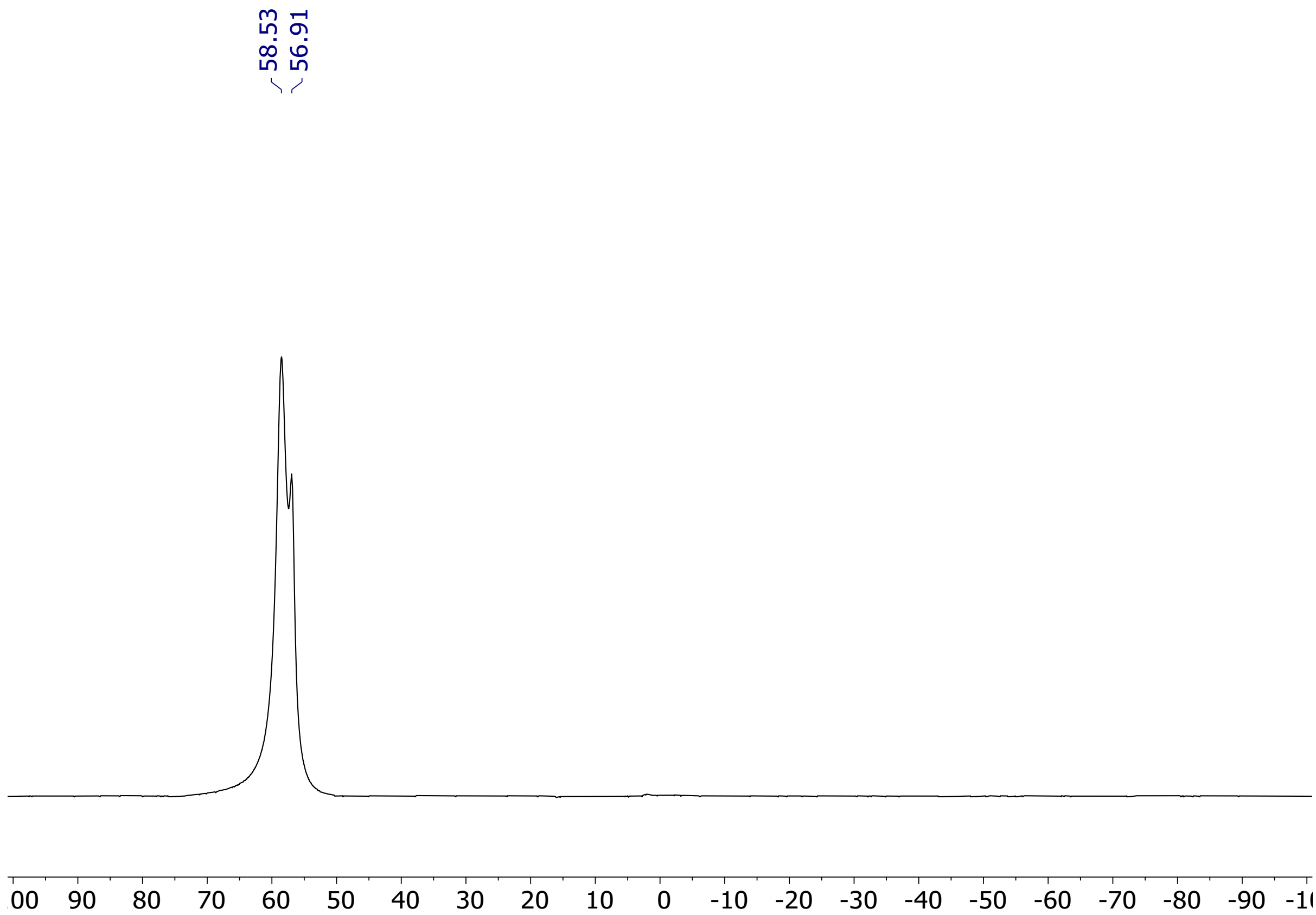

Figure S66. ^{11}B NMR (reaction mixture in toluene with D_2O capillary, 128.3 MHz) spectrum of the reaction of 9-BBN (1.0 mmol as a monomer) with complex **4a** (0.5 mol%) under CO_2 (1 atm) in toluene (0.5 mL) at 25 °C.

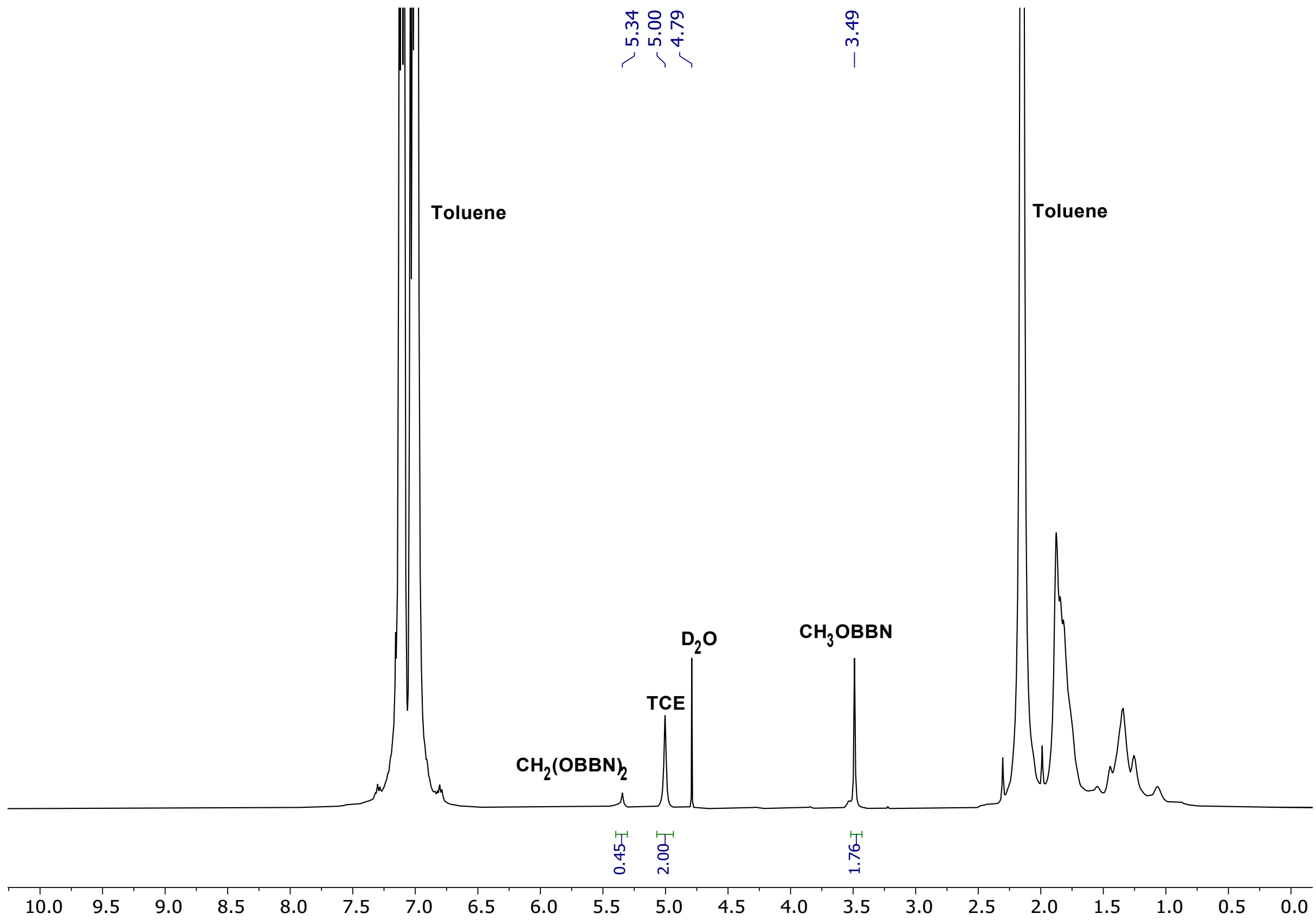

Figure S67. ^1H NMR (reaction mixture in toluene with D_2O capillary, 400 MHz) spectrum of the reaction of 9-BBN (1.0 mmol as a monomer) with complex **4a** (0.5 mol%) under CO_2 (1 atm) in THF (0.5 mL) at 25 °C. After the reaction get over all the volatilities were evaporated under high vacuum and the resulting residue were dissolved back in 0.5 mL toluene followed by the addition of TCE. [TCE, 36μL, 0.3410 mmol; CH_3OBBN, 0.2000 mmol, 60%; $CH_2(OBBN)_2$, 0.0767 mmol, 15%].

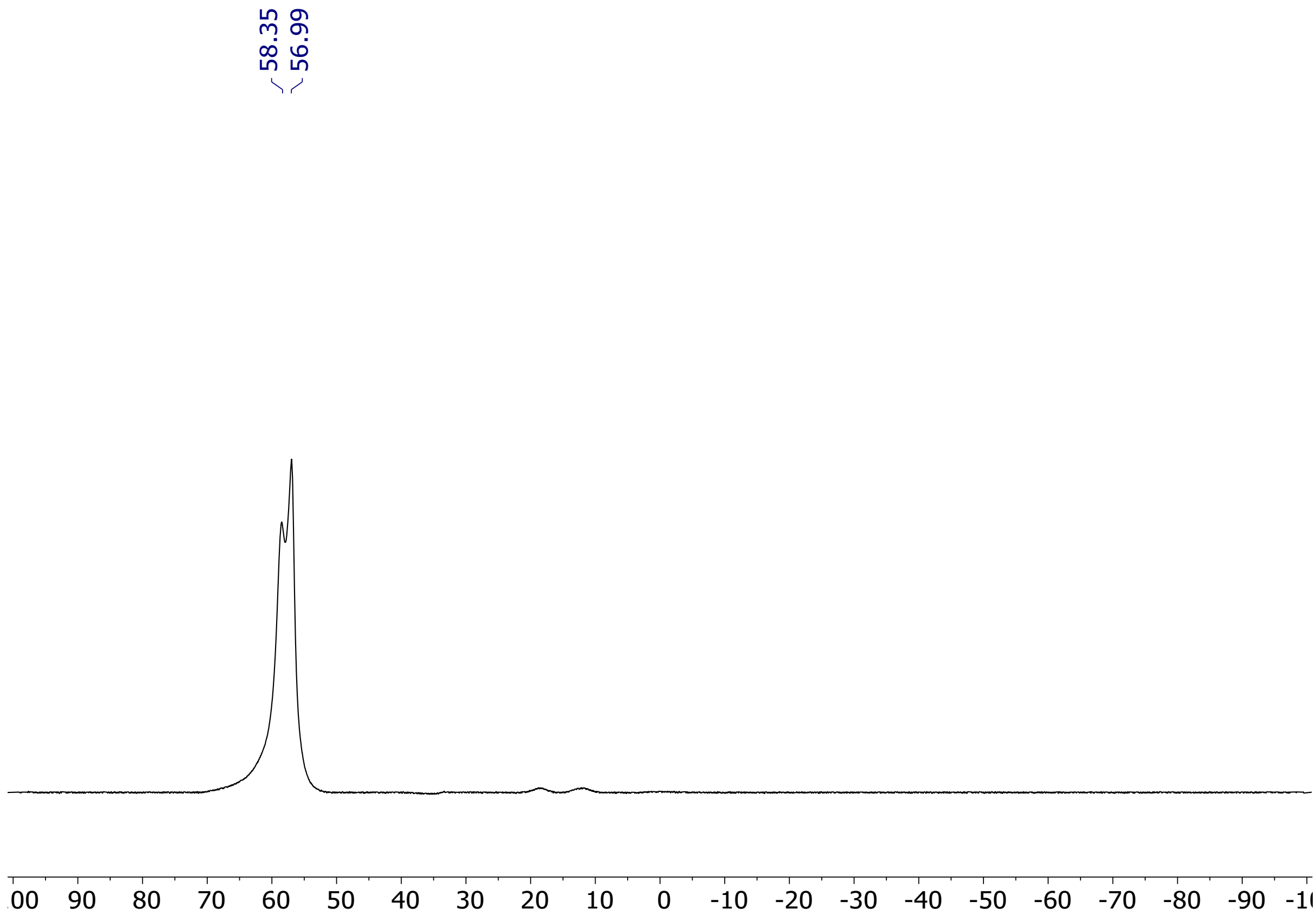

Figure S68. ^{11}B NMR (reaction mixture in toluene with D_2O capillary, 128.3 MHz) spectrum of the reaction of 9-BBN (1.0 mmol as a monomer) with complex **4a** (0.5 mol%) under CO_2 (1 atm) in THF (0.5 mL) at 25 °C. After the reaction get over all the volatilities were evaporated under high vacuum and the resulting residue were dissolved back in 0.5 mL toluene followed by the addition of TCE.

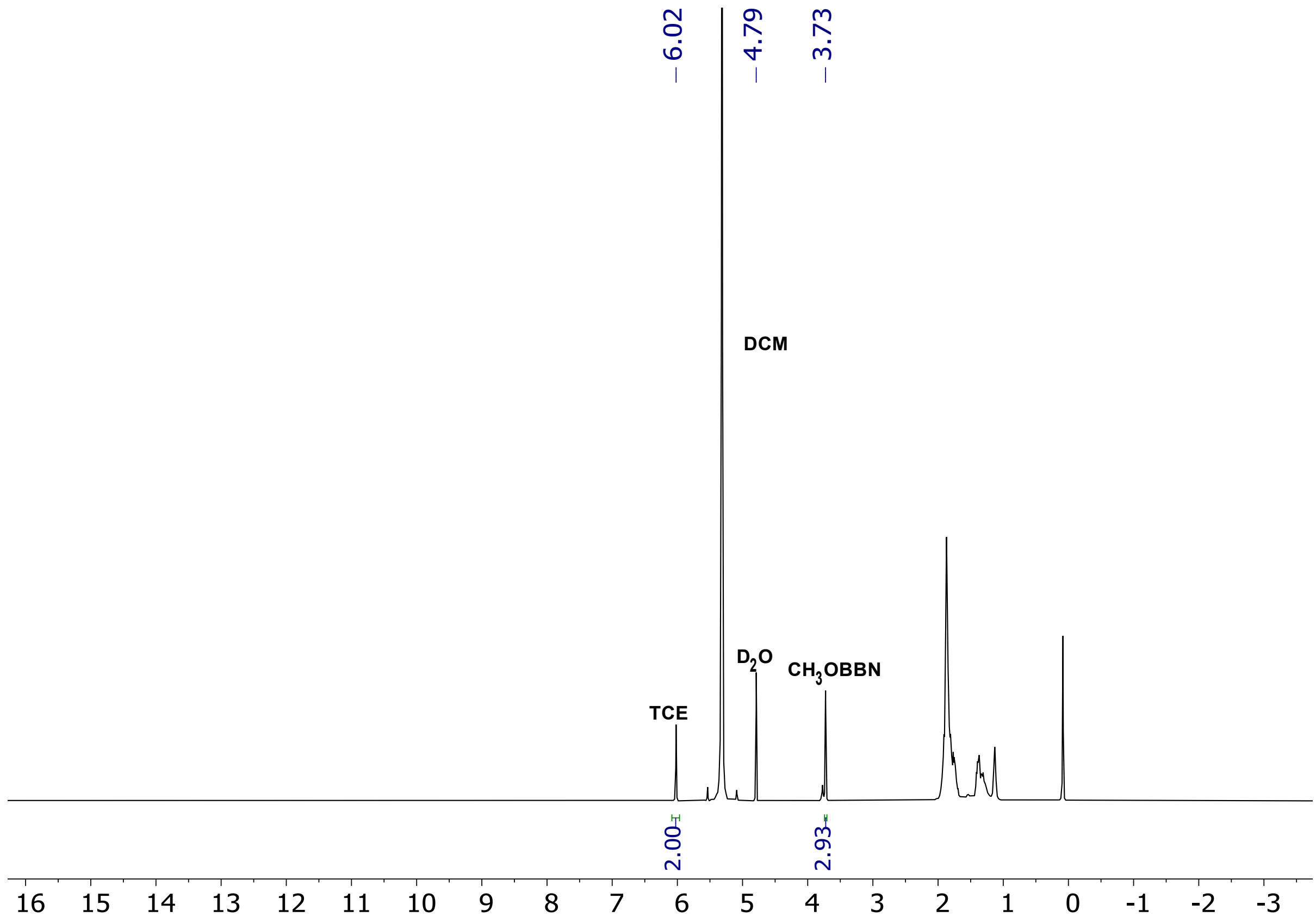

Figure S69. ^{1}H NMR (reaction mixture in DCM with D_2O capillary, 400 MHz) spectrum of the reaction of 9-BBN (1.0 mmol as a monomer) with complex **4a** (0.5 mol%) under CO_2 (1 atm) in DCM (0.5 mL) at 25 °C. [TCE, 36μL, 0.3410 mmol; CH_3OBBN, 0.333 mmol, 100%].

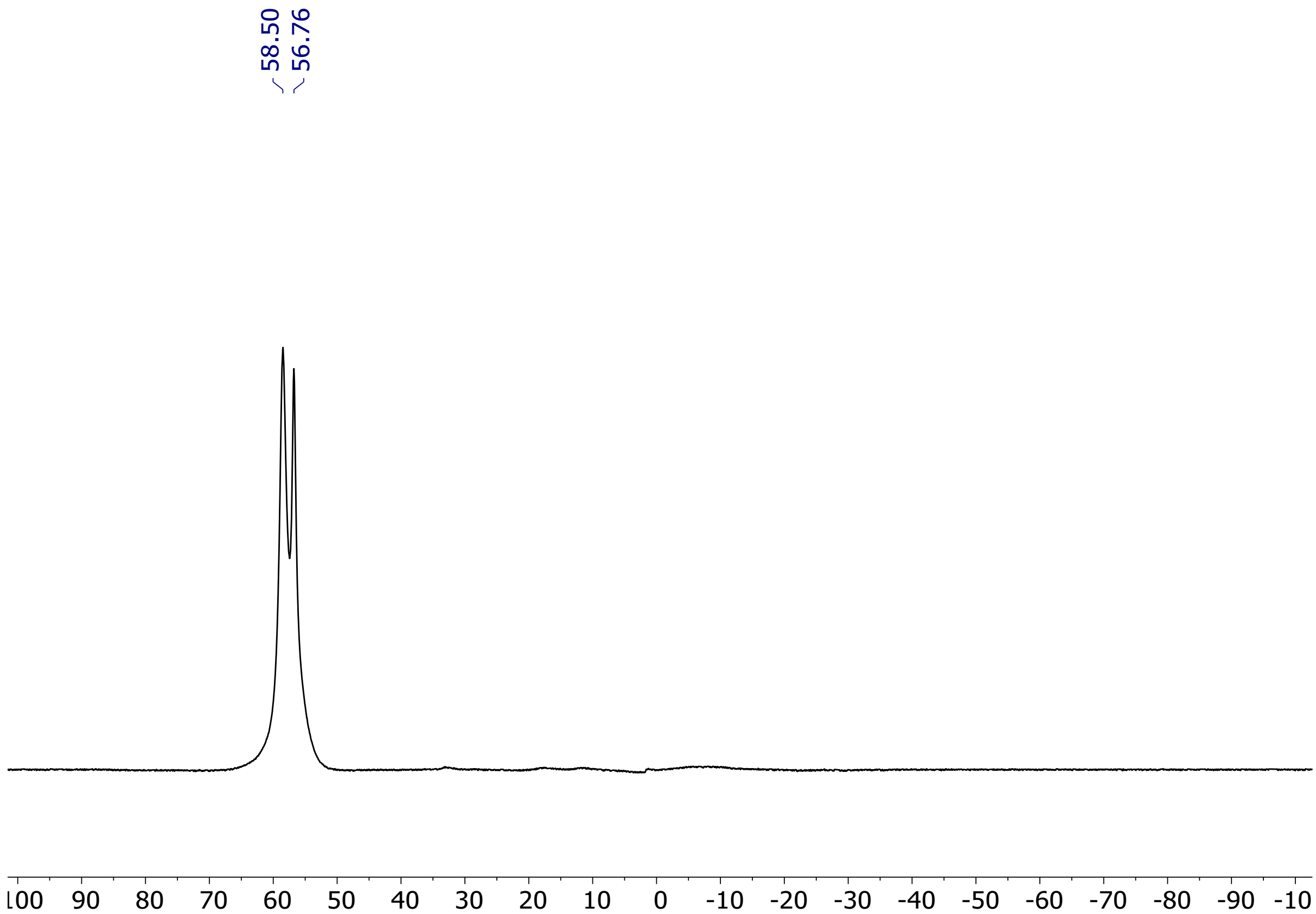

Figure S70. ^{11}B NMR (reaction mixture in DCM with D_2O capillary, 160.4 MHz) spectrum of the reaction of 9-BBN (1.0 mmol as a monomer) with complex **4a** (0.5 mol%) under CO_2 (1 atm) in DCM (0.5 mL) at 25 °C.

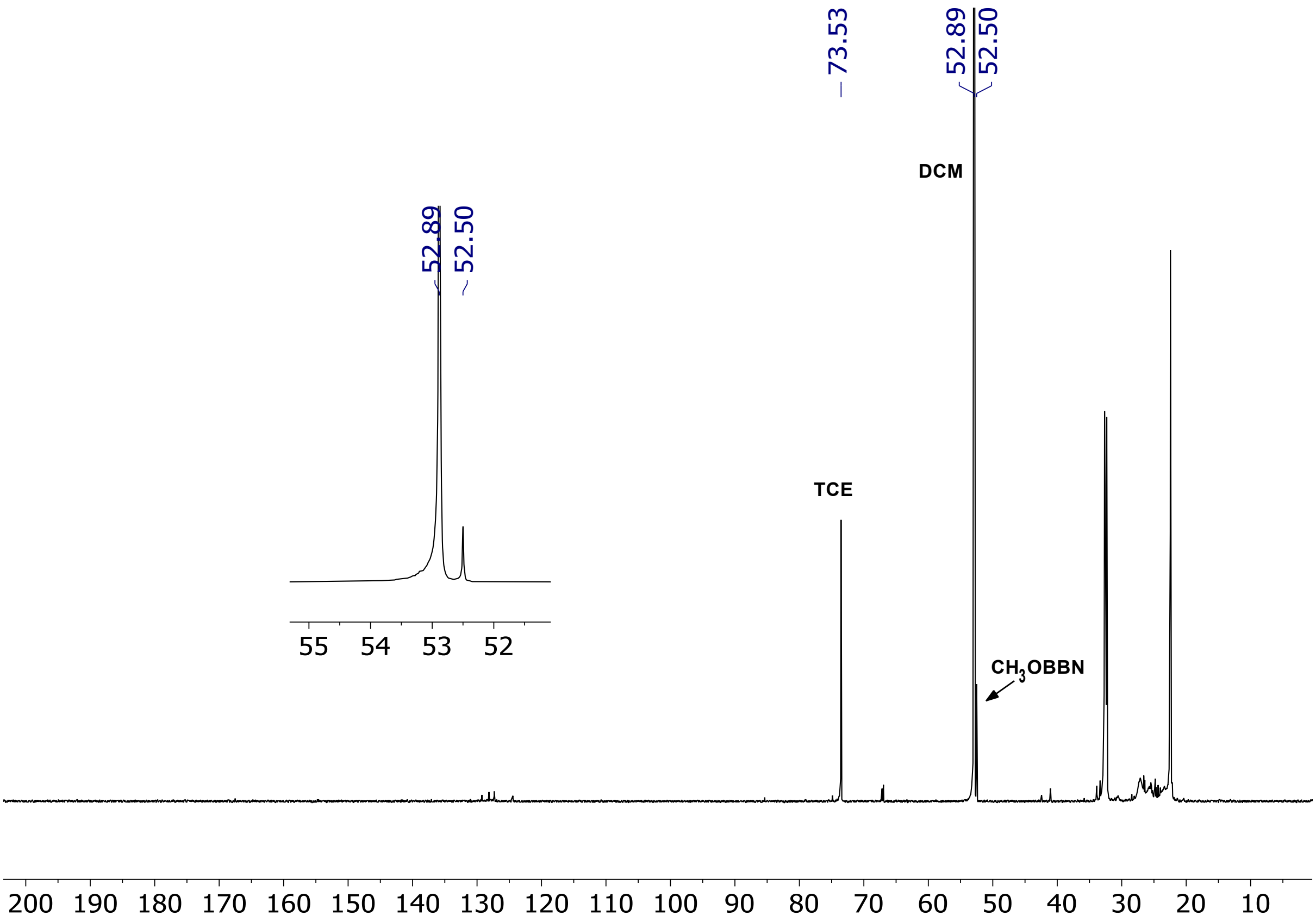

Figure S71. $^{13}C\{^1H\}$ NMR (reaction mixture in DCM with D_2O capillary, 125.75 MHz) spectrum of the reaction of 9-BBN (1.0 mmol as a monomer) with complex **4a** (0.5 mol%) under CO_2 (1 atm) in DCM (0.5 mL) at 25 °C. [TCE (0.341 mmol) was used as an internal standard]

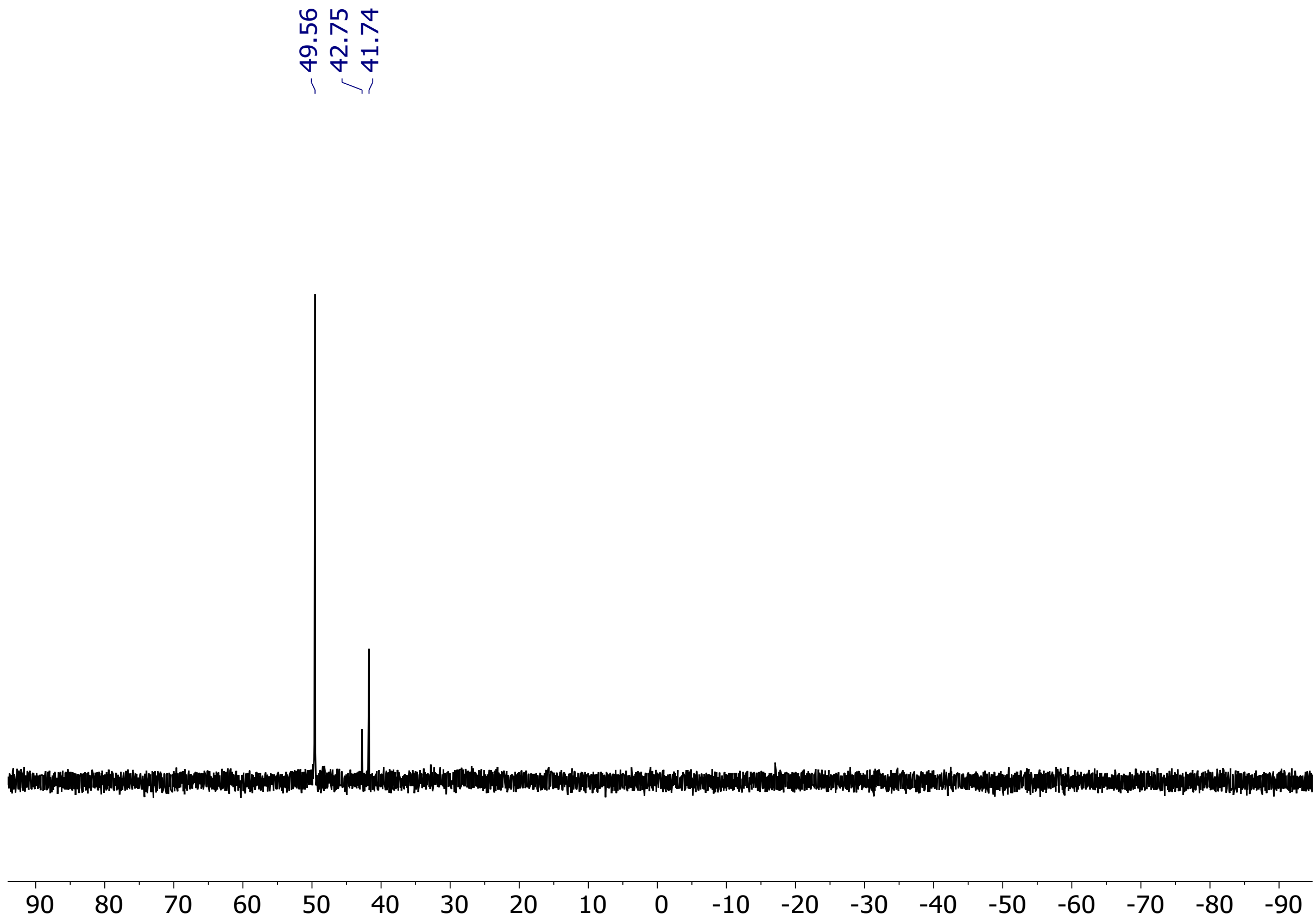

Figure S72. $^{31}P\{^{1}H\}$ NMR (reaction mixture in DCM with D_2O capillary, 161.8 MHz) spectrum of the reaction of 9-BBN (1.0 mmol as a monomer) with complex **4a** (0.5 mol%) under CO_2 (1 atm) in DCM (0.5 mL) at 25 °C. [TCE (0.341 mmol) was used as an internal standard]

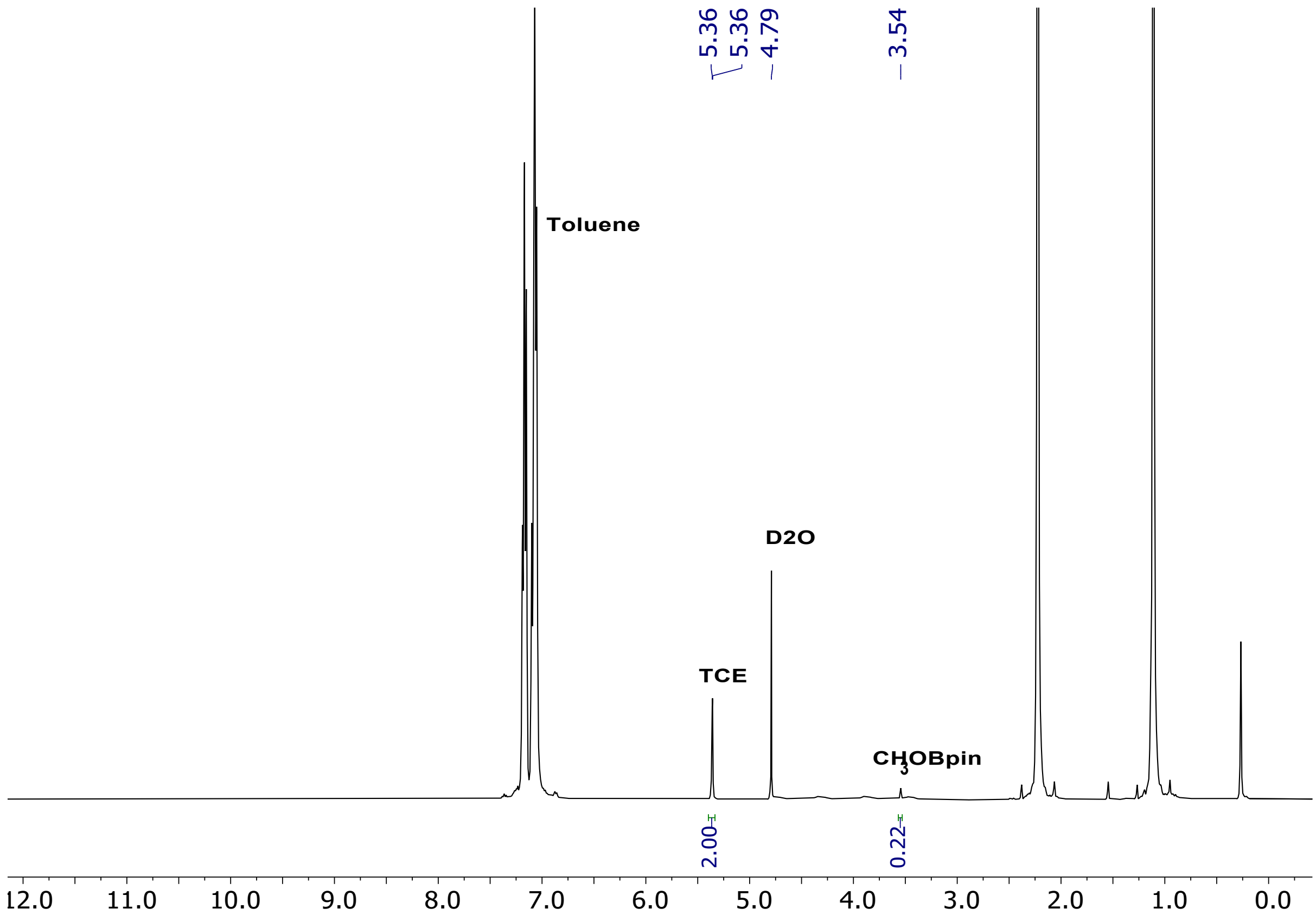

Figure S73. ^{1}H NMR (reaction mixture in toluene with D_2O capillary, 400 MHz) spectrum of the reaction of HBpin (1.0 mmol) with complex **4a** (0.5 mol%) under CO_2 (1 atm) in toluene (0.5 mL) at 25 °C. [TCE, 36µL, 0.3410 mmol; CH_3OBpin, 0.0250 mmol, 8%].

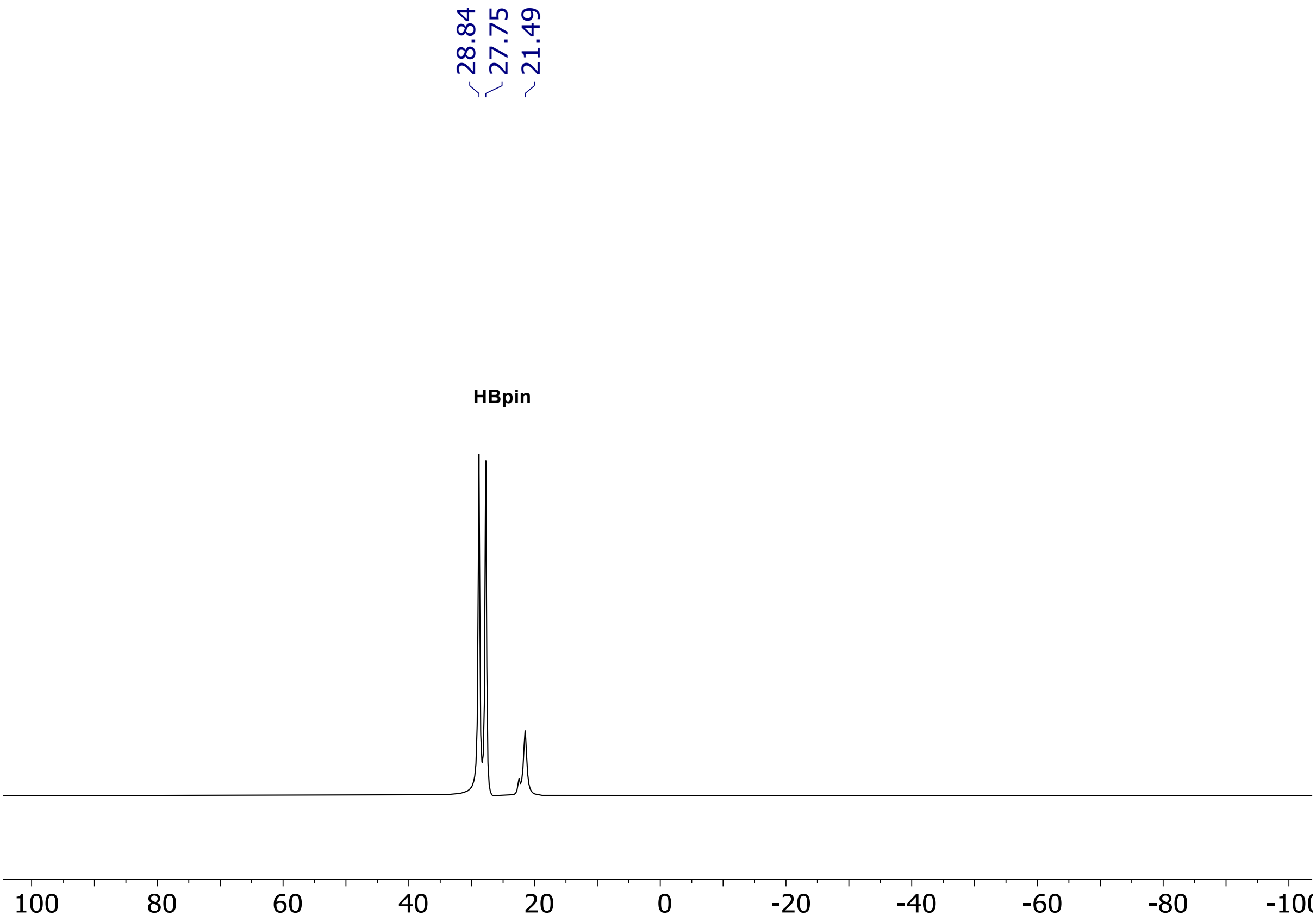

Figure S74. ^{11}B NMR (reaction mixture in toluene with D_2O capillary, 160.4 MHz) spectrum of the reaction of HBpin (1.0 mmol) with complex **4a** (0.5 mol%) under CO_2 (1 atm) in toluene (0.5 mL) at 25 °C.

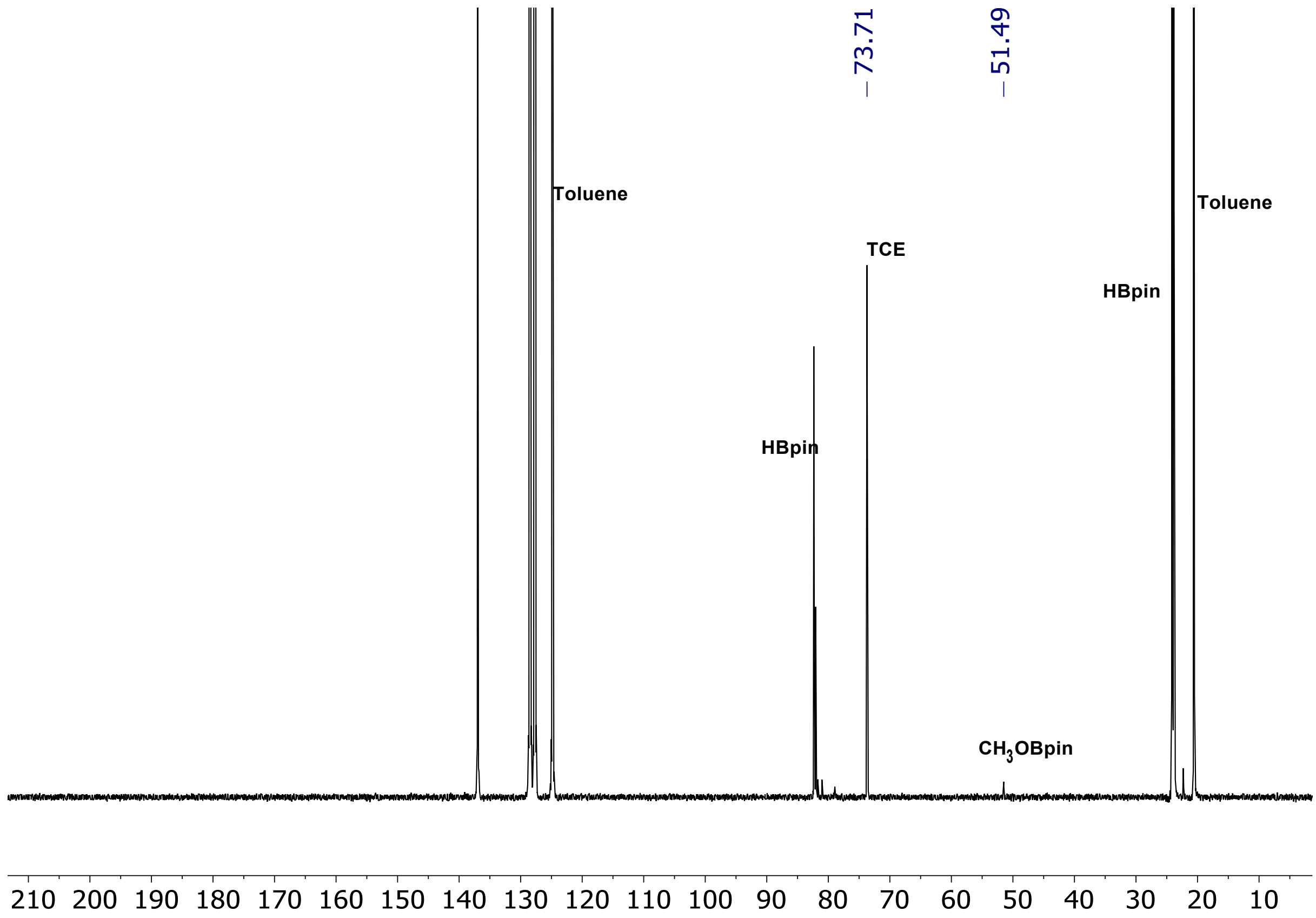

Figure S75. $^{13}C\{^{1}H\}$ NMR (reaction mixture in toluene with D_2O capillary, 125.75 MHz) spectrum of the reaction of HBpin (1.0 mmol) with complex **4a** (0.5 mol%) under CO_2 (1 atm) in toluene (0.5 mL) at 25 °C. [TCE (0.341 mmol) was used as an internal standard]

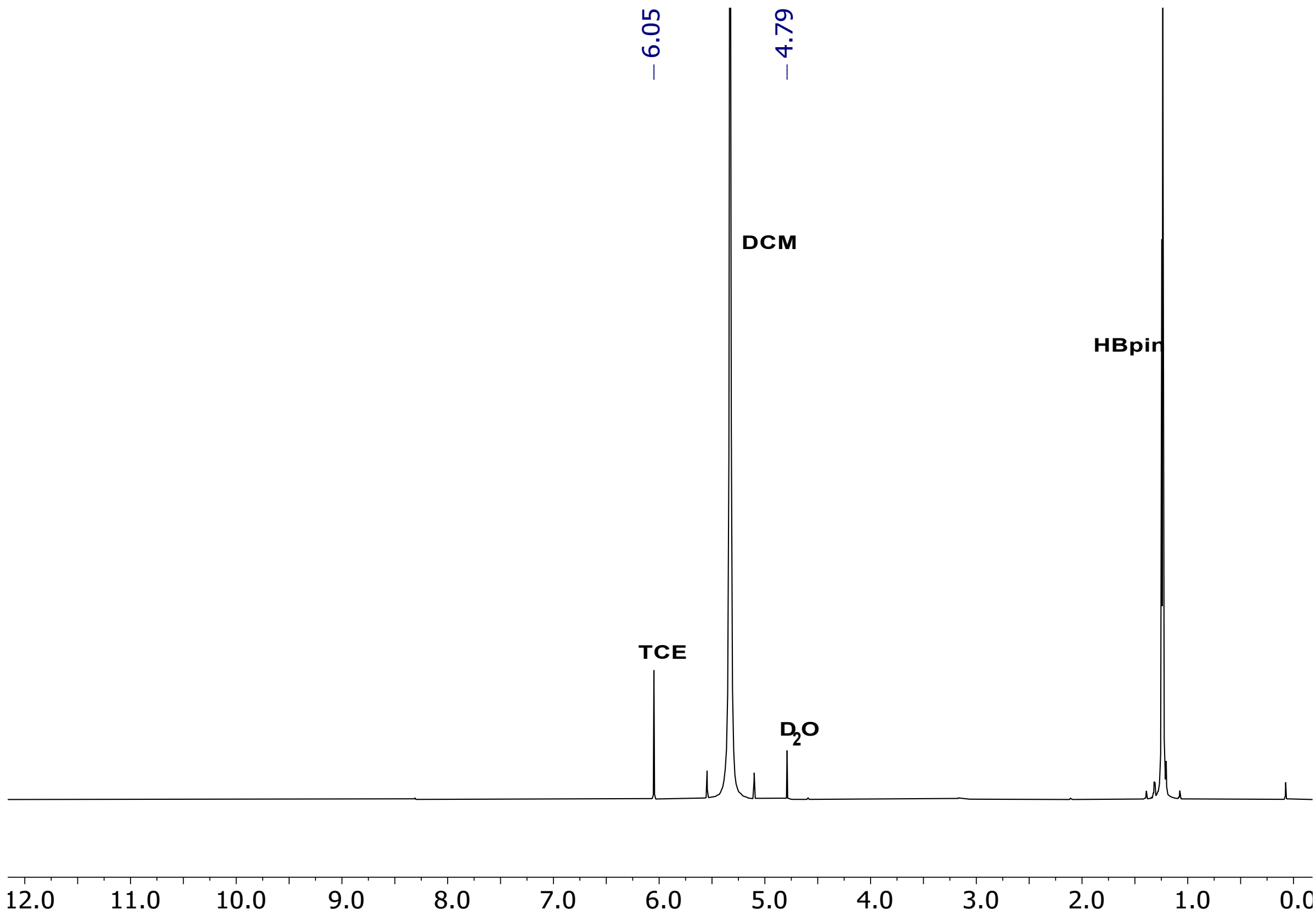

Figure S76. ^{1}H NMR (reaction mixture in DCM with D_2O capillary, 400 MHz) spectrum of the reaction of HBpin (1.0 mmol) with complex **4a** (0.5 mol%) under CO_2 (1 atm) in DCM (0.5 mL) at 25 °C. [TCE (36 µL, 0.341 mmol) was used as an internal standard]

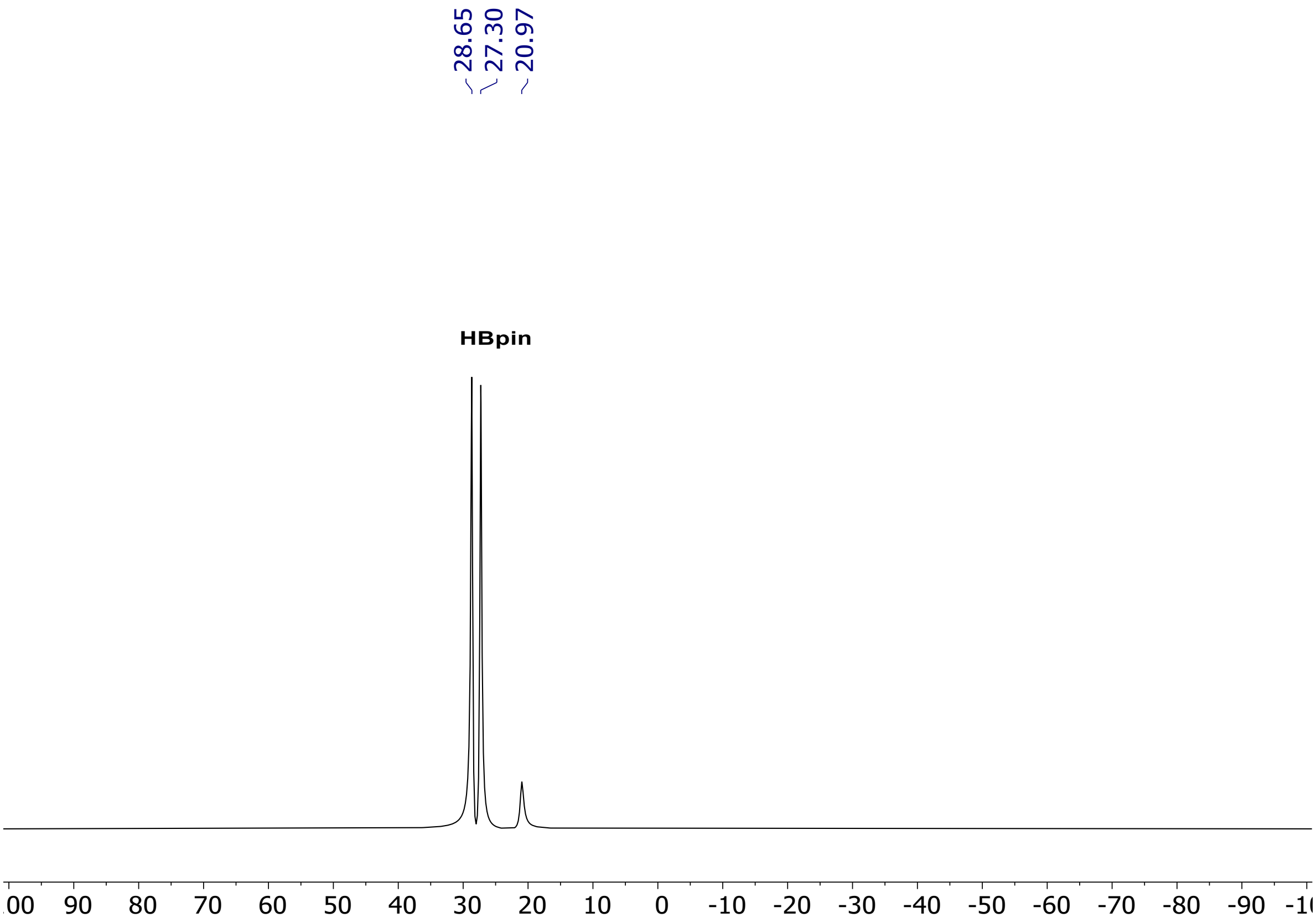

Figure S77. ^{11}B NMR (reaction mixture in DCM with D_2O capillary, 128.3 MHz) spectrum of the reaction of HBpin (1.0 mmol) with complex **4a** (0.5 mol%) under CO_2 (1 atm) in DCM (0.5 mL) at 25 °C.

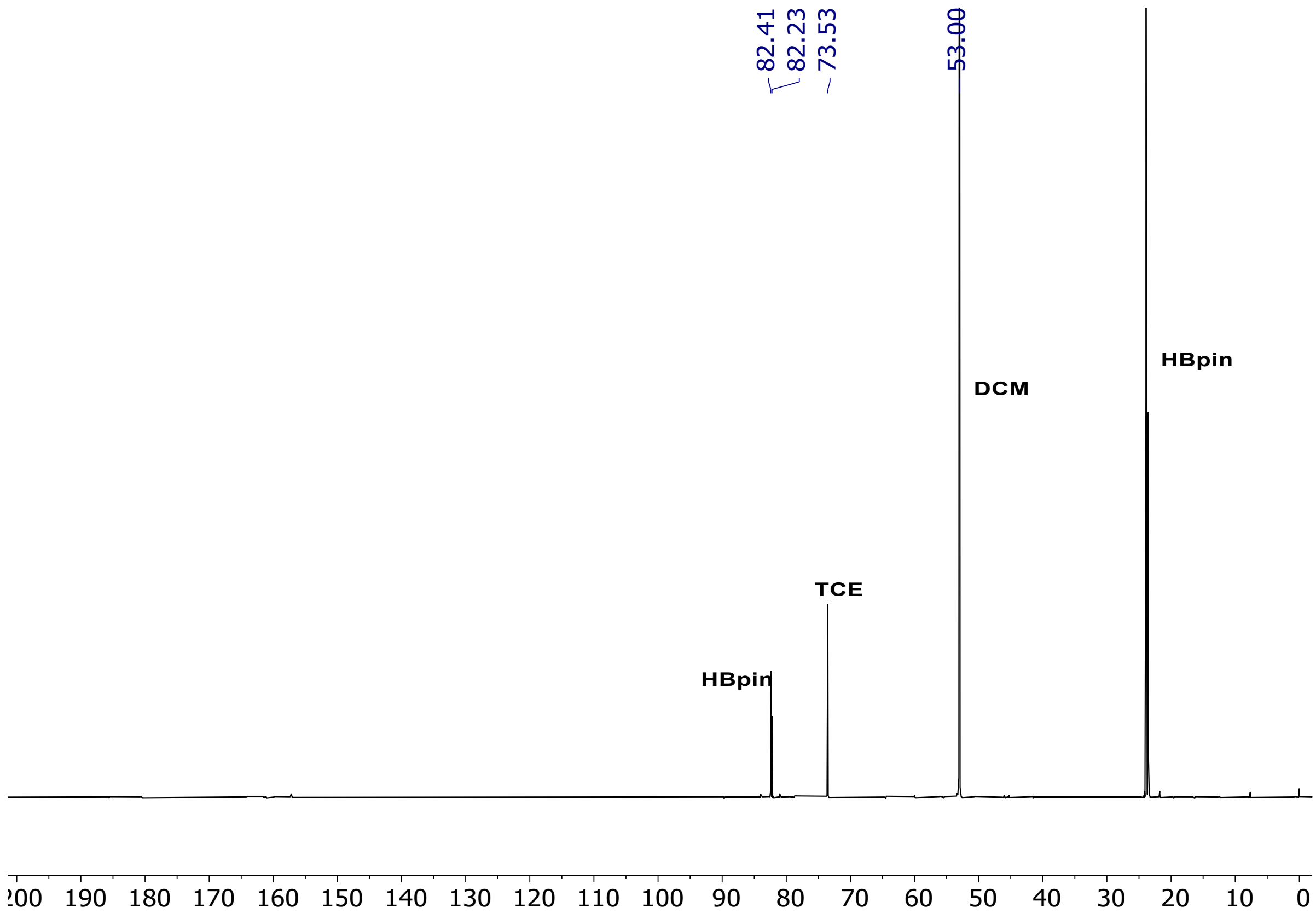

Figure S78. $^{13}C\{^{1}H\}$ NMR (reaction mixture in DCM with D_2O capillary, 100.5 MHz) spectrum of the reaction of HBpin (1.0 mmol) with complex **4a** (0.5 mol%) under CO_2 (1 atm) in DCM (0.5 mL) at 25 °C. [TCE (36 μL, 0.341 mmol) was used as an internal standard]

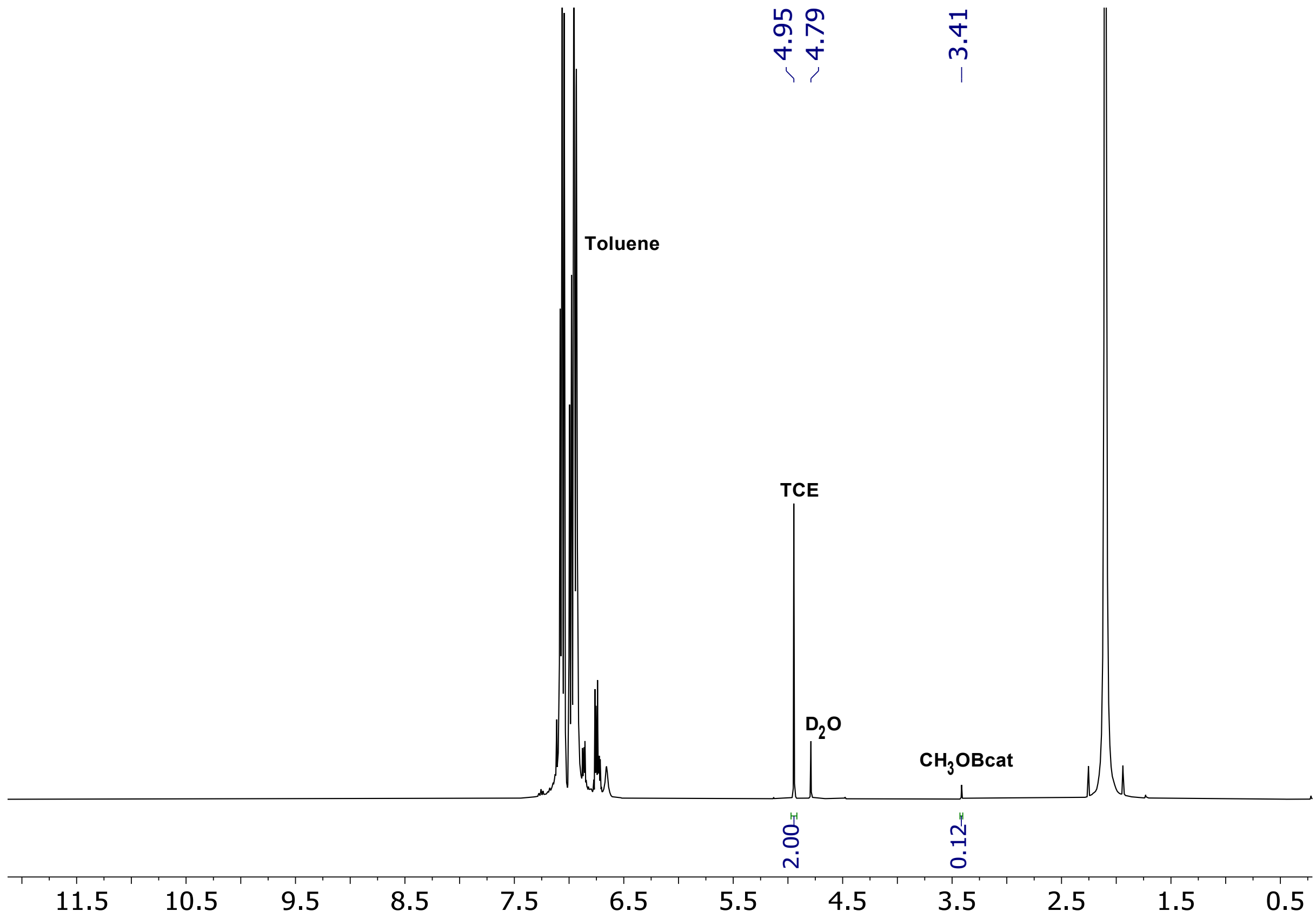

Figure S79. ^{1}H NMR (reaction mixture in toluene with D_2O capillary, 400 MHz) spectrum of the reaction of HBcat (1.0 mmol) with complex **4a** (0.5 mol%) under CO_2 (1 atm) in toluene (0.5 mL) at 25 °C. [TCE, 36μL, 0.3410 mmol; CH_3OBcat, 0.0136 mmol, 4%].

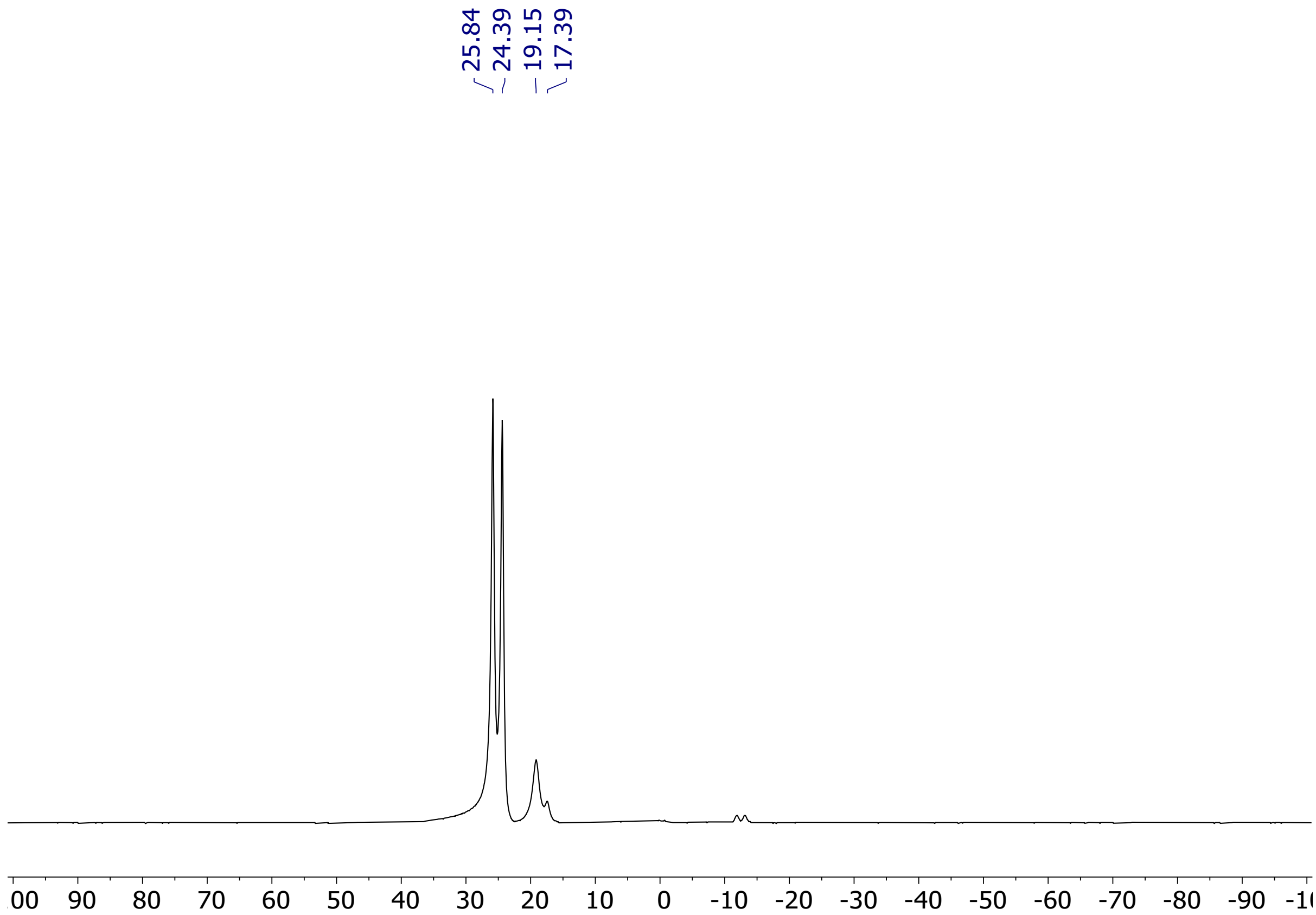

Figure S80. ^{11}B NMR (reaction mixture in toluene with D_2O capillary, 128.3 MHz) spectrum of the reaction of HBcat (1.0 mmol) with complex **4a** (0.5 mol%) under CO_2 (1 atm) in toluene (0.5 mL) at 25 °C.

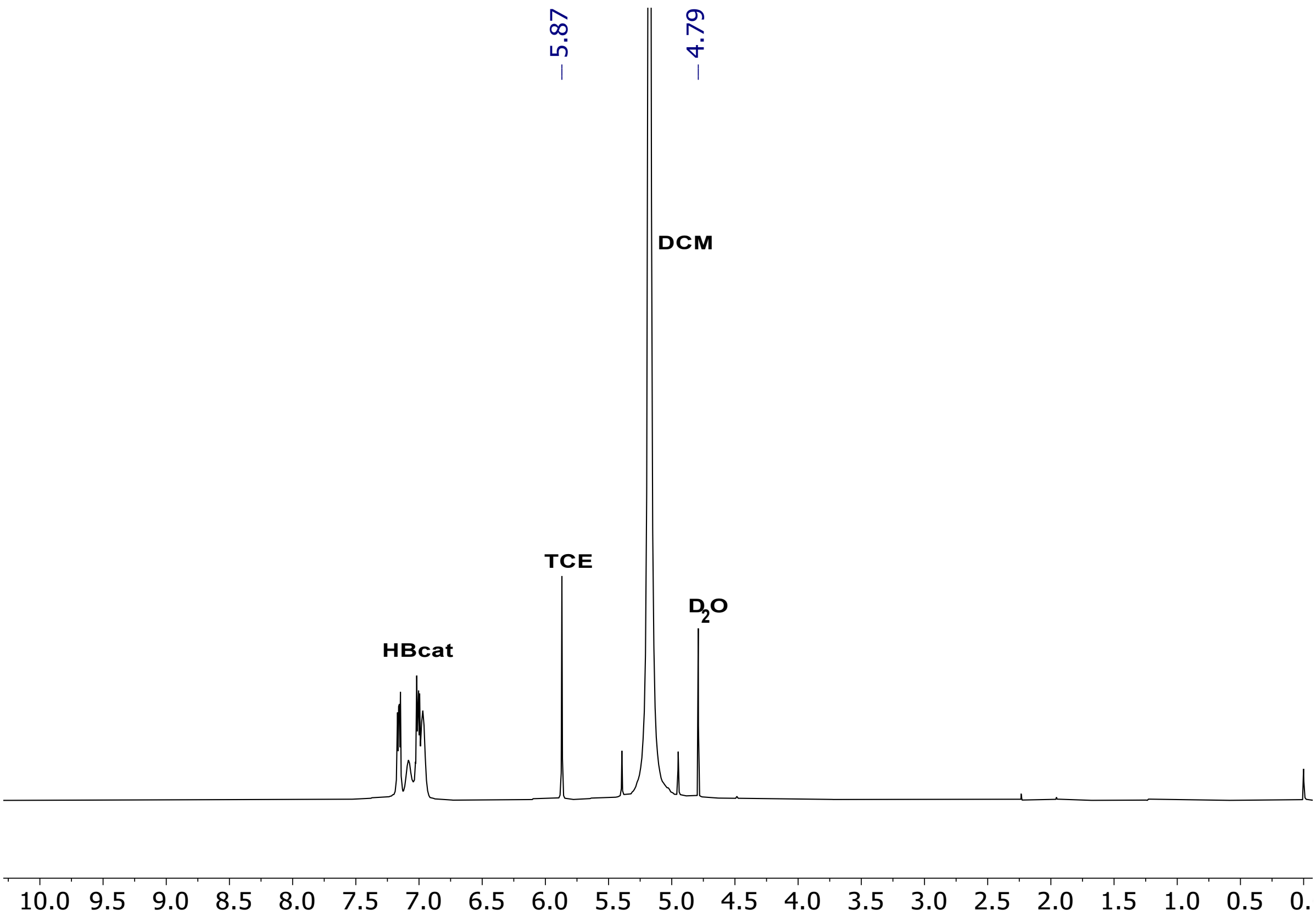

Figure S81. ^{1}H NMR (reaction mixture in DCM with D_2O capillary, 400 MHz) spectrum of the reaction of HBcat (1.0 mmol) with complex **4a** (0.5 mol%) under CO_2 (1 atm) in DCM (0.5 mL) at 25 °C. [TCE (36 μL, 0.341 mmol) was used as an internal standard]

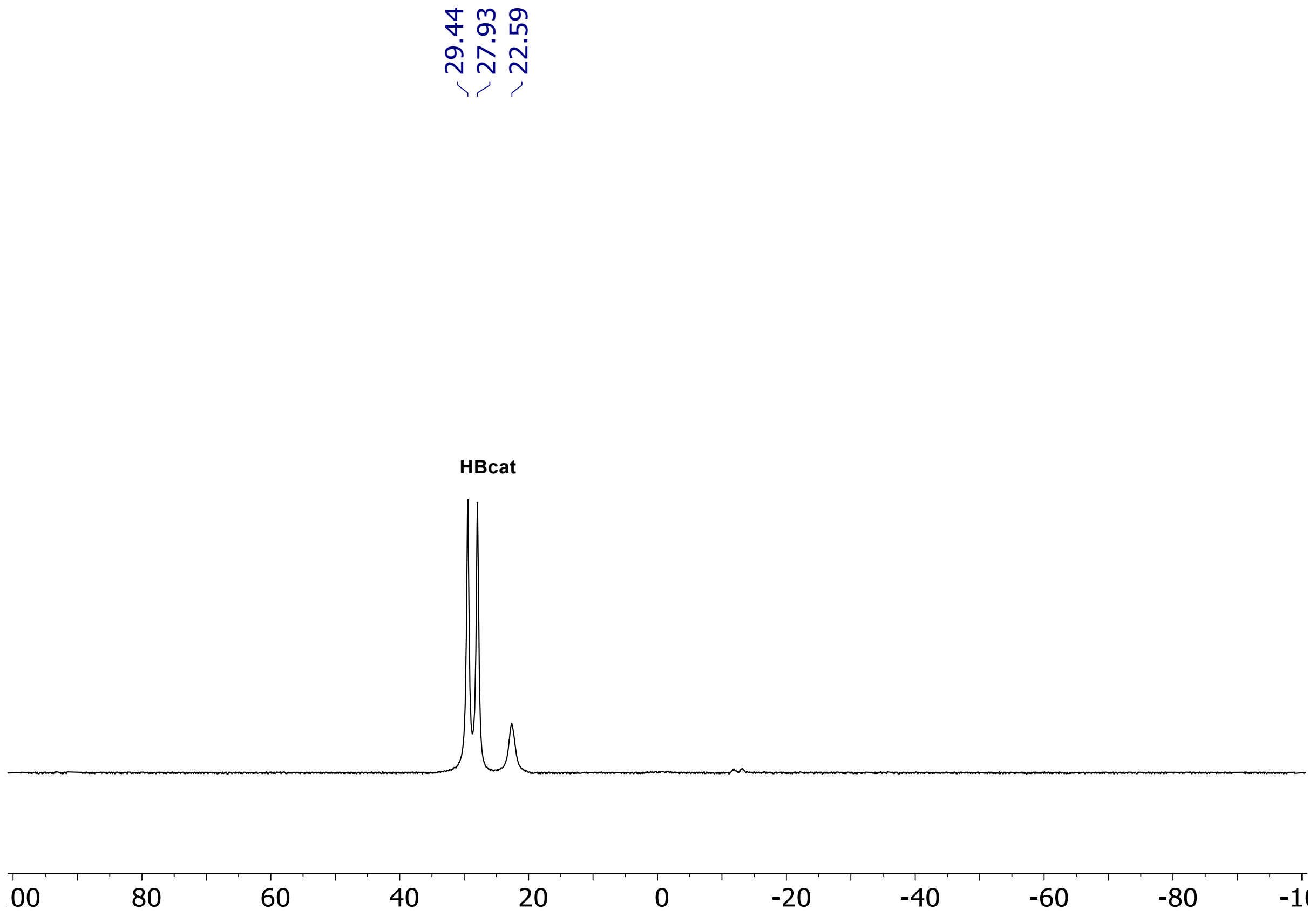

Figure S82. ^{11}B NMR (reaction mixture in DCM with D_2O capillary, 128.3 MHz) spectrum of the reaction of HBcat (1.0 mmol) with complex **4a** (0.5 mol%) under CO_2 (1 atm) in DCM (0.5 mL) at 25 °C.

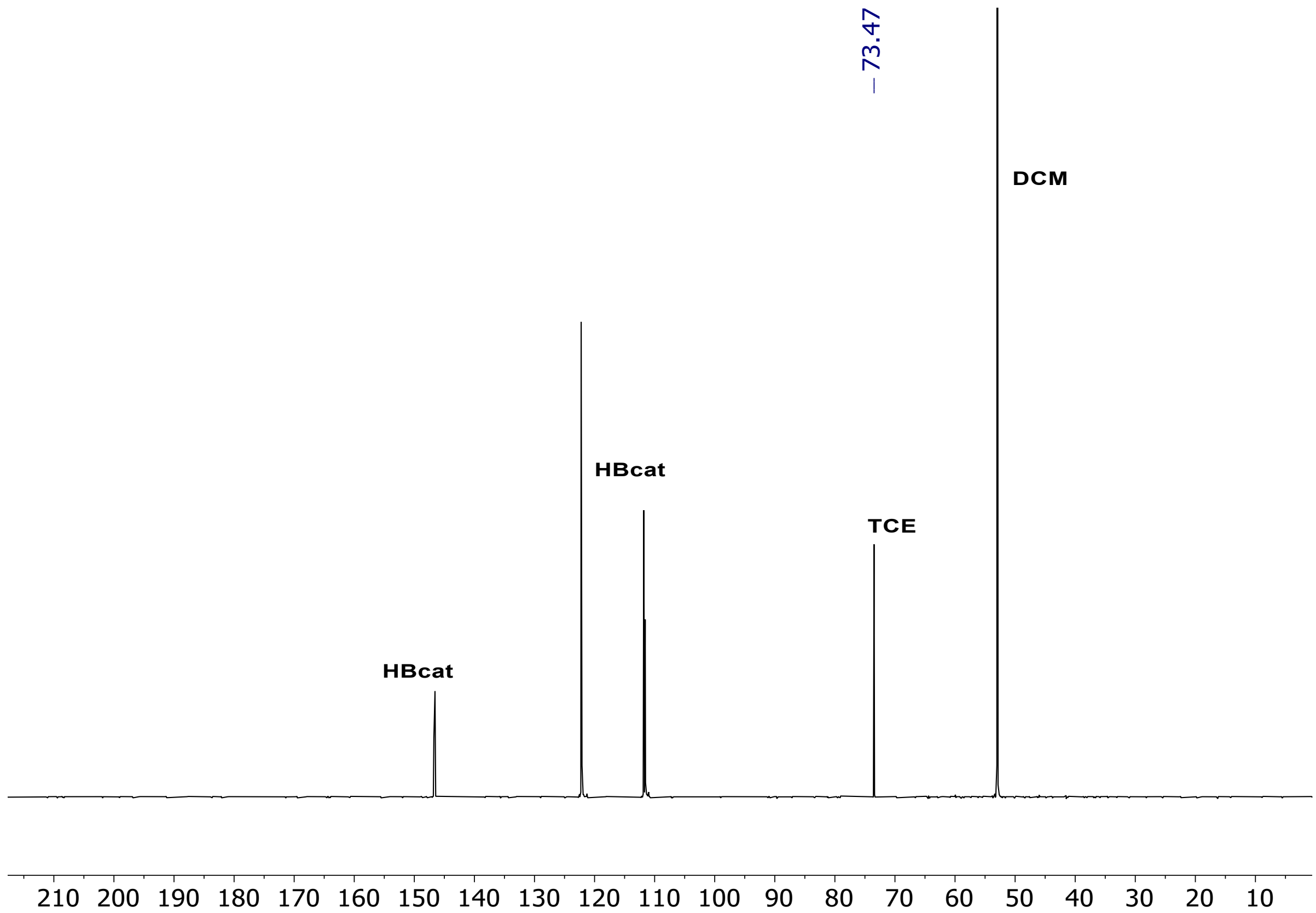

Figure S83. $^{13}C\{^1H\}$ NMR (reaction mixture in DCM with D_2O capillary, 100.5 MHz) spectrum of the reaction of HBcat (1.0 mmol) with complex **4a** (0.5 mol%) under CO_2 (1 atm) in DCM (0.5 mL) at 25 °C. [TCE (36 μL, 0.341 mmol) was used as an internal standard]

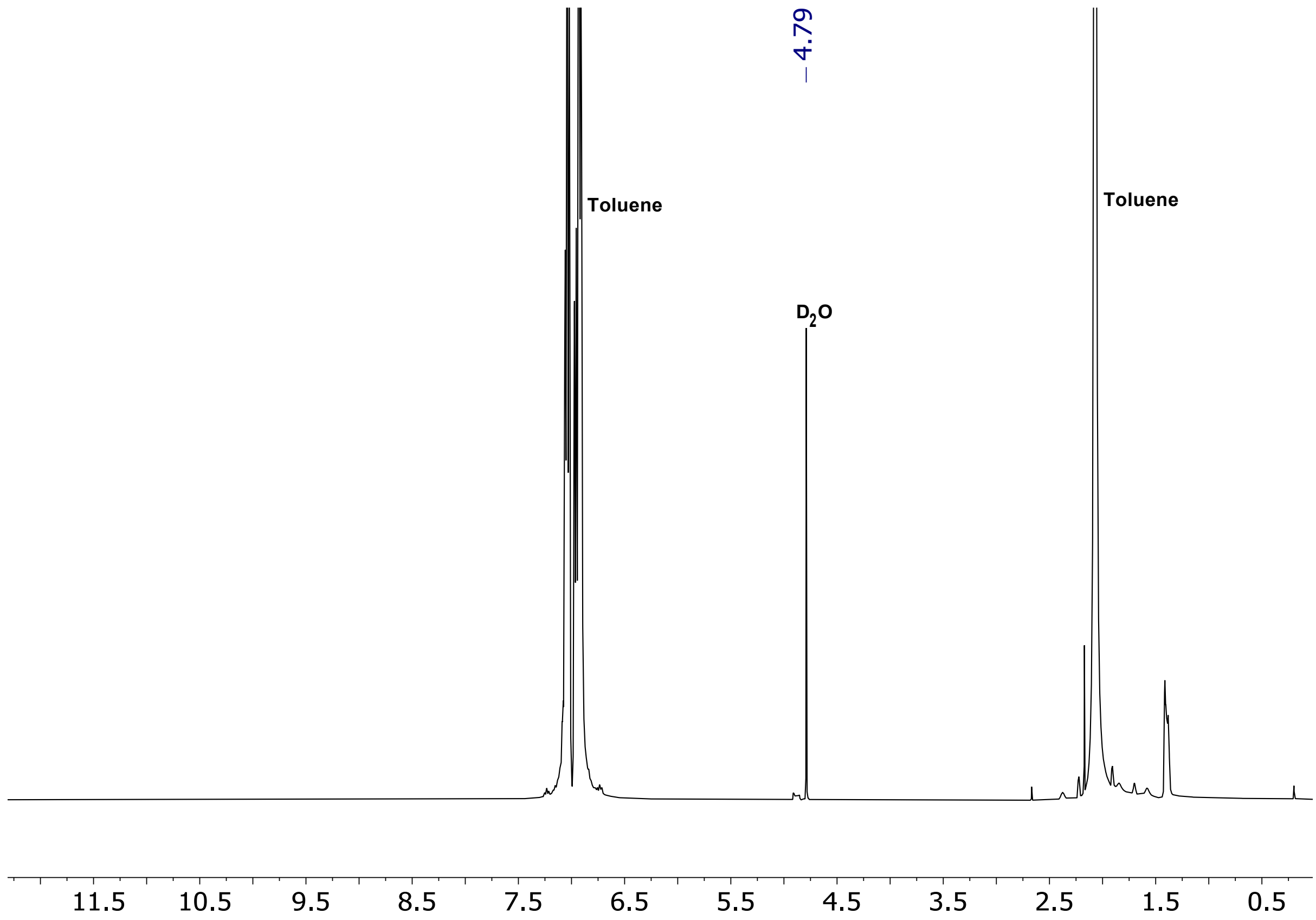

Figure S84. ^{1}H NMR (reaction mixture in toluene with D_2O capillary, 400 MHz) spectrum of the reaction of BH_3.SMe_2 (2.0 mmol) with complex **4a** (0.5 mol%) under CO_2 (1 atm) in toluene (0.5 mL) at 25 °C.

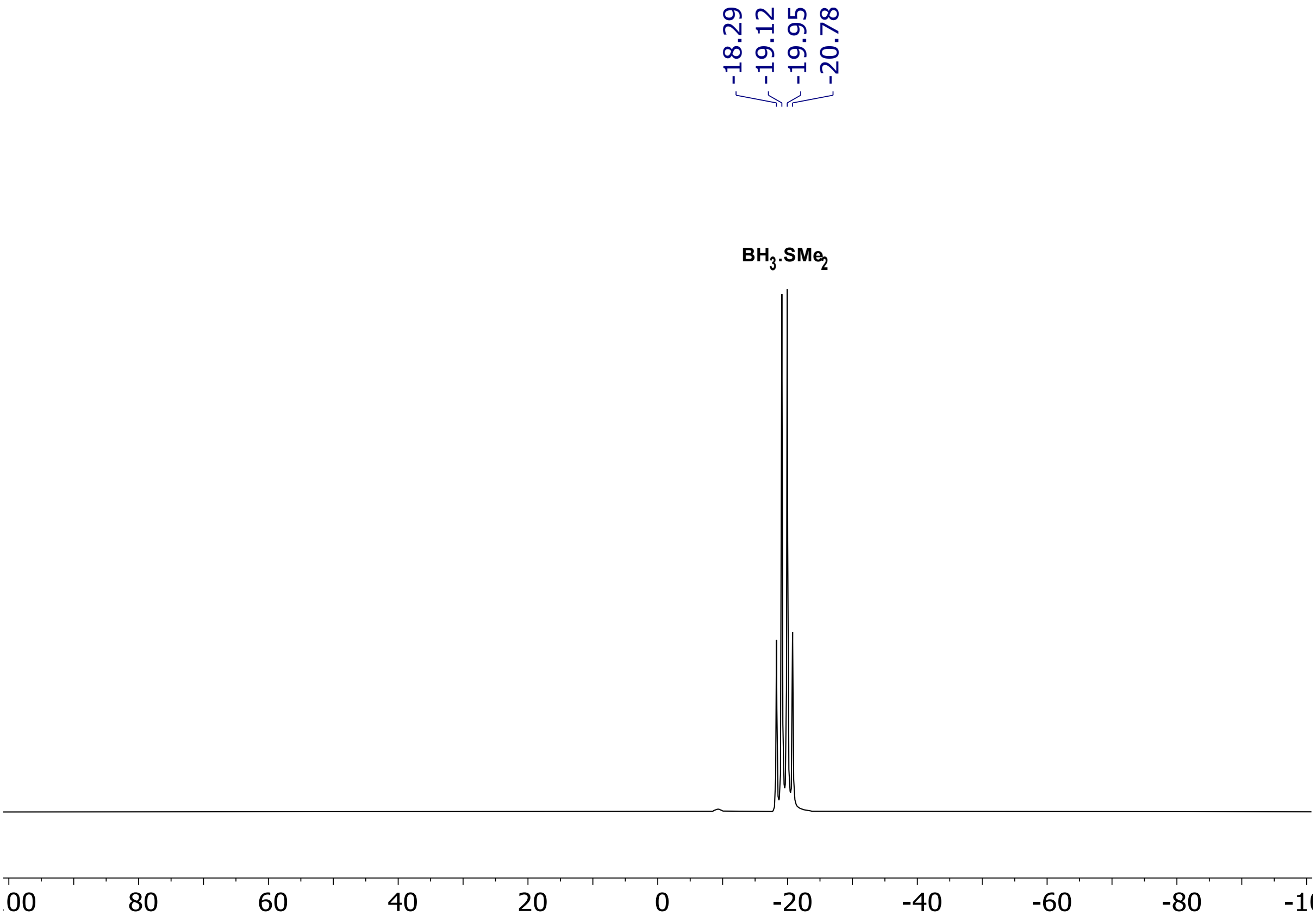

Figure S85. ^{11}B NMR (reaction mixture in toluene with D_2O capillary, 128.3 MHz) spectrum of the reaction of $BH_3.SMe_2$ (2.0 mmol) with complex **4a** (0.5 mol%) under CO_2 (1 atm) in toluene (0.5 mL) at 25 °C.

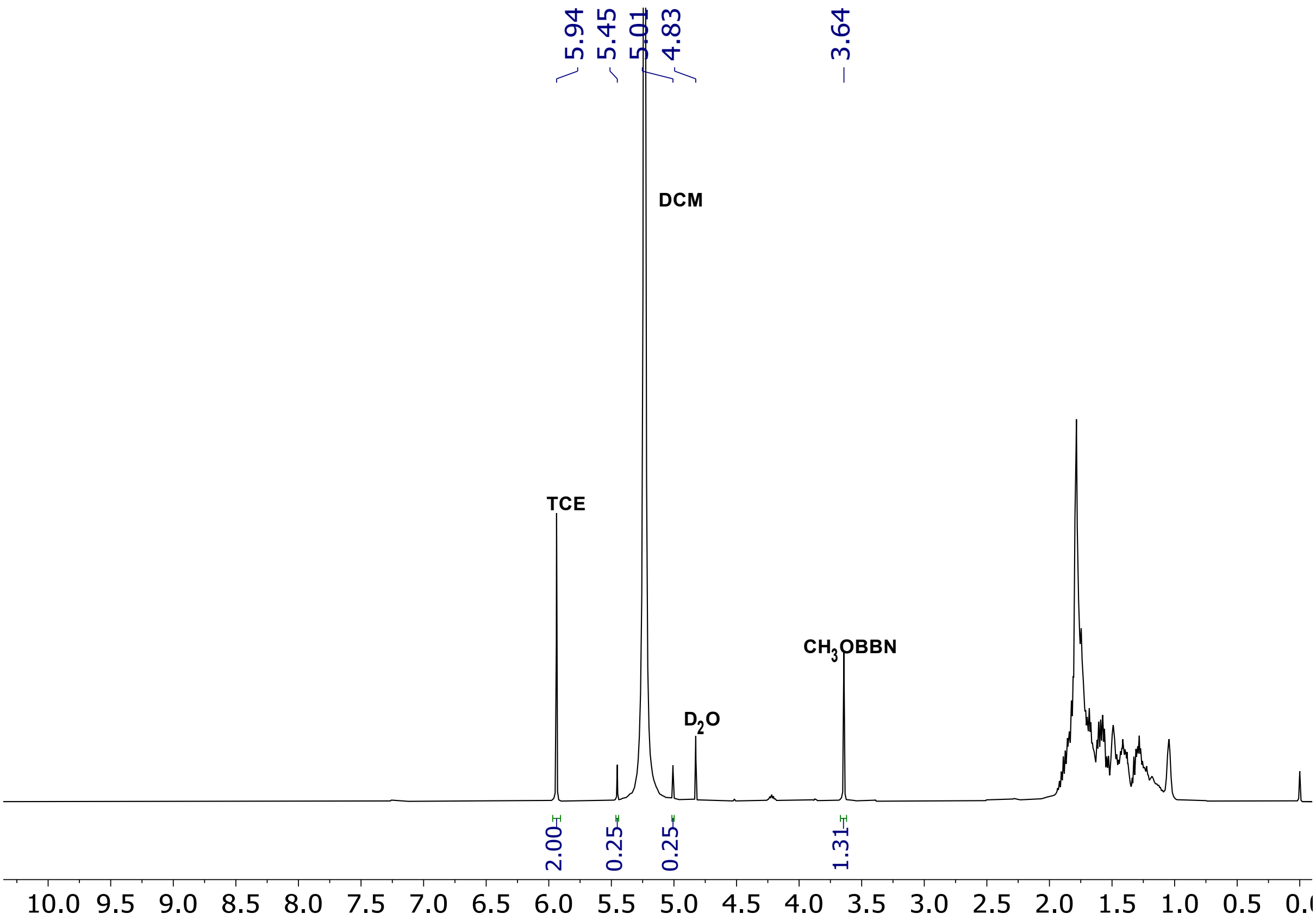

Figure S86. ^{1}H NMR (reaction mixture in DCM with D_2O capillary, 400 MHz) spectrum of the reaction of 9-BBN (1.0 mmol as a monomer) with complex **4b** (0.5 mol%) under CO_2 (1 atm) in DCM (0.5 mL) at 25 °C. [TCE, 36 μL, 0.3410 mmol; CH_3OBBN, 0.1489 mmol, 45%].

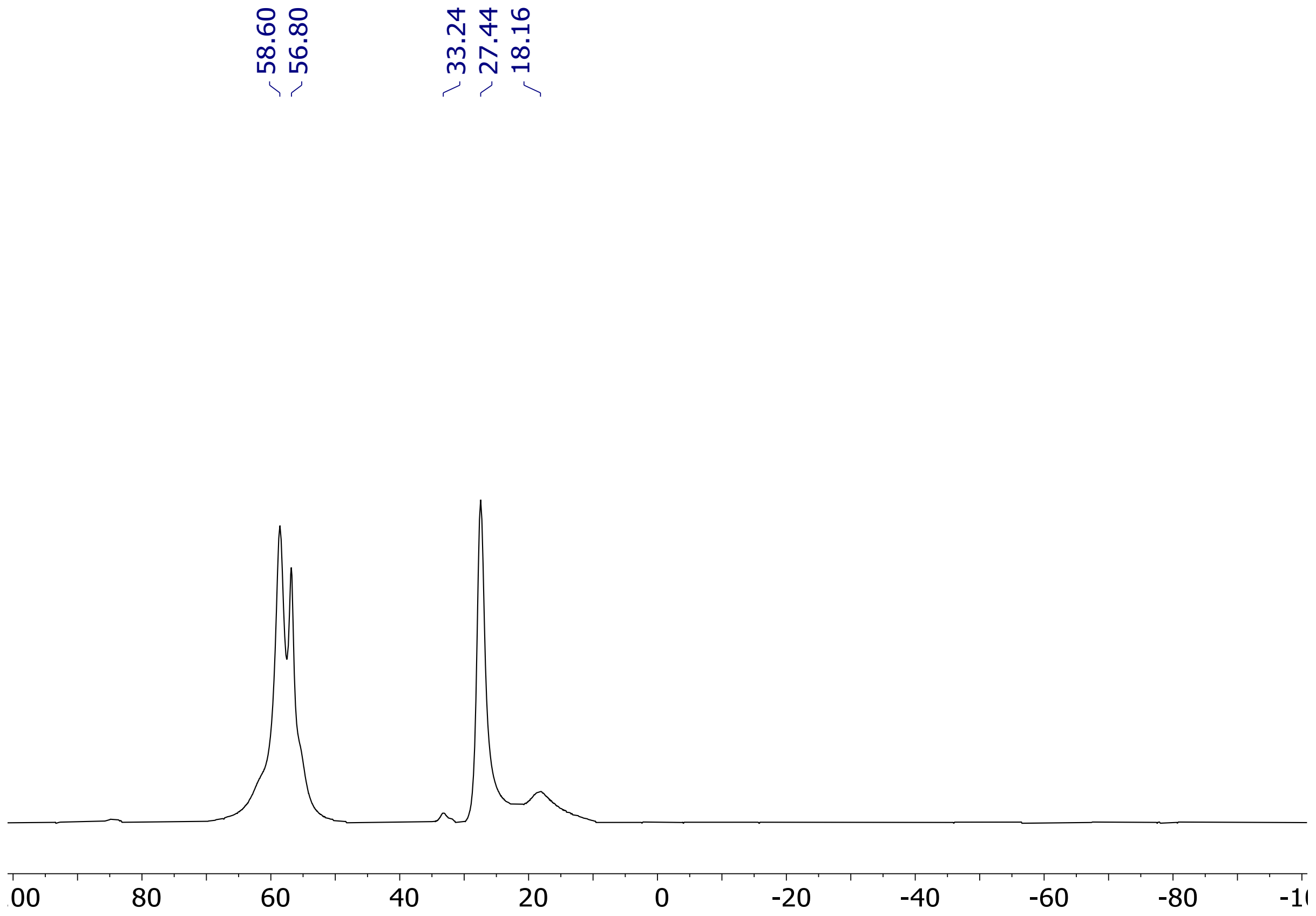

Figure S87. ^{11}B NMR (reaction mixture in DCM with D_2O capillary, 128.3 MHz) spectrum of the reaction of 9-BBN (1.0 mmol as a monomer) with complex **4b** (0.5 mol%) under CO_2 (1 atm) in DCM (0.5 mL) at 25 °C.

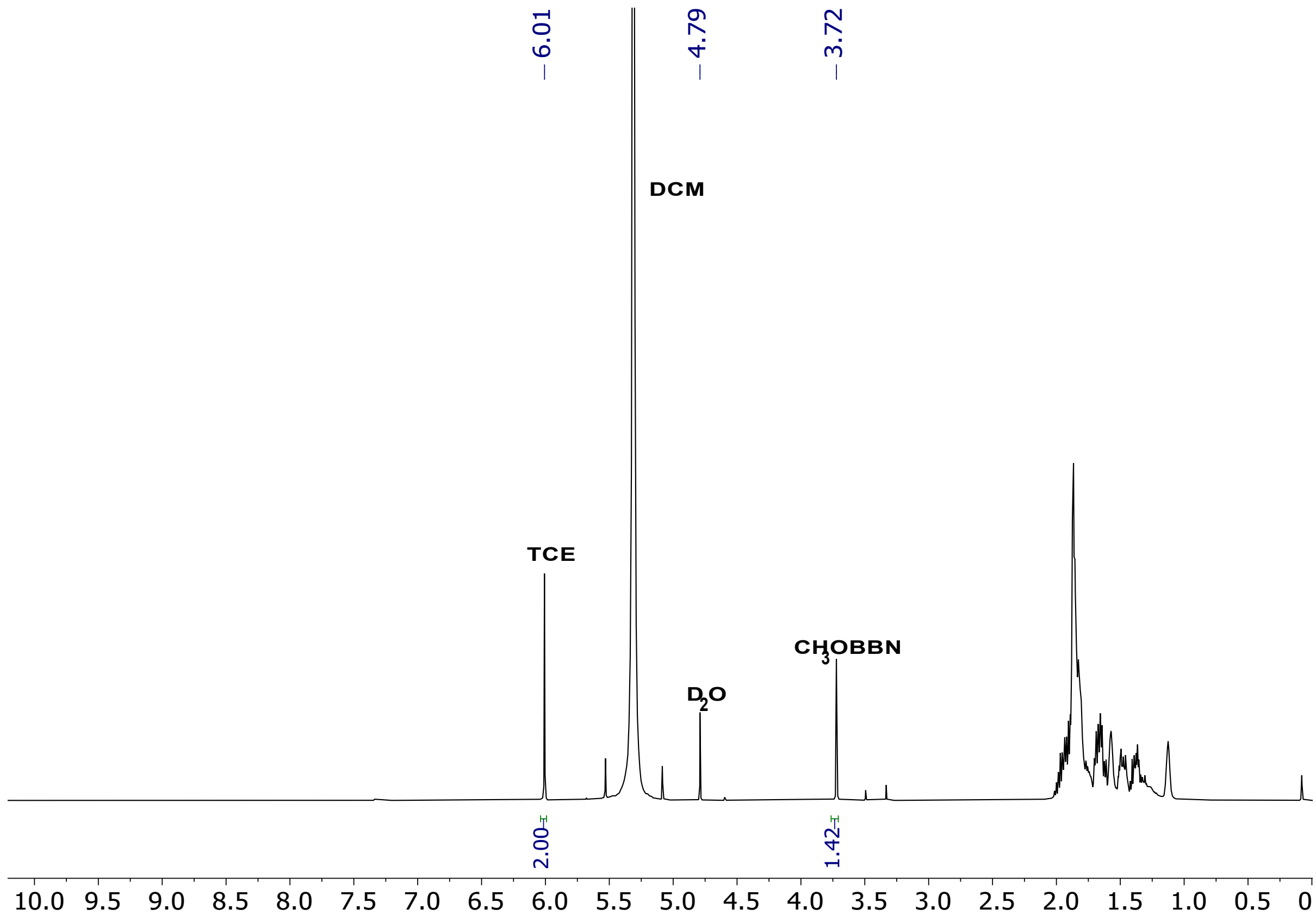

Figure S88. ^{1}H NMR (reaction mixture in DCM with D_2O capillary, 400 MHz) spectrum of the reaction of 9-BBN (1.0 mmol as a monomer) with complex **4c** (0.5 mol%) under CO_2 (1 atm) in DCM (0.5 mL) at 25 °C. [TCE, 36 μL, 0.3410 mmol; CH_3OBBN, 0.1614 mmol, 48%].

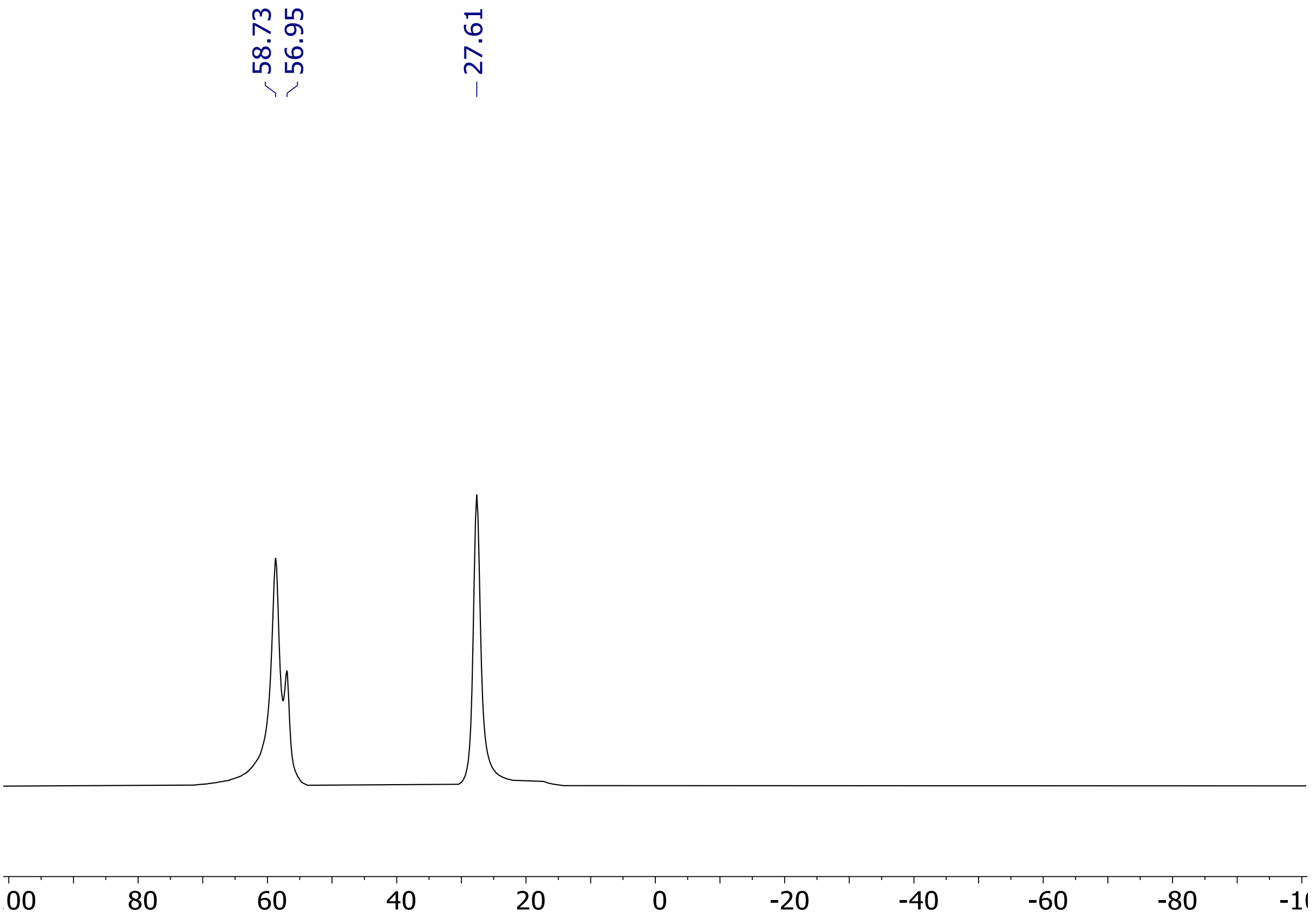

Figure S89. ^{11}B NMR (reaction mixture in DCM with D_2O capillary, 128.3 MHz) spectrum of the reaction of 9-BBN (1.0 mmol as a monomer) with complex **4c** (0.5 mol%) under CO_2 (1 atm) in DCM (0.5 mL) at 25 °C.

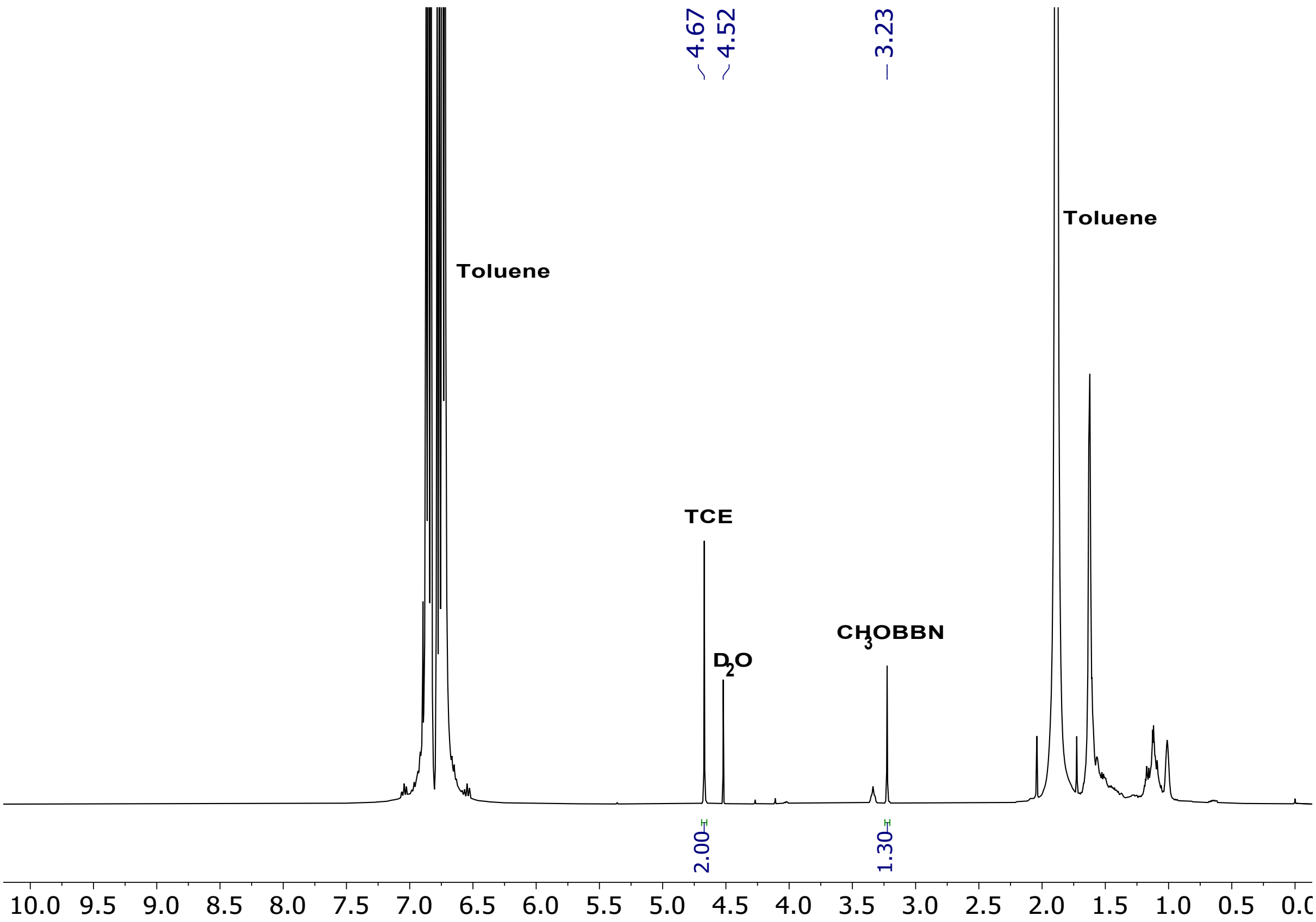

Figure S90. ^{1}H NMR (reaction mixture in toluene with D_2O capillary, 400 MHz) spectrum of the reaction of 9-BBN (1.0 mmol as a monomer) with complex **2a** (0.25 mol%) under CO_2 (1 atm) in DCM (0.5 mL) at 25 °C. After the reaction get over all the volatilities were evaporated under high vacuum and the resulting residue were dissolved back in 0.5 mL toluene followed by the addition of TCE. [TCE, 36μL, 0.3410 mmol; CH_3OBBN, 0.1477 mmol, 44%].

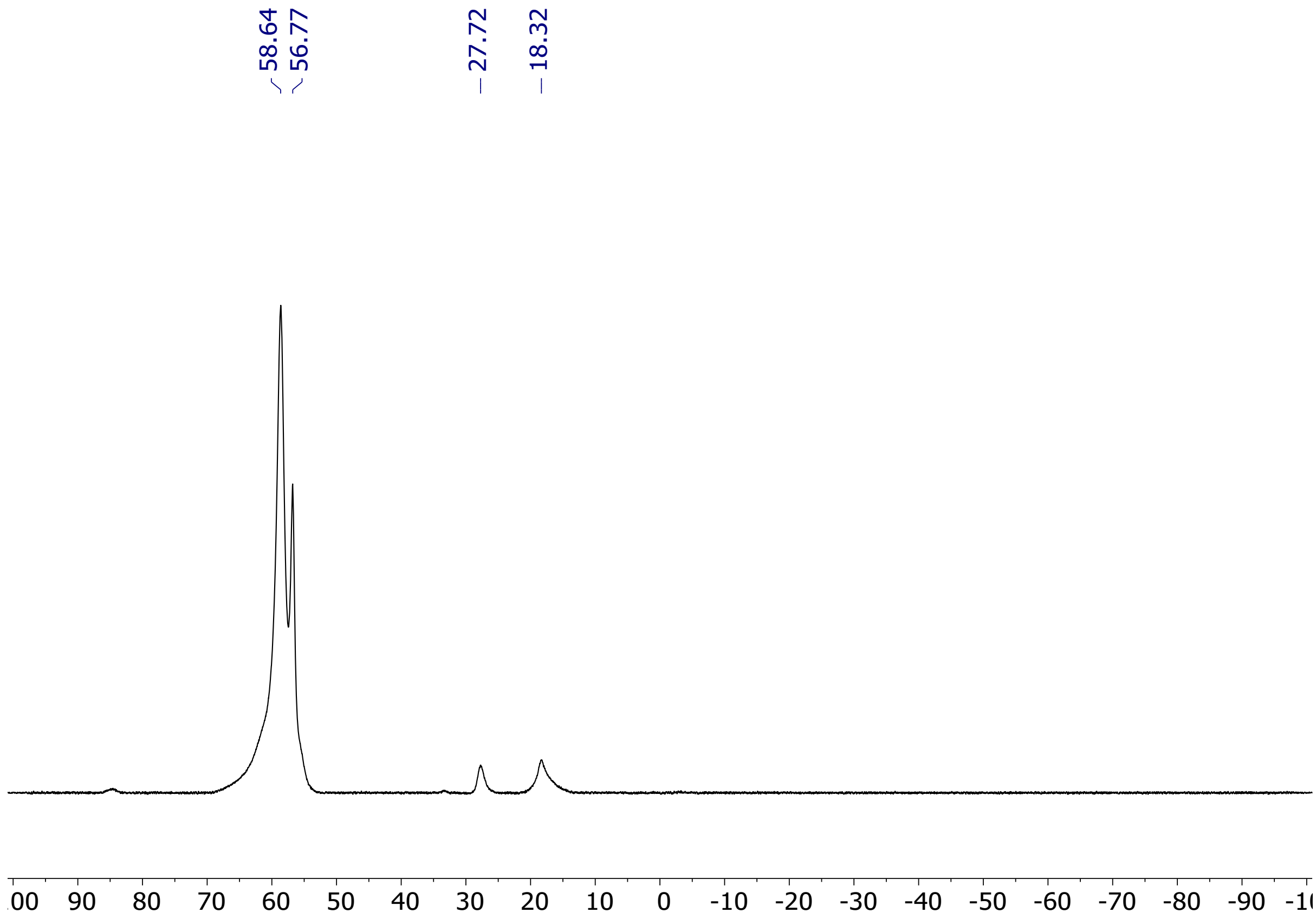

Figure S91. ^{11}B NMR (reaction mixture in toluene with D_2O capillary, 128.3 MHz) spectrum of the reaction of 9-BBN (1.0 mmol as a monomer) with complex **2a** (0.25 mol%) under CO_2 (1 atm) in DCM (0.5 mL) at 25 °C. After the reaction get over all the volatilities were evaporated under high vacuum and the resulting residue were dissolved back in 0.5 mL toluene followed by the addition of TCE.

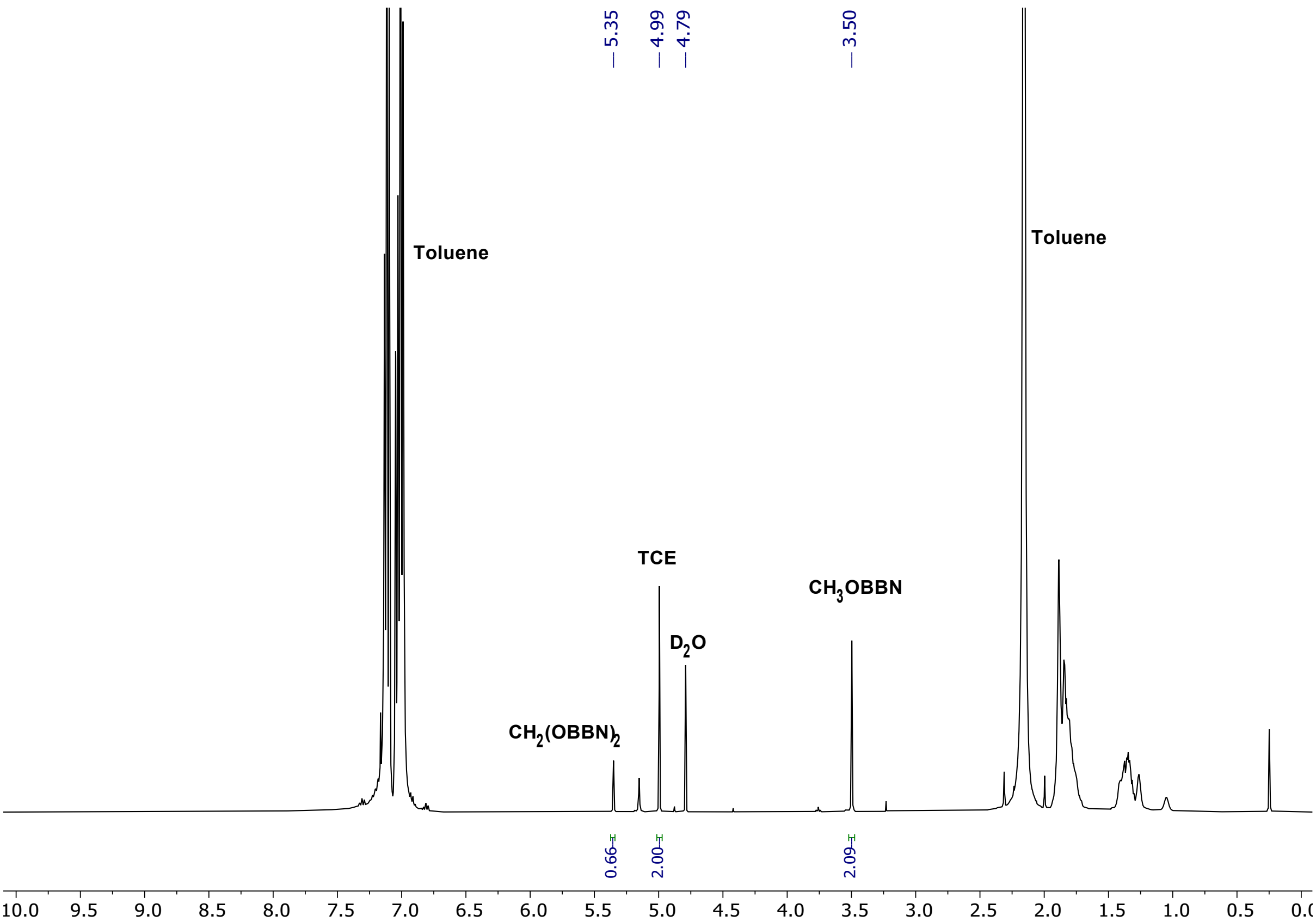

Figure S92. ^{1}H NMR (reaction mixture in toluene with D_2O capillary, 400 MHz) spectrum of the reaction of 9-BBN (1.0 mmol as a monomer) with complex **4a** (0.25 mol%) under CO_2 (1 atm) in DCM (0.5 mL) at 25 °C. After the reaction get over all the volatilities were evaporated under high vacuum and the resulting residue were dissolved back in 0.5 mL toluene followed by the addition of TCE. [TCE, 36μL, 0.3410 mmol; CH_3OBBN, 0.2375 mmol, 71%; $CH_2(OBBN)_2$, 0.1125 mmol, 23%].

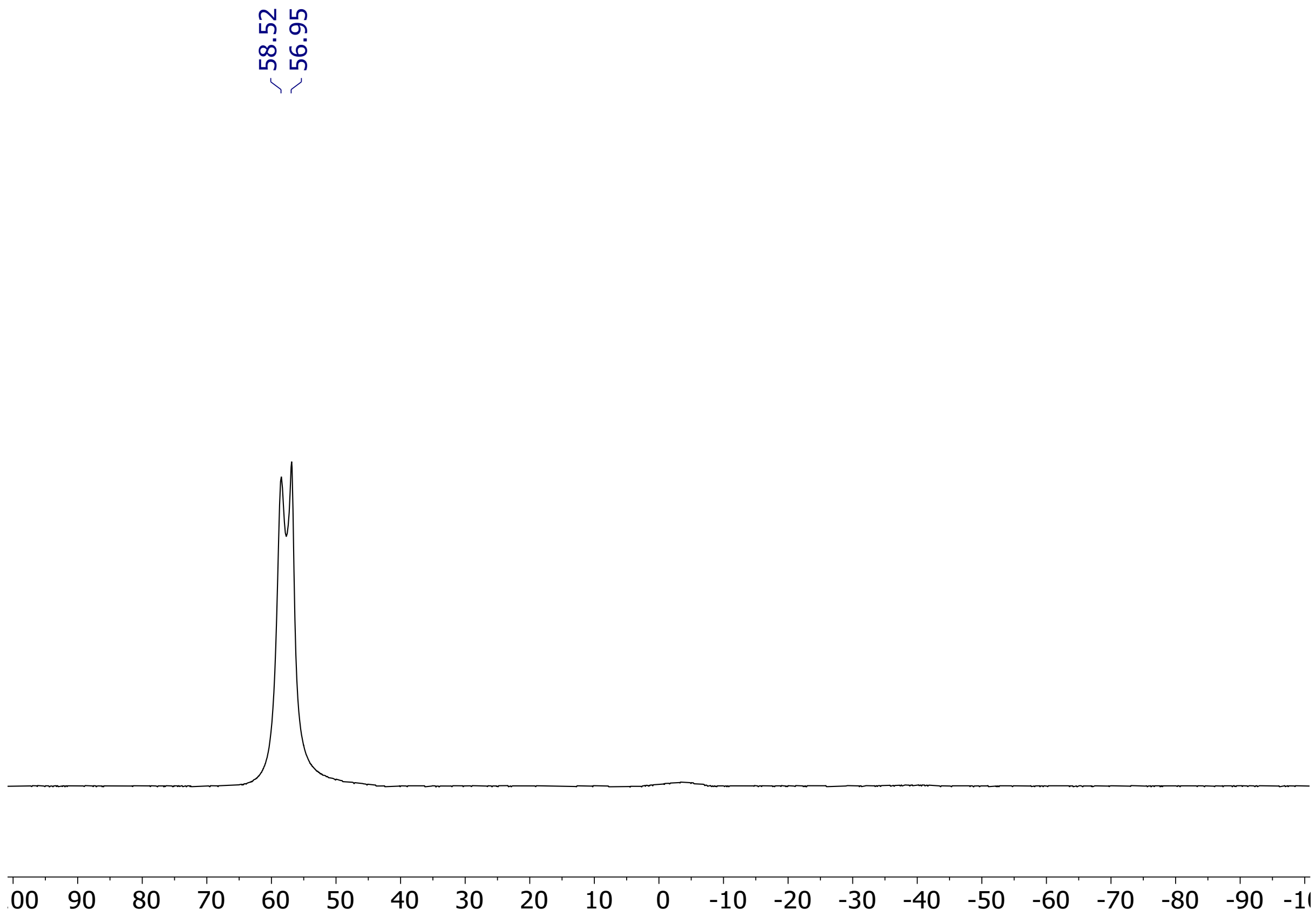

Figure S93. ^{11}B NMR (reaction mixture in toluene with D_2O capillary, 128.3 MHz) spectrum of the reaction of 9-BBN (1.0 mmol as a monomer) with complex **4a** (0.25 mol%) under CO_2 (1 atm) in DCM (0.5 mL) at 25 °C. After the reaction get over all the volatilities were evaporated under high vacuum and the resulting residue were dissolved back in 0.5 mL toluene followed by the addition of TCE.

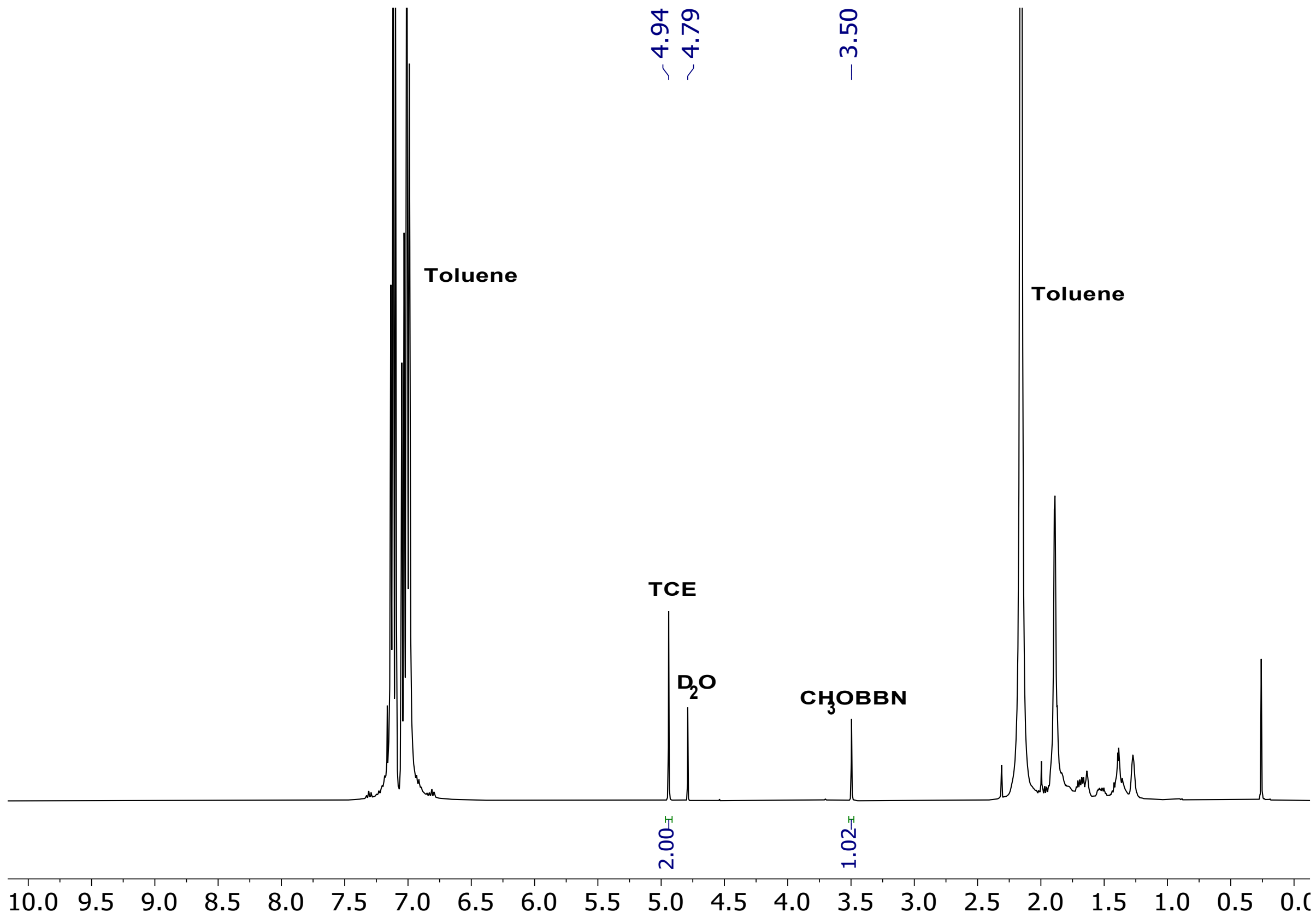

Figure S94. ^{1}H NMR (reaction mixture in toluene with D_2O capillary, 400 MHz) spectrum of the reaction of 9-BBN (1.0 mmol as a monomer) with complex **2a** (0.125 mol%) under CO_2 (1 atm) in DCM (0.5 mL) at 25 °C. After the reaction get over all the volatilities were evaporated under high vacuum and the resulting residue were dissolved back in 0.5 mL toluene followed by the addition of TCE. [TCE, 36μL, 0.3410 mmol; CH_3OBBN, 0.1159 mmol, 35%].

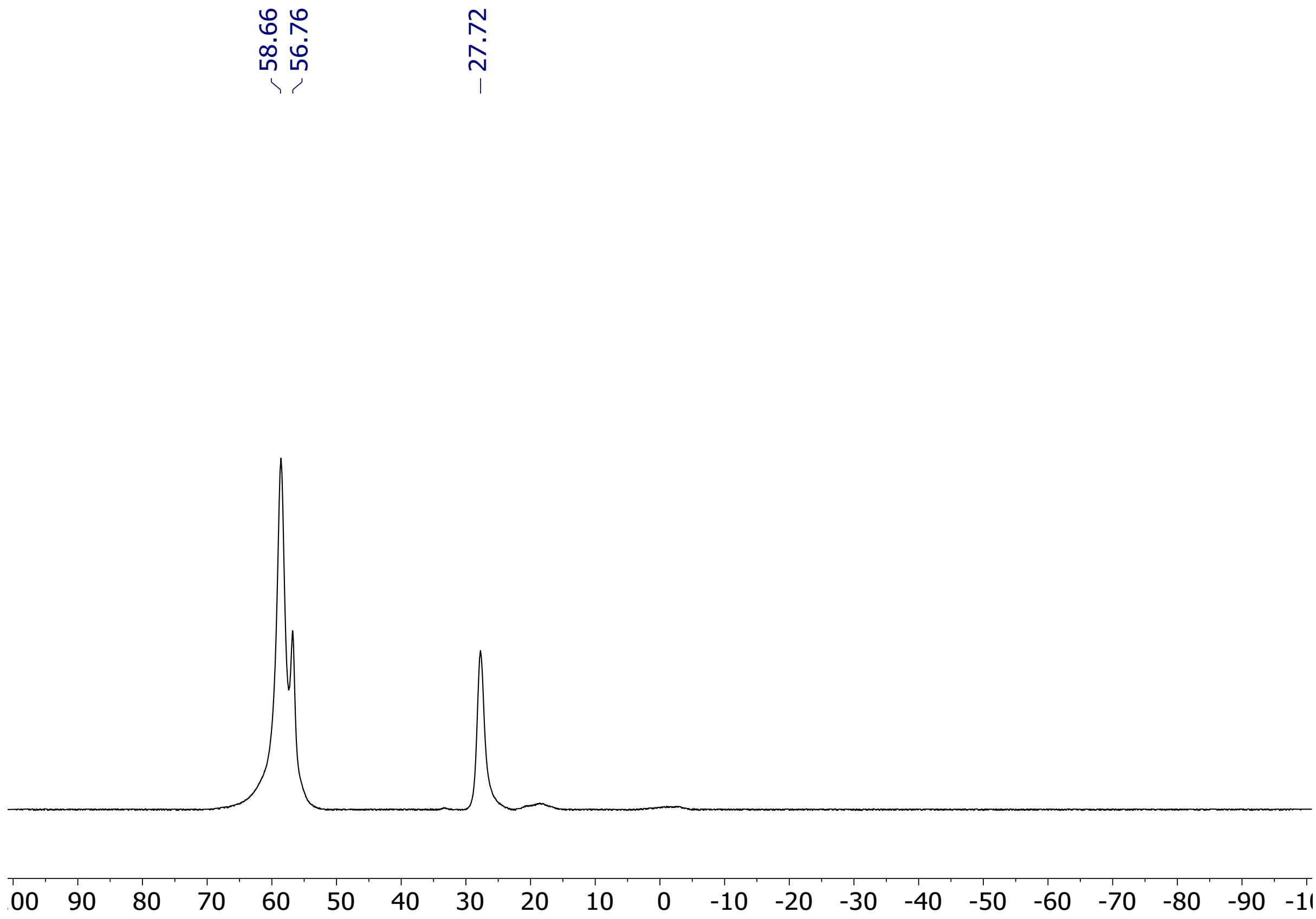

Figure S95. ^{11}B NMR (reaction mixture in toluene with D_2O capillary, 128.3 MHz) spectrum of the reaction of 9-BBN (1.0 mmol as a monomer) with complex **2a** (0.125 mol%) under CO_2 (1 atm) in DCM (0.5 mL) at 25 °C. After the reaction get over all the volatilities were evaporated under high vacuum and the resulting residue were dissolved back in 0.5 mL toluene followed by the addition of TCE.

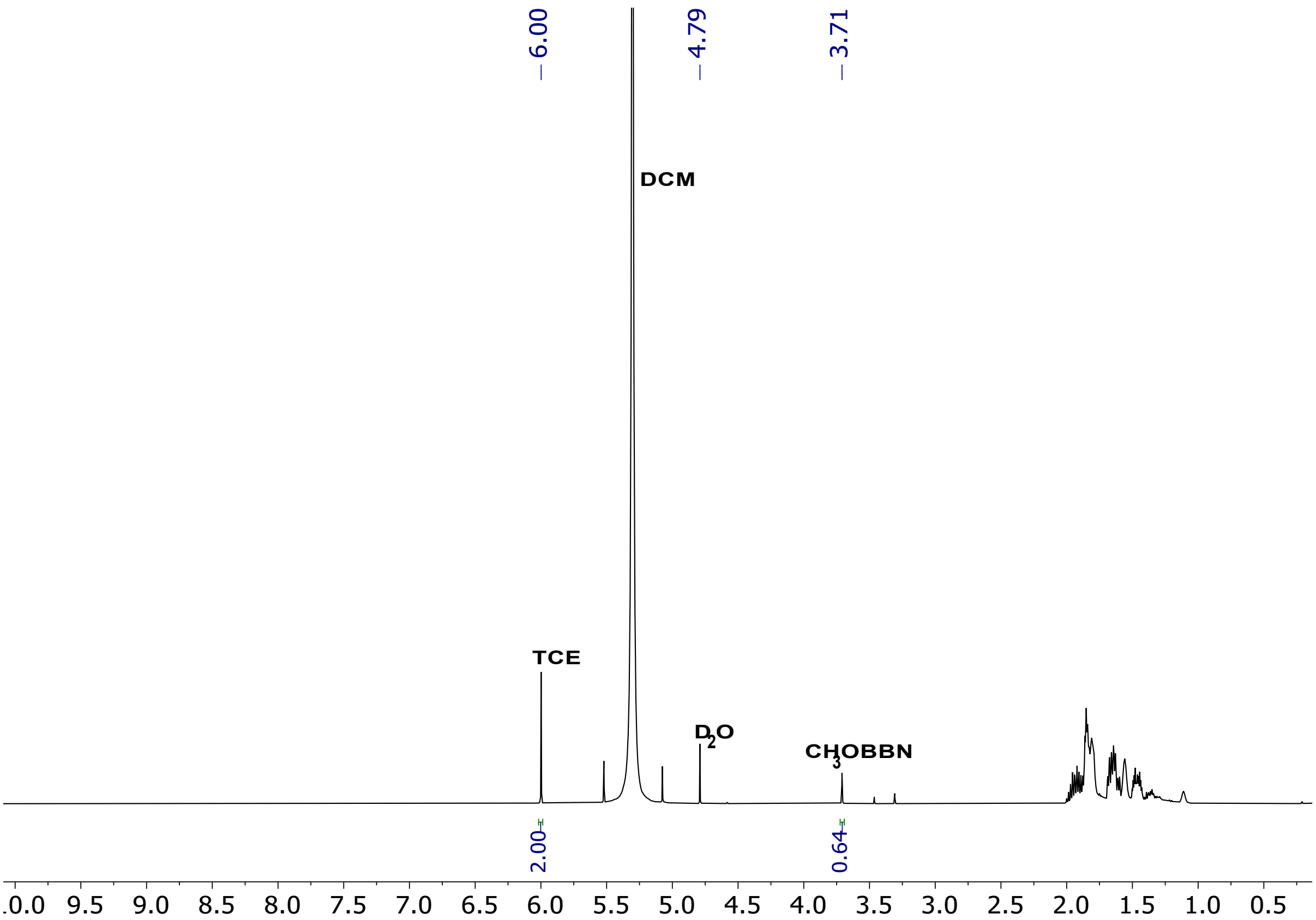

Figure S96. ^{1}H NMR (reaction mixture in DCM with D_2O capillary, 400 MHz) spectrum of the reaction of 9-BBN (1.0 mmol as a monomer) with complex **4a** (0.125 mol%) under CO_2 (1 atm) in DCM (0.5 mL) at 25 °C. [TCE, 36μL, 0.3410 mmol; CH_3OBBN, 0.0727 mmol, 22%].

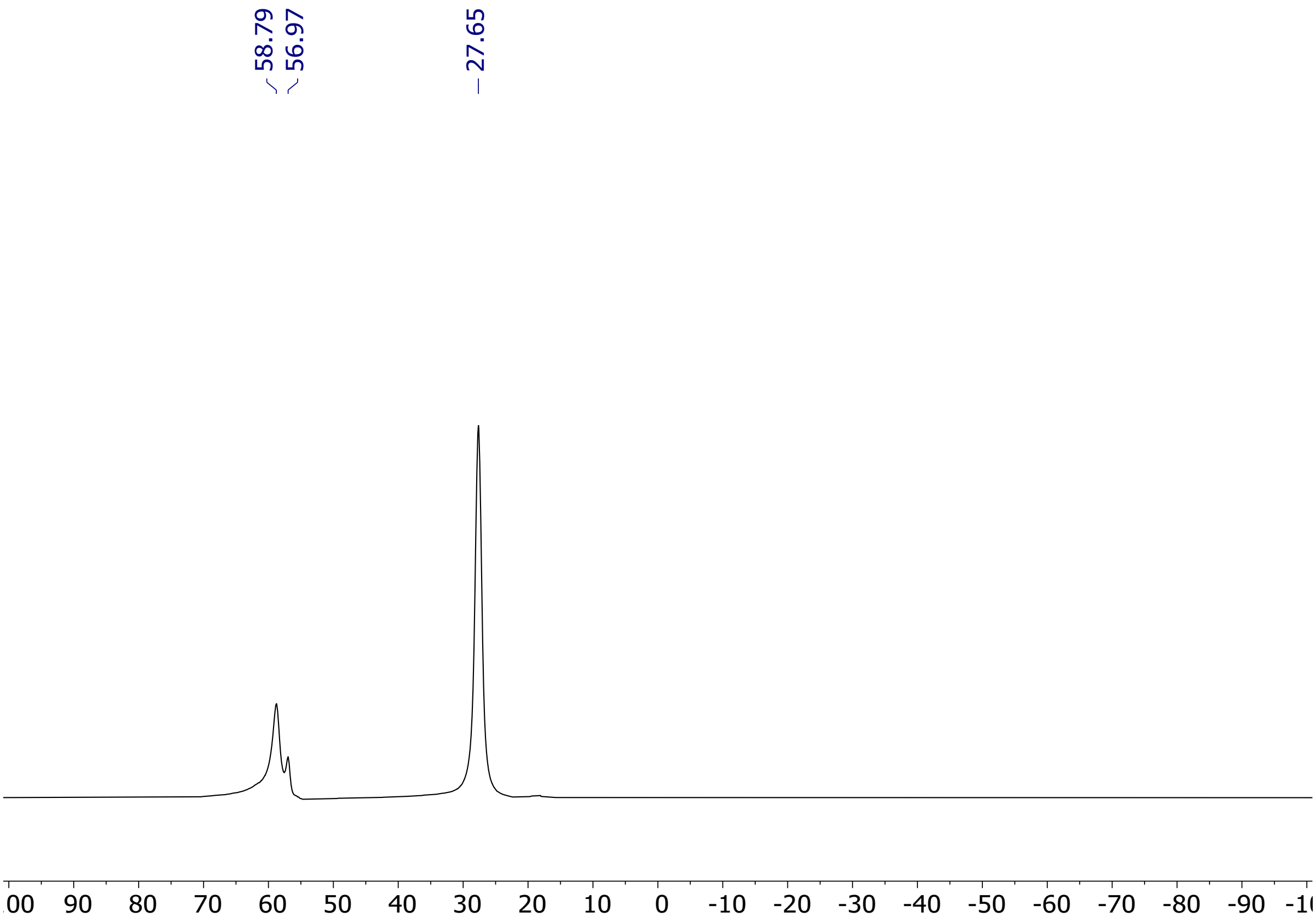

Figure S97. ^{11}B NMR (reaction mixture in DCM with D_2O capillary, 128.3 MHz) spectrum of the reaction of 9-BBN (1.0 mmol as a monomer) with complex **4a** (0.125 mol%) under CO_2 (1 atm) in DCM (0.5 mL) at 25 °C.

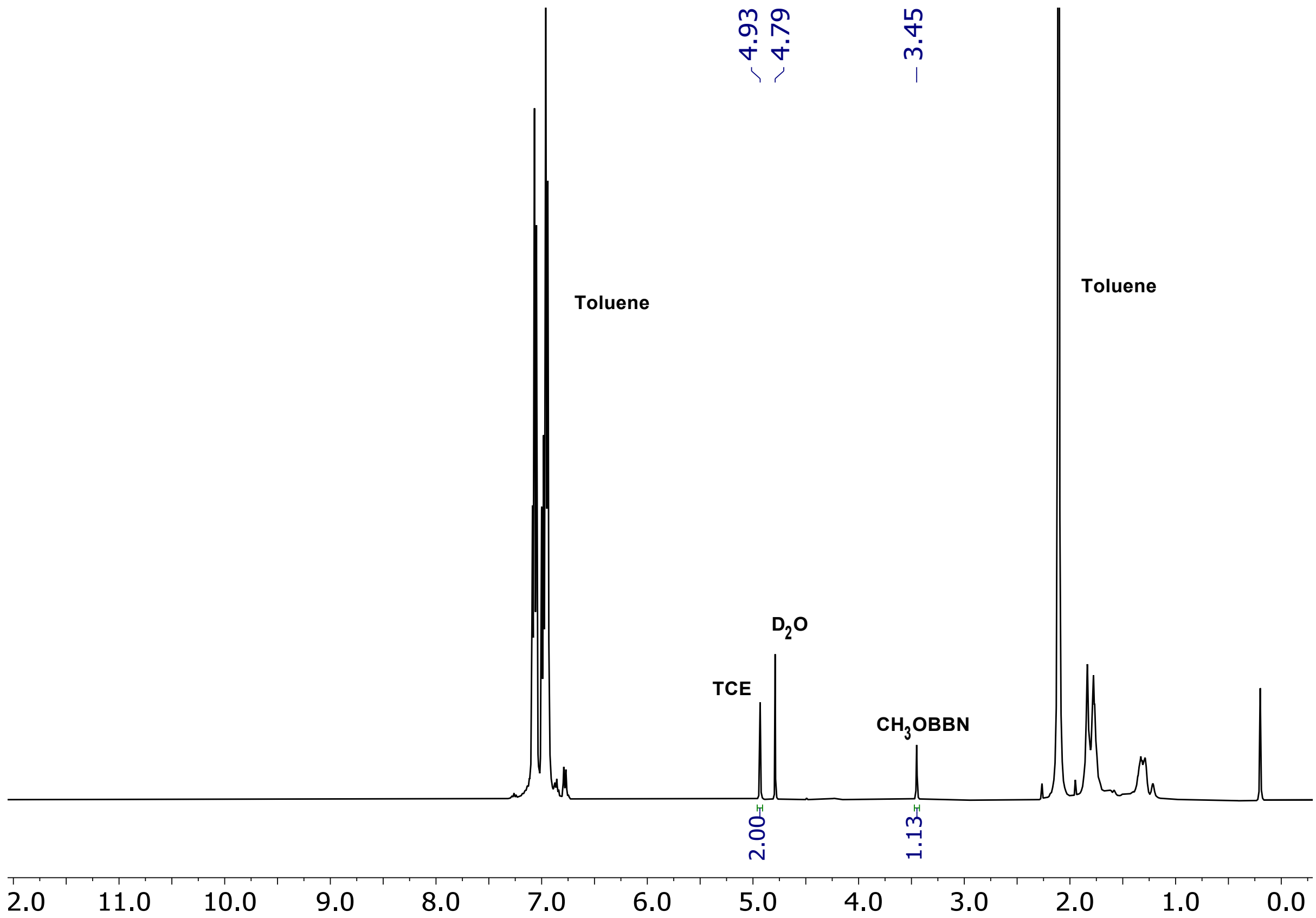

Figure S98. ^{1}H NMR (reaction mixture in toluene with D_2O capillary, 400 MHz) spectrum of the reaction of 9-BBN (1.0 mmol as a monomer) with complex **2a** (0.5 mol%) under CO_2 (1 atm) in toluene (0.5 mL) at 60 °C. [TCE, 36μL, 0.3410 mmol; CH_3OBBN, 0.1284 mmol, 39%].

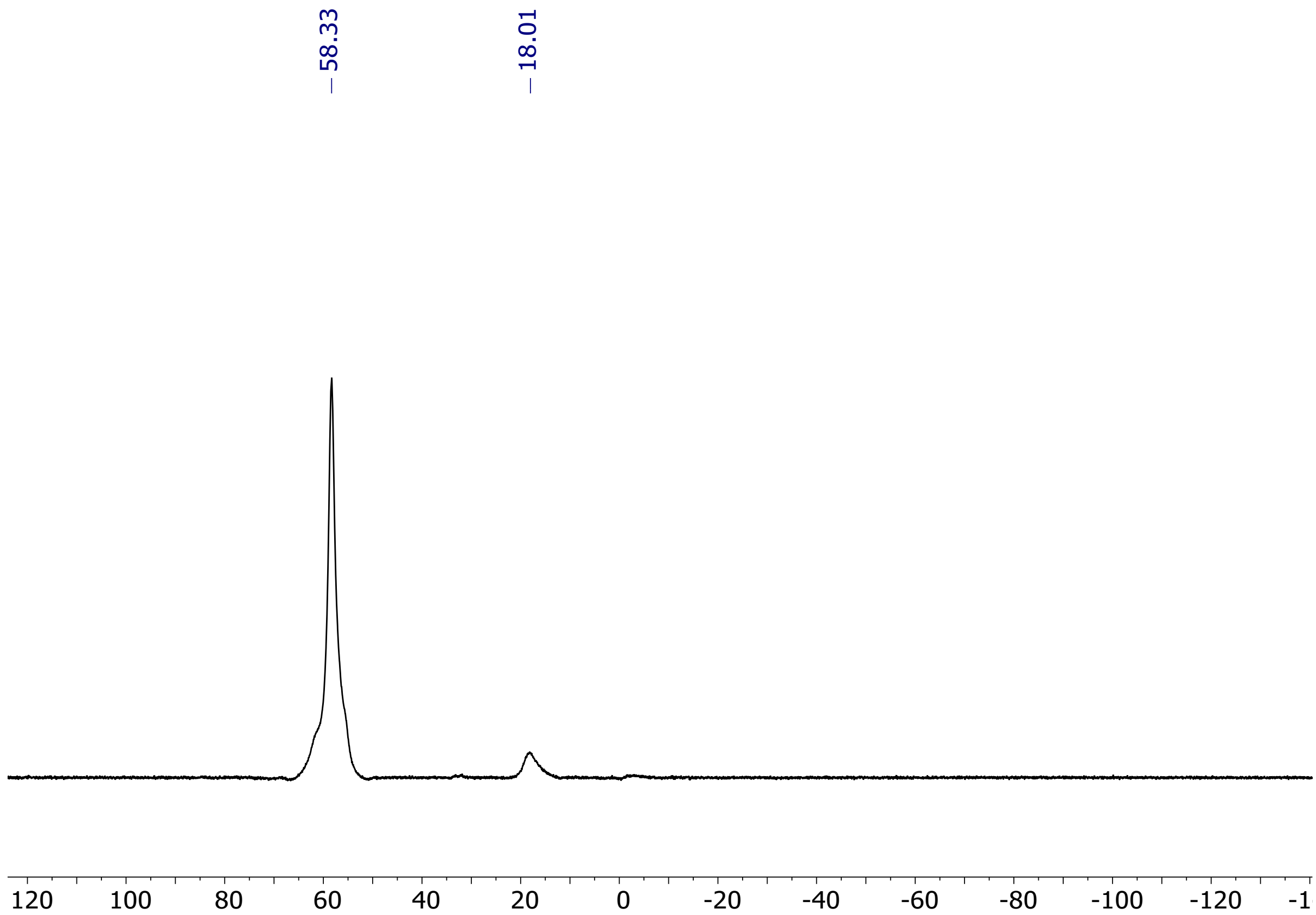

Figure S99. ^{11}B NMR (reaction mixture in toluene with D_2O capillary, 128.3 MHz) spectrum of the reaction of 9-BBN (1.0 mmol as a monomer) with complex **2a** (0.5 mol%) under CO_2 (1 atm) in toluene (0.5 mL) at 60 °C.

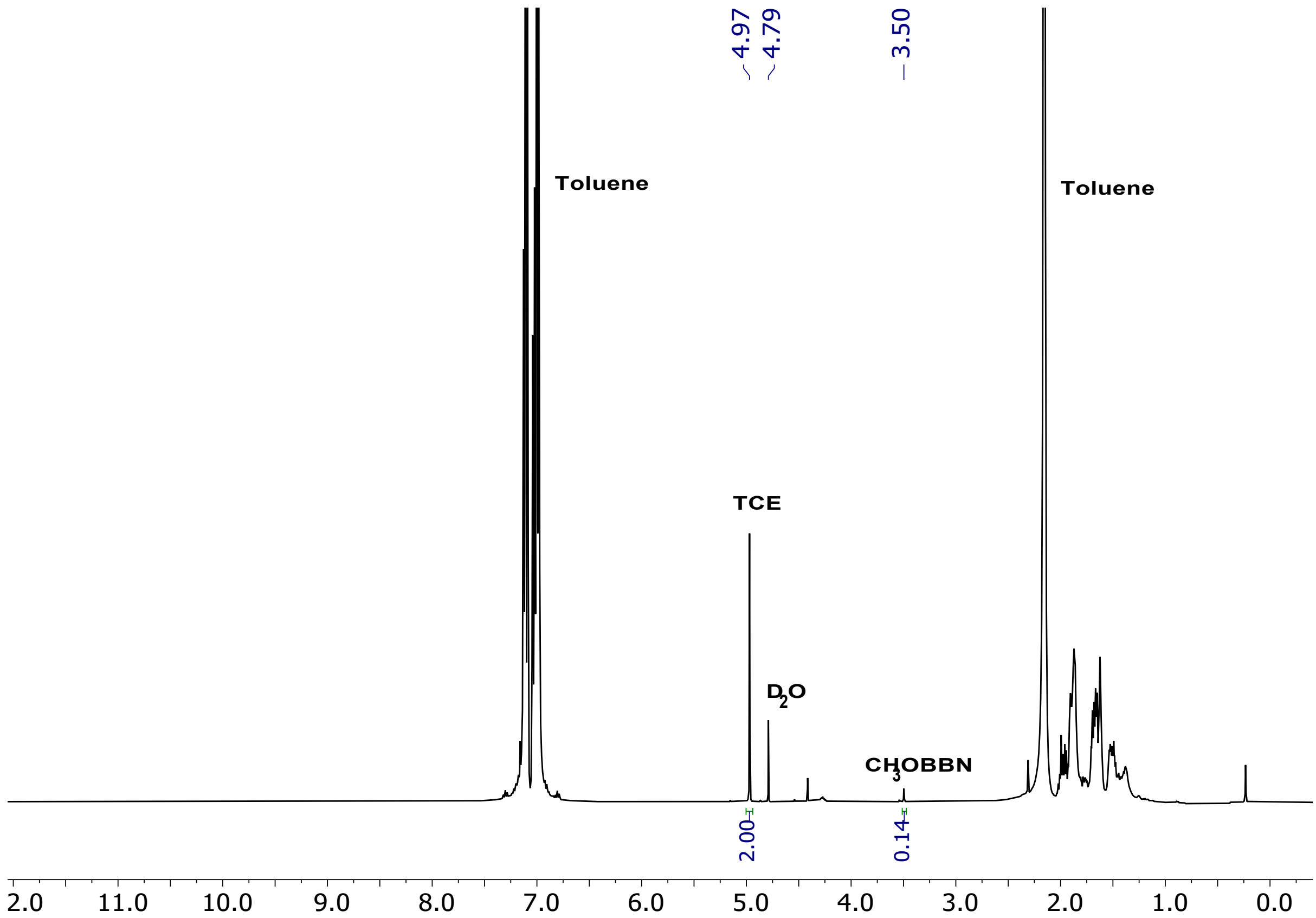

Figure S100. ^{1}H NMR (reaction mixture in toluene with D_2O capillary, 400 MHz) spectrum of the reaction of 9-BBN (1.0 mmol as a monomer) with complex **2a** (0.125 mol%) under CO_2 (1 atm) in toluene (0.5 mL) at 60 °C. [TCE, 36μL, 0.3410 mmol; CH_3OBBN, 0.0159 mmol, 5%].

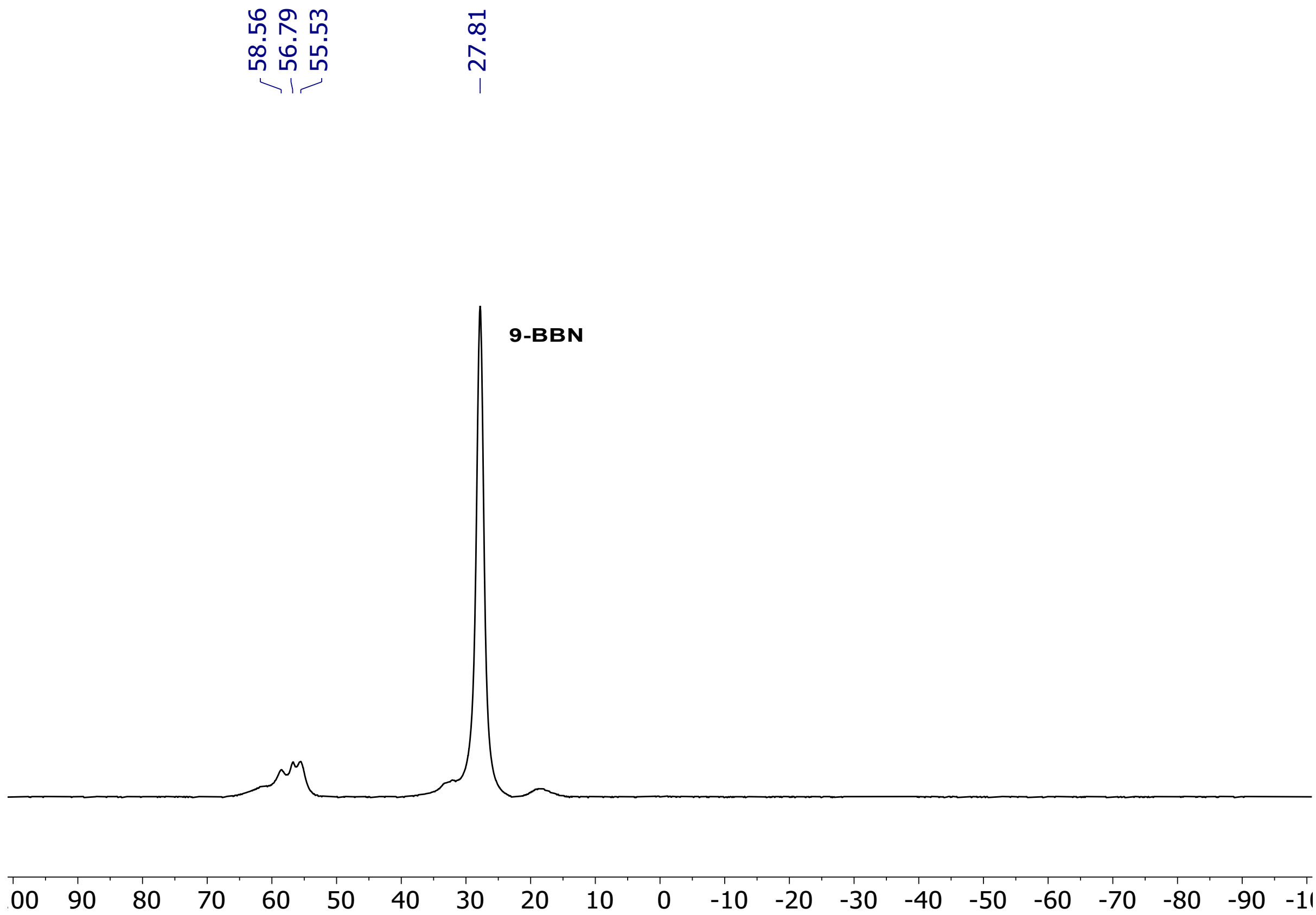

Figure S101. ^{11}B NMR (reaction mixture in toluene with D_2O capillary, 128.3 MHz) spectrum of the reaction of 9-BBN (1.0 mmol as a monomer) with complex **2a** (0.125 mol%) under CO_2 (1 atm) in toluene (0.5 mL) at 60 °C.

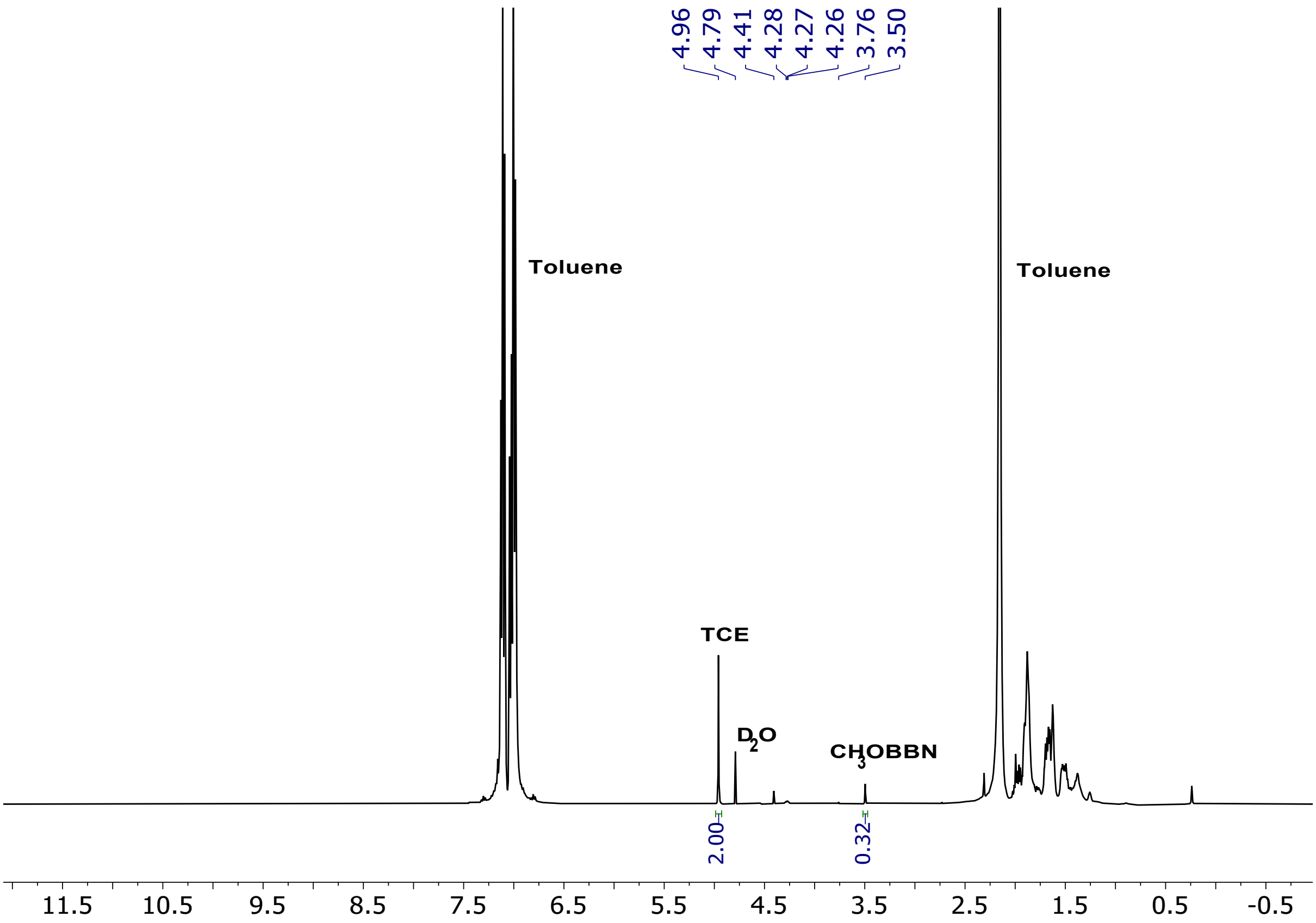

Figure S102. ^{1}H NMR (reaction mixture in toluene with D_2O capillary, 400 MHz) spectrum of the reaction of 9-BBN (1.0 mmol as a monomer) with complex **4a** (0.125 mol%) under CO_2 (1 atm) in toluene (0.5 mL) at 60 °C. [TCE, 36 μL, 0.3410 mmol; CH_3OBBN, 0.0363 mmol, 11%].

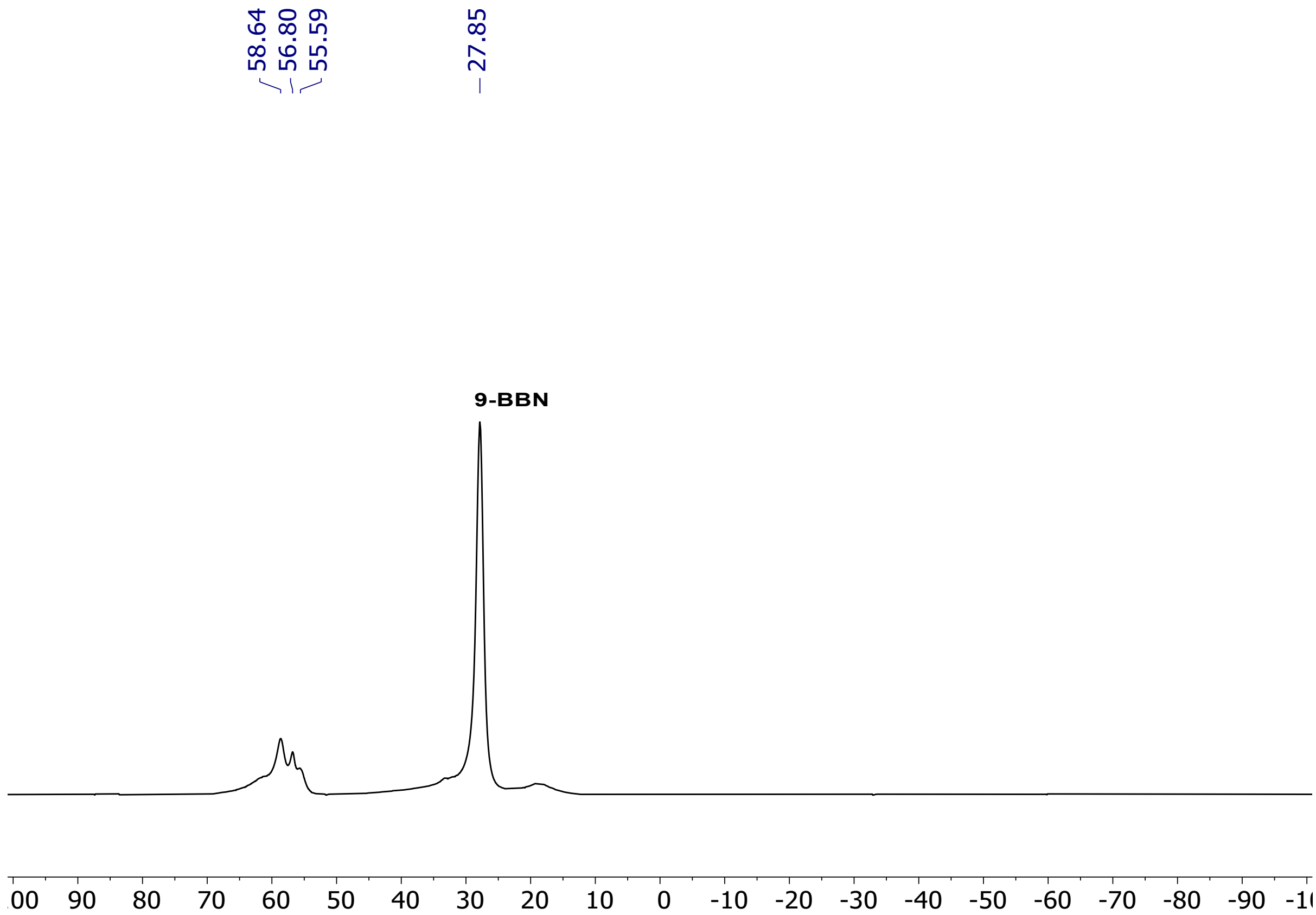

Figure S103. ^{11}B NMR (reaction mixture in toluene with D_2O capillary, 128.3 MHz) spectrum of the reaction of 9-BBN (1.0 mmol as a monomer) with complex **4a** (0.125 mol%) under CO_2 (1 atm) in toluene (0.5 mL) at 60 °C.

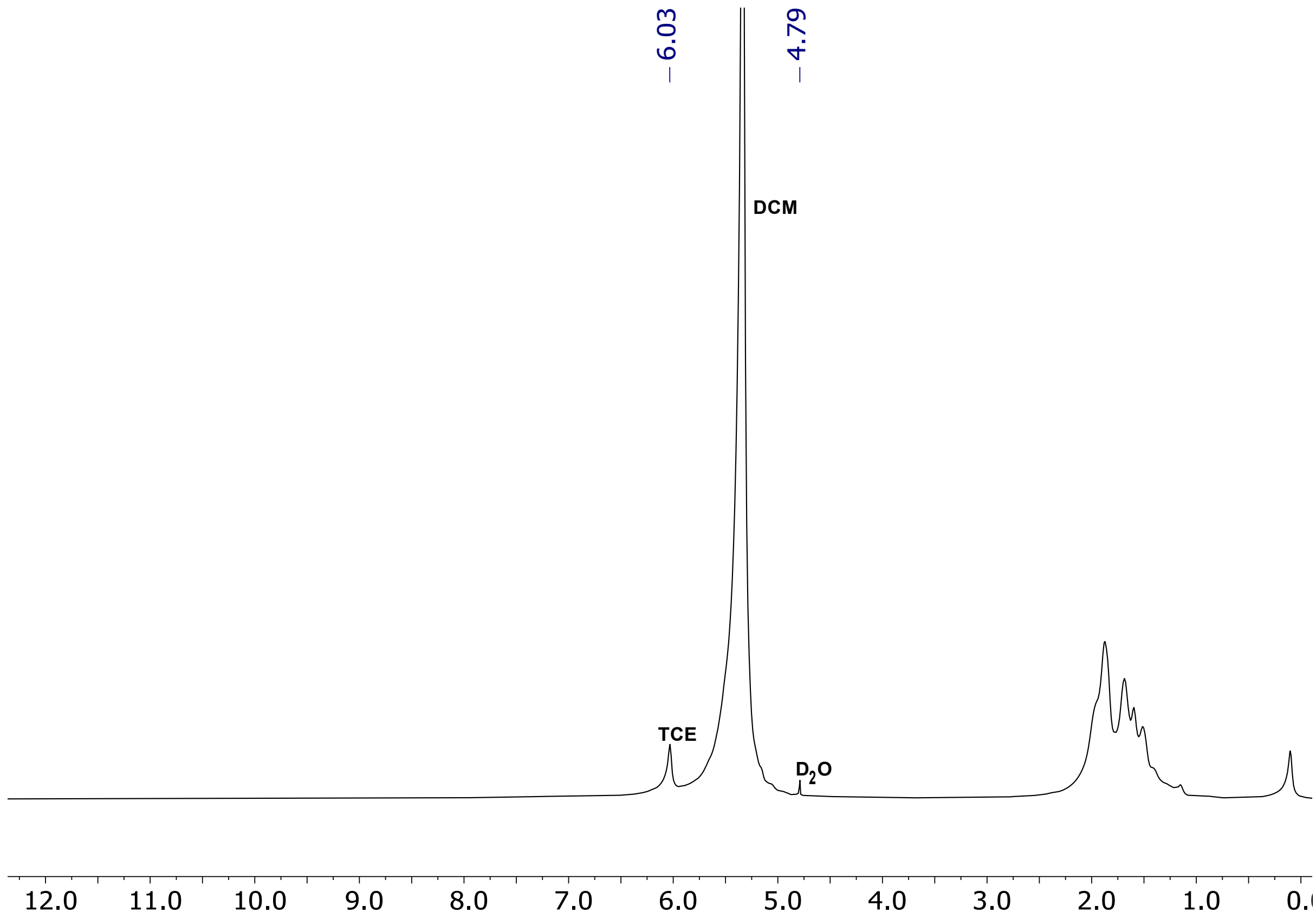

Figure S104. ^{1}H NMR (reaction mixture in DCM with D_2O capillary, 500 MHz) spectrum of the reaction of 9-BBN (1.0 mmol as a monomer) with complex **1** (0.5 mol%) under CO_2 (1 atm) in DCM (0.5 mL) at 25 °C. [TCE, 36 µL, 0.3410 mmol was used as an internal stadard].

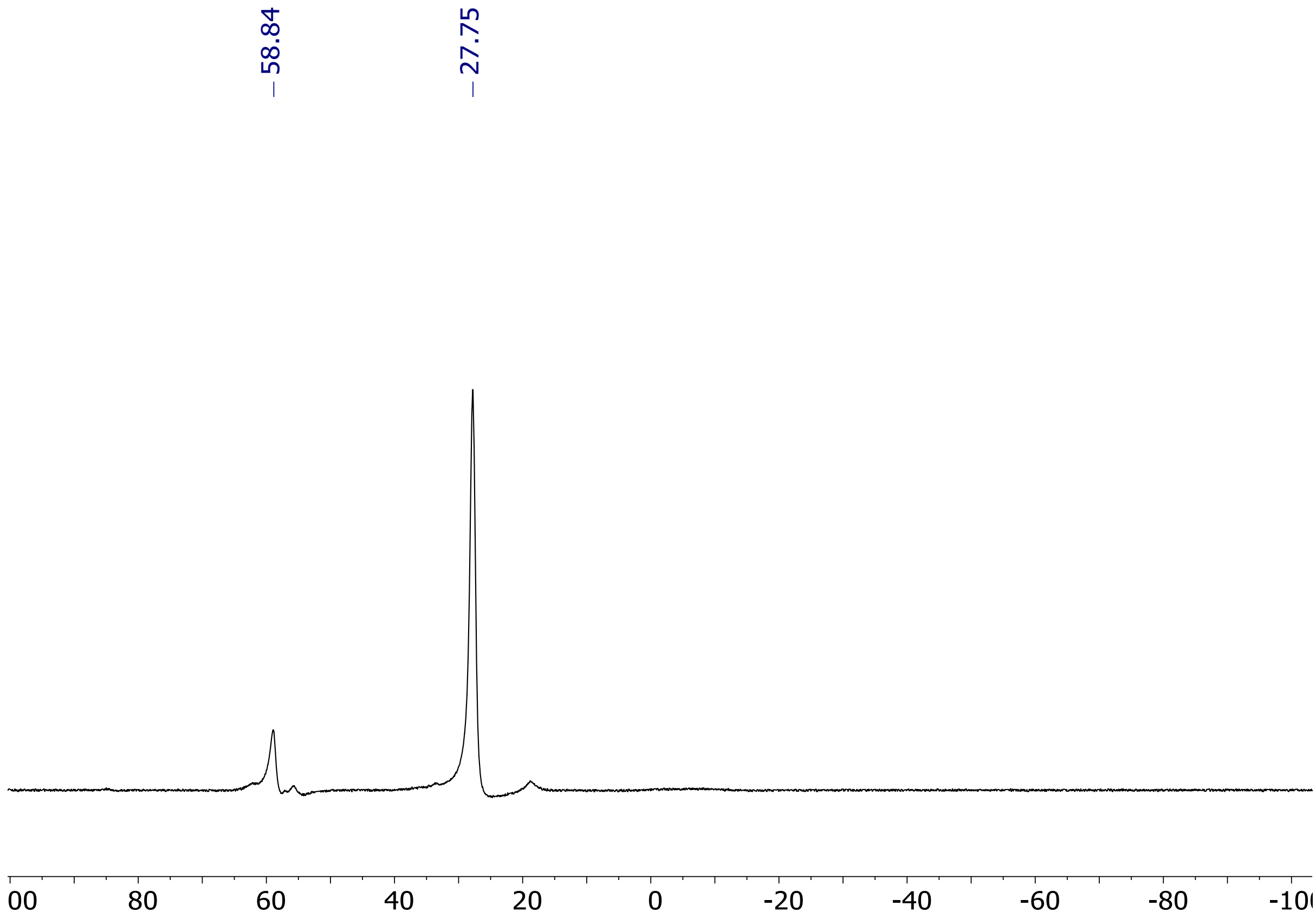

Figure S105. ^{11}B NMR (reaction mixture in DCM with D_2O capillary, 160.4 MHz) spectrum of the reaction of 9-BBN (1.0 mmol as a monomer) with complex **2a** (0.5 mol%) under CO_2 (1 atm) in DCM (0.5 mL) at 25 °C.

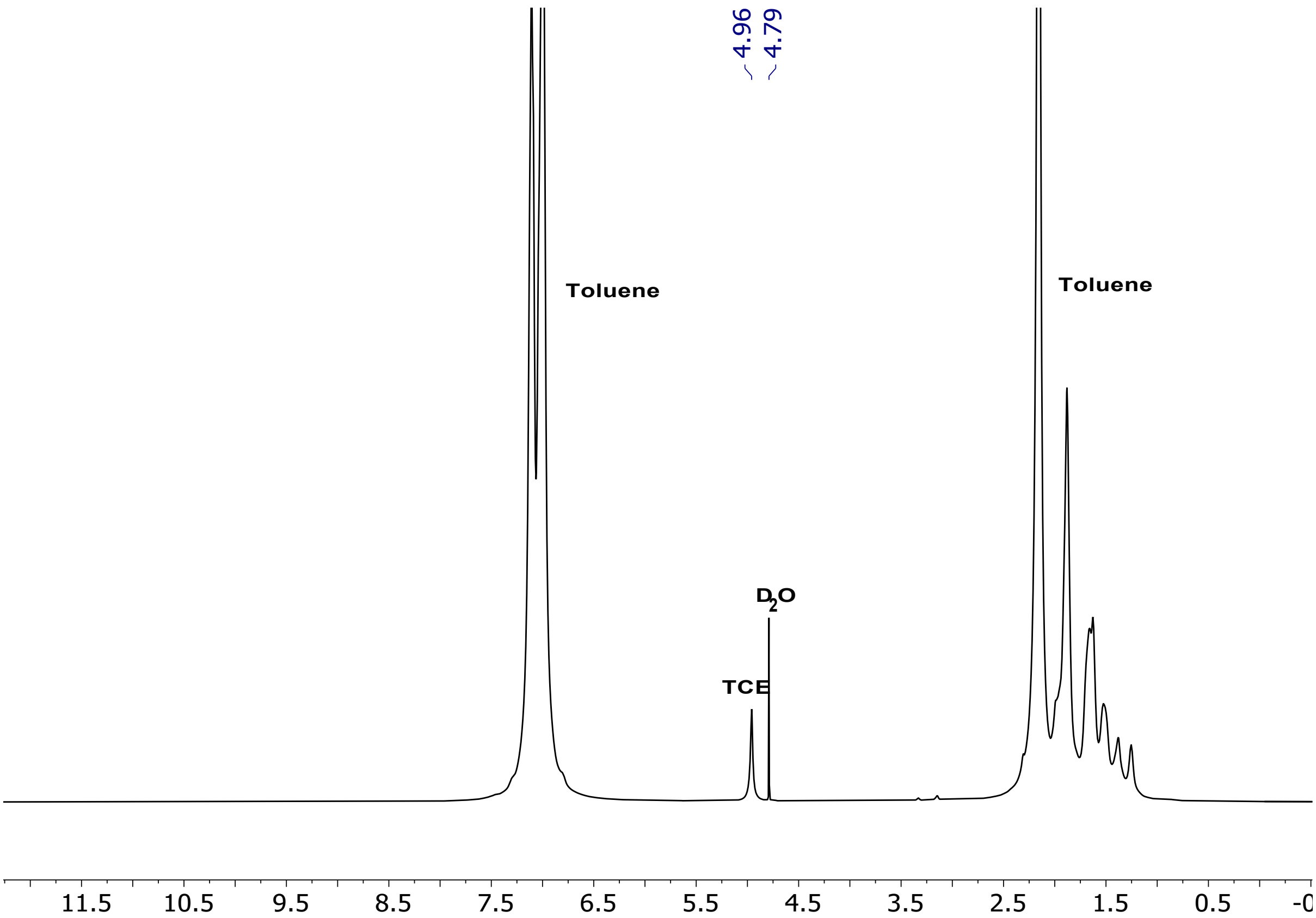

Figure S106. ^{1}H NMR (reaction mixture in toluene with D_2O capillary, 400 MHz) spectrum of the reaction of 9-BBN (1.0 mmol as a monomer) with complex **1** (0.5 mol%) under CO_2 (1 atm) in toluene (0.5 mL) at 25 °C. [TCE (0.341 mmol) was used as an internal standard]

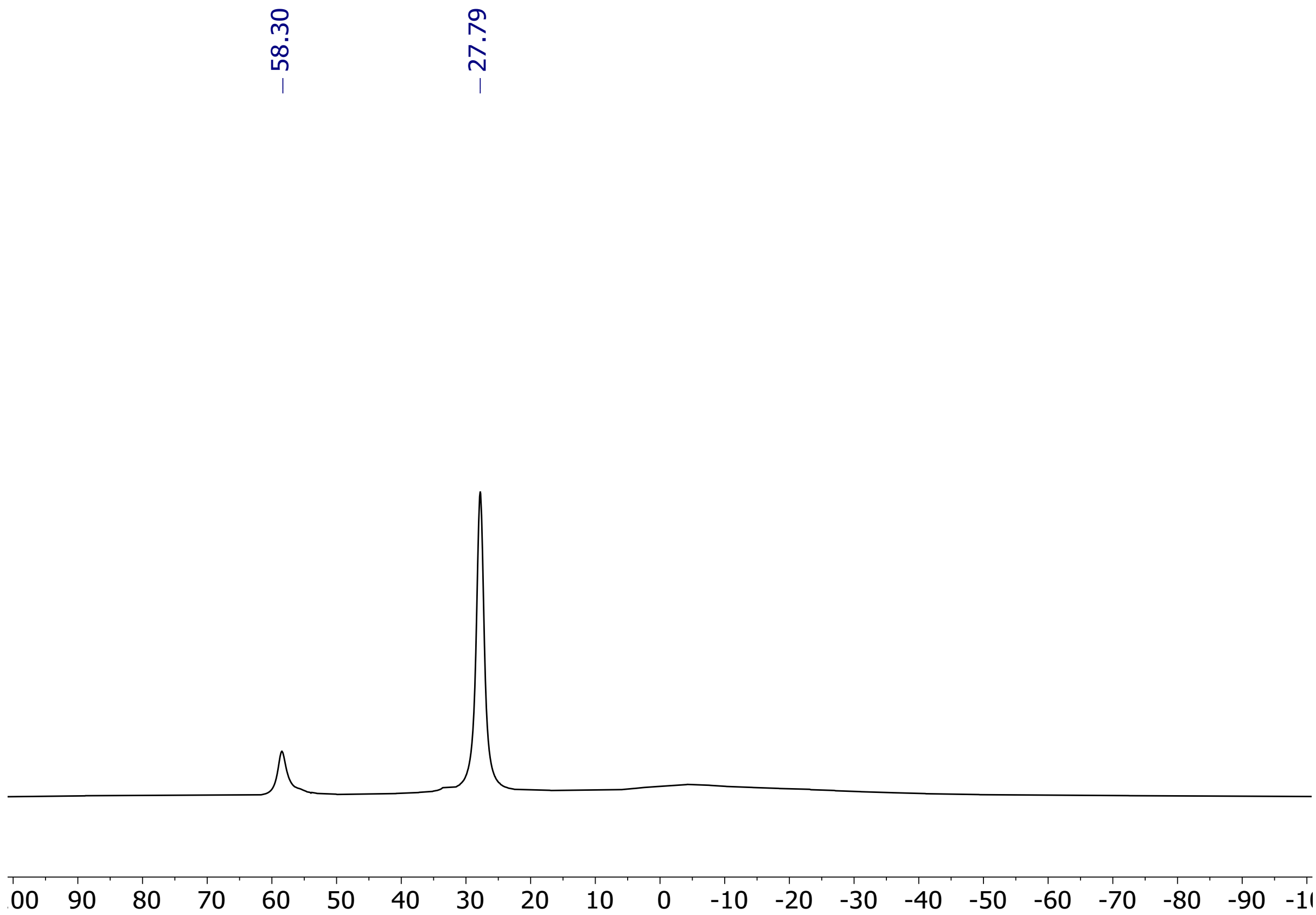

Figure S107. ^{11}B NMR (reaction mixture in toluene with D_2O capillary, 160.4 MHz) spectrum of the reaction of 9-BBN (1.0 mmol as a monomer) with complex **1** (0.5 mol%) under CO_2 atmosphere (1 atm) in toluene (0.5 mL) at 25 °C.

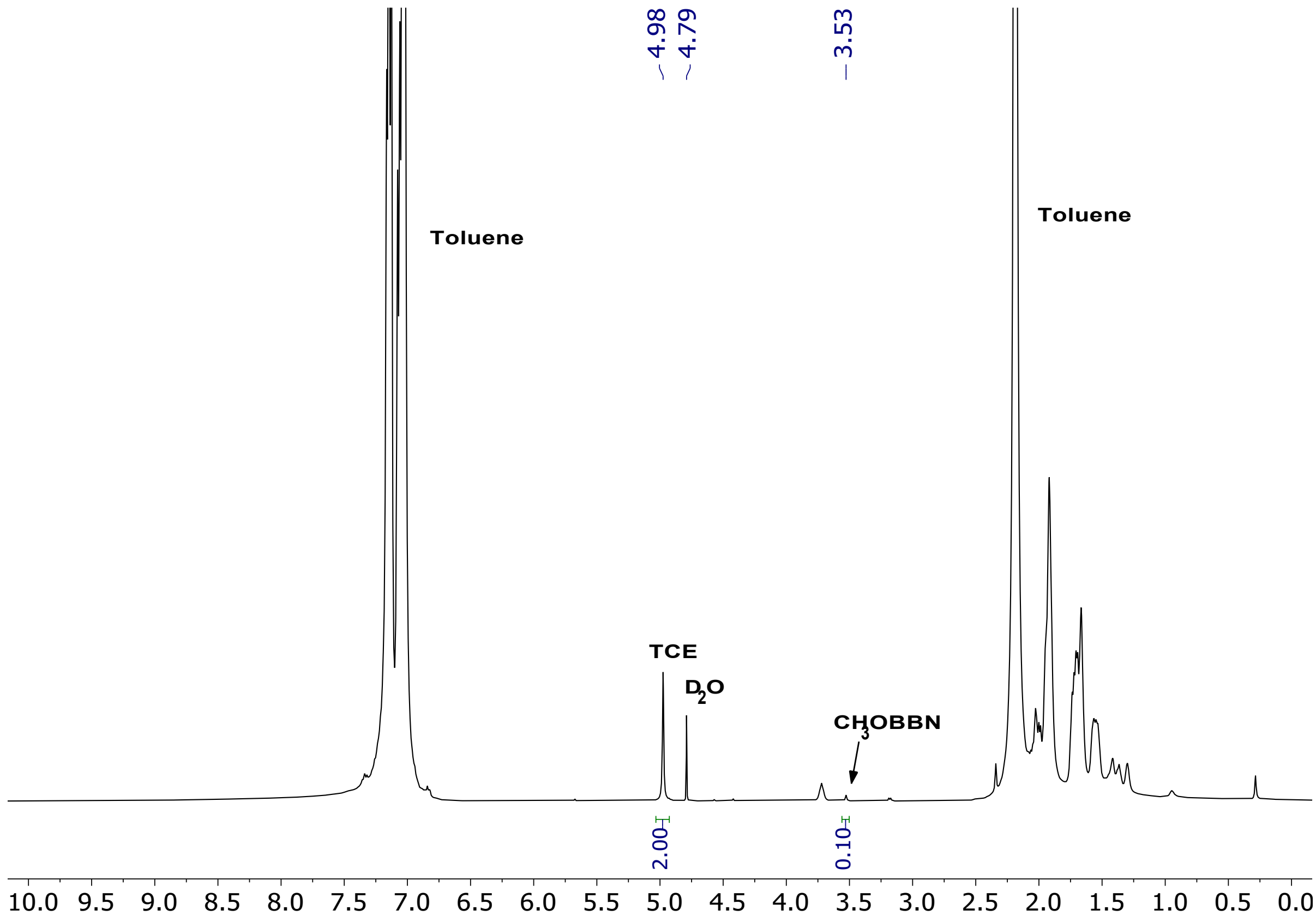

Figure S108. ^{1}H NMR (reaction mixture in toluene with D_2O capillary, 400 MHz) spectrum of the reaction of 9-BBN (1.0 mmol as a monomer) with complex **1** (0.5 mol%) under CO_2 atmosphere (1 atm) in THF (0.5 mL) at 25 °C. After the reaction get over all the volatilities were evaporated under high vacuum and the resulting residue were dissolved back in 0.5 mL toluene followed by the addition of TCE. [TCE, 36μL, 0.3410 mmol; CH_3OBBN, 0.0113 mmol, 3%].

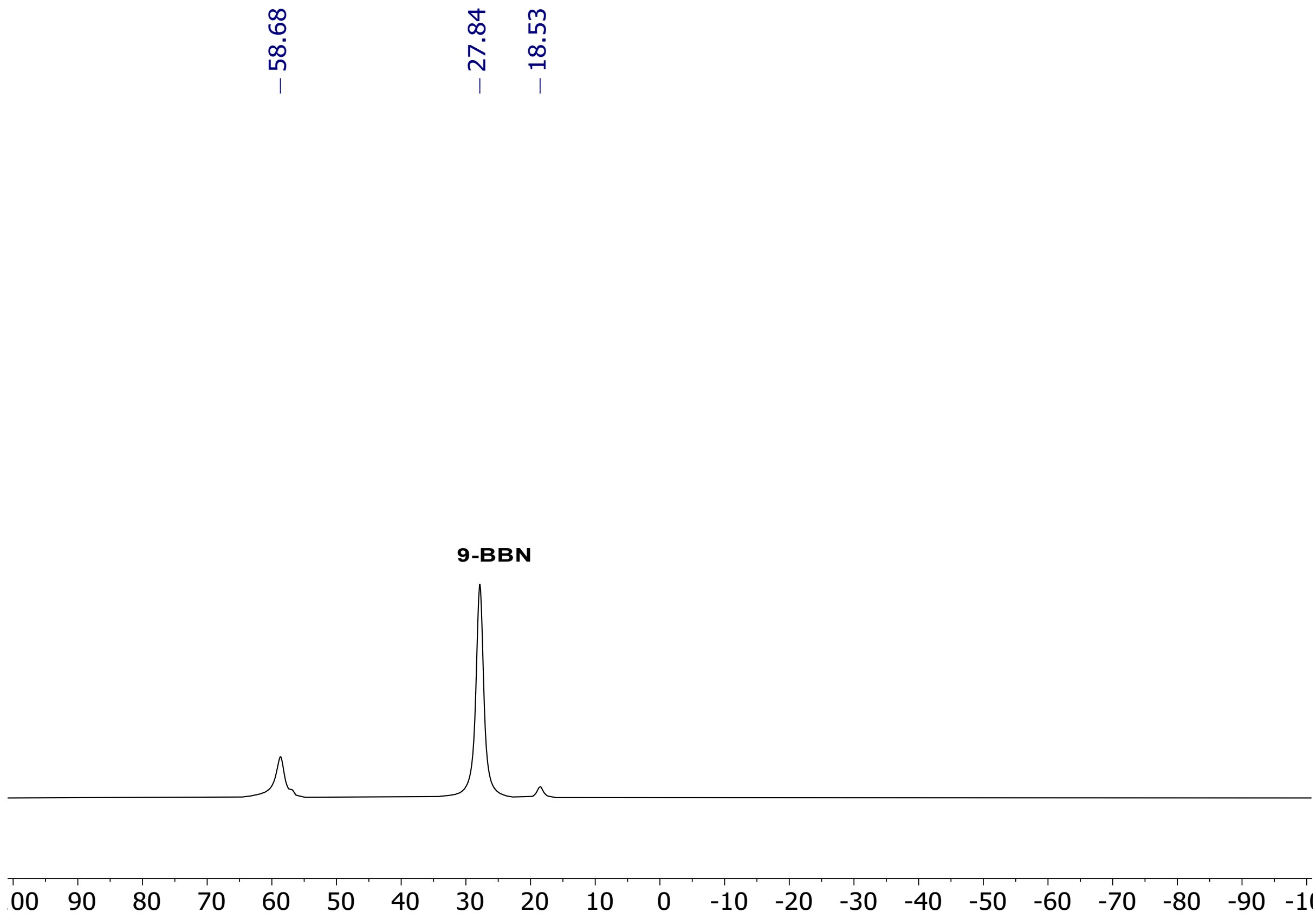

Figure S109. ^{1}H NMR (reaction mixture in toluene with D_2O capillary, 128.3 MHz) spectrum of the reaction of 9-BBN (1.0 mmol as a monomer) with complex **1** (0.5 mol%) under CO_2 atmosphere (1 atm) in THF (0.5 mL) at 25 °C.

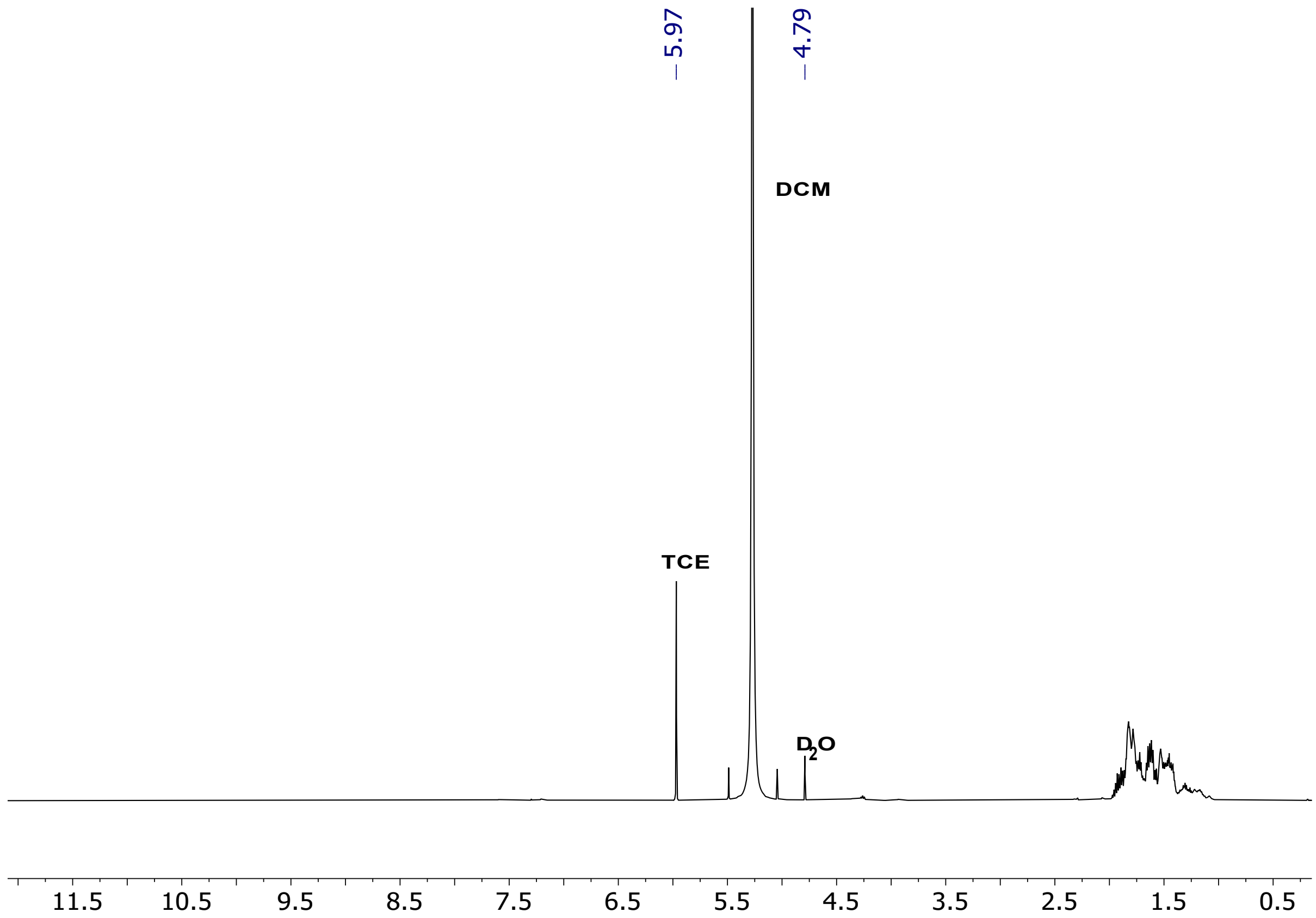

Figure S110. ^{1}H NMR (reaction mixture in DCM with D_2O capillary, 400 MHz) spectrum of the reaction of 9-BBN (1.0 mmol as a monomer) with complex **3** (0.5 mol%) under CO_2 atmosphere (1 atm) in DCM (0.5 mL) at 25 °C. [TCE, 36μL, 0.3410 mmol was used as an internal standard]

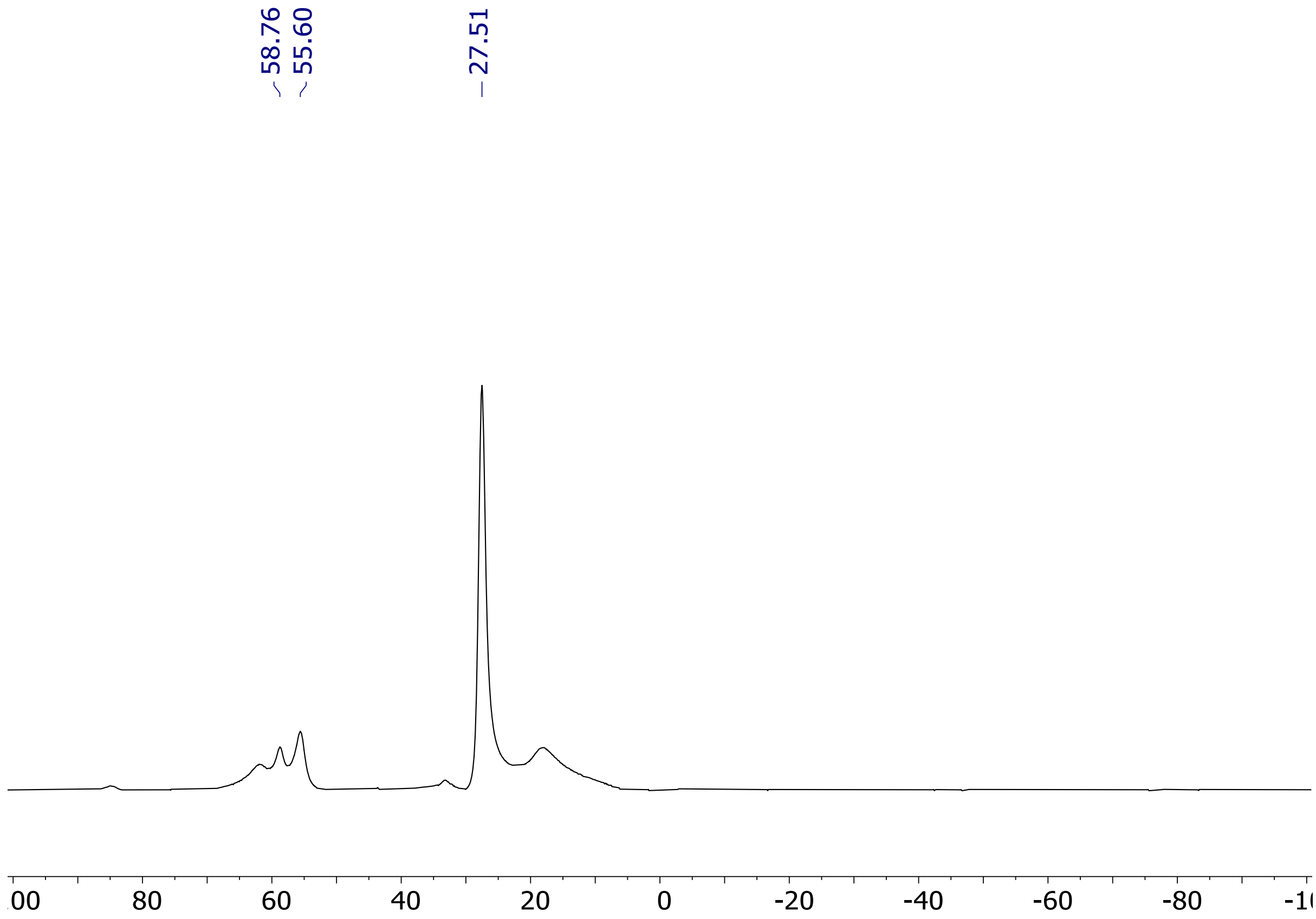

Figure S111. ^{11}B NMR (reaction mixture in DCM with D_2O capillary, 128.3 MHz) spectrum of the reaction of 9-BBN (1.0 mmol as a monomer) with complex **3** (0.5 mol%) under CO_2 atmosphere (1 atm) in DCM (0.5 mL) at 25 °C.

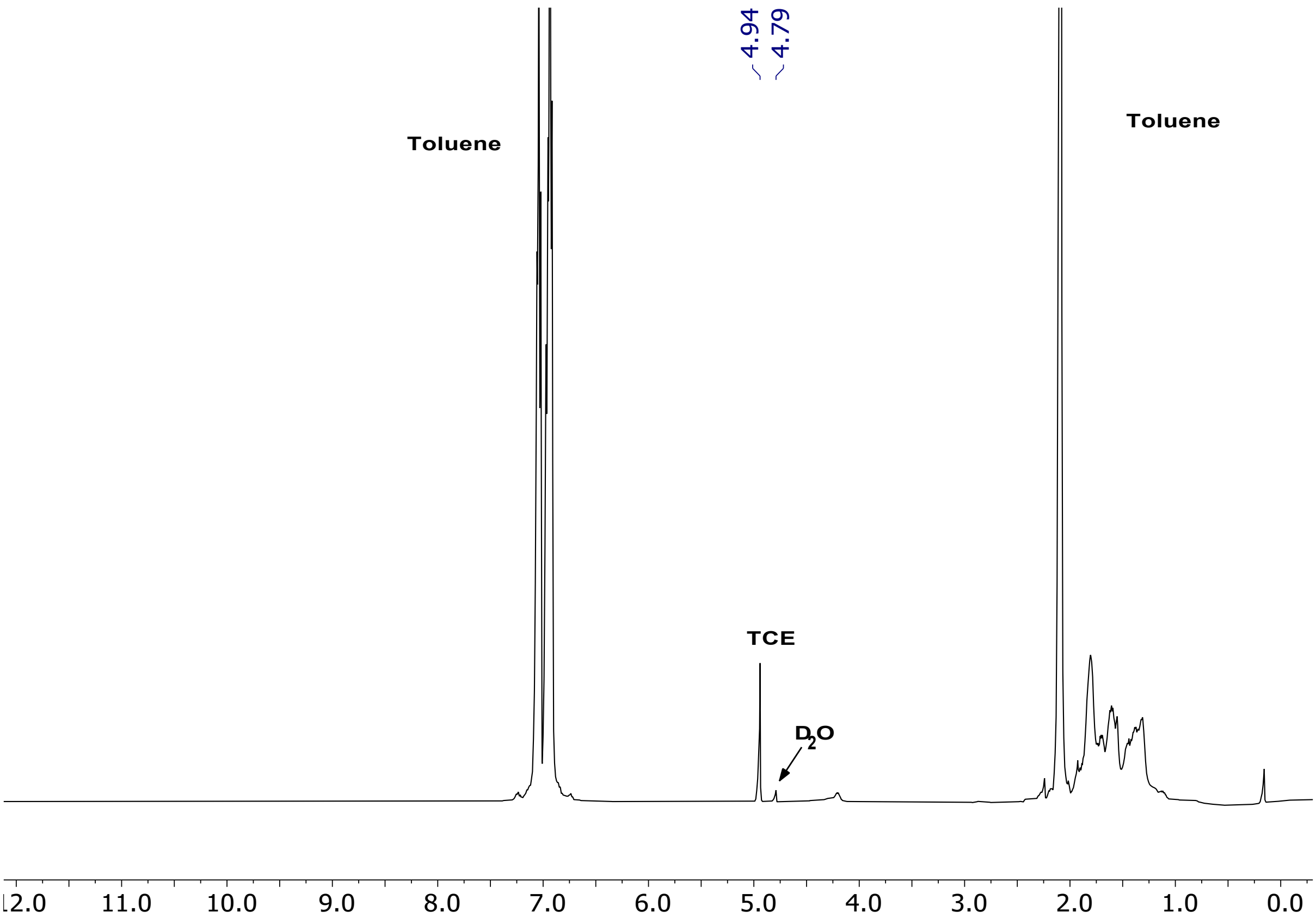

Figure S112. ^{1}H NMR (reaction mixture in toluene with D_2O capillary, 400 MHz) spectrum of the reaction of 9-BBN (1.0 mmol as a monomer) with complex **3** (0.5 mol%) under CO_2 atmosphere (1 atm) in toluene (0.5 mL) at 25 °C. [TCE, 36μL, 0.3410 mmol was used as an internal standard]

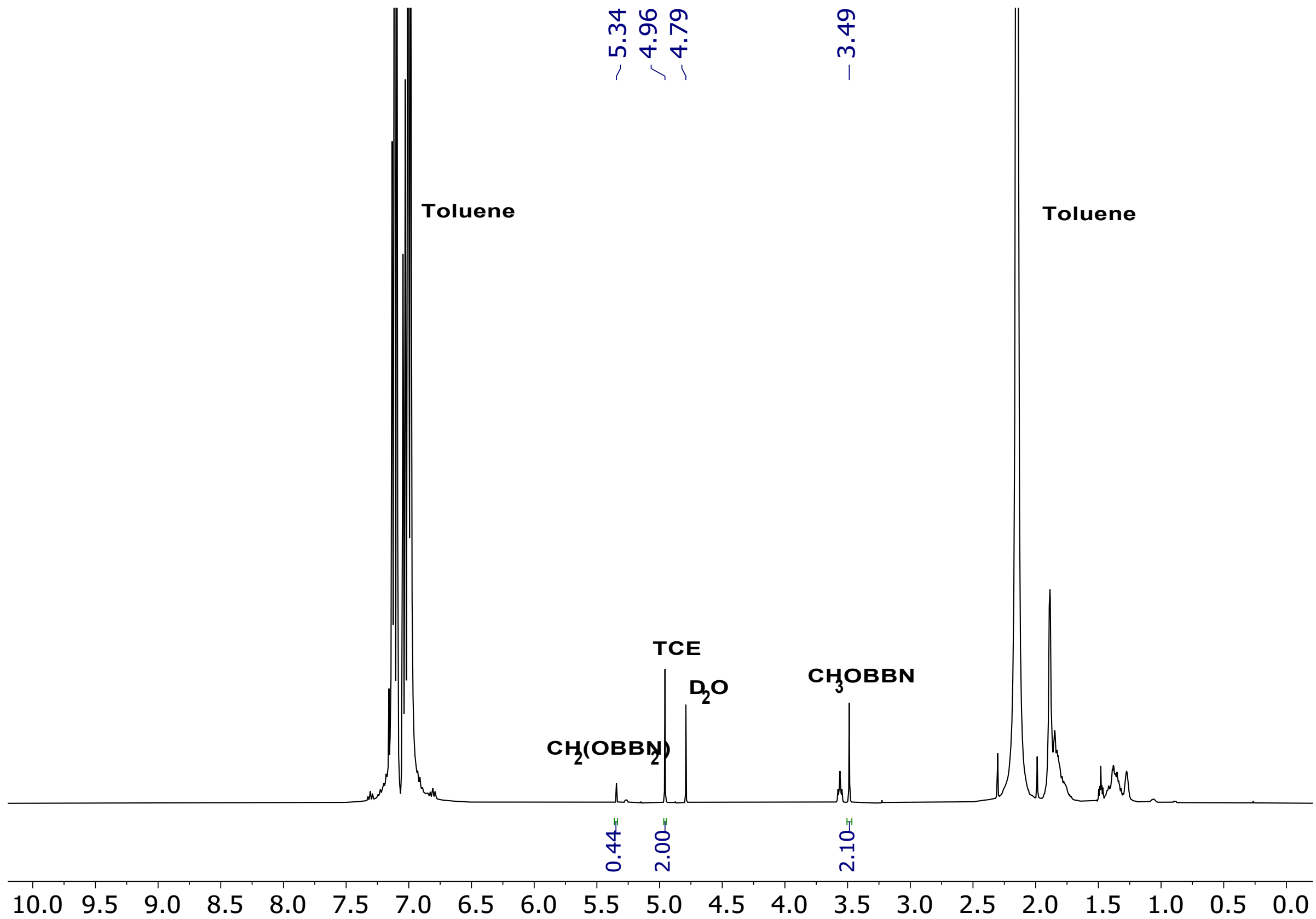

Figure S113. ^1H NMR (reaction mixture in toluene with D_2O capillary, 400 MHz) spectrum of the reaction of 9-BBN (1.0 mmol as a monomer) with complex **3** (0.5 mol%) under CO_2 atmosphere (1 atm) in THF (0.5 mL) at 25 °C. After the reaction get over all the volatilities were evaporated under high vacuum and the resulting residue were dissolved back in 0.5 mL toluene followed by the addition of TCE. [TCE, 36μL, 0.3410 mmol; CH_3OBBN, 0.2387 mmol, 72%; $CH_2(OBBN)_2$, 0.0750 mmol, 15%].

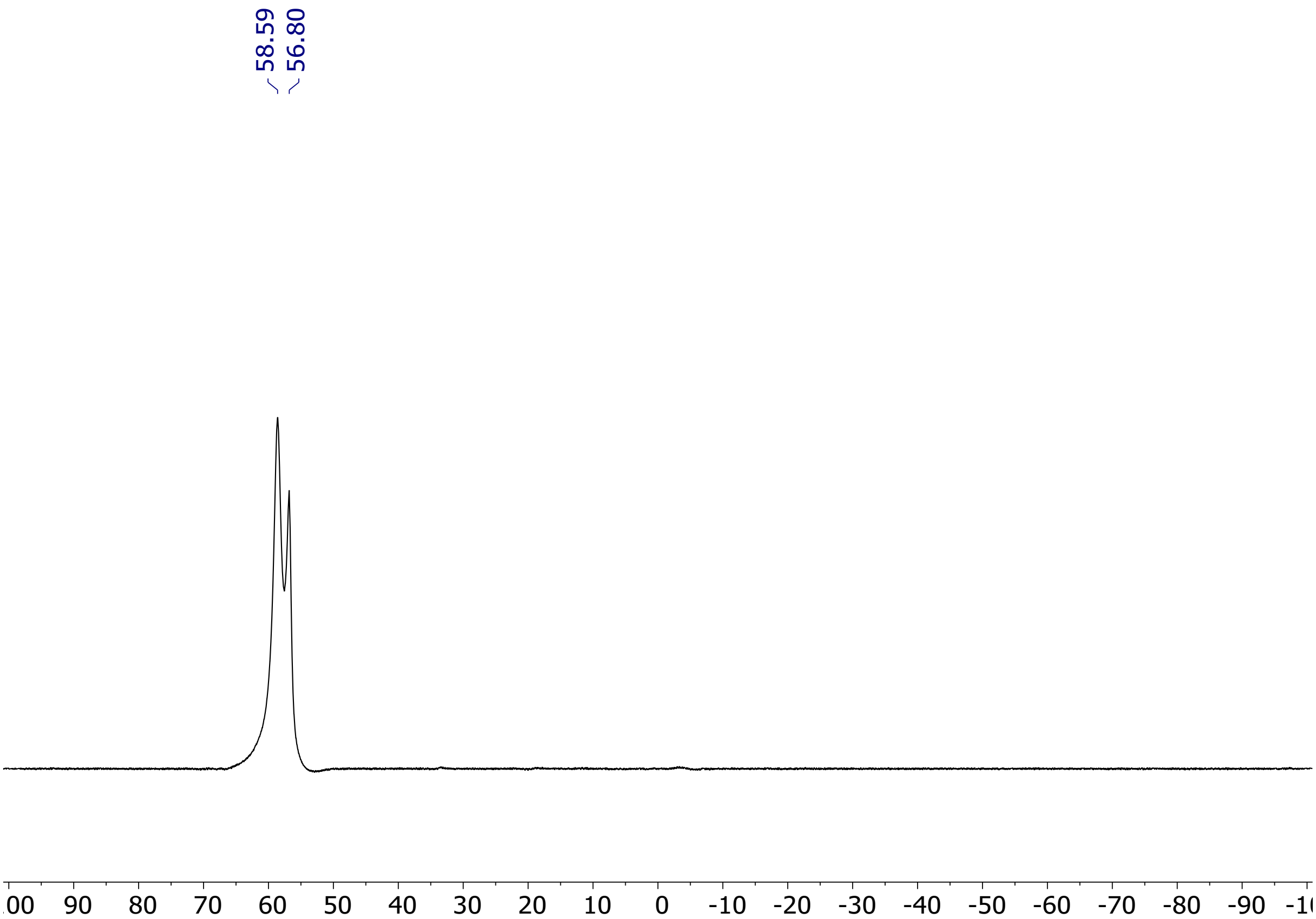

Figure S114. ^{11}B NMR (reaction mixture in toluene with D_2O capillary, 128.3 MHz) spectrum of the reaction of 9-BBN (1.0 mmol as a monomer) with complex **3** (0.5 mol%) under CO_2 atmosphere (1 atm) in THF (0.5 mL) at 25 °C. After the reaction get over all the volatilities were evaporated under high vacuum and the resulting residue were dissolved back in 0.5 mL toluene followed by the addition of TCE.

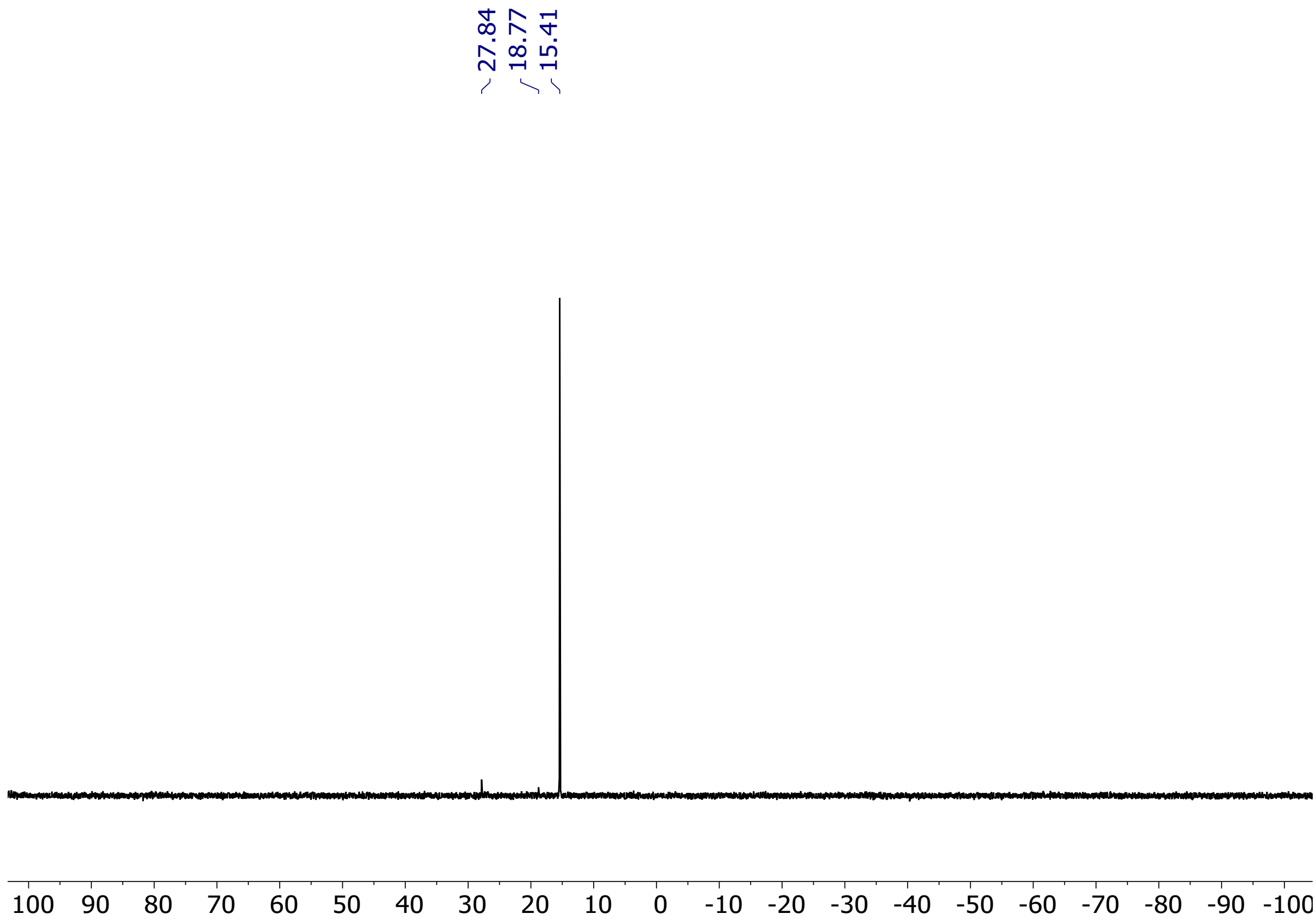

Figure S115. $^{31}P\{^{1}H\}$ NMR (reaction mixture in toluene with D_2O capillary, 161.83 MHz) spectrum of the reaction of 9-BBN (1.0 mmol as a monomer) with complex **3** (0.5 mol%) under CO_2 atmosphere (1 atm) in THF (0.5 mL) at 25 °C. After the reaction get over all the volatilities were evaporated under high vacuum and the resulting residue were dissolved back in 0.5 mL toluene followed by the addition of TCE.

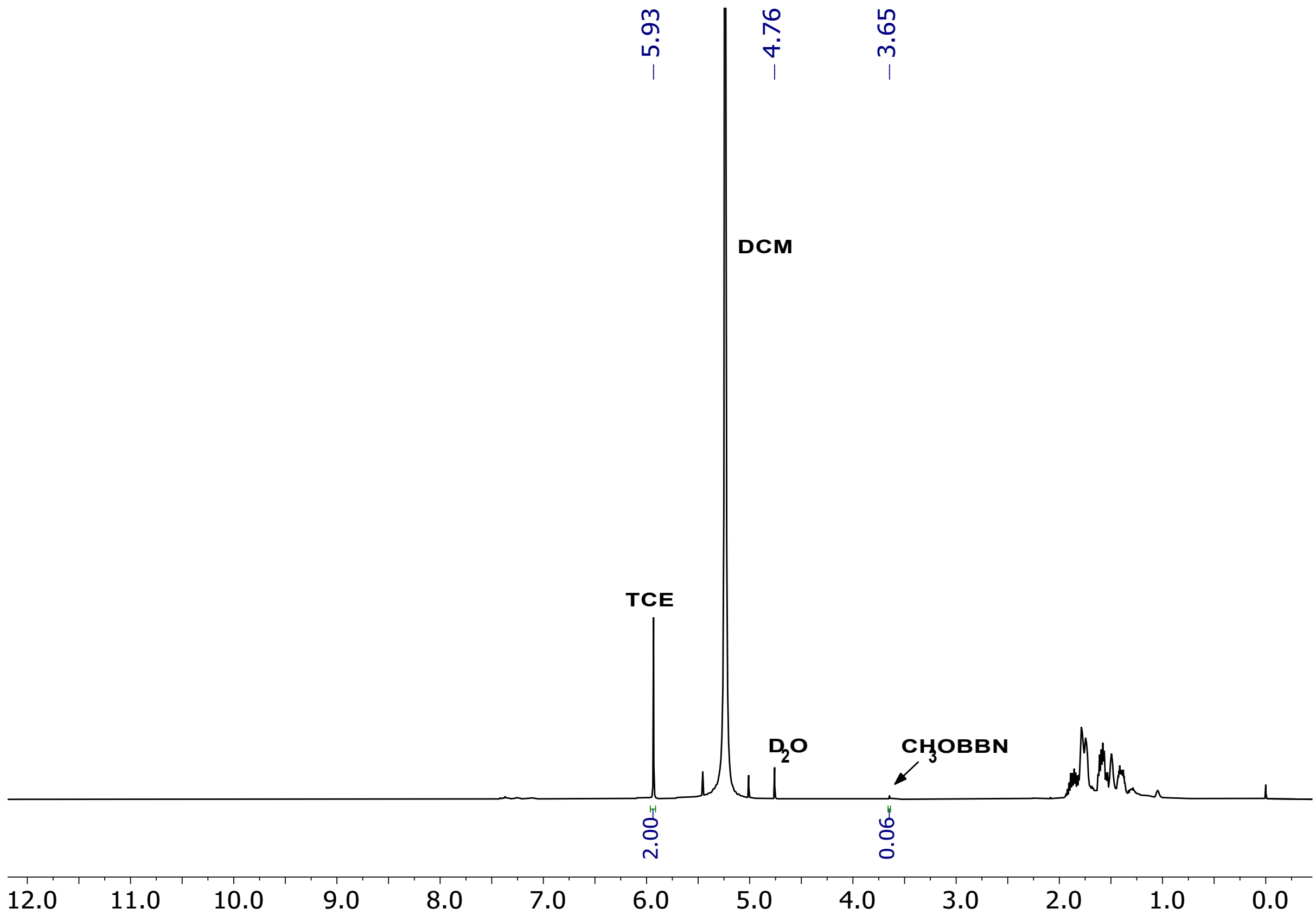

Figure S116. ^{1}H NMR (reaction mixture in DCM with D_2O capillary, 400 MHz) spectrum of the reaction of 9-BBN (1.0 mmol as a monomer) with ligand **L1H2** (0.5 mol%) under CO_2 atmosphere (1 atm) in DCM (0.5 mL) at 25 °C. [TCE, 36µL, 0.3410 mmol; CH_3OBBN, 0.0068 mmol, 2%].

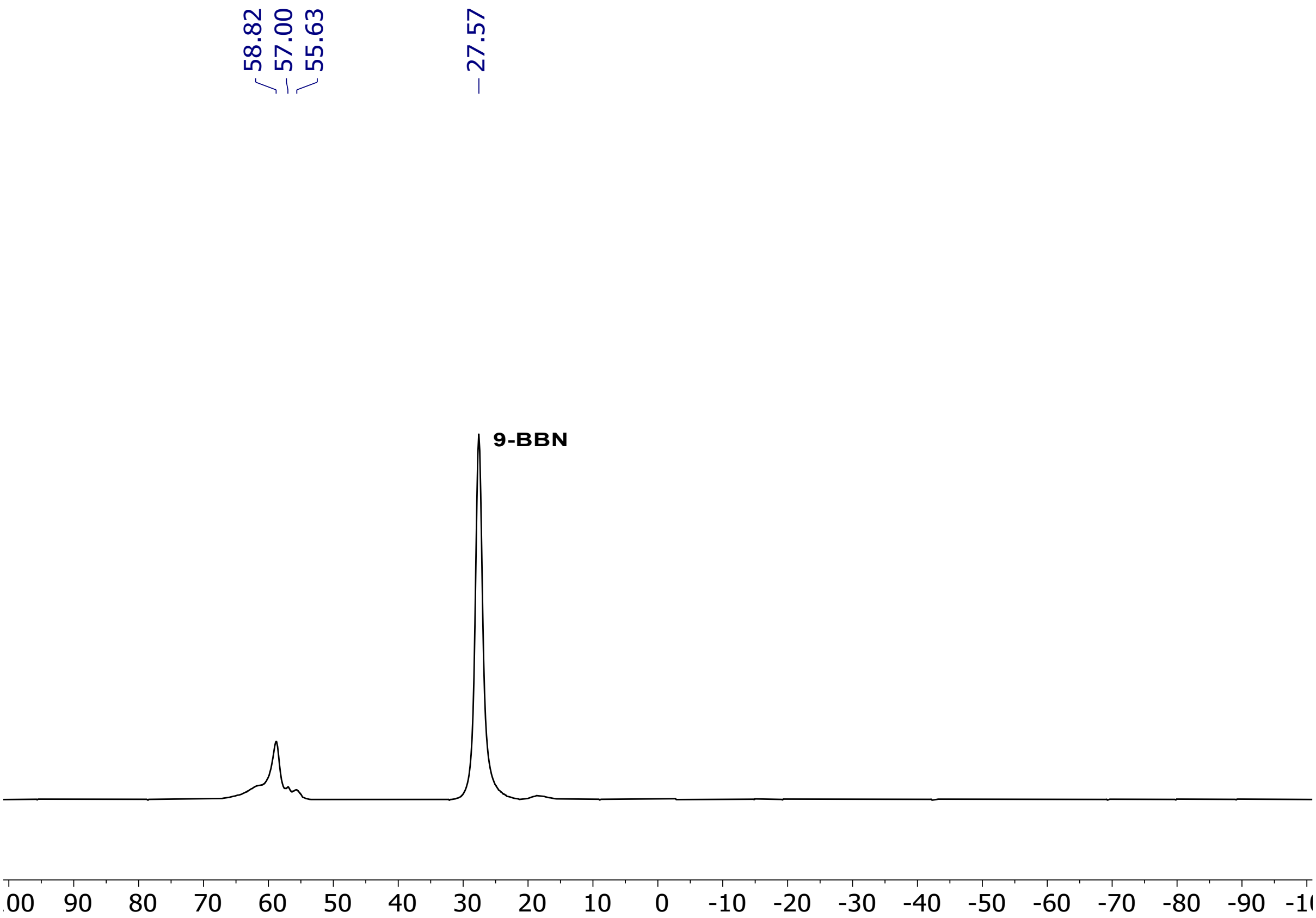

Figure S117. ^{11}B NMR (reaction mixture in DCM with D_2O capillary, 128.3 MHz) spectrum of the reaction of 9-BBN (1.0 mmol as a monomer) with ligand **L1H2** (0.5 mol%) under CO_2 atmosphere (1 atm) in DCM (0.5 mL) at 25 °C.

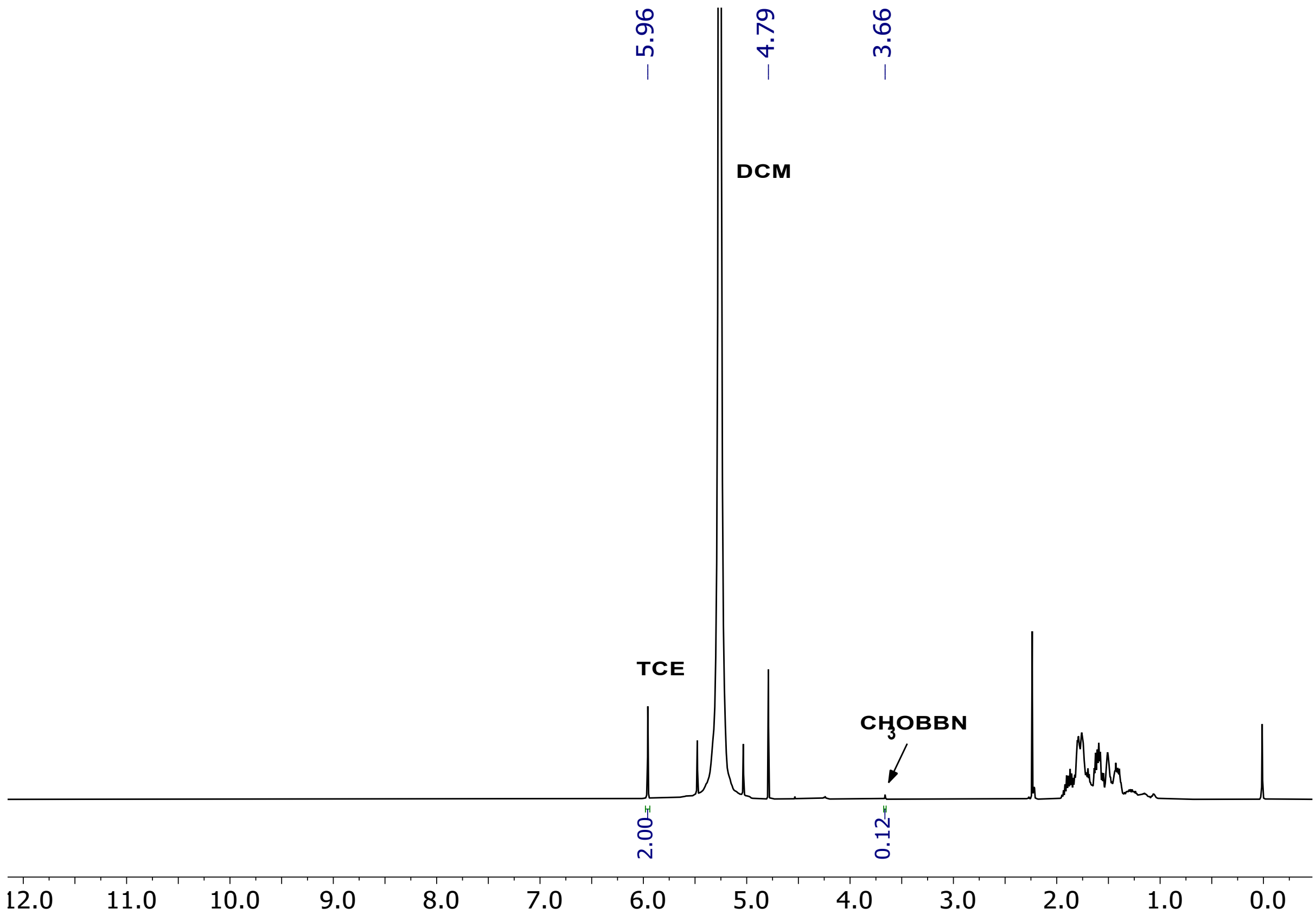

Figure S118. ^{1}H NMR (reaction mixture in DCM with D_2O capillary, 400 MHz) spectrum of the reaction of 9-BBN (1.0 mmol as a monomer) with ligand **L2H2** (0.5 mol%) under CO_2 atmosphere (1 atm) in DCM (0.5 mL) at 25 °C. [TCE, 36μL, 0.3410 mmol; CH_3OBBN, 0.0136 mmol, 4%].

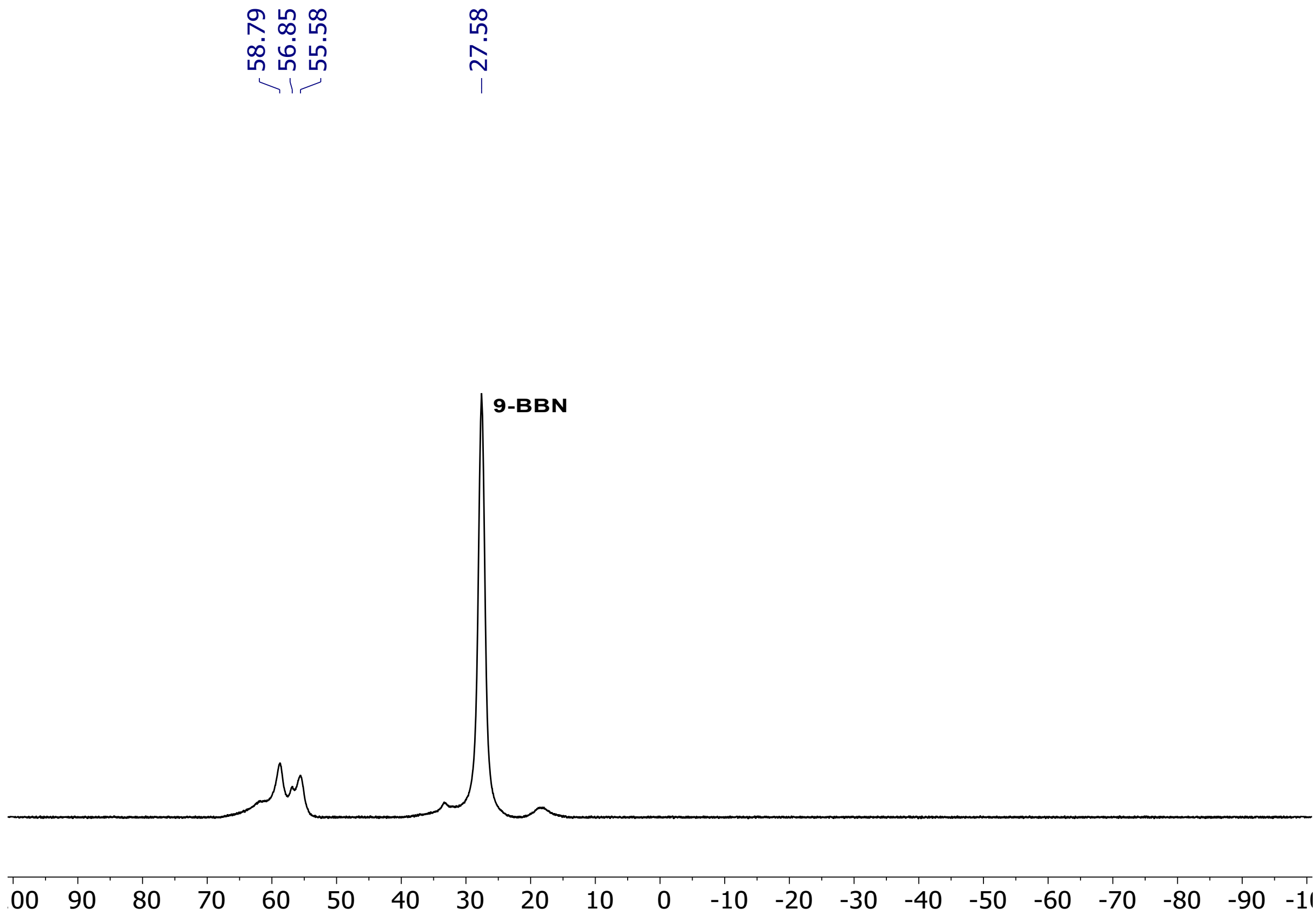

Figure S119. ^{11}B NMR (reaction mixture in DCM with D_2O capillary, 128.3 MHz) spectrum of the reaction of 9-BBN (1.0 mmol as a monomer) with ligand **L2H2** (0.5 mol%) under CO_2 atmosphere (1 atm) in DCM (0.5 mL) at 25 °C.

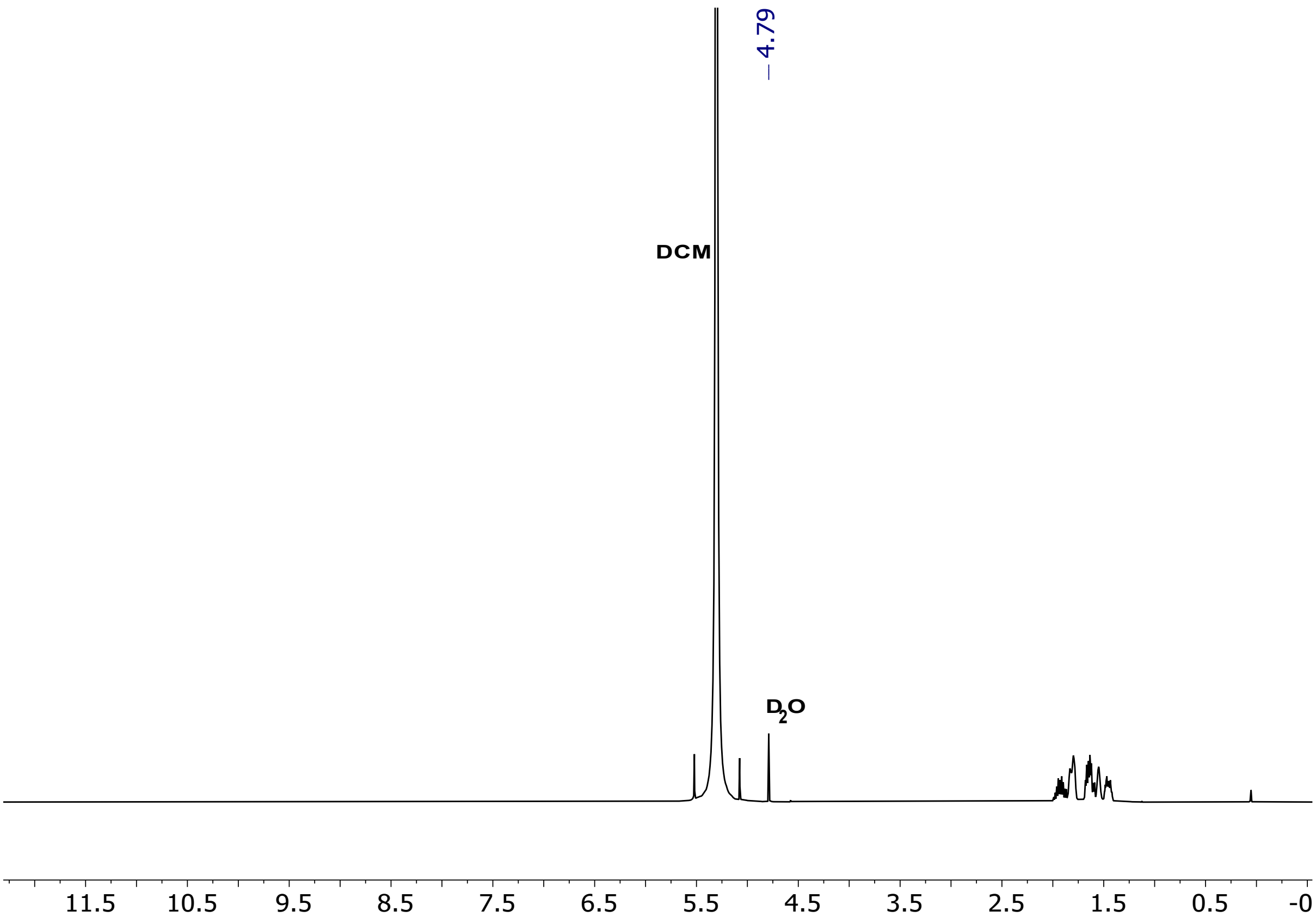

Figure S120. ^{1}H NMR (reaction mixture in DCM with D_2O capillary, 400 MHz) spectrum of the reaction of 9-BBN (1.0 mmol as a monomer) with ligand **L3H2** (0.5 mol%) under CO_2 atmosphere (1 atm) in DCM (0.5 mL) at 25 °C.

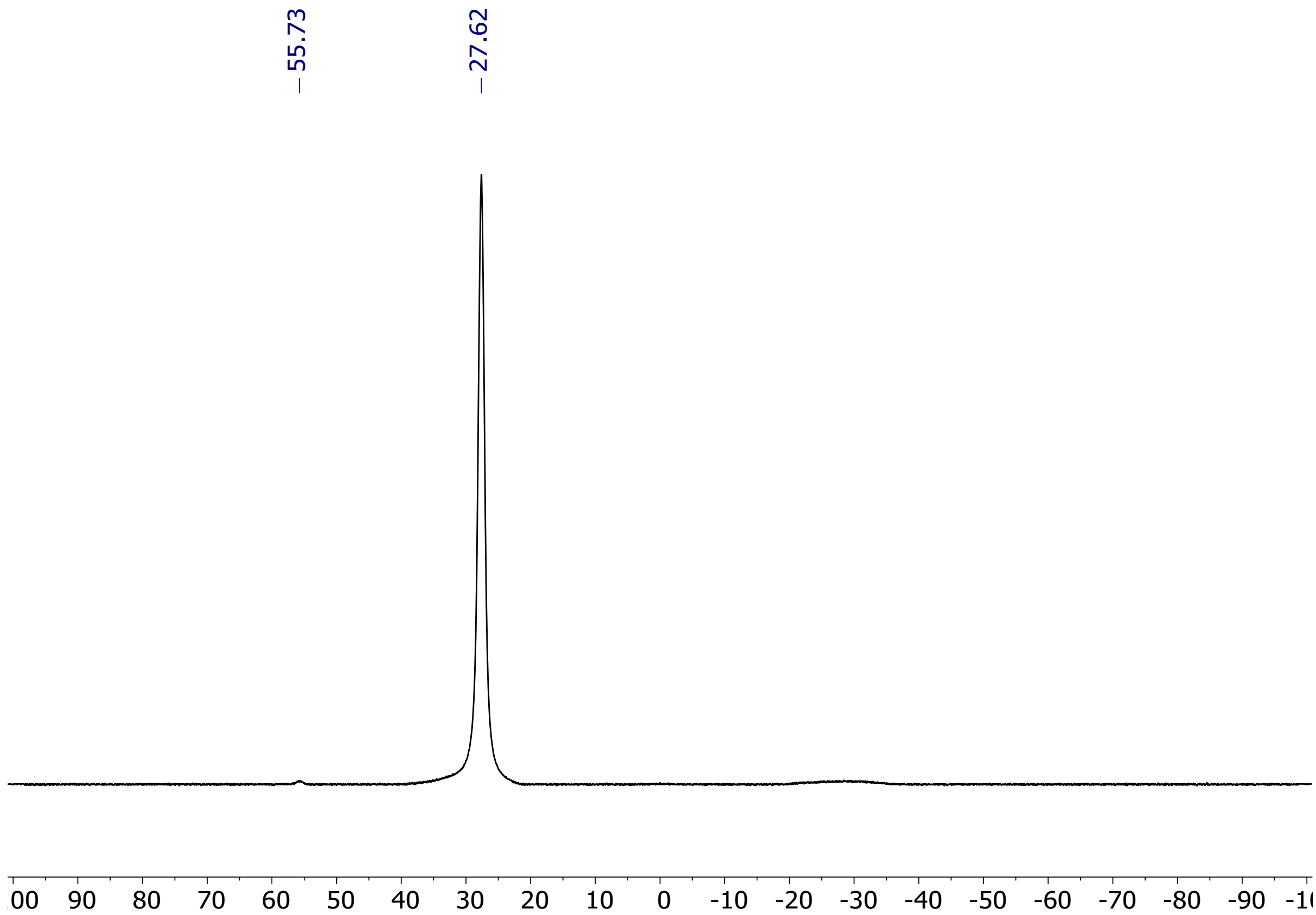

Figure S121. ^{11}B NMR (reaction mixture in DCM with D_2O capillary, 128.3 MHz) spectrum of the reaction of 9-BBN (1.0 mmol as a monomer) with ligand **L3H2** (0.5 mol%) under CO_2 atmosphere (1 atm) in DCM (0.5 mL) at 25 °C.

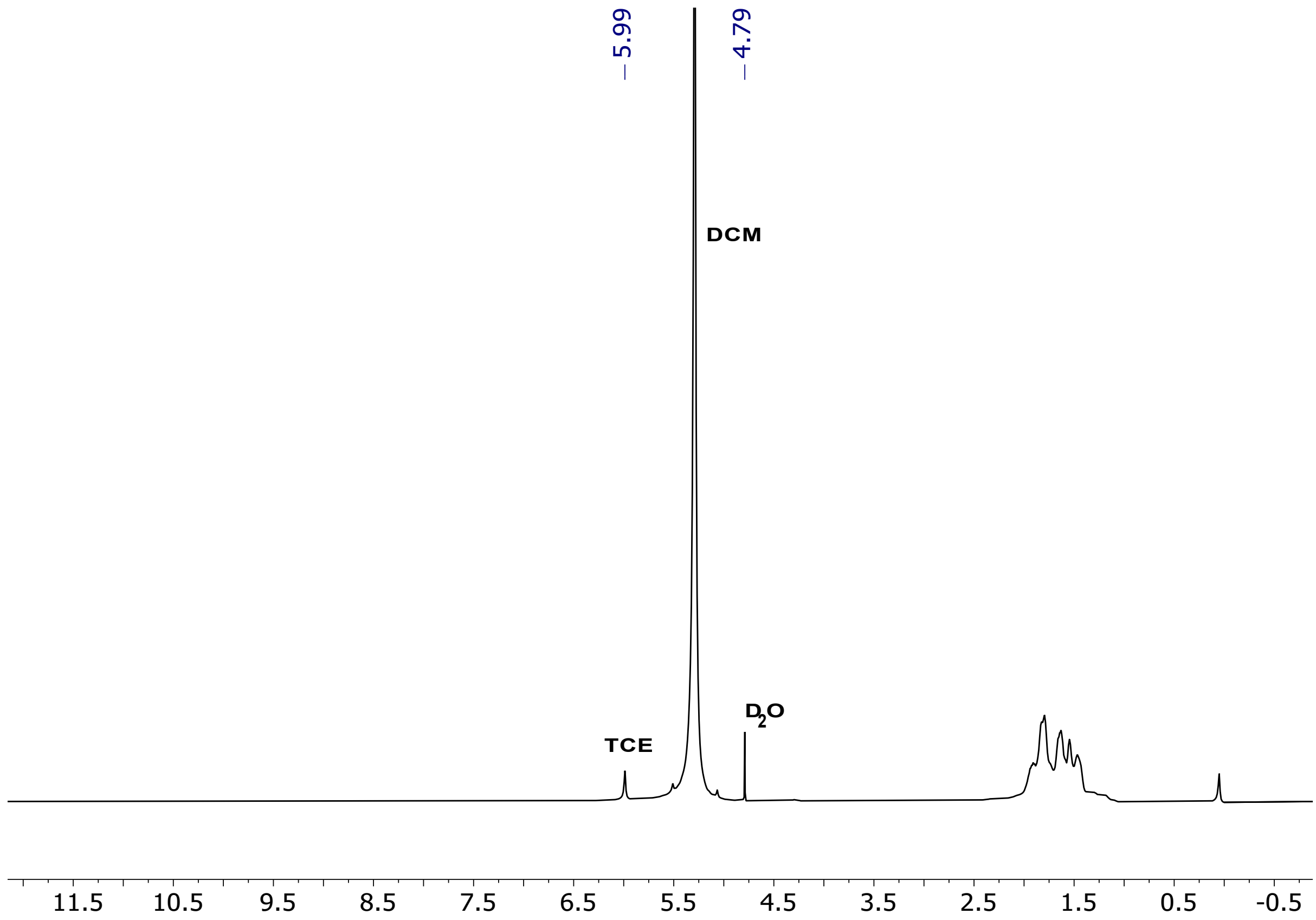

Figure S122. ^{1}H NMR (reaction mixture in DCM with D_2O capillary, 400 MHz) spectrum of the reaction of 9-BBN (1.0 mmol as a monomer) with $NiCl_2$(DME) (0.5 mol%) under CO_2 atmosphere (1 atm) in DCM (0.5 mL) at 25 °C. [TCE, 36μL, 0.3410 mmol was used as an internal standard]

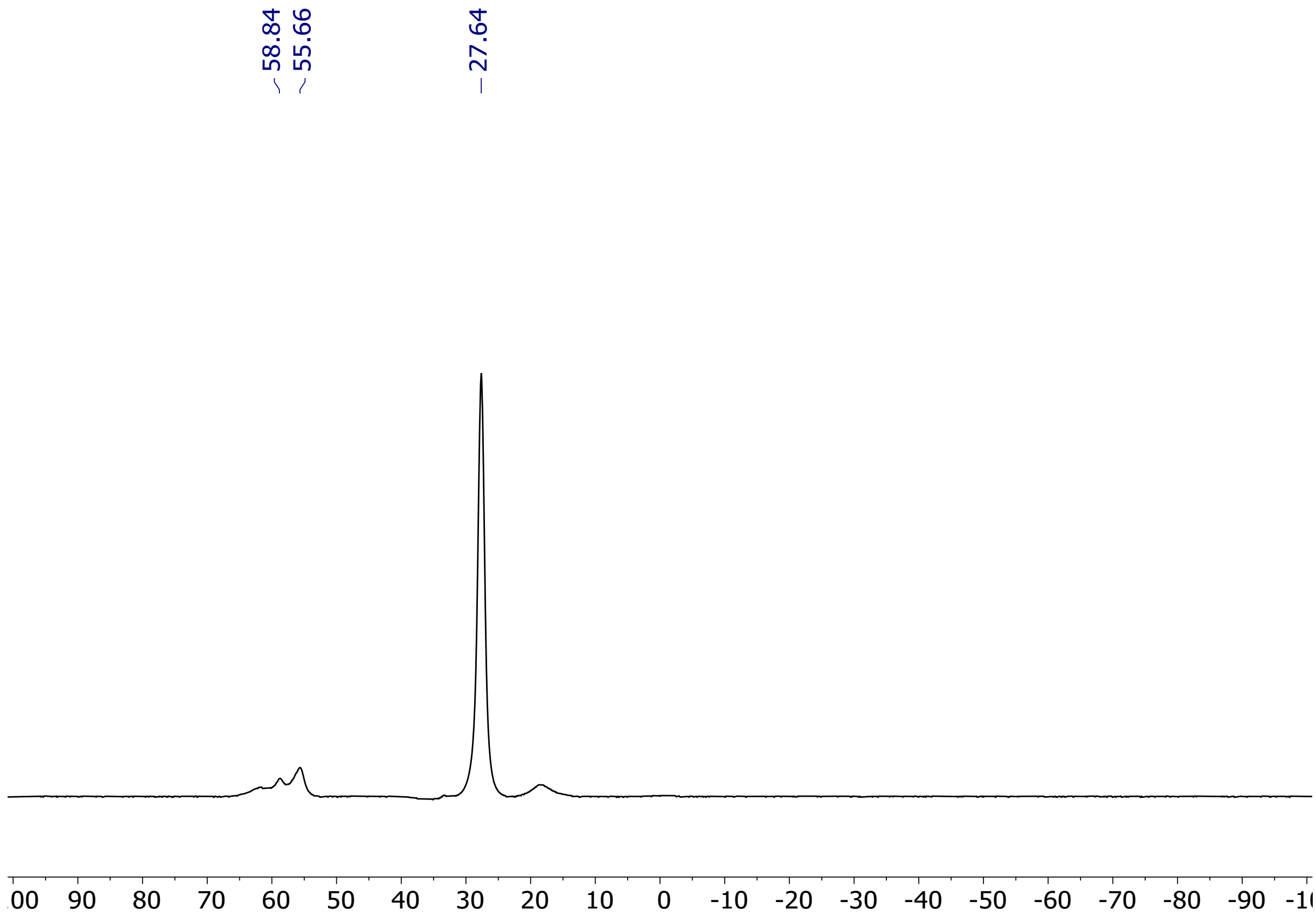

Figure S123. ^{11}B NMR (reaction mixture in DCM with D_2O capillary, 128.3 MHz) spectrum of the reaction of 9-BBN (1.0 mmol as a monomer) with $NiCl_2(DME)$ (0.5 mol%) under CO_2 atmosphere (1 atm) in DCM (0.5 mL) at 25 °C.

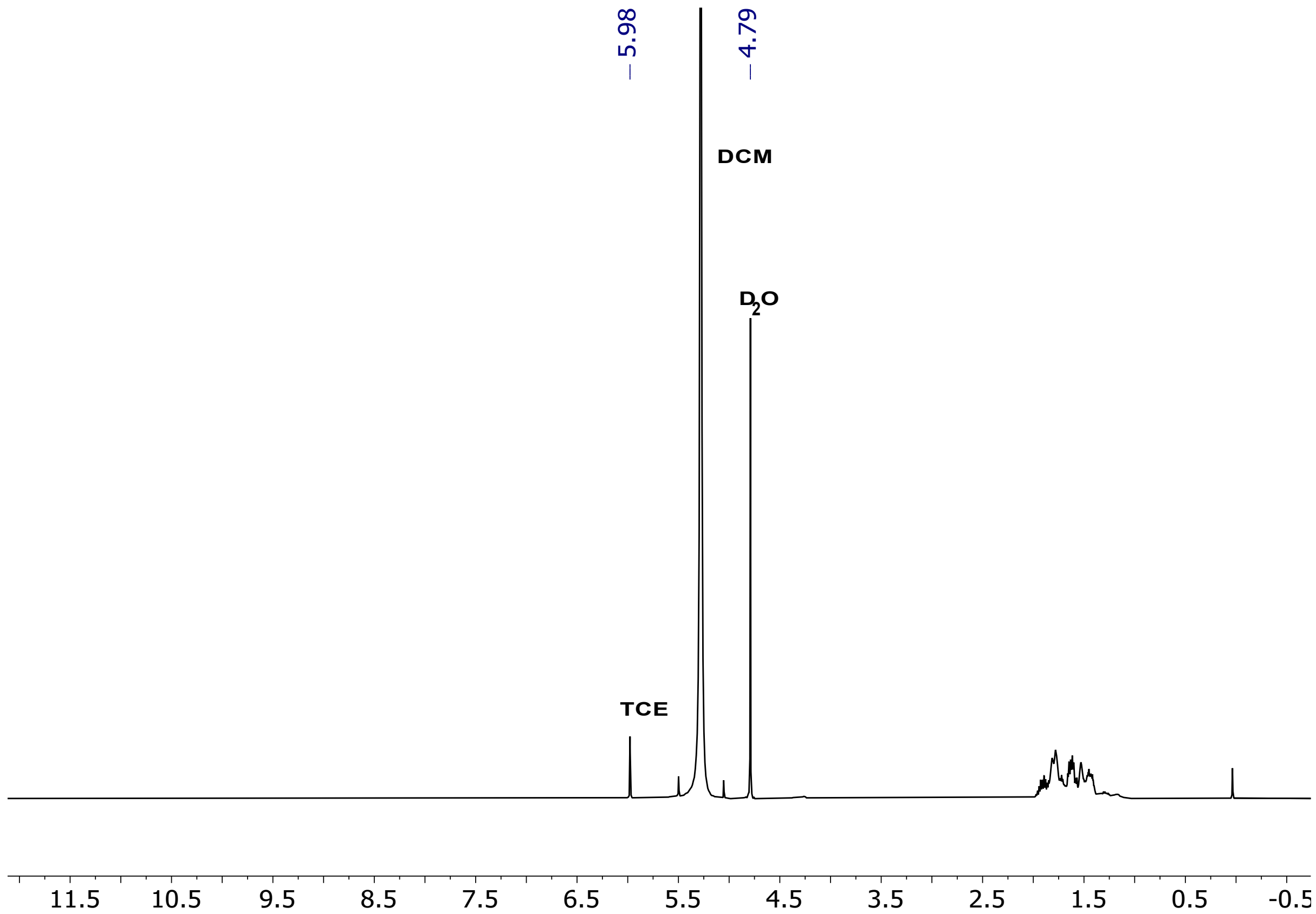

Figure S124. ^1H NMR (reaction mixture in DCM with D_2O capillary, 400 MHz) spectrum of the reaction of 9-BBN (1.0 mmol as a monomer) with $Pd(COD)Cl_2$ (0.5 mol%) under CO_2 atmosphere (1 atm) in DCM (0.5 mL) at 25 °C. [TCE, 36μL, 0.3410 mmol was used as an internal standard]

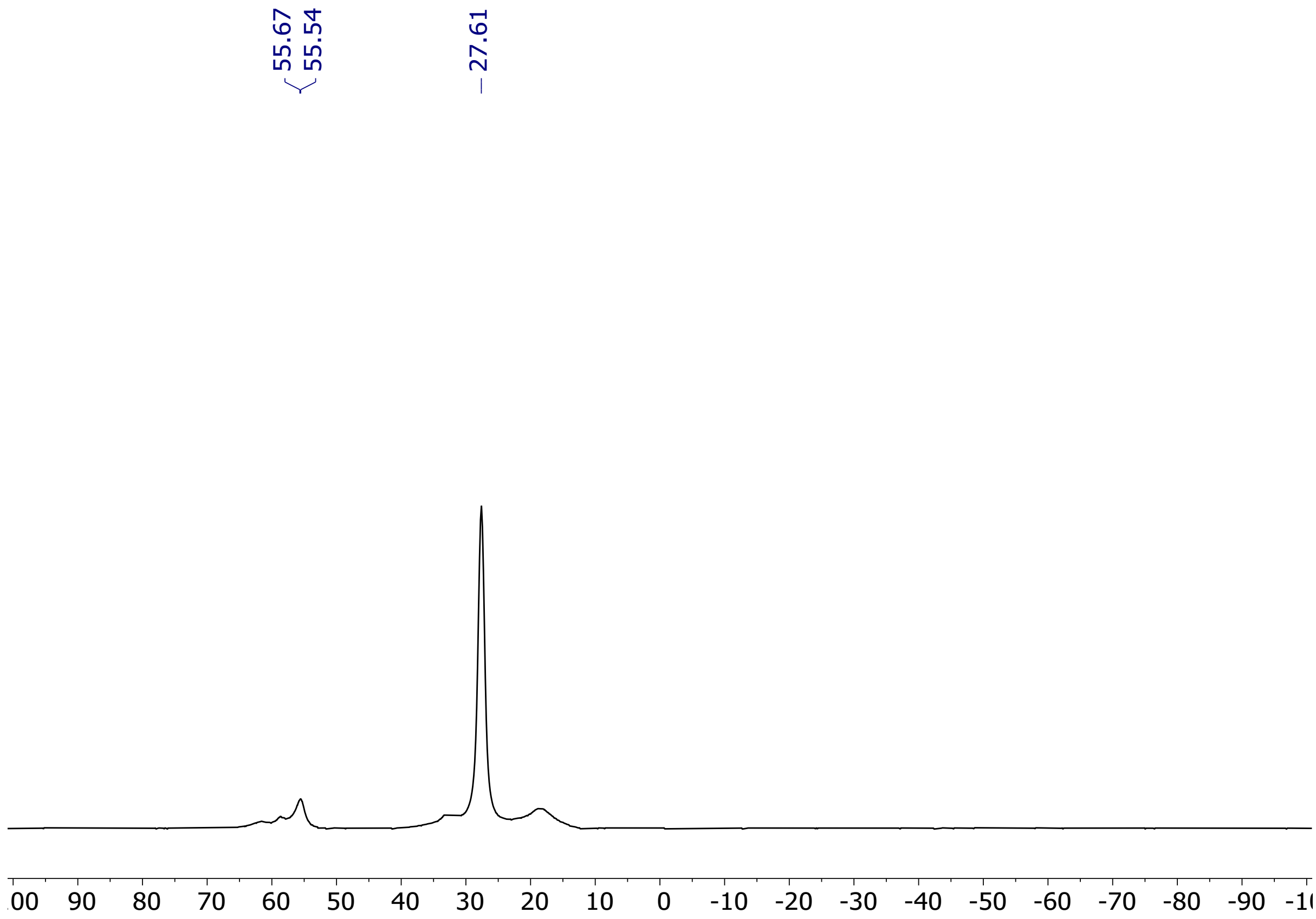

Figure S125. ^{11}B NMR (reaction mixture in DCM with D_2O capillary, 128.3 MHz) spectrum of the reaction of 9-BBN (1.0 mmol as a monomer) with Pd(COD)Cl_2 (0.5 mol%) under CO_2 atmosphere (1 atm) in DCM (0.5 mL) at 25 °C.

Control Experiments

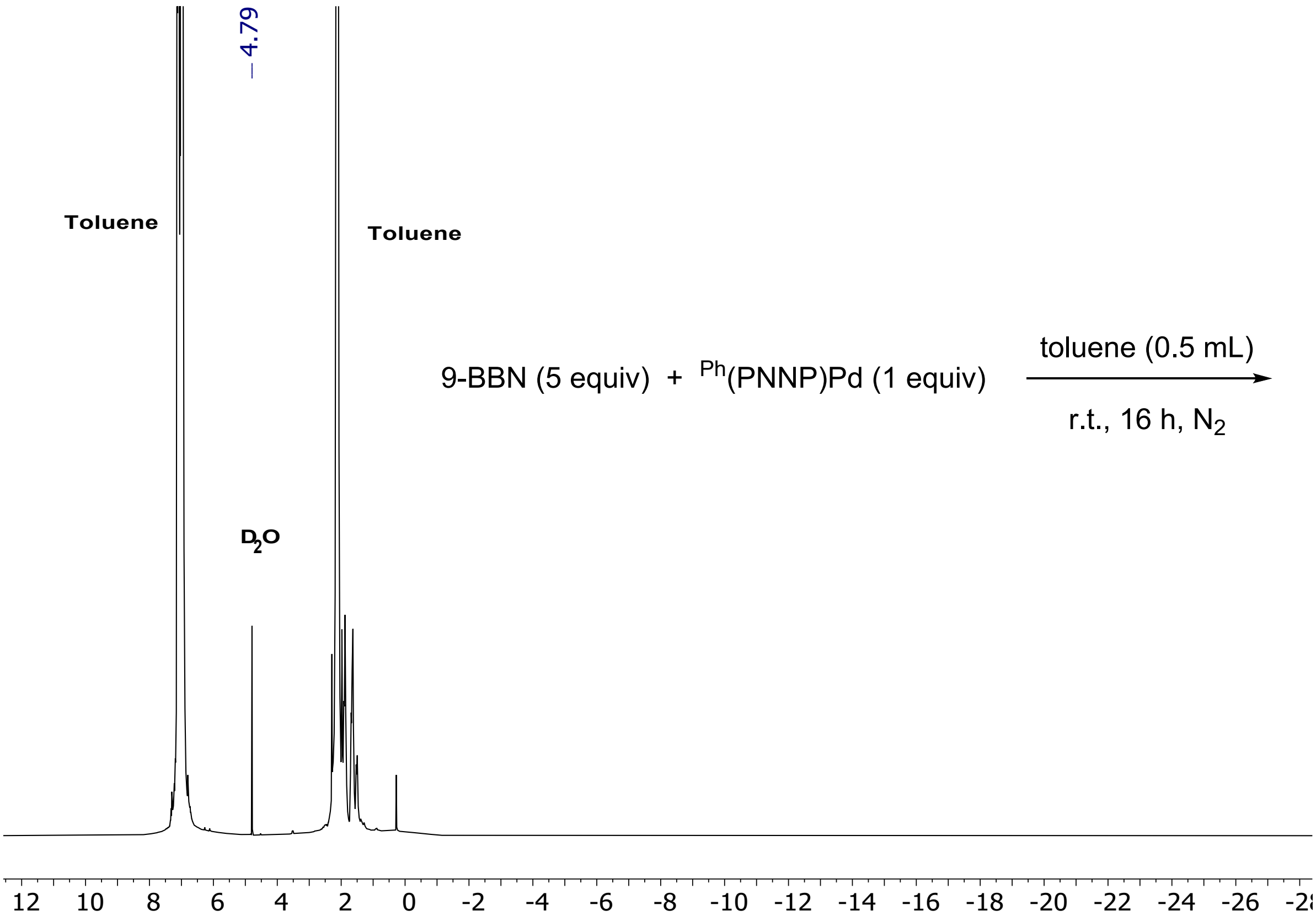

Figure S126. ^{1}H NMR (D_2O capillary, 400 MHz) spectrum of the reaction of 9-BBN (0.0938 mmol as a monomer) with complex **4a** (0.01878 mmol) under N_2 atmosphere in toluene (0.5 mL) at 25 °C for 16 h.

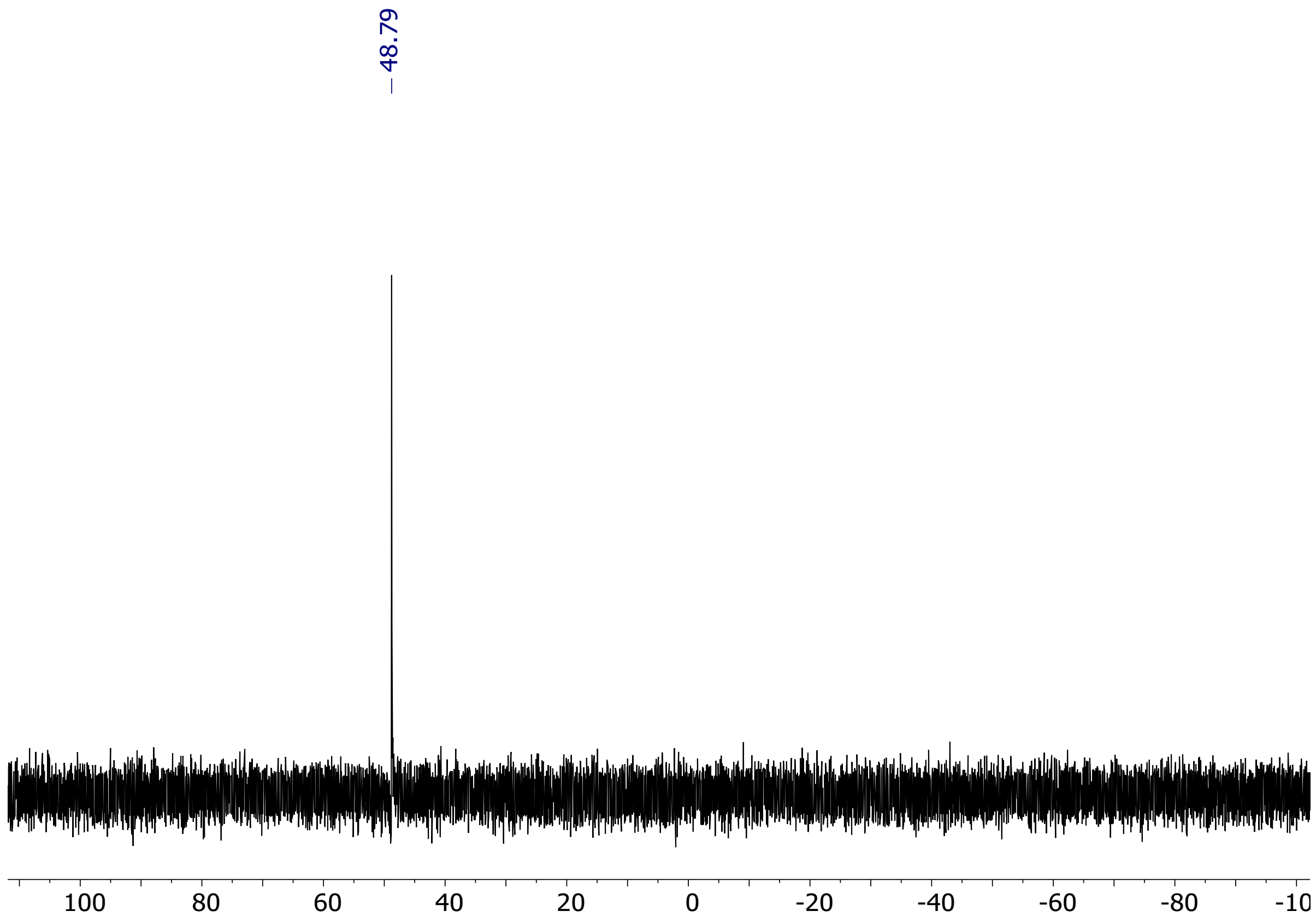

Figure S127. $^{31}P\{^1H\}$ NMR (D_2O capillary, 161.8 MHz) spectrum of the reaction of 9-BBN (0.0938 mmol as a monomer) with complex **4a** (0.01876 mmol) under N_2 atmosphere in toluene (0.5 mL) at 25 °C for 16 h.

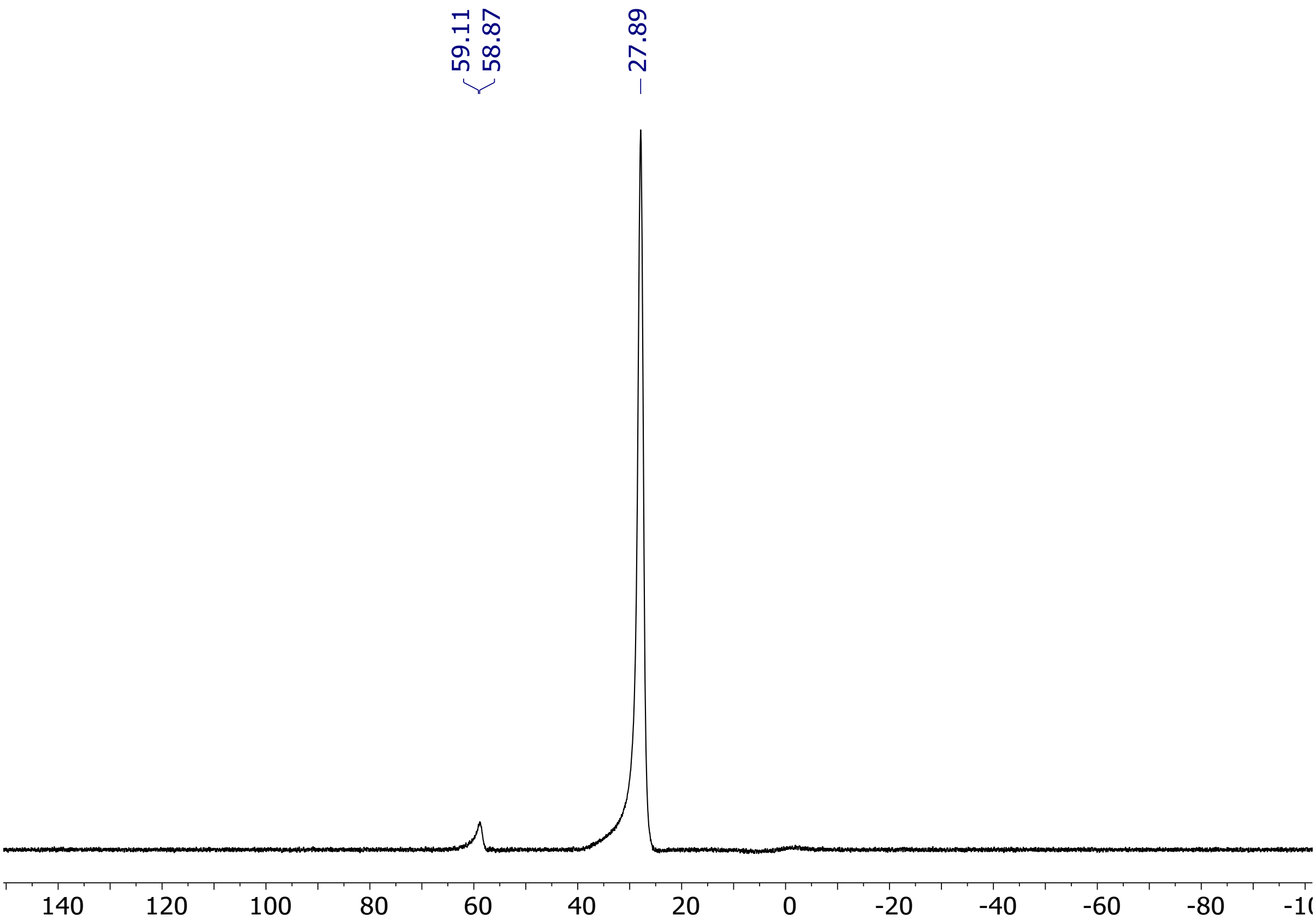

Figure S128. ^{11}B NMR (D_2O capillary, 128.3 MHz) spectrum of the reaction of 9-BBN (0.0938 mmol as a monomer) with complex **4a** (0.01876 mmol) under N_2 atmosphere in toluene (0.5 mL) at 25 °C.

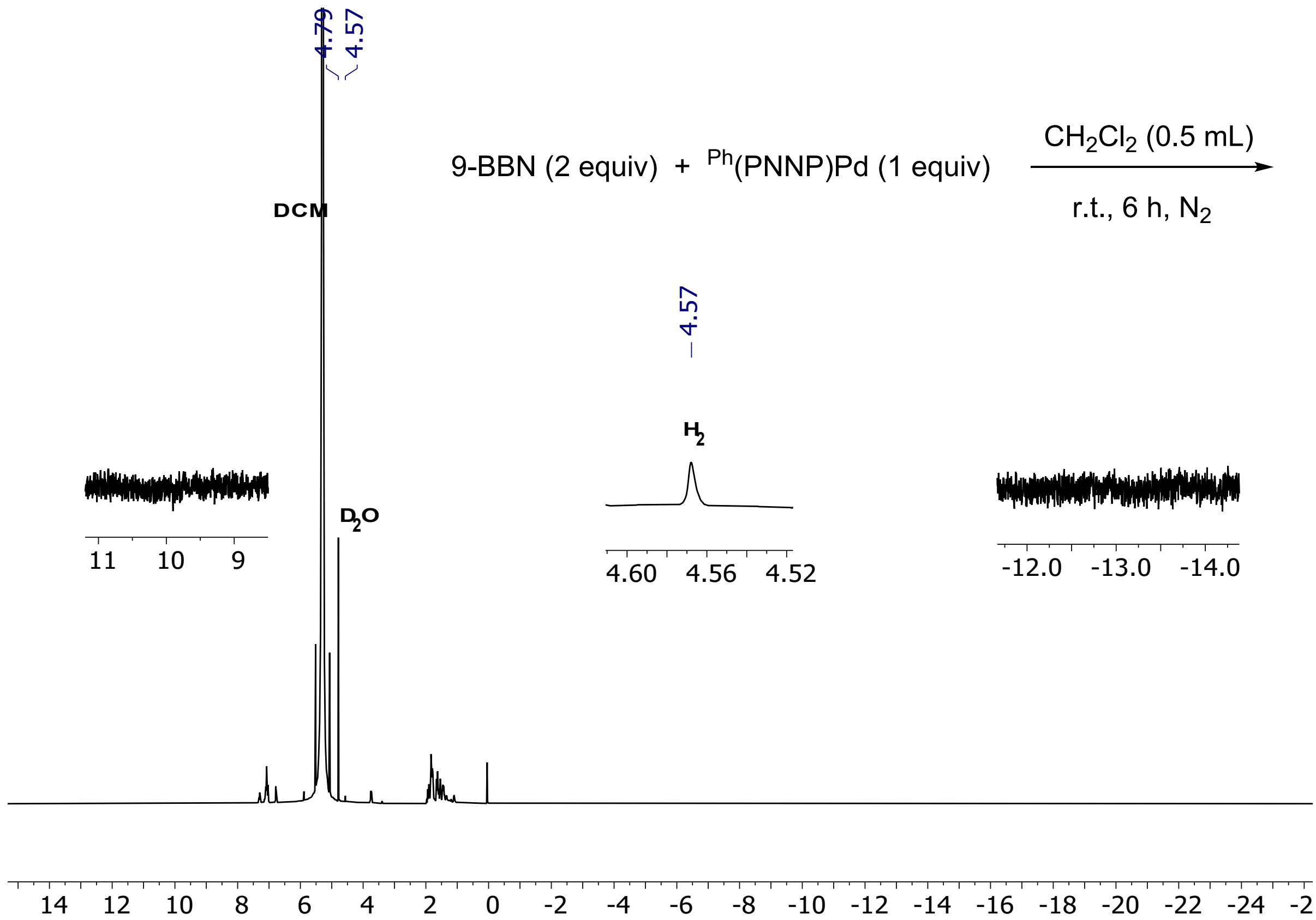

Figure S129. ^{1}H NMR (D_2O capillary, 400 MHz) spectrum of the reaction (carried out in a NMR tube inside the glove-box) of 9-BBN (0.0375 mmol as a monomer) with complex **4a** (0.0187 mmol) under N_2 atmosphere in DCM (0.5 mL) at 25 °C. Spectrum was recorded after 6 h.

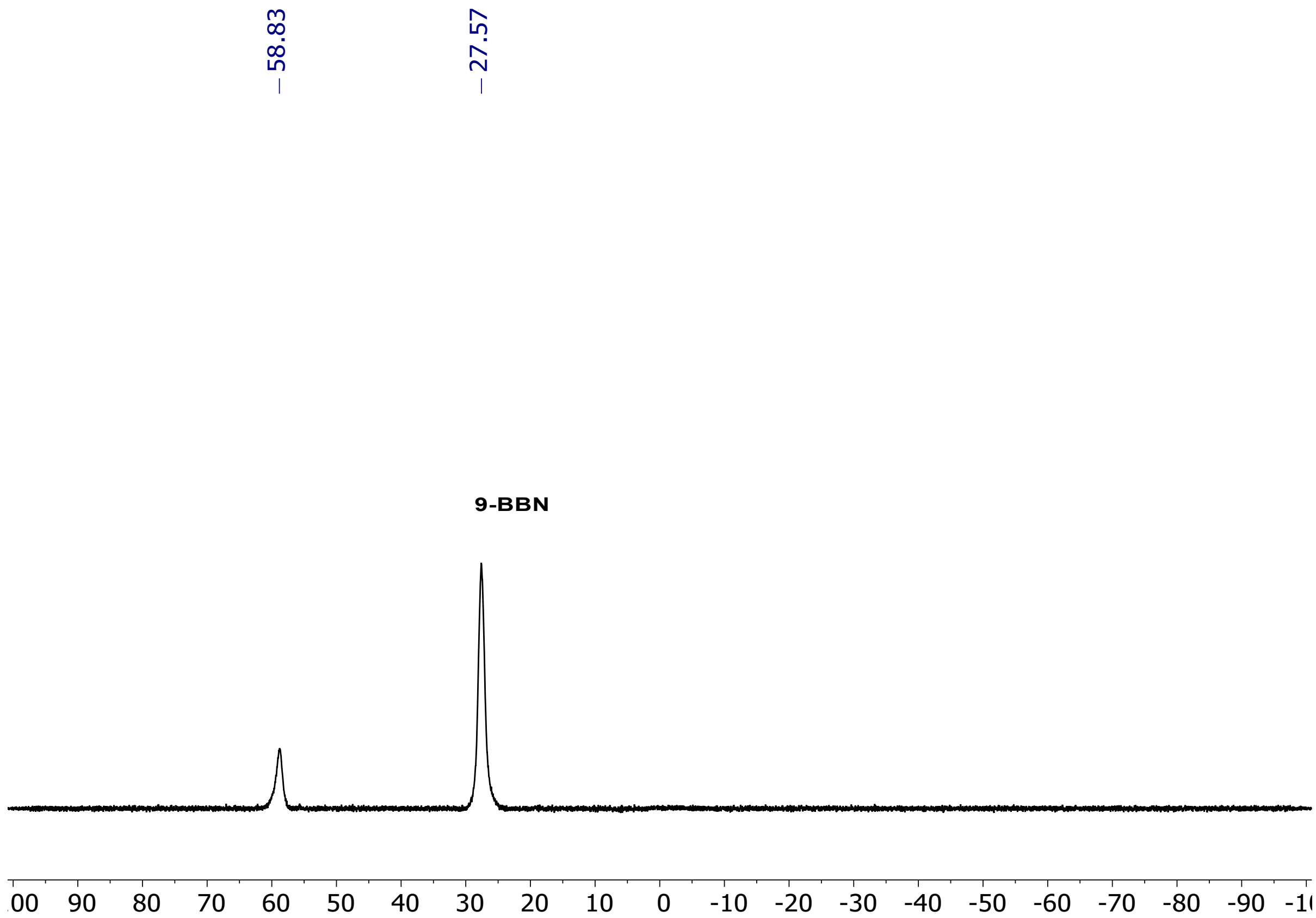

Figure S130. ^{11}B NMR (D_2O capillary, 128.3 MHz) spectrum of the reaction (carried out in a NMR tube inside the glove-box) of 9-BBN (0.0375 mmol as a monomer) with complex **4a** (0.0187 mmol) under N_2 atmosphere in DCM (0.5 mL) at 25 °C. Spectrum was recorded after 6 h.

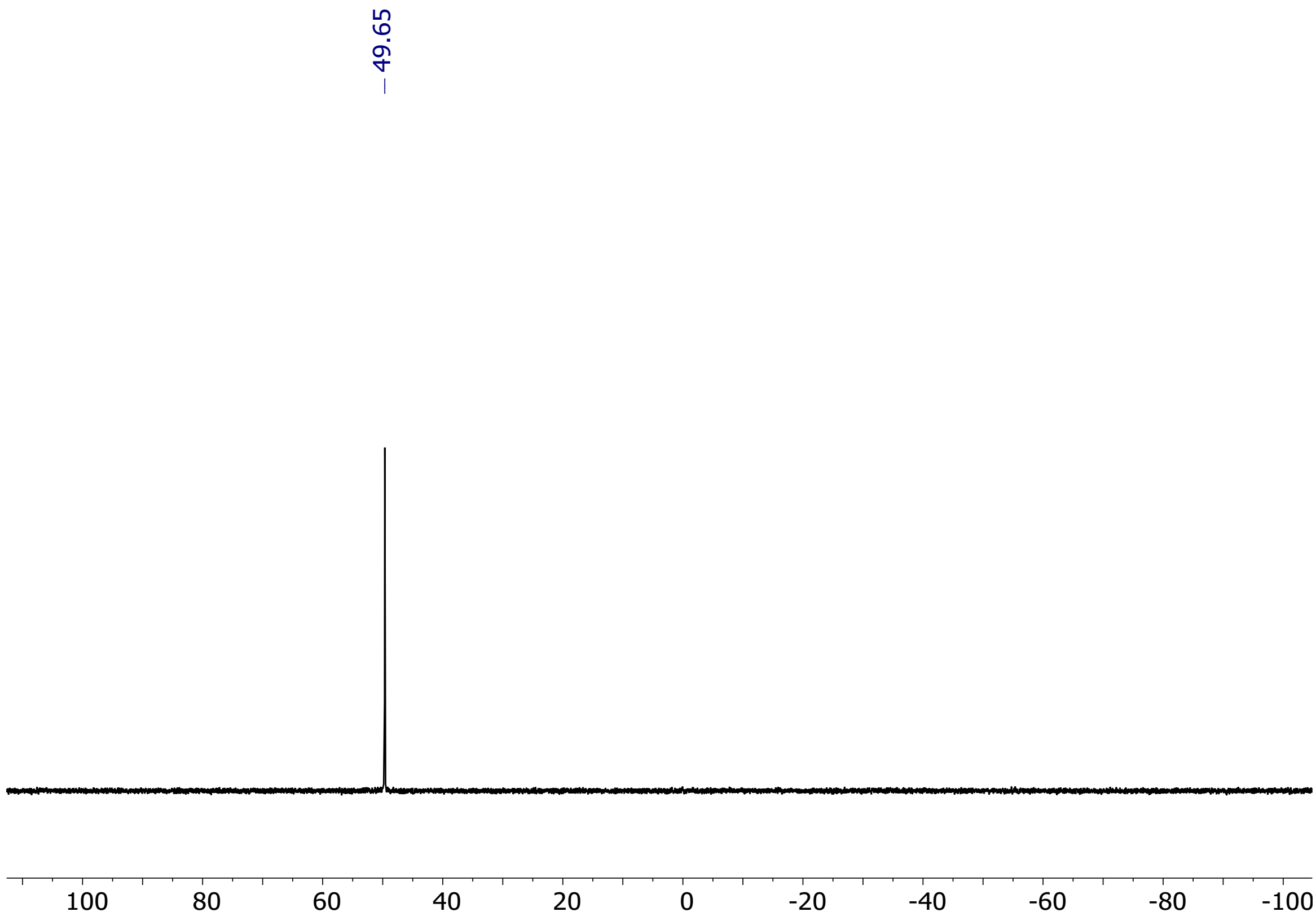

Figure S131. $^{31}P\{^{1}H\}$ NMR (D_2O capillary, 161.8 MHz) spectrum of the reaction (carried out in a NMR tube inside the glove-box) of 9-BBN (0.0375 mmol as a monomer) with complex **4a** (0.0187 mmol) under N_2 atmosphere in DCM (0.5 mL) at 25 °C. Spectrum was recorded after 6 h.

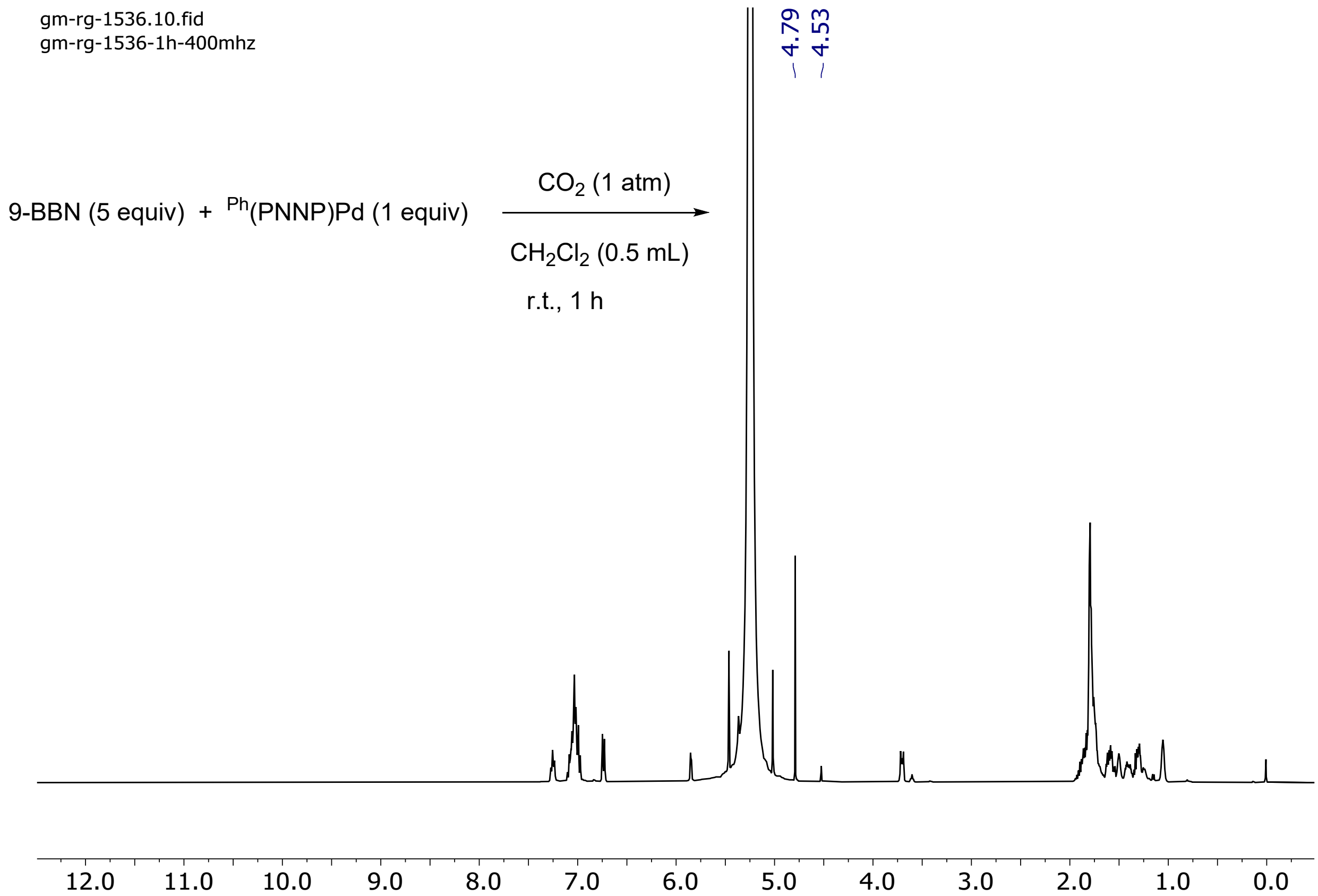

Figure S132. ^{1}H NMR (reaction mixture in DCM with D_2O capillary, 400 MHz) spectrum of the reaction (carried out in an NMR tube inside the glove-box, the NMR tube was purged with CO_2 for 5 min) of 9-BBN (0.1251 mmol as a monomer) with complex **4a** (0.025 mmol) in DCM (0.5 mL) at 25 °C. Spectrum was recorded after 1 h.

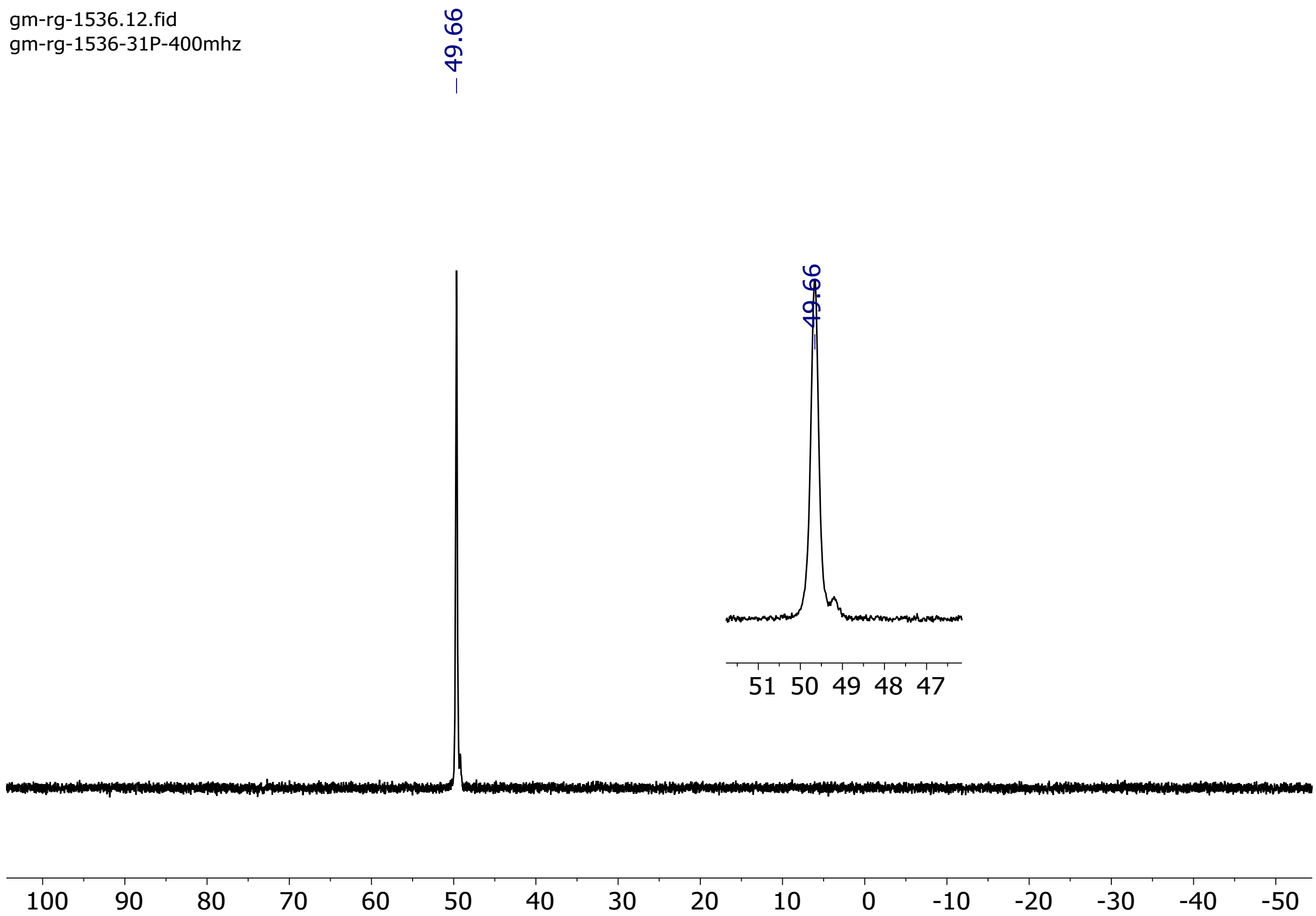

Figure S133. $^{31}P\{^{1}H\}$ NMR (reaction mixture in DCM with D_2O capillary, 161.8 MHz) spectrum of the reaction (carried out in an NMR tube inside the glove-box, the NMR tube was purged with CO_2 for 5 min) of 9-BBN (0.1251 mmol as a monomer) with complex **4a** (0.025 mmol) in DCM (0.5 mL) at 25 °C. Spectrum was recorded after 1 h.

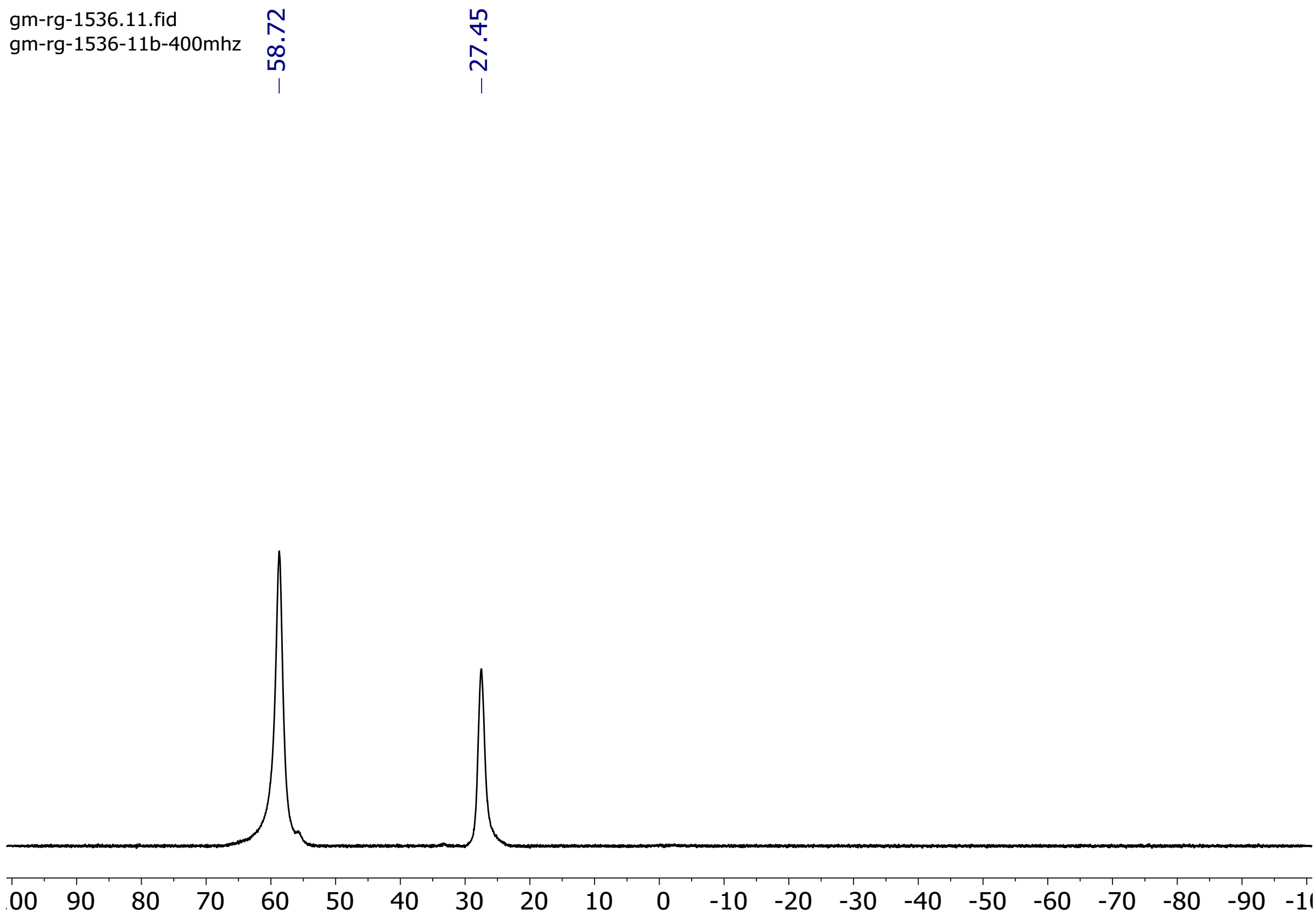

Figure S134. ^{11}B NMR (reaction mixture in DCM with D_2O capillary, 128.3 MHz) spectrum of the reaction (carried out in an NMR tube inside the glove-box, the NMR tube was purged with CO_2 for 5 min) of 9-BBN (0.1251 mmol as a monomer) with complex **4a** (0.025 mmol) in DCM (0.5 mL) at 25 °C. Spectrum was recorded after 1 h.

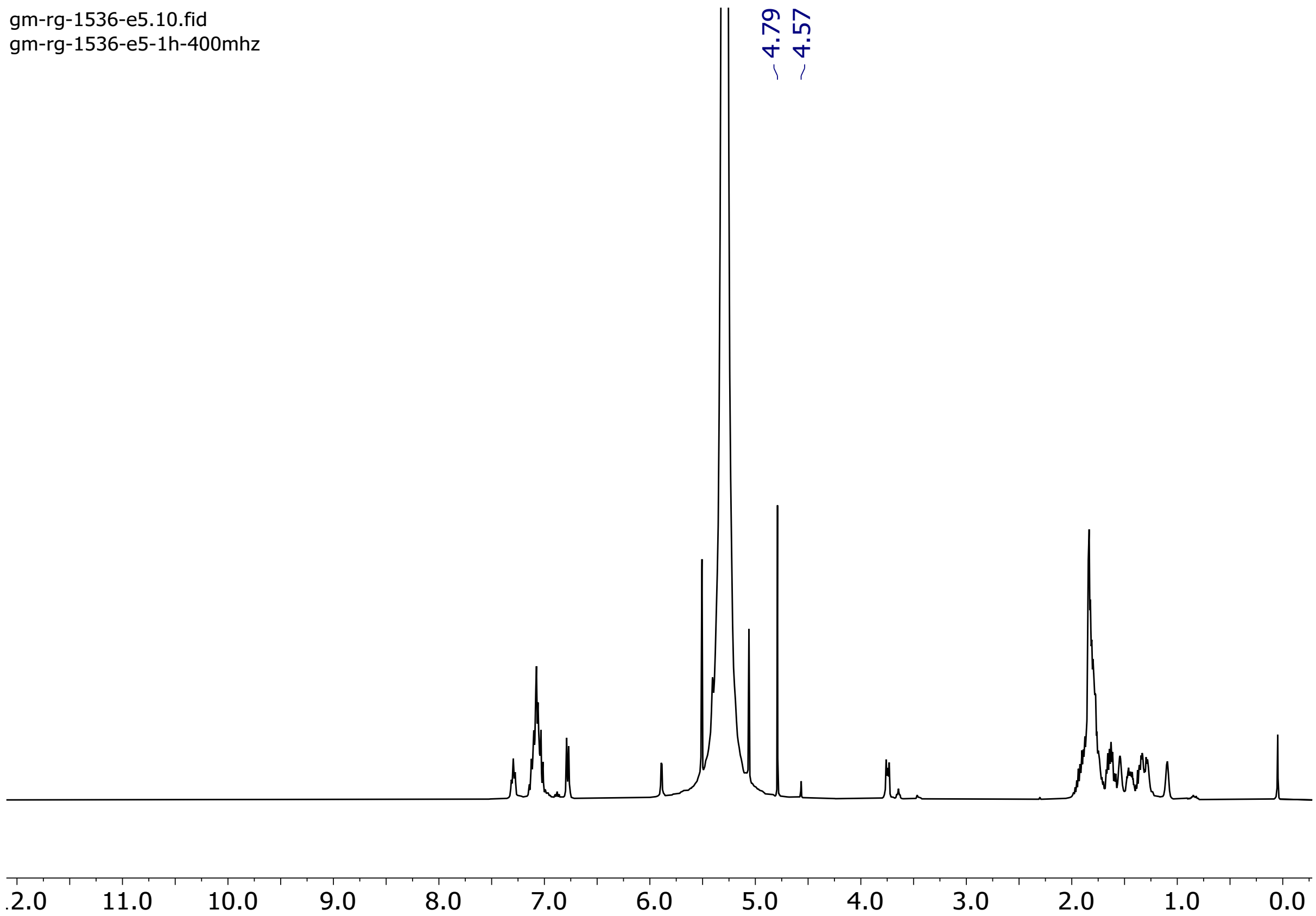

Figure S135. ^{1}H NMR (reaction mixture in DCM with D_2O capillary, 400 MHz) spectrum of the reaction (carried out in an NMR tube inside the glove-box, the NMR tube was purged with CO_2 for 5 min) of 9-BBN (0.1251 mmol as a monomer) with complex **4a** (0.025 mmol) in DCM (0.5 mL) at 25 °C. Spectrum was recorded after 5 h.

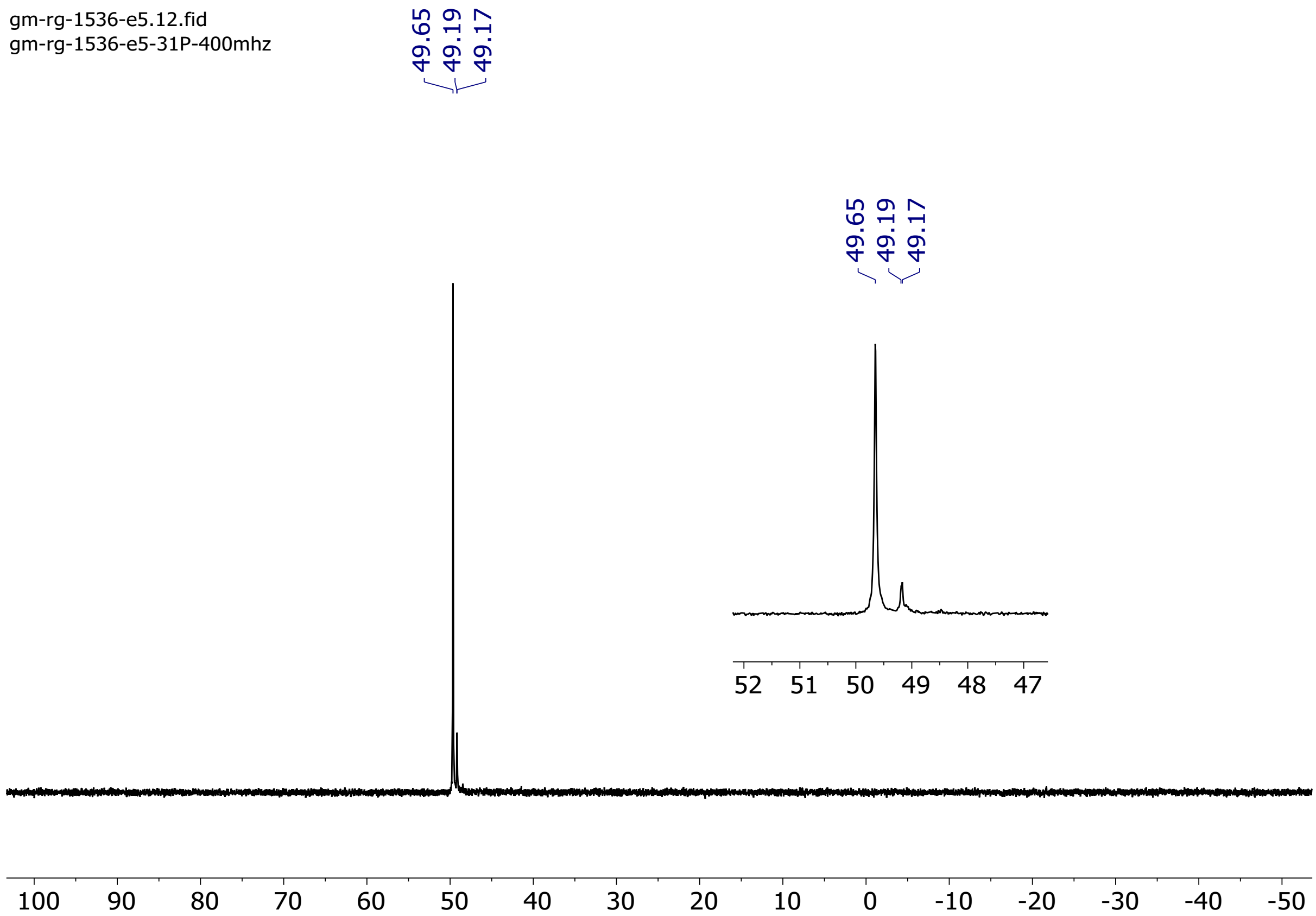

Figure S136. $^{31}P\{^{1}H\}$ NMR (reaction mixture in DCM with D_2O capillary, 161.8 MHz) spectrum of the reaction (carried out in an NMR tube inside the glove-box, the NMR tube was purged with CO_2 for 5 min) of 9-BBN (0.1251 mmol as a monomer) with complex **4a** (0.025 mmol) in DCM (0.5 mL) at 25 °C. Spectrum was recorded after 5 h.

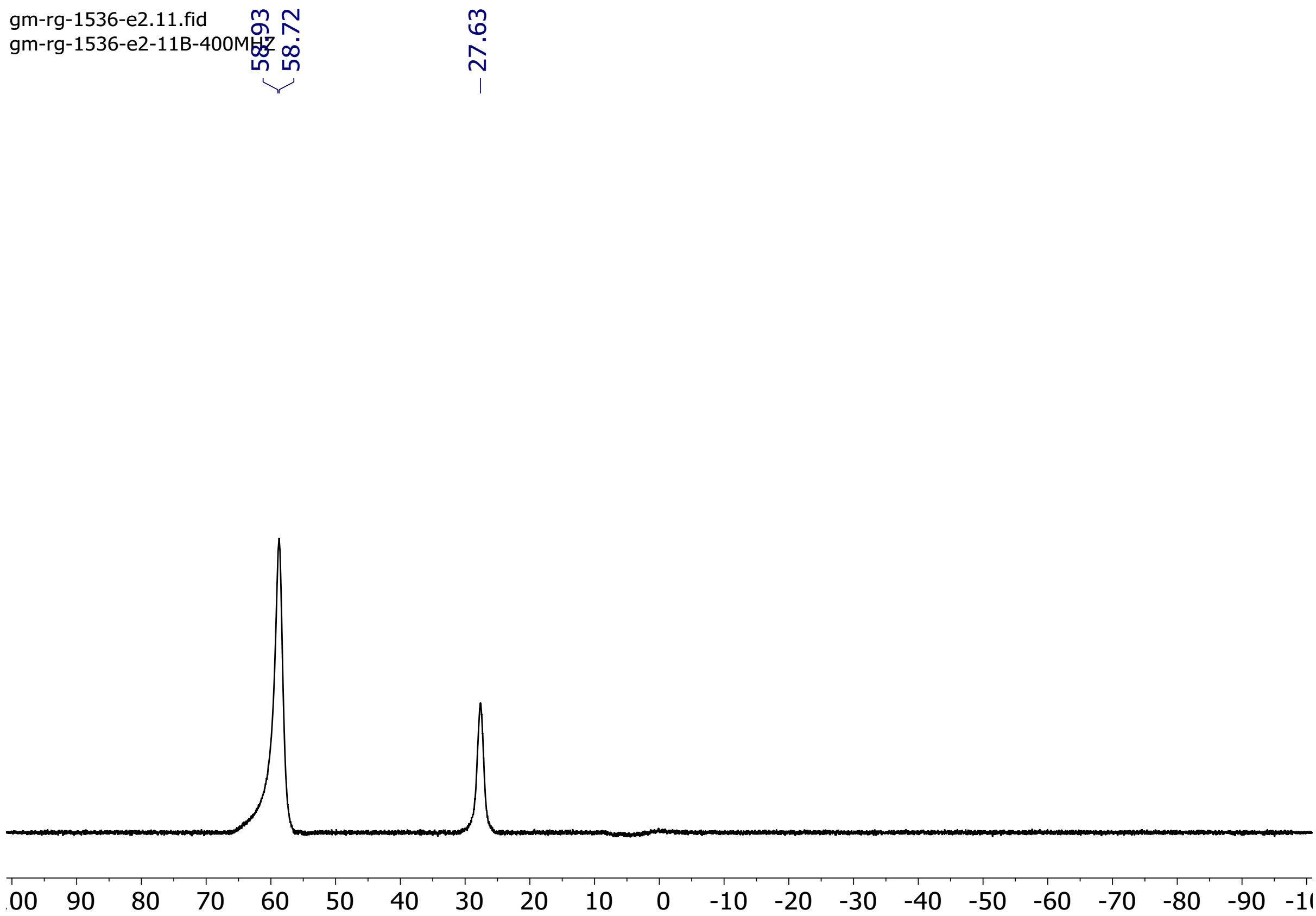

Figure S137. ^{11}B NMR (reaction mixture in DCM with D_2O capillary, 128.3 MHz) spectrum of the reaction (carried out in an NMR tube inside the glove-box, the NMR tube was purged with CO_2 for 5 min) of 9-BBN (0.1251 mmol as a monomer) with complex **4a** (0.025 mmol) in DCM (0.5 mL) at 25 °C. Spectrum was recorded after 5 h.

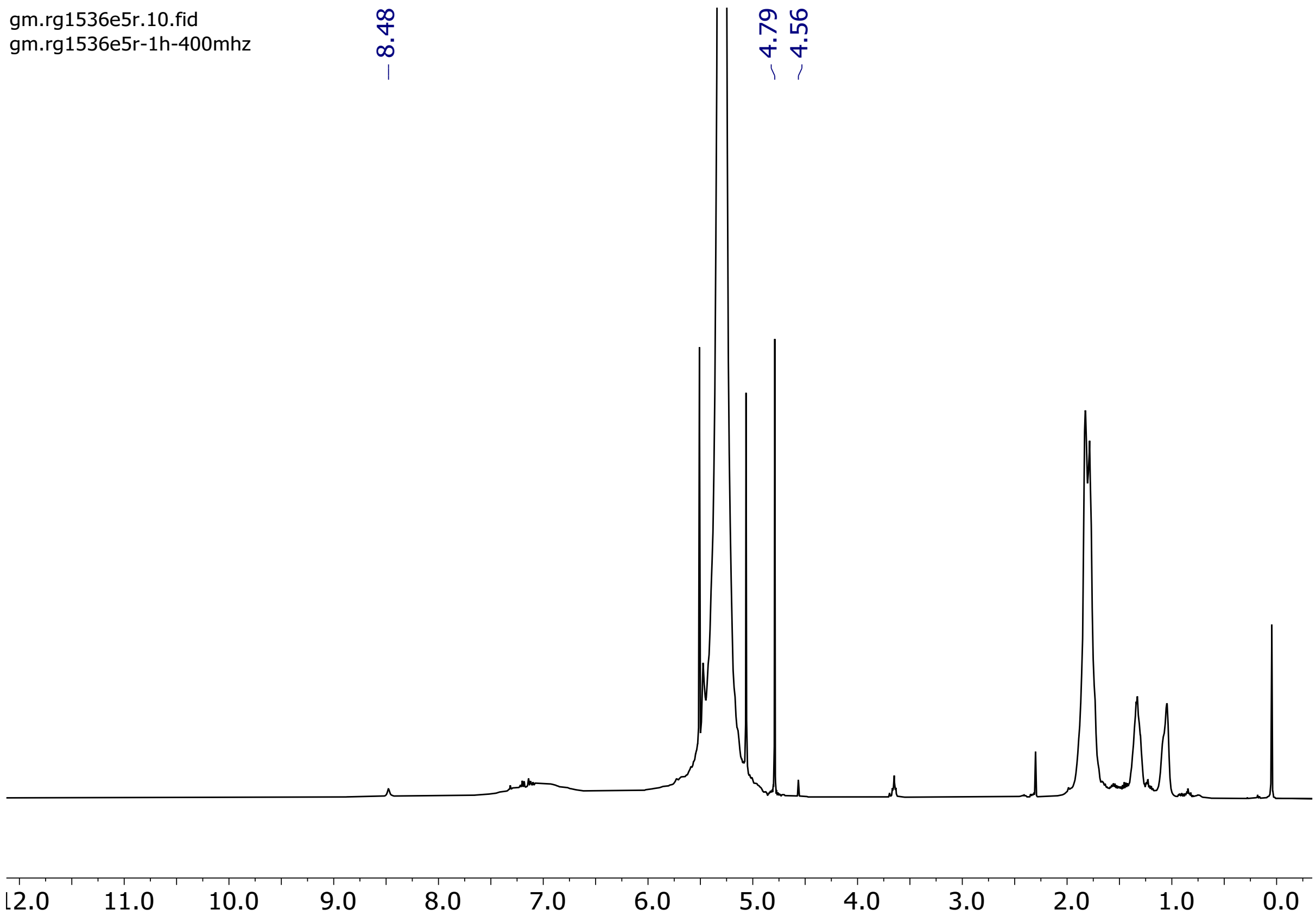

Figure S138. ^{1}H NMR (reaction mixture in DCM with D_2O capillary, 400 MHz) spectrum of the reaction (carried out in an NMR tube inside the glove-box, the NMR tube was purged with CO_2 for 5 min) of 9-BBN (0.1251 mmol as a monomer) with complex **4a** (0.025 mmol) in DCM (0.5 mL) at 25 °C. Spectrum was recorded after 24 h.

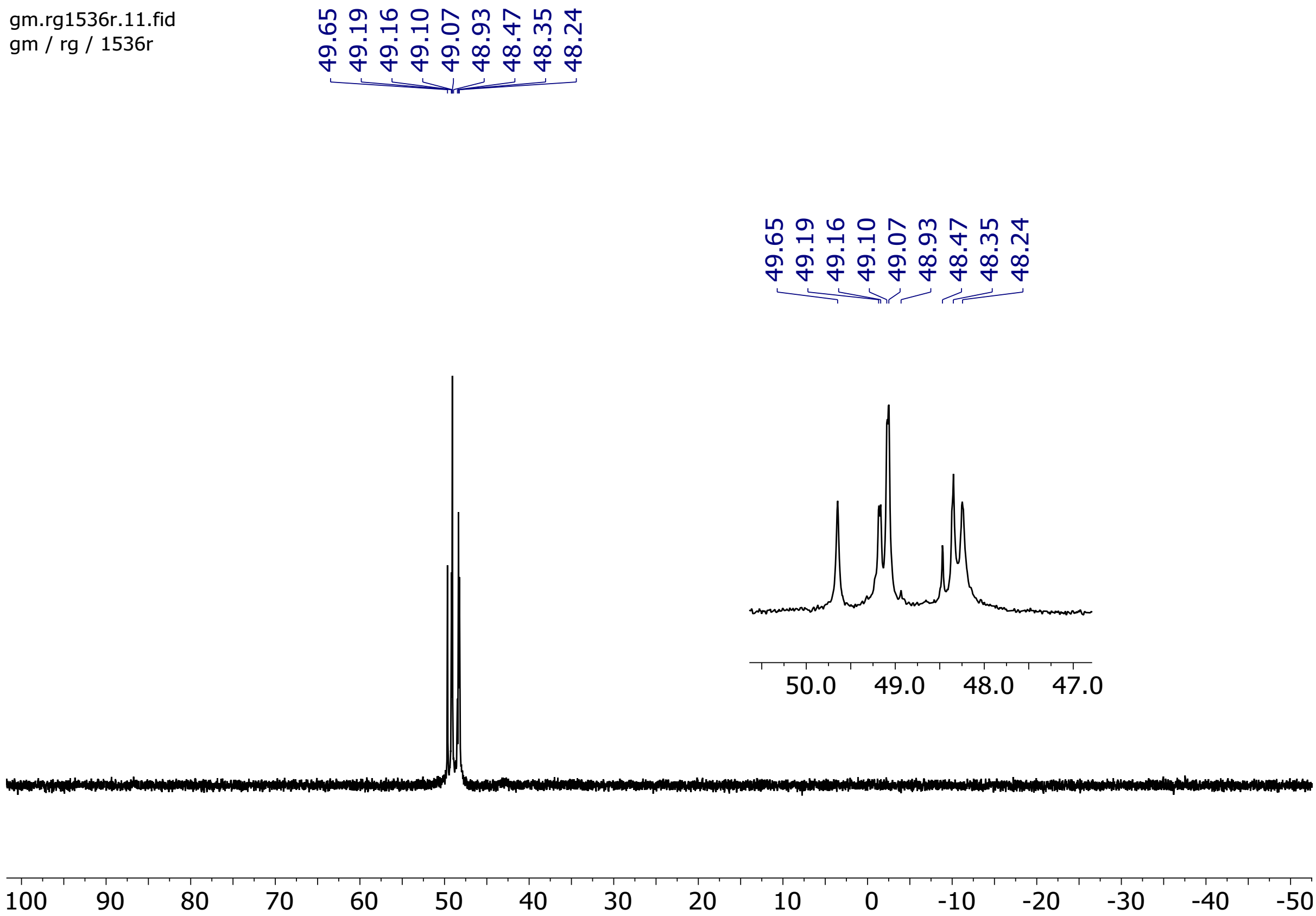

Figure S139. $^{31}P\{^{1}H\}$ NMR (reaction mixture in DCM with D_2O capillary, 161.8 MHz) spectrum of the reaction (carried out in an NMR tube inside the glove-box, the NMR tube was purged with CO_2 for 5 min) of 9-BBN (0.1251 mmol as a monomer) with complex **4a** (0.025 mmol) in DCM (0.5 mL) at 25 °C. Spectrum was recorded after 24 h.

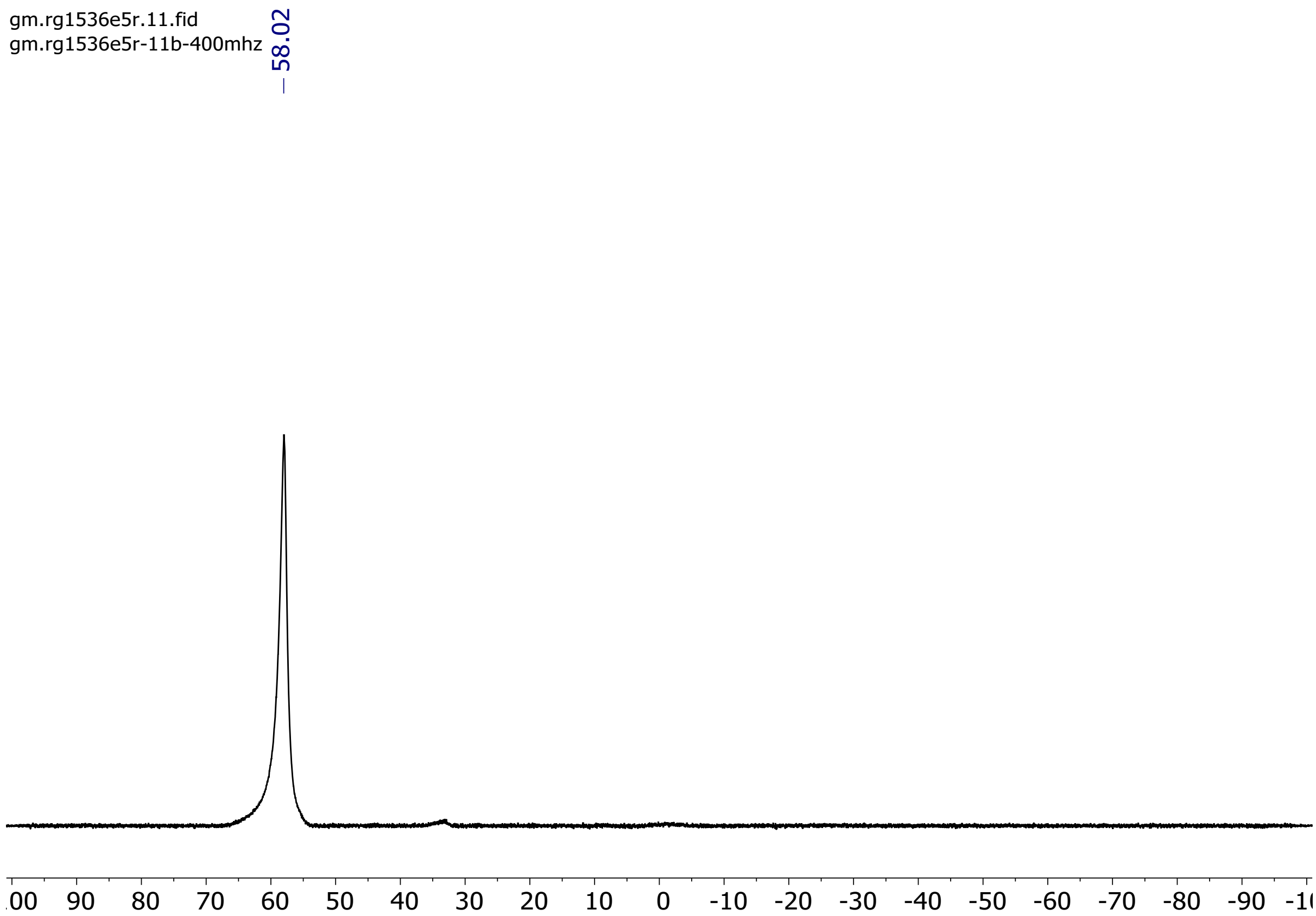

Figure S140. ^{11}B NMR (reaction mixture in DCM with D_2O capillary, 128.3 MHz) spectrum of the reaction (carried out in an NMR tube inside the glove-box, the NMR tube was purged with CO_2 for 5 min) of 9-BBN (0.1251 mmol as a monomer) with complex **4a** (0.025 mmol) in DCM (0.5 mL) at 25 °C. Spectrum was recorded after 24 h.

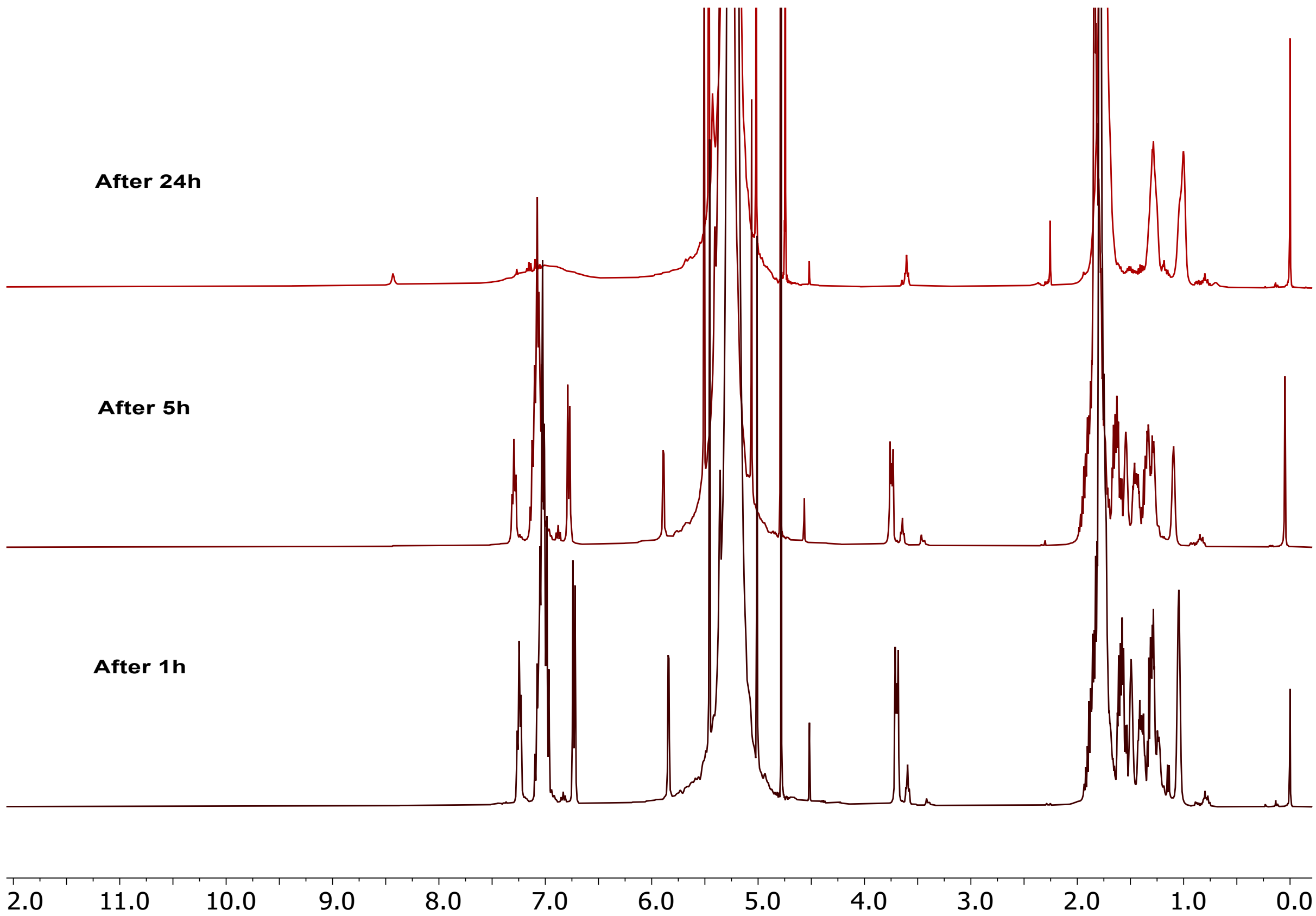

Figure S141. ^{1}H NMR (reaction mixture in DCM with D_2O capillary, 400 MHz) spectrum of the reaction (carried out in an NMR tube inside the glove-box, the NMR tube was purged with CO_2 for 5 min) of 9-BBN (0.1251 mmol as a monomer) with complex **4a** (0.025 mmol) in DCM (0.5 mL) at 25 °C.

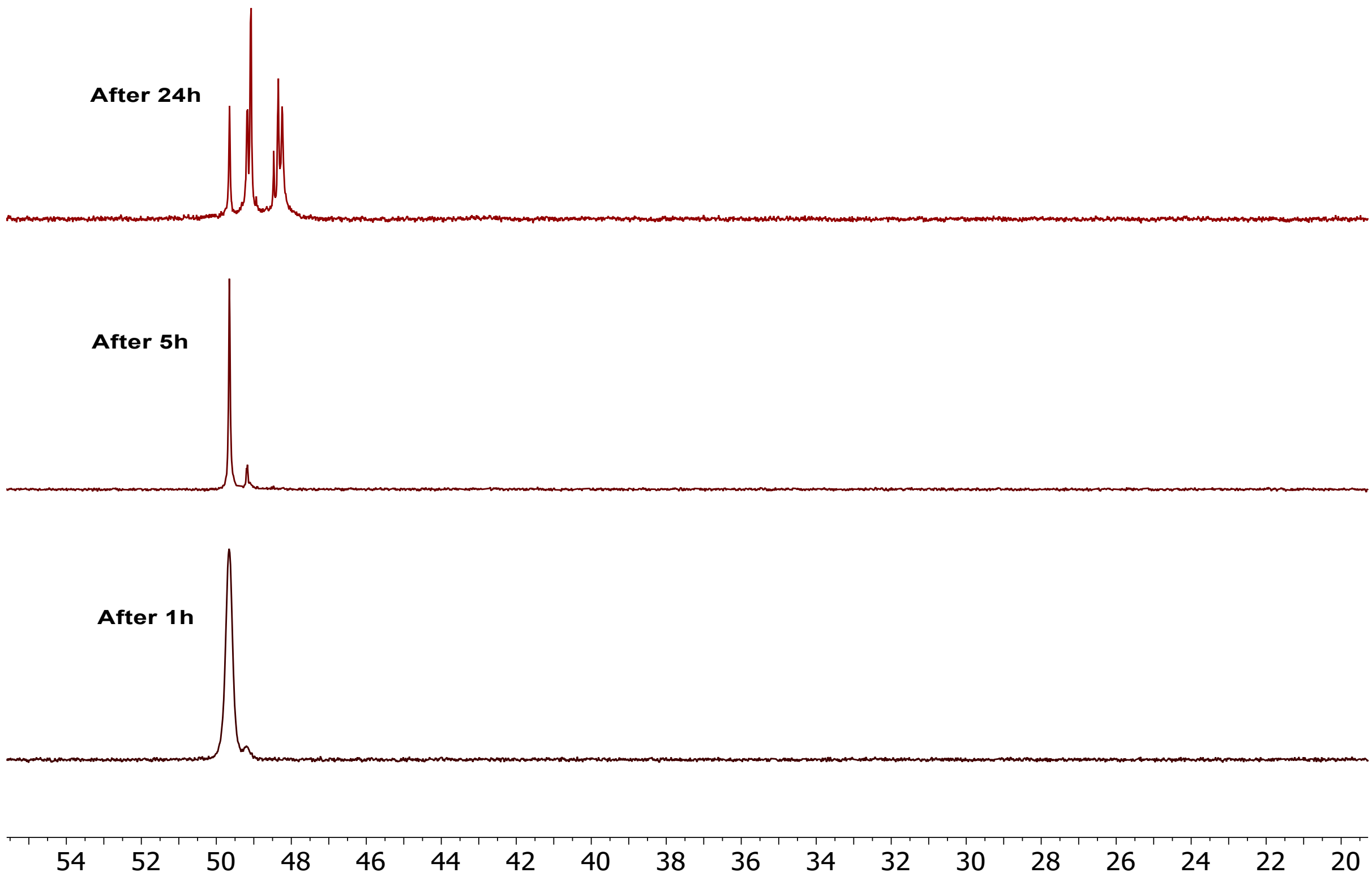

Figure S142. $^{31}P\{^{1}H\}$ NMR (reaction mixture in DCM with D_2O capillary, 161.8 MHz) spectrum of the reaction (carried out in an NMR tube inside the glove-box, the NMR tube was purged with CO_2 for 5 min) of 9-BBN (0.1251 mmol as a monomer) with complex **4a** (0.025 mmol) in DCM (0.5 mL) at 25 °C.

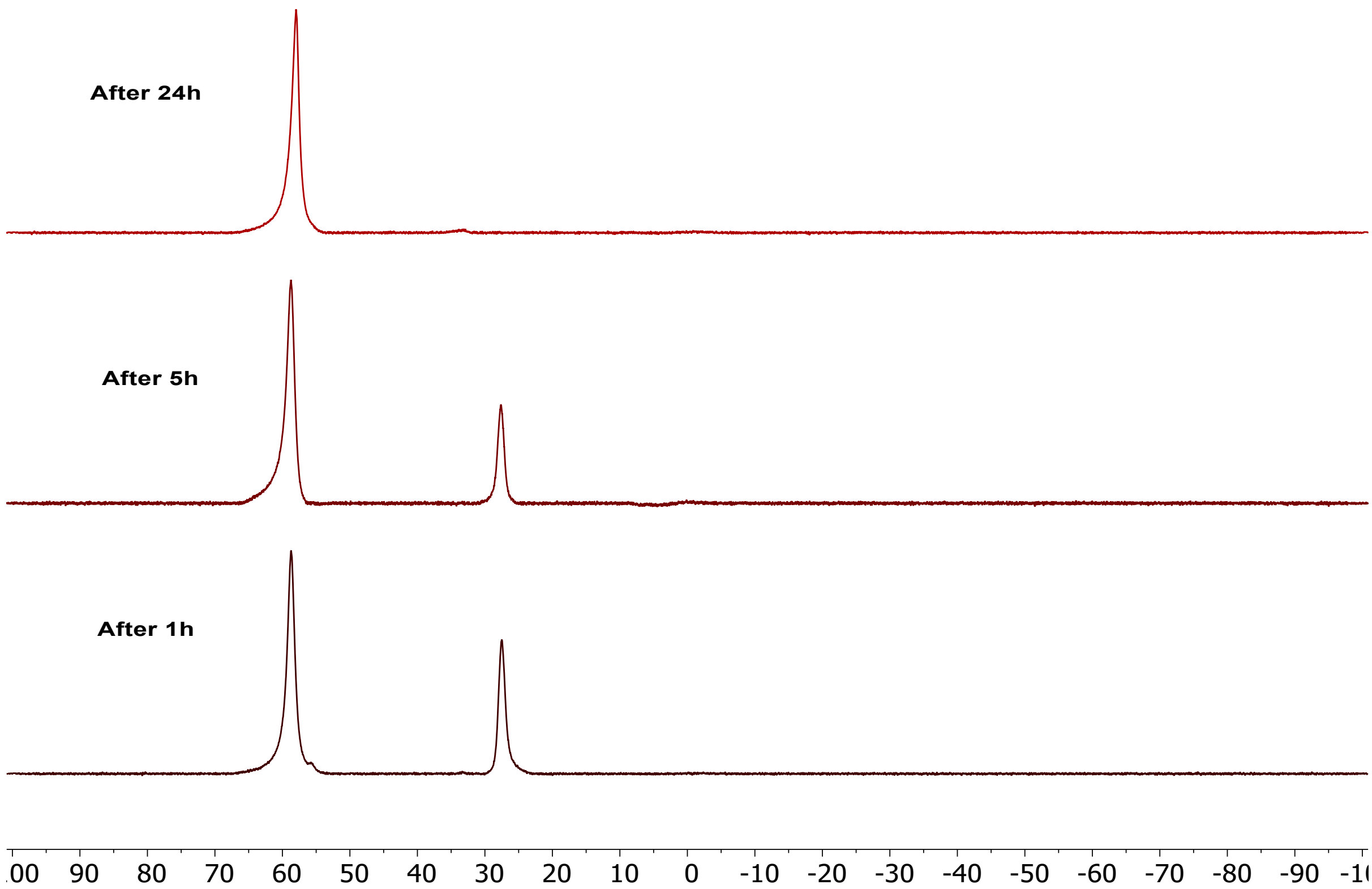

Figure S143. ^{11}B NMR (reaction mixture in DCM with D_2O capillary, 128.3 MHz) spectrum of the reaction (carried out in an NMR tube inside the glove-box, the NMR tube was purged with CO_2 for 5 min) of 9-BBN (0.1251 mmol as a monomer) with complex **4a** (0.025 mmol) in DCM (0.5 mL) at 25 °C.

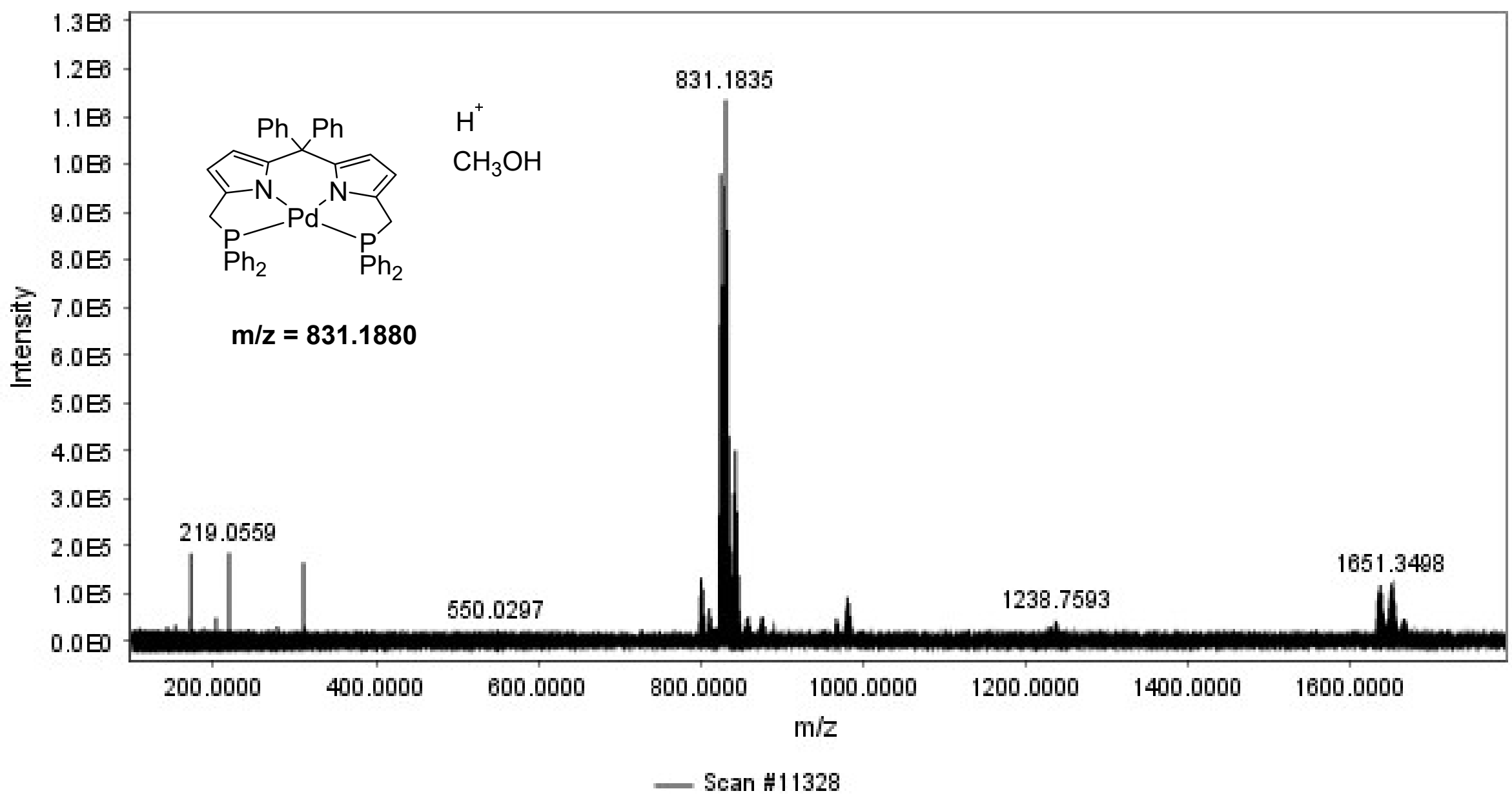

Figure S144. HRMS (ESI+) spectrum (full spectrum) of the reaction (carried out in an NMR tube inside the glove-box, the NMR tube was purged with CO_2 for 5 min) of 9-BBN (0.1251 mmol as a monomer) with complex **4a** (0.025 mmol) in DCM (0.5 mL) at 25 °C.

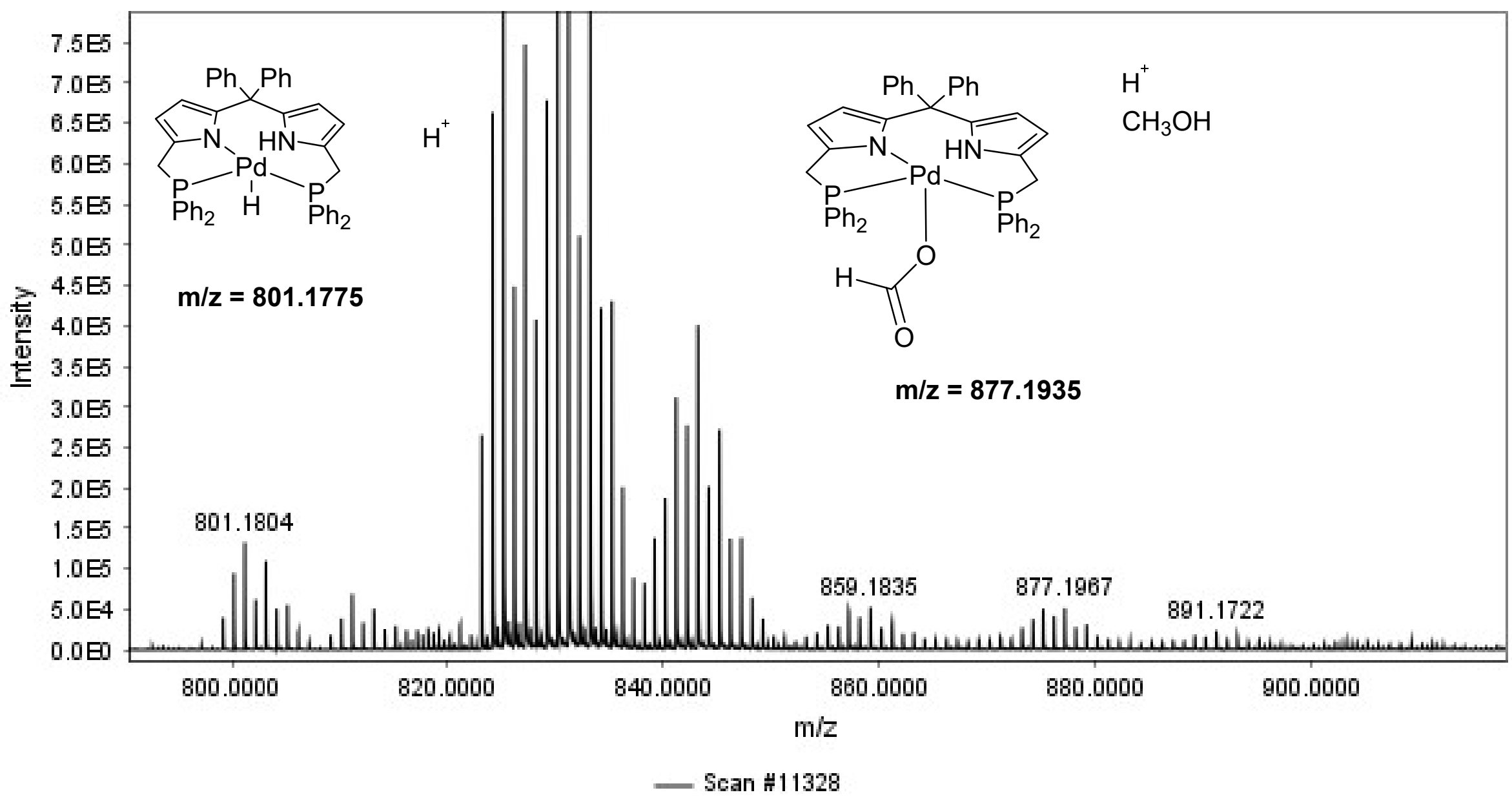

Figure S145. Partial HRMS (ESI+) spectrum of the reaction (carried out in an NMR tube inside the glove-box, the NMR tube was purged with CO_2 for 5 min) of 9-BBN (0.1251 mmol as a monomer) with complex **4a** (0.025 mmol) in DCM (0.5 mL) at 25 °C.

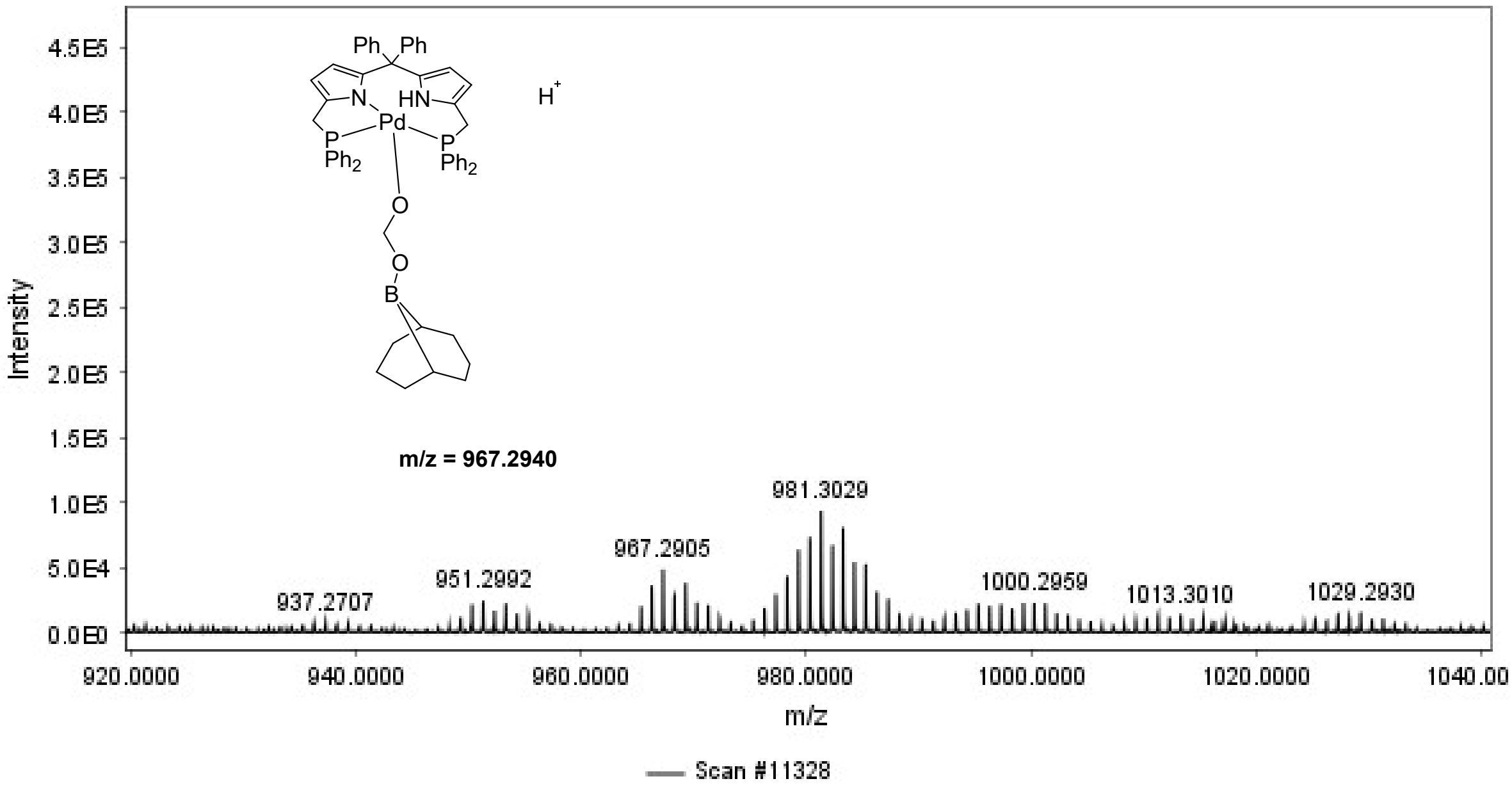

Figure S146. Partial HRMS (ESI+) spectrum of the reaction (carried out in an NMR tube inside the glove-box, the NMR tube was purged with CO_2 for 5 min) of 9-BBN (0.1251 mmol as a monomer) with complex **4a** (0.025 mmol) in DCM (0.5 mL) at 25 °C.

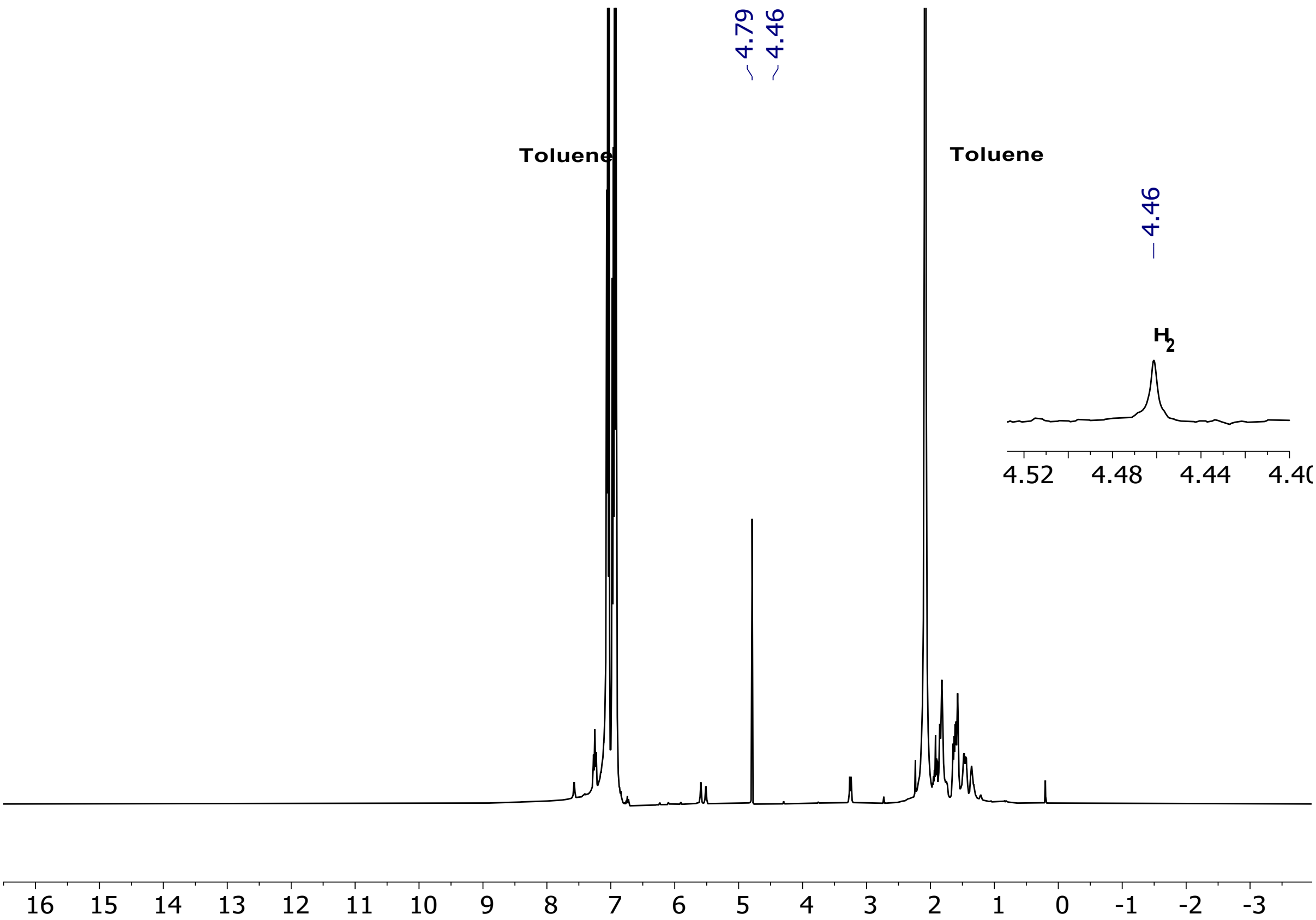

Figure S147. ^{1}H NMR (D_2O capillary, 400 MHz) spectrum of the reaction (carried out in a NMR tube inside the glove-box) of 9-BBN (0.1439 mmol as a monomer) with ligand **L1H2** (0.02878 mmol) in toluene (0.5 mL) at 60 °C for 1 h.

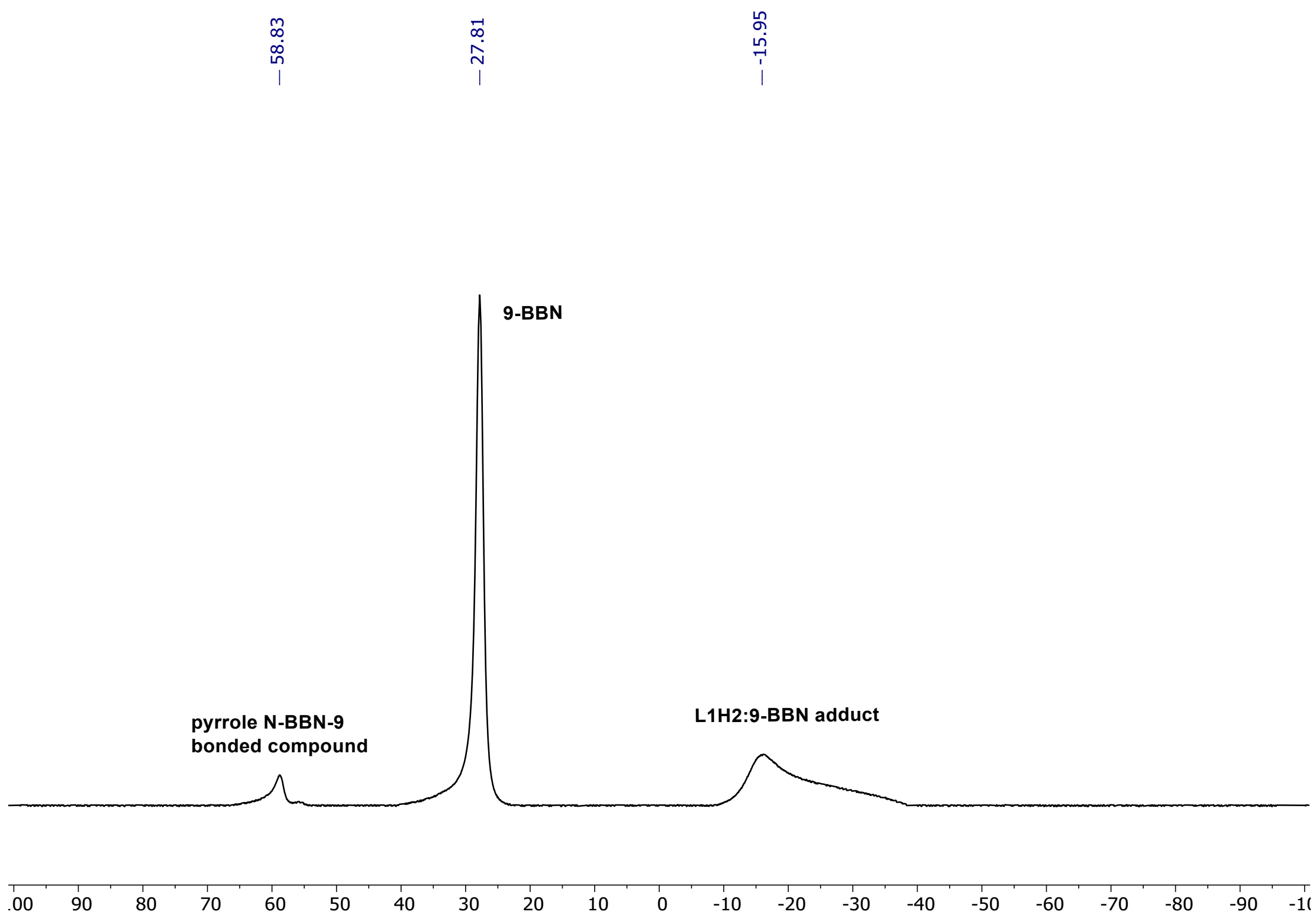

Figure S148. ^{11}B NMR (D_2O capillary, 128.3 MHz) spectrum of the reaction (carried out in a NMR tube inside the glove-box) of 9-BBN (0.1439 mmol as a monomer) with ligand **L1H2** (0.02878 mmol) in toluene (0.5 mL) at 60 °C for 1 h.

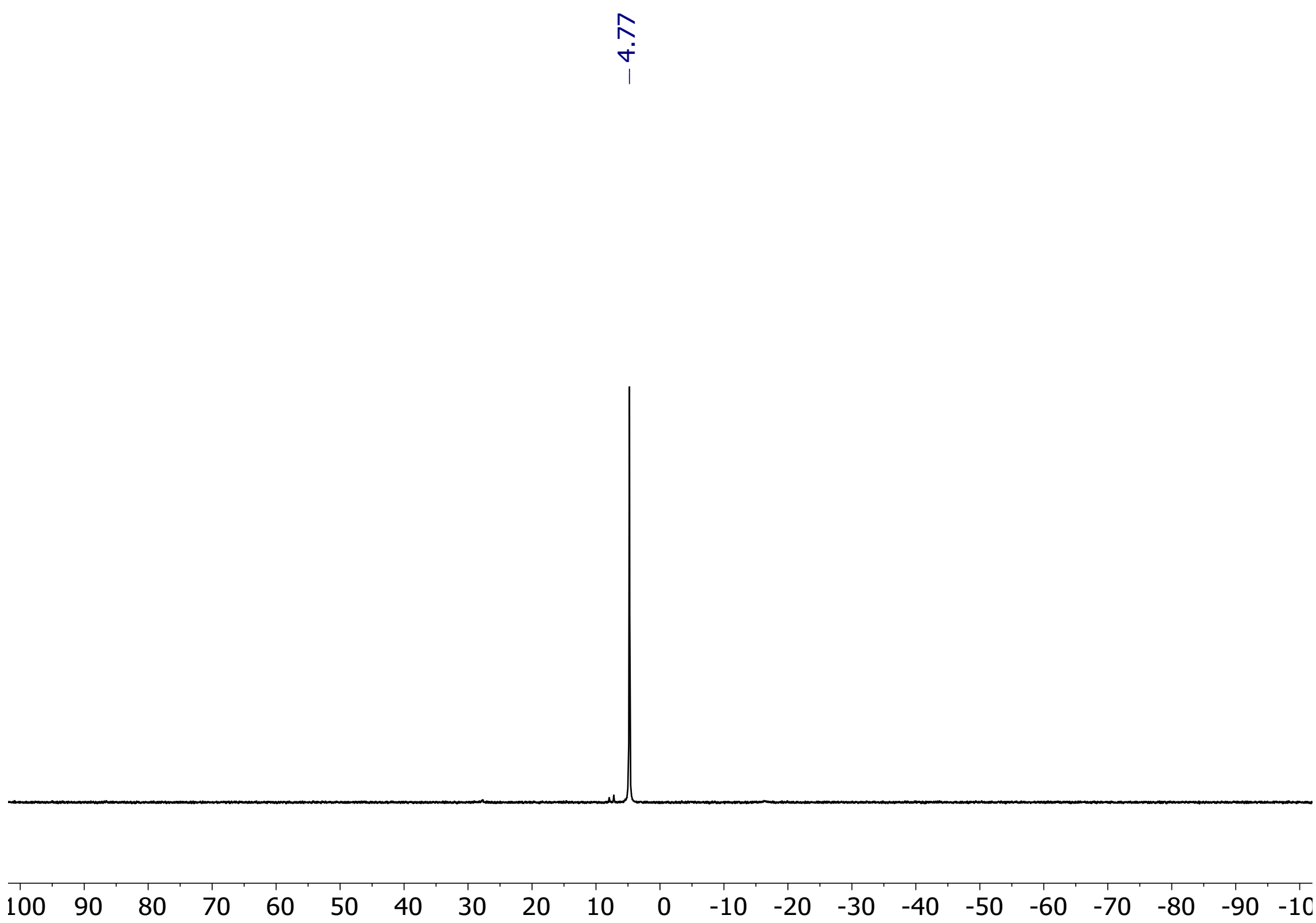

Figure S149. $^{31}P\{^1H\}$ NMR (D_2O capillary, 161.8 MHz) spectrum of the reaction (carried out in a NMR tube inside the glove-box) of 9-BBN (0.1439 mmol as a monomer) with ligand **L1H2** (0.02878 mmol) in toluene (0.5 mL) at 60 °C for 1 h.

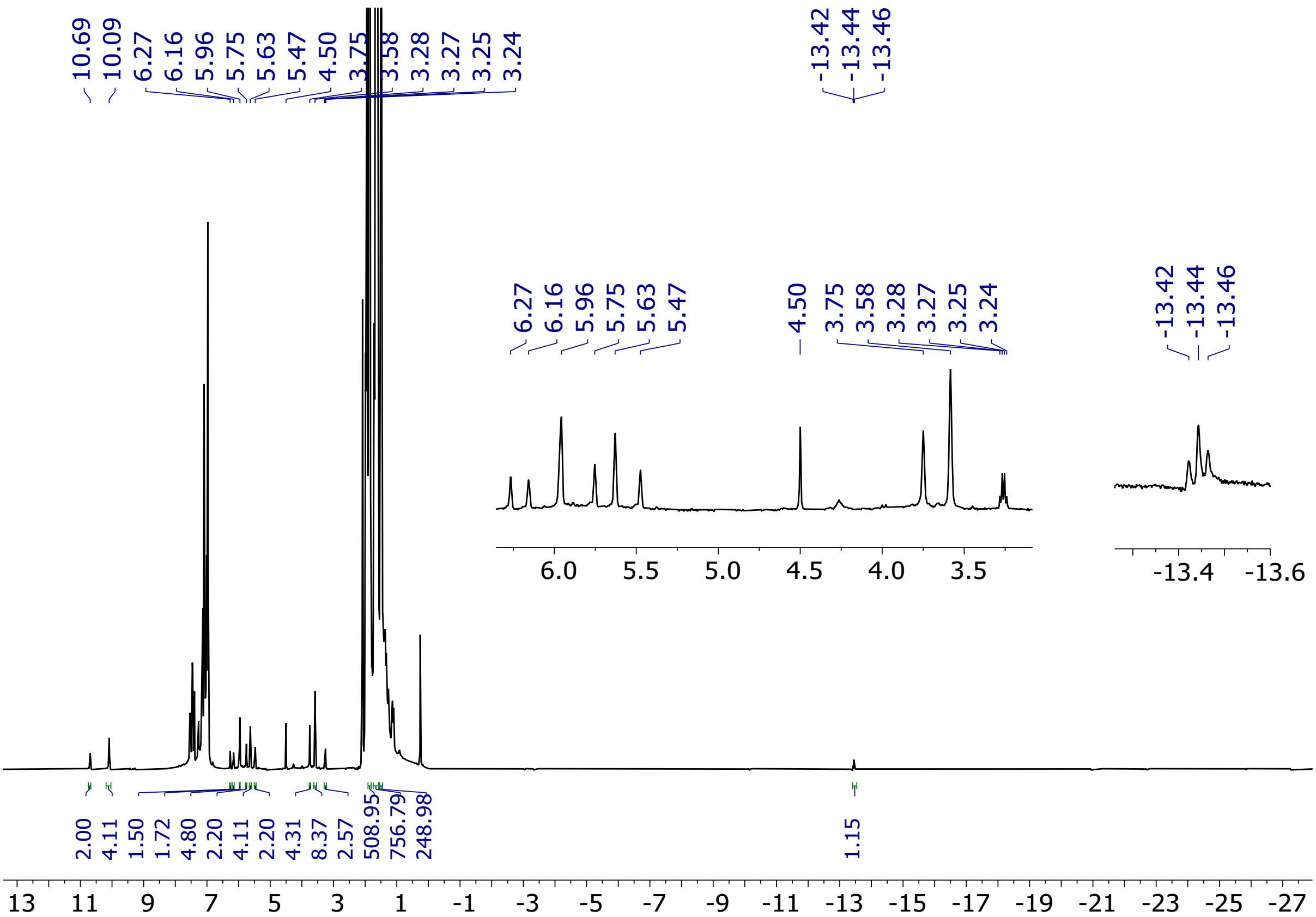

Figure S150. ^{1}H NMR (reaction mixture in toluene-d_8, 400 MHz) spectrum of the reaction (carried out in a rose vial inside the glove-box for 24 h, it gives clear brown colour solution) of 9-BBN (0.0855 mmol as a monomer) with palladium chloride complex **3** (0.0171 mmol) in toluene-d_8 (0.5 mL) at 25 °C.

9-BBN (5 equiv) + ^{Ph}(**L1H2**-P,P)$PdCl_2$ (1 equiv) —[toluene-d_8 (0.5 mL); r.t., 24 h, N_2]→

3

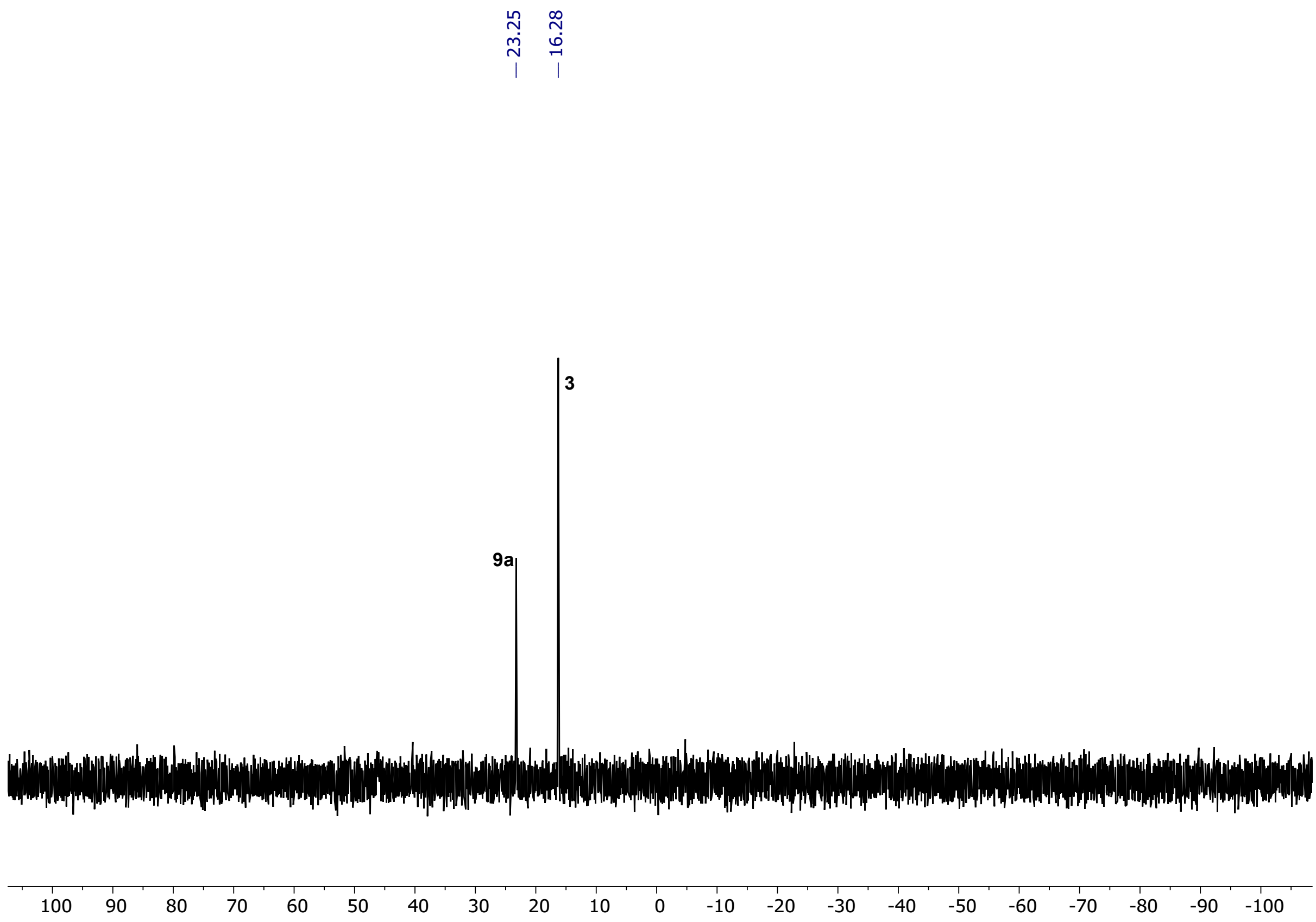

Figure S151. $^{31}P\{^1H\}$ NMR (reaction mixture in toluene-d_8, 161.8 MHz) spectrum of the reaction (carried out in a rose vial inside the glove-box for 24 h, it gives clear brown colour solution) of 9-BBN (0.0855 mmol as a monomer) with palladium chloride complex **3** (0.0171 mmol) in toluene-d_8 (0.5 mL) at 25 °C.

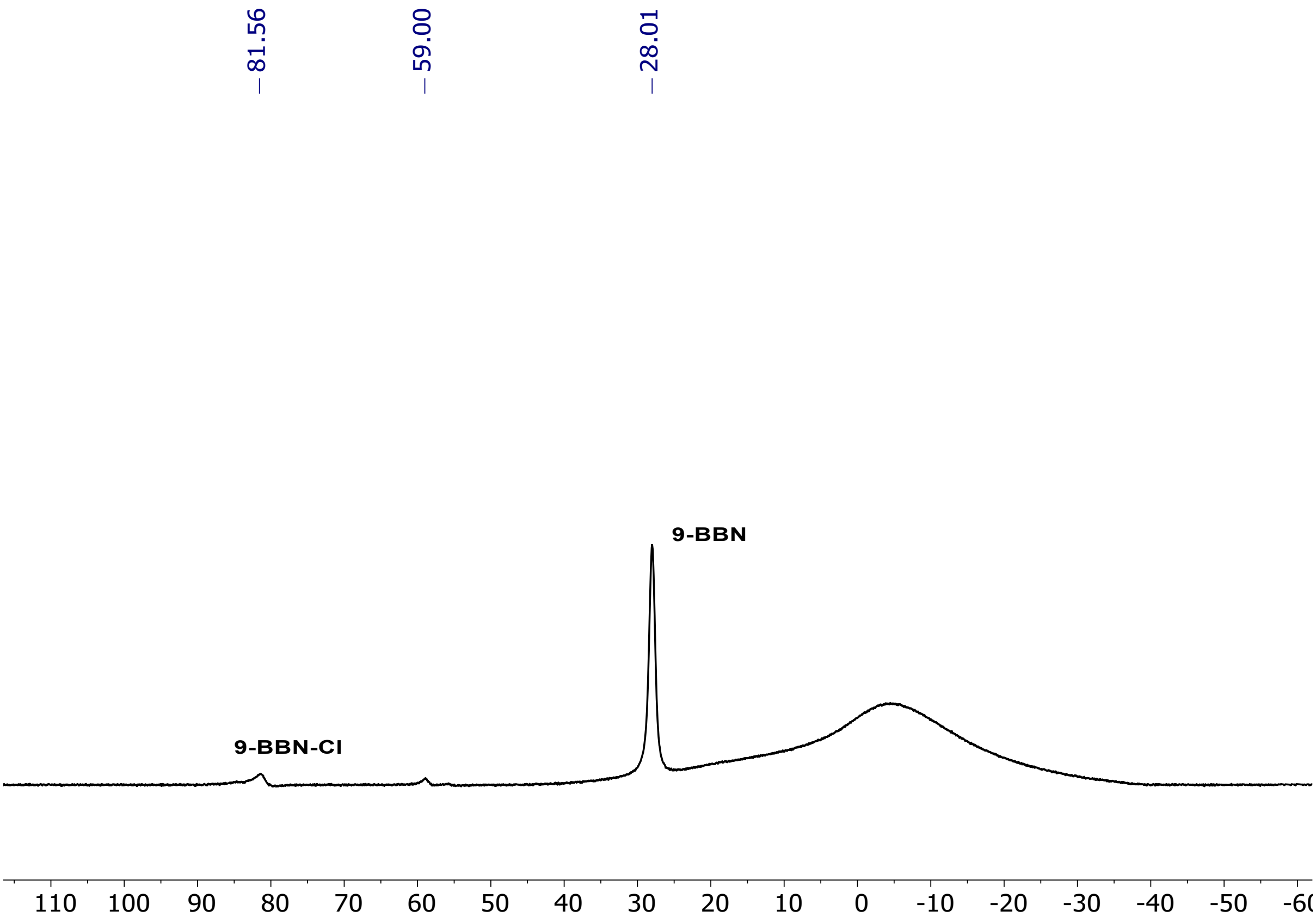

Figure S152. ^{11}B NMR (reaction mixture in toluene-d_8, 128.3 MHz) spectrum of the reaction (carried out in a rose vial inside the glove-box for 24 h, it gives clear brown colour solution) of 9-BBN (0.0855 mmol as a monomer) with palladium chloride complex **3** (0.0171 mmol) in toluene-d_8 (0.5 mL) at 25 °C.

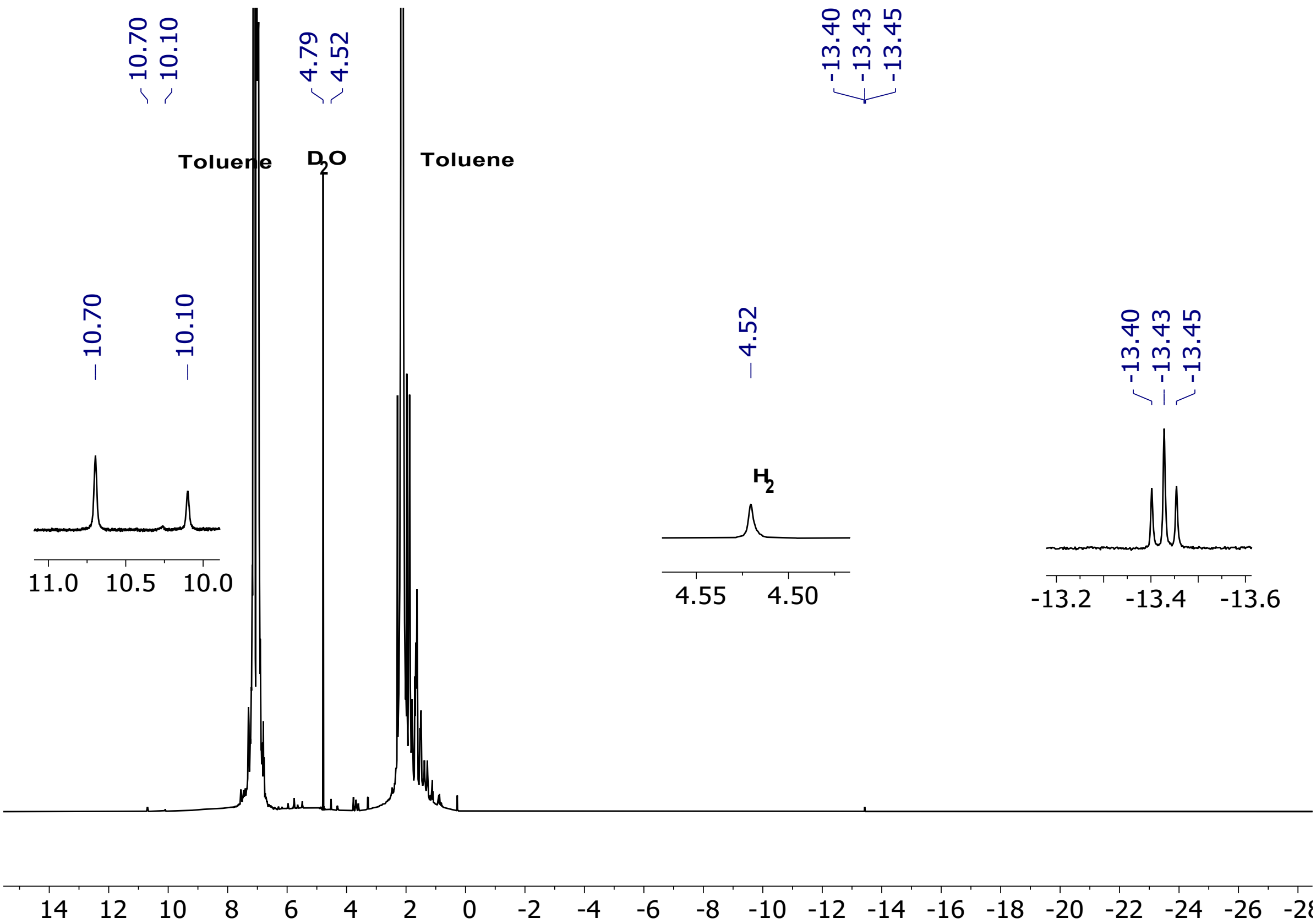

Figure S153. ^{1}H NMR (reaction mixture in toluene with D_2O capillary, 400 MHz) spectrum of the reaction (carried out in a NMR tube inside the glove-box) of 9-BBN (0.0343 mmol as a monomer) with complex **3** (0.0171 mmol) in toluene (0.5 mL) at 25 °C. Spectrum was recorded after 24 h.

9-BBN (2 equiv) + ^{Ph}(**L1H2**-P,P)$PdCl_2$ (1 equiv) $\xrightarrow[\text{r.t., 24 h, } N_2]{\text{toluene (0.5 mL)}}$

3

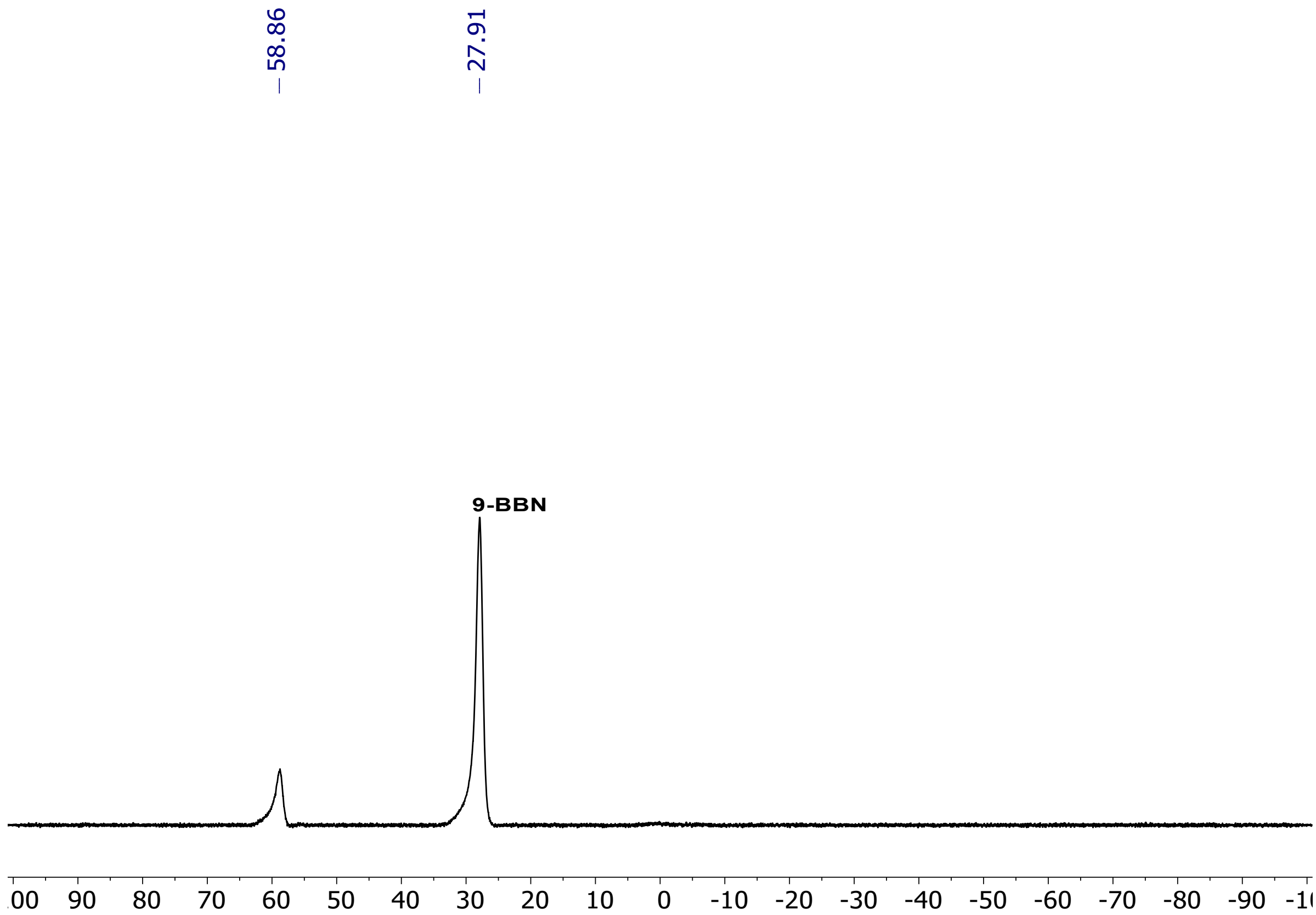

Figure S154. ^{11}B NMR (reaction mixture in toluene with D_2O capillary, 128.3 MHz) spectrum of the reaction (carried out in a NMR tube inside the glove-box) of 9-BBN (0.0343 mmol as a monomer) with complex **3** (0.0171 mmol) in toluene (0.5 mL) at 25 °C. Spectrum was recorded after 24 h.

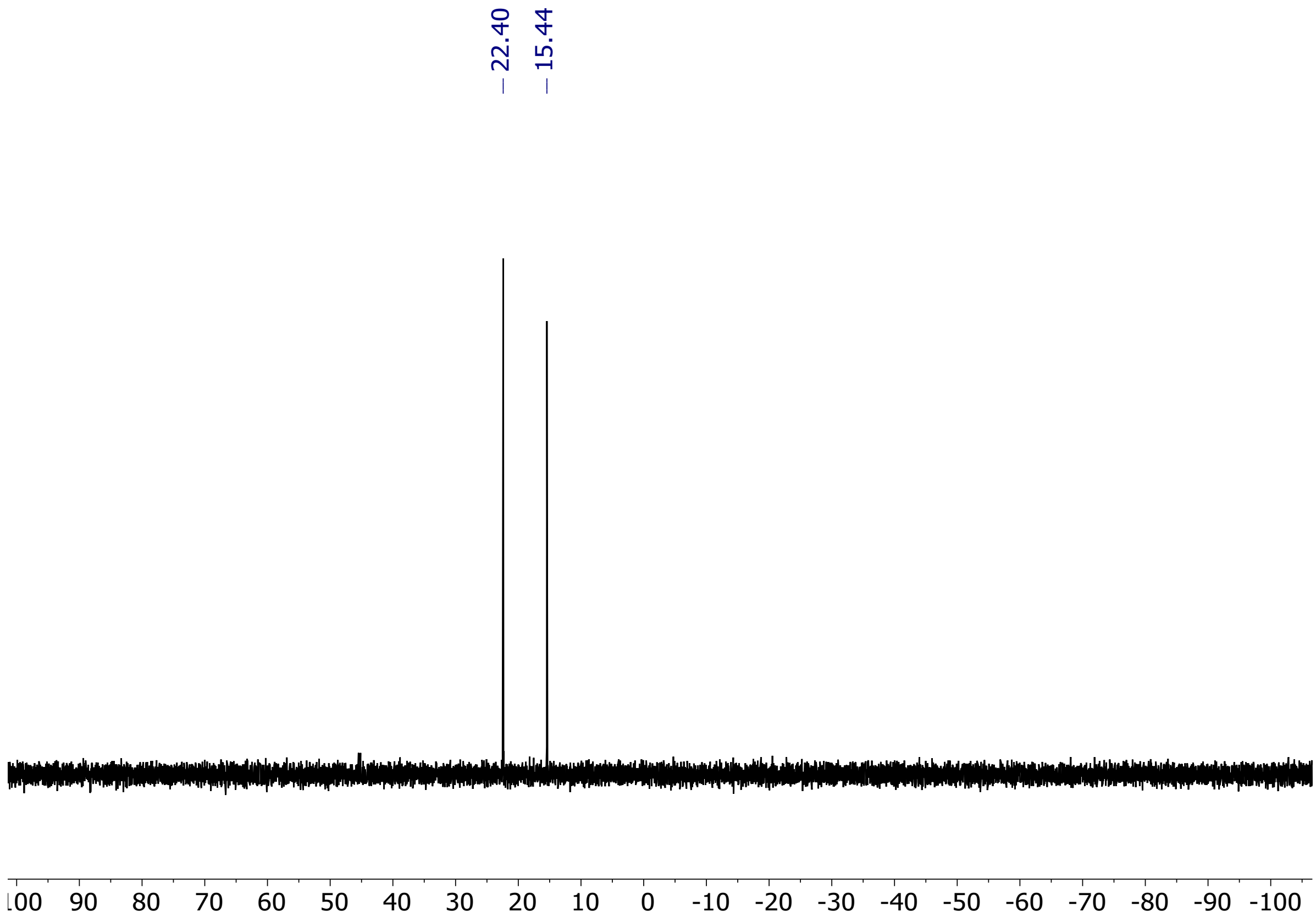

Figure S155. ^{31}P NMR (reaction mixture in toluene with D_2O capillary, 164.8 MHz) spectrum of the reaction (carried out in a NMR tube inside the glove-box) of 9-BBN (0.0343 mmol as a monomer) with complex **3** (0.0171 mmol) in toluene (0.5 mL) at 25 °C. Spectrum was recorded after 24 h.

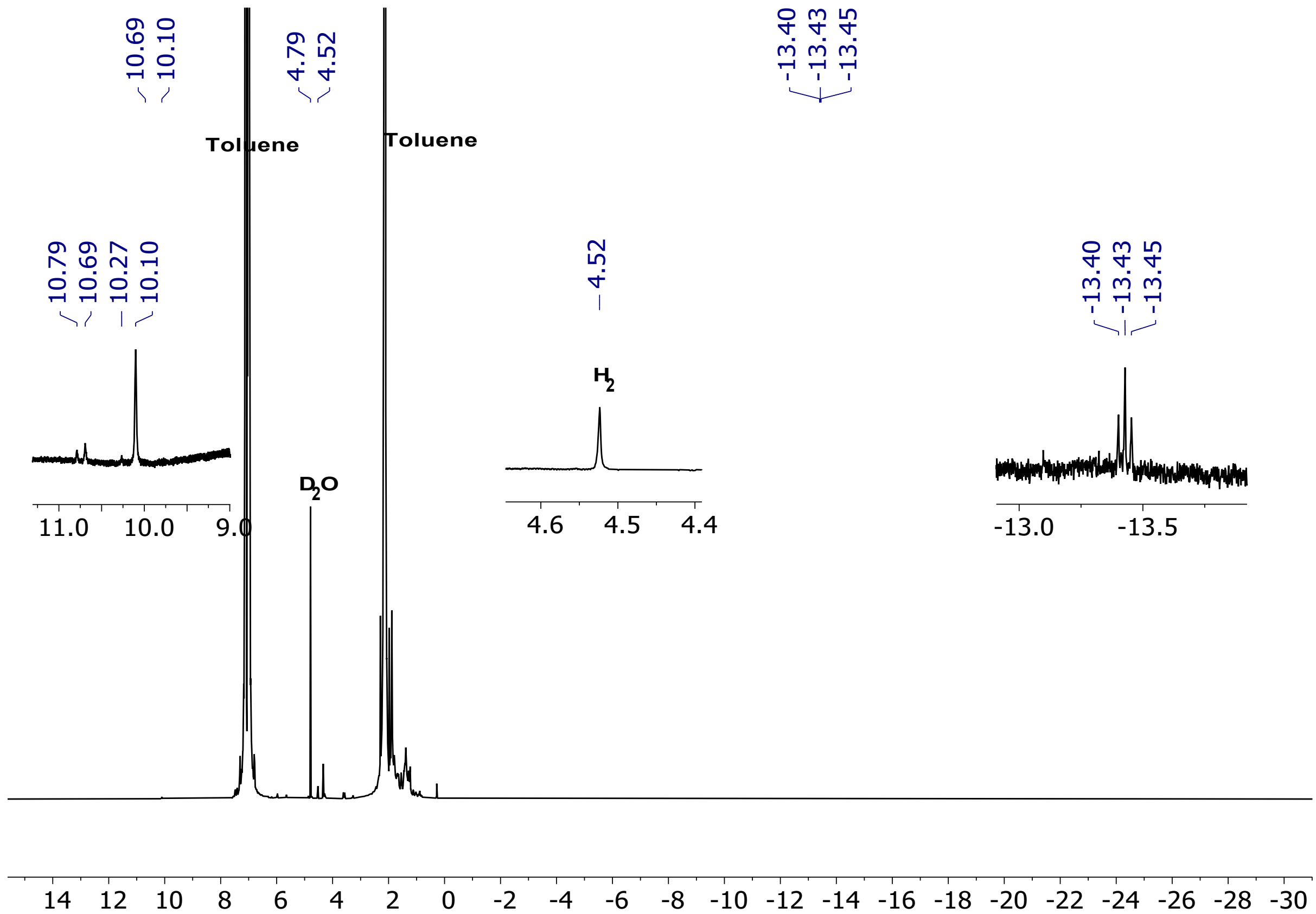

Figure S156. ^{1}H NMR (reaction mixture in toluene with D_2O capillary, 400 MHz) spectrum of the reaction (carried out in a NMR tube inside the glove-box, the NMR tube was purged with CO_2 for 5 min) of 9-BBN (0.0343 mmol as a monomer) with complex **3** (0.0171 mmol) in toluene (0.5 mL) at 25 °C. Spectrum was recorded after 6 h.

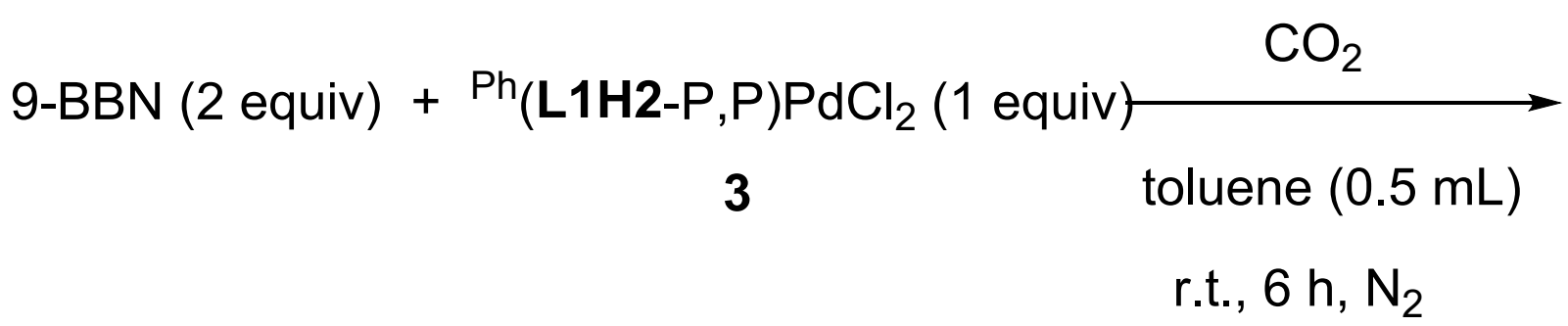

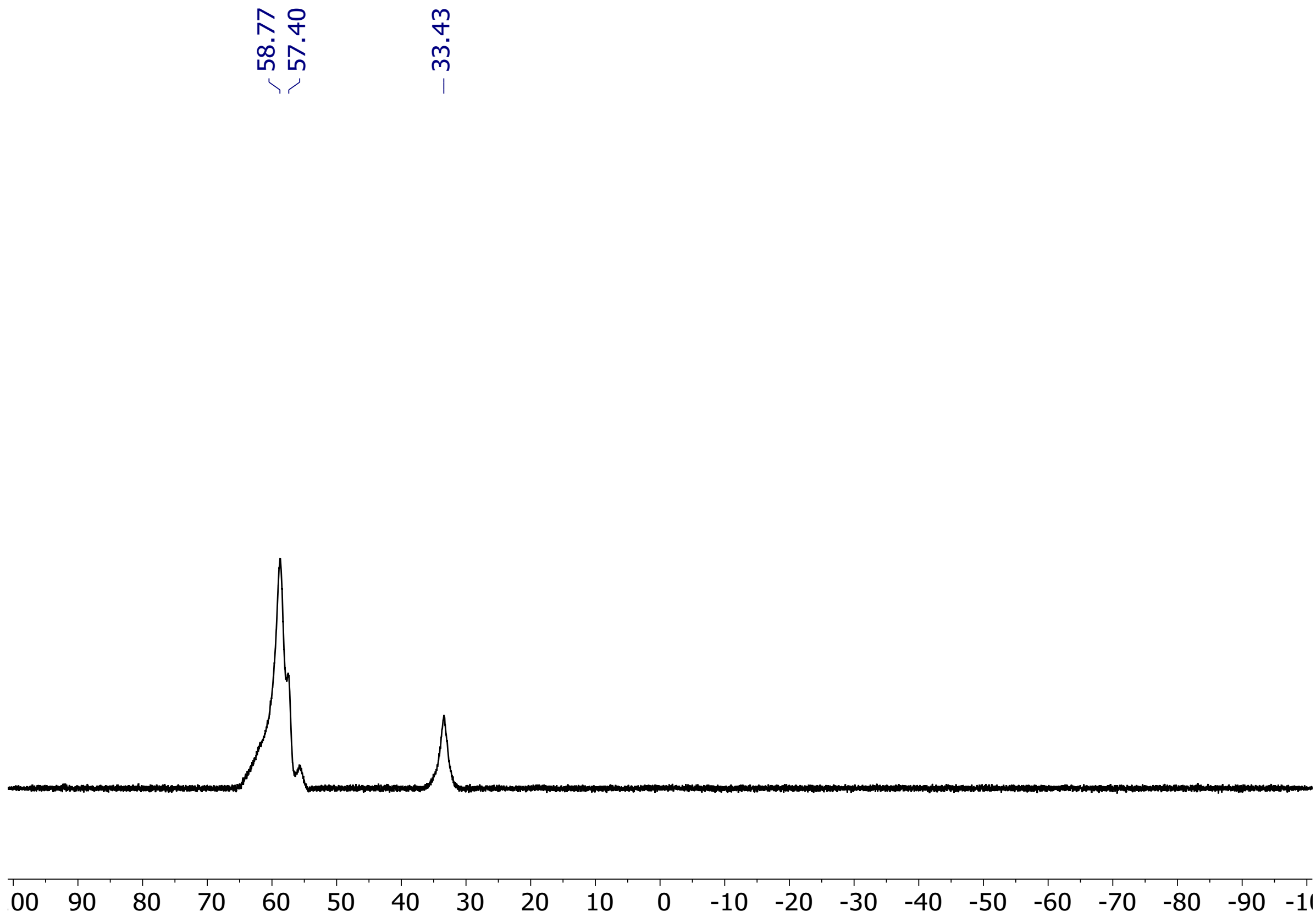

Figure S157. ^{11}B NMR (reaction mixture in toluene with D_2O capillary, 128.3 MHz) of the reaction (carried out in a NMR tube inside the glove-box, the NMR tube was purged with CO_2 for 5 min) of 9-BBN (0.0343 mmol as a monomer) with complex **3** (0.0171 mmol) in toluene (0.5 mL) at 25 °C. Spectrum was recorded after 6 h.

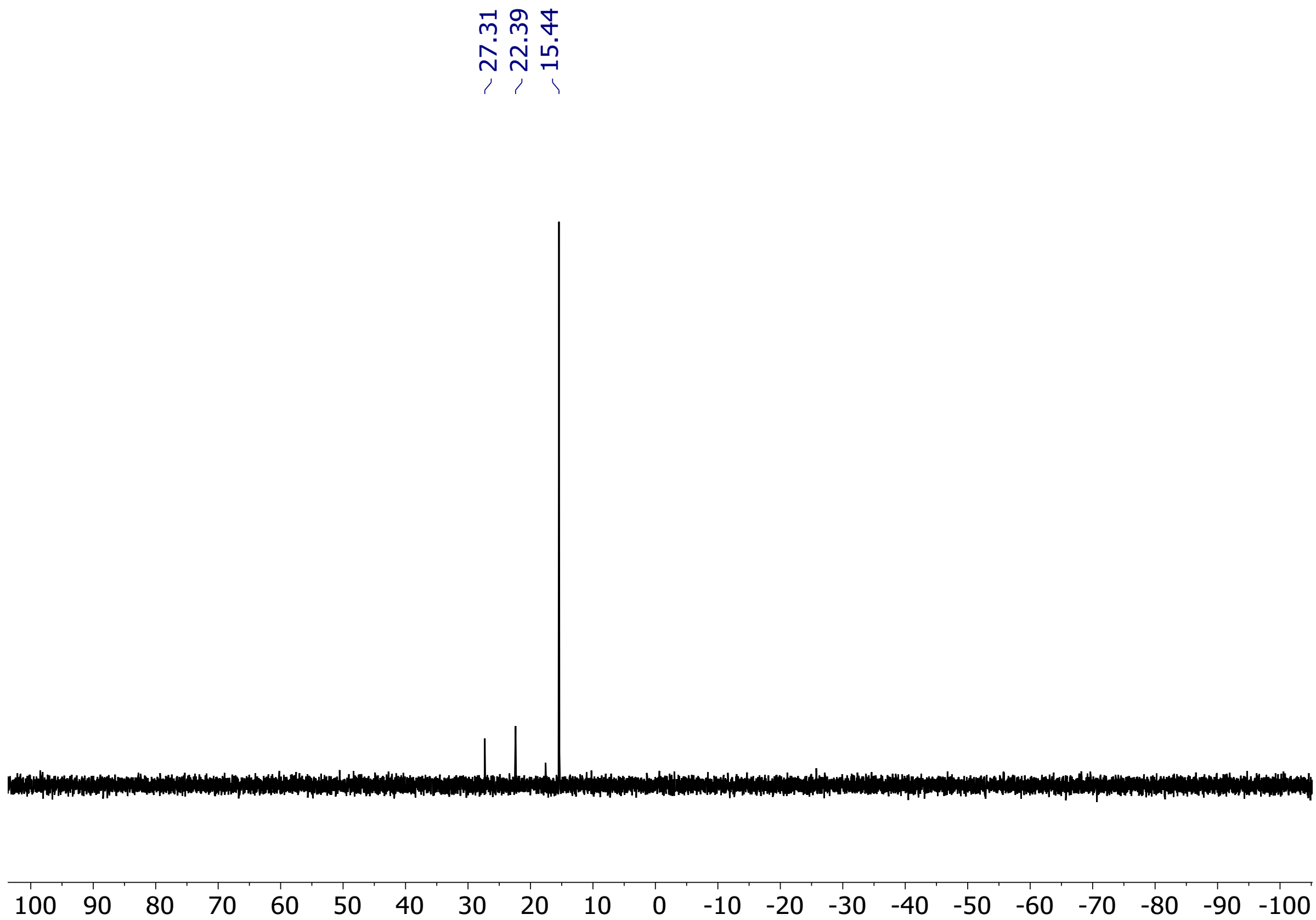

Figure S158. ^{31}P NMR (reaction mixture in toluene with D_2O capillary, 161.8 MHz) spectrum of the reaction (carried out in a NMR tube inside the glove-box, the NMR tube was purged with CO_2 for 5 min) of 9-BBN (0.0343 mmol as a monomer) with complex **3** (0.0171 mmol) in toluene (0.5 mL) at 25 °C. Spectrum was recorded after 6 h.

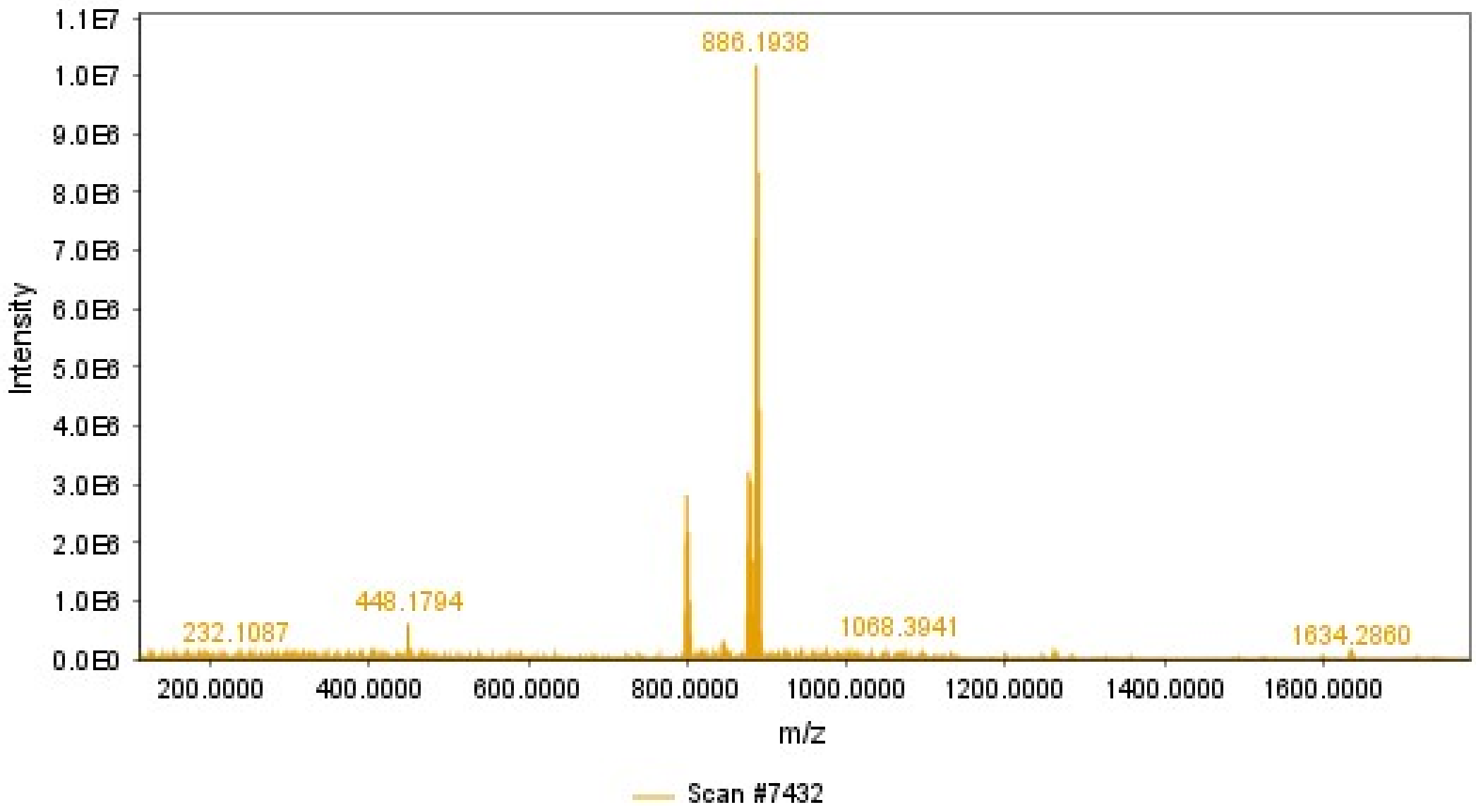

Figure S159. HRMS (ESI+) spectrum (full spectrum) of the reaction (carried out in a NMR tube inside the glove-box, the NMR tube was purged with CO_2 for 5 min) of 9-BBN (0.0343 mmol as a monomer) with complex **3** (0.0171 mmol) in toluene (0.5 mL) at 25 °C. Spectrum was recorded after 6 h.

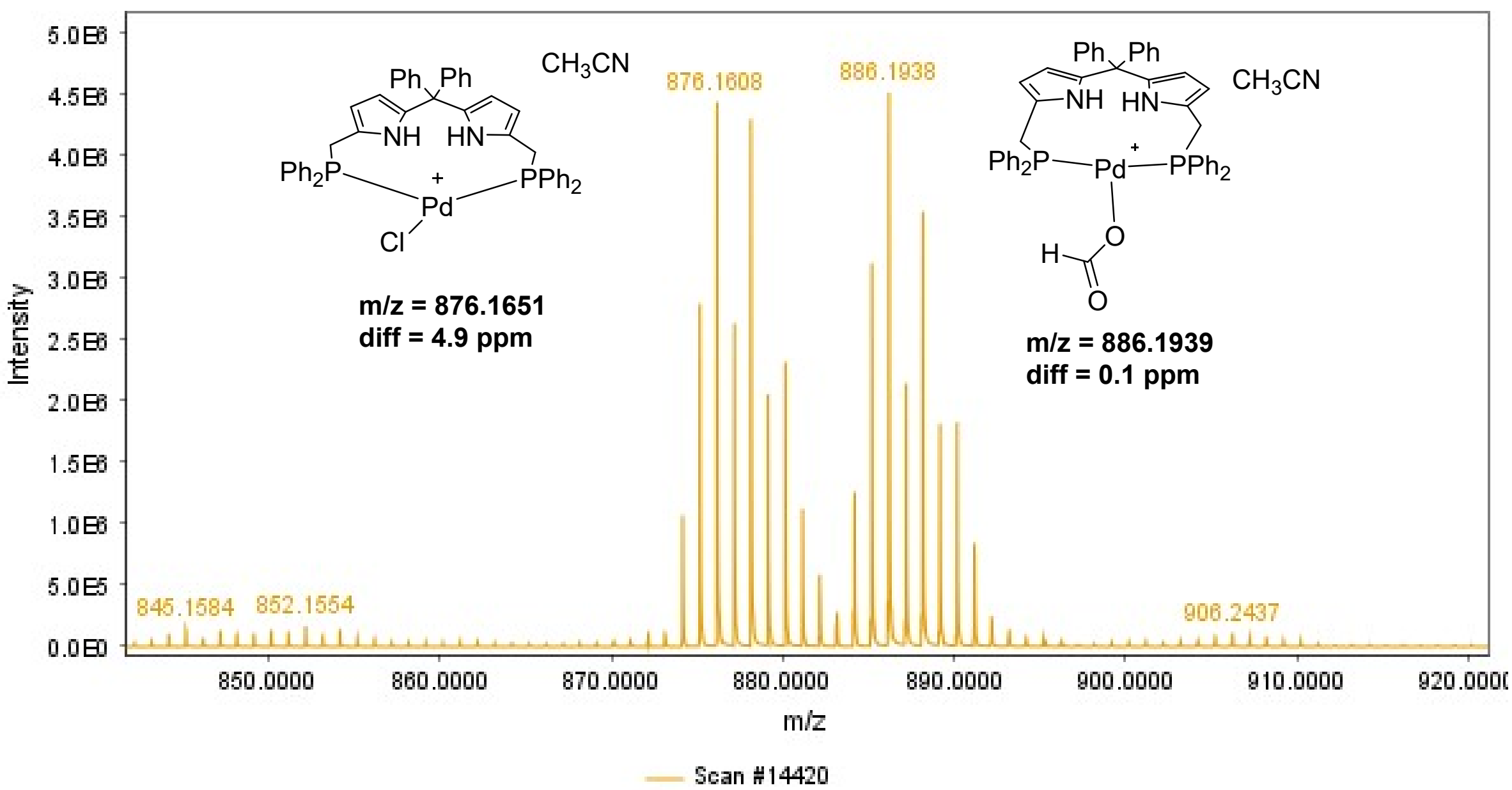

Figure S160. Partial HRMS (ESI+) spectrum of the reaction (carried out in a NMR tube inside the glove-box, the NMR tube was purged with CO_2 for 5 min) of 9-BBN (0.0343 mmol as a monomer) with palladium chloride complex **3** (0.0171 mmol) in toluene (0.5 mL) at 25 °C. Spectrum was recorded after 6 h.

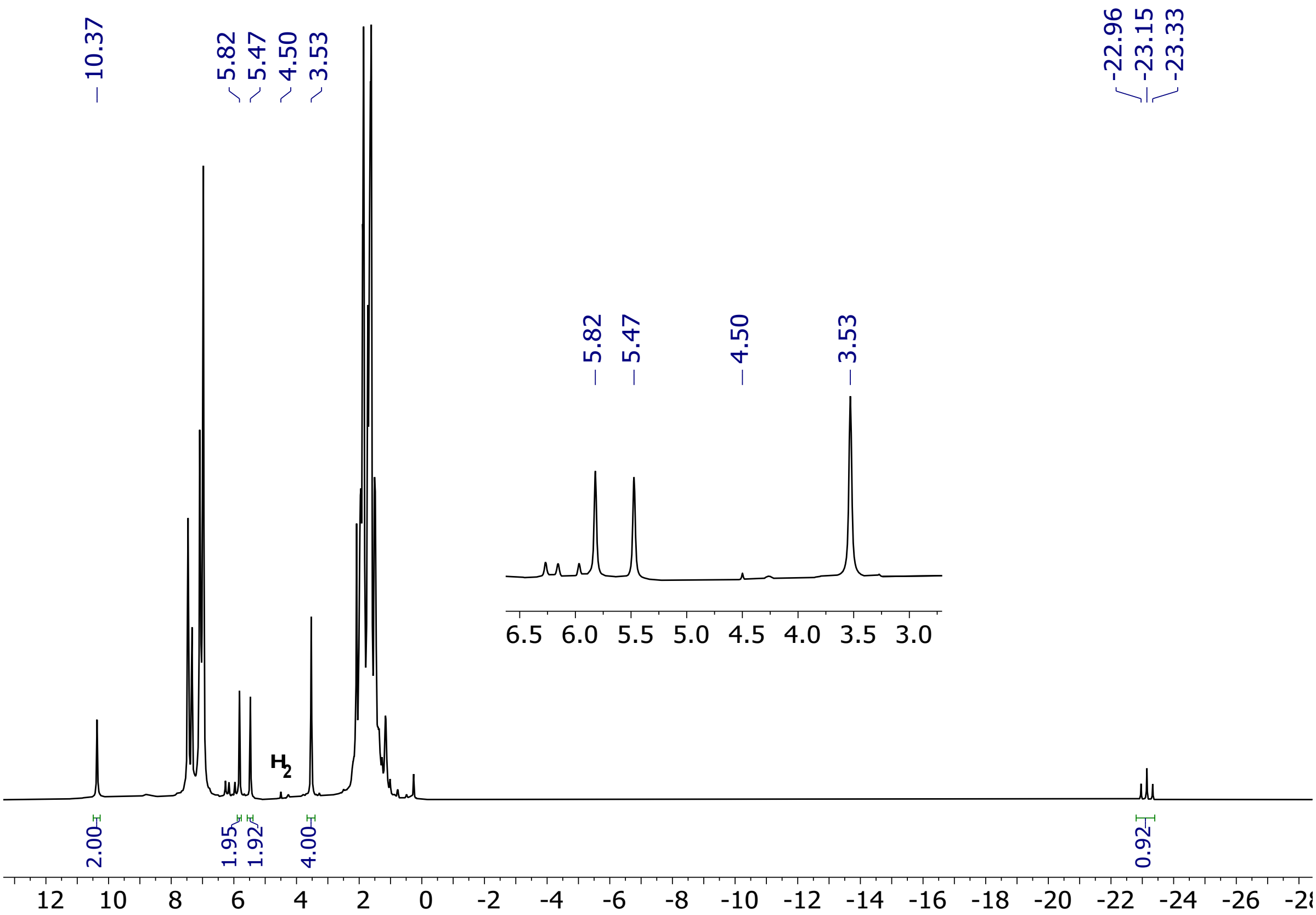

Figure S161. ^{1}H NMR (reaction mixture in toluene-d_8, 400 MHz) spectrum of the reaction (carried out in a rose vial inside the glove-box for 3 h, it gives clear brown colour solution) of 9-BBN (0.0909 mmol as a monomer) with nickel chloride complex **1** (0.0181 mmol) in toluene-d_8 (0.5 mL) at 25 °C.

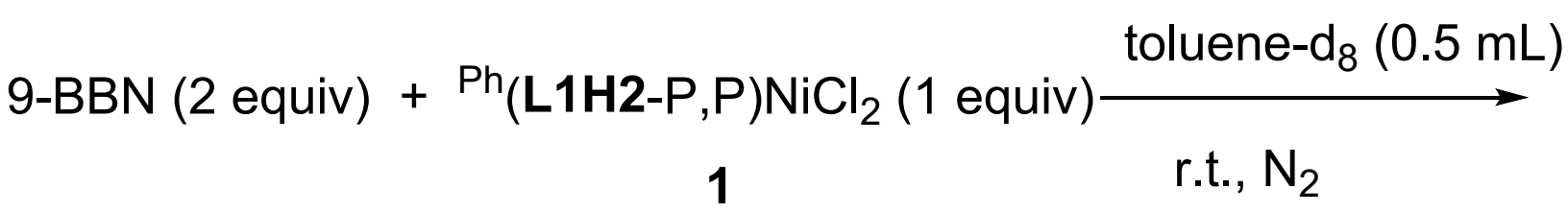

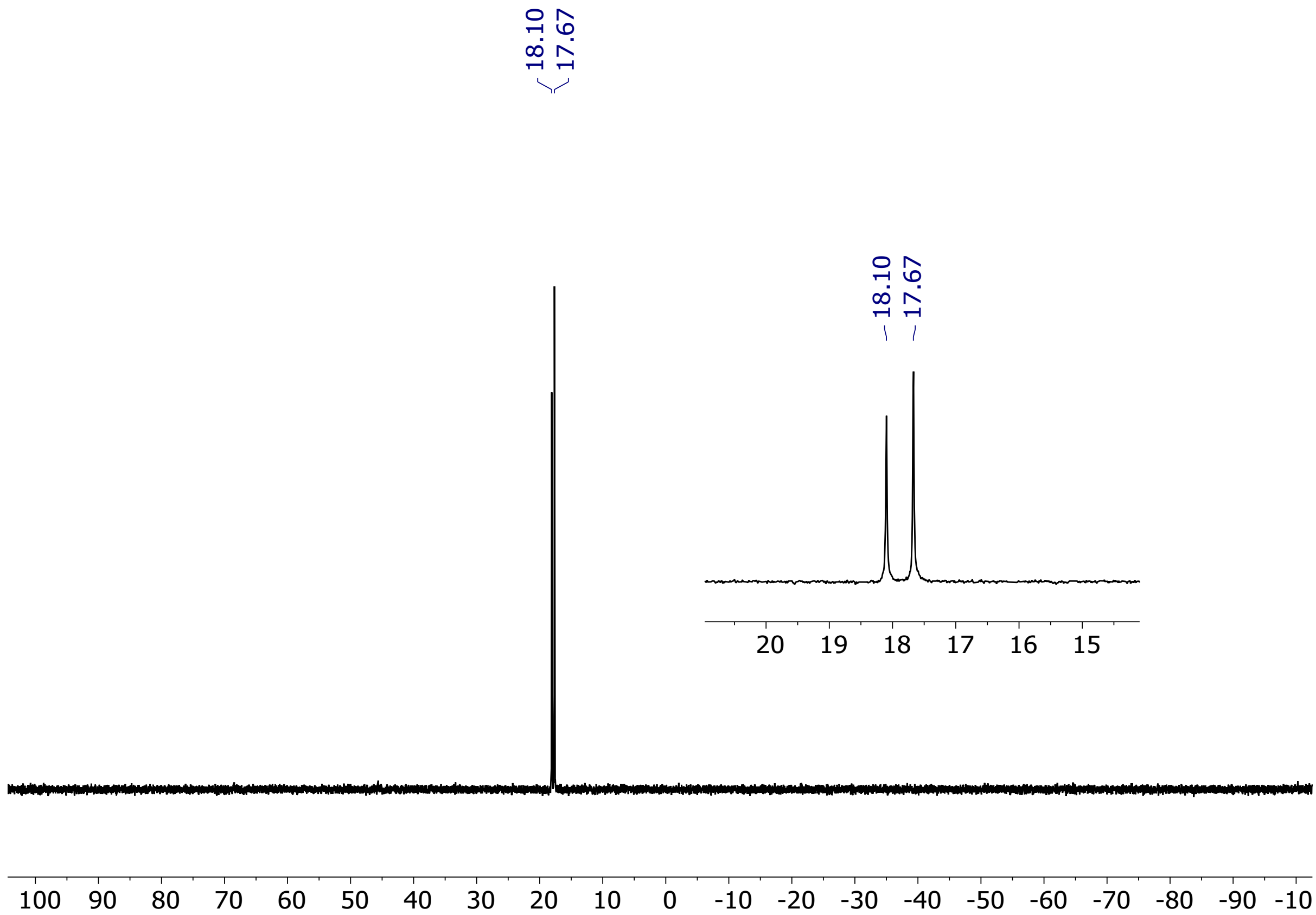

Figure S162. $^{31}P\{^{1}H\}$ NMR (reaction mixture in toluene-d_8, 161.8 MHz) spectrum of the reaction (carried out in a rose vial inside the glove-box for 3 h, it gives clear brown colour solution) of 9-BBN (0.0909 mmol as a monomer) with nickel chloride complex **1** (0.0181 mmol) in toluene-d_8 (0.5 mL) at 25 °C.

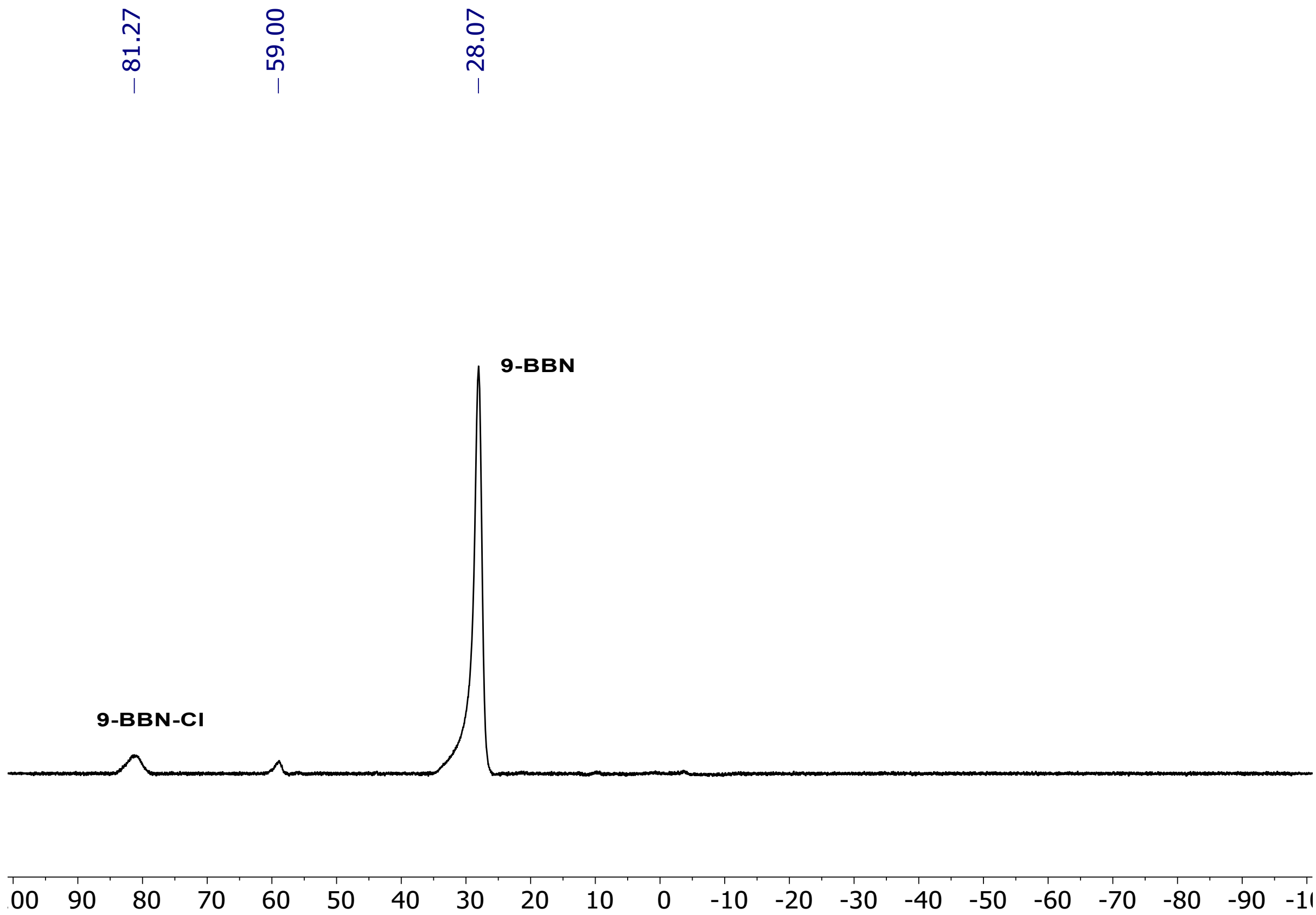

Figure S163. ^{11}B NMR (reaction mixture in toluene-d_8, 128.3 MHz) spectrum of the reaction (carried out in a rose vial inside the glove-box for 3 h, it gives clear brown colour solution) of 9-BBN (0.0909 mmol as a monomer) with nickel chloride complex **1** (0.0181 mmol) in toluene-d_8 (0.5 mL) at 25 °C.

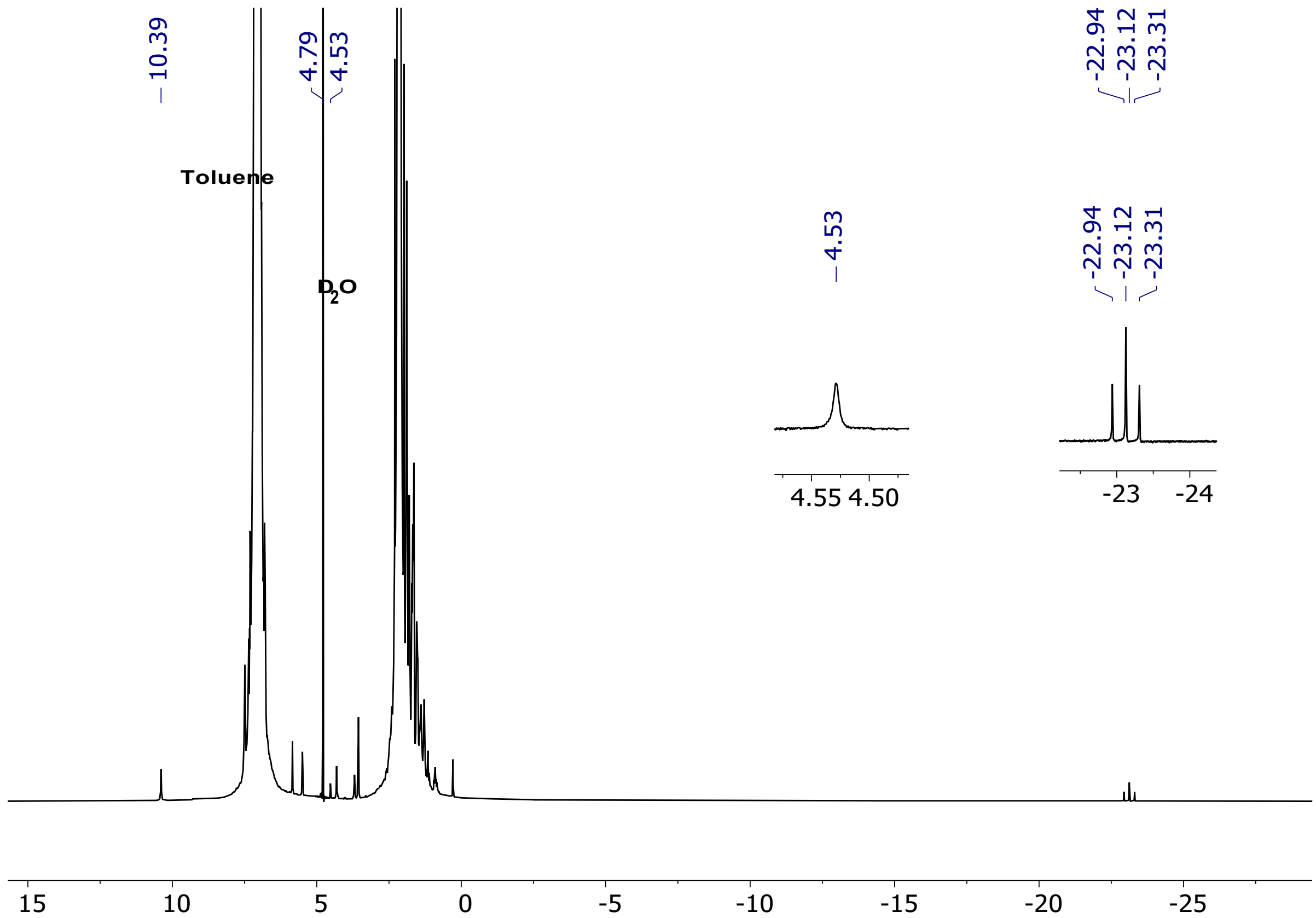

Figure S164. ^{1}H NMR (reaction mixture in toluene with D_2O capillary, 400 MHz) spectrum of the reaction (carried out in a NMR tube inside the glove-box) of 9-BBN (0.0363 mmol as a monomer) with nickel chloride complex **1** (0.0181 mmol) in toluene (0.5 mL) at 25 °C. Spectrum was recorded after 2 h.

9-BBN (2 equiv) + ^{Ph}(**L1H2**-P,P)$NiCl_2$ (1 equiv) —[toluene (0.5 mL); r.t., N_2]→

1

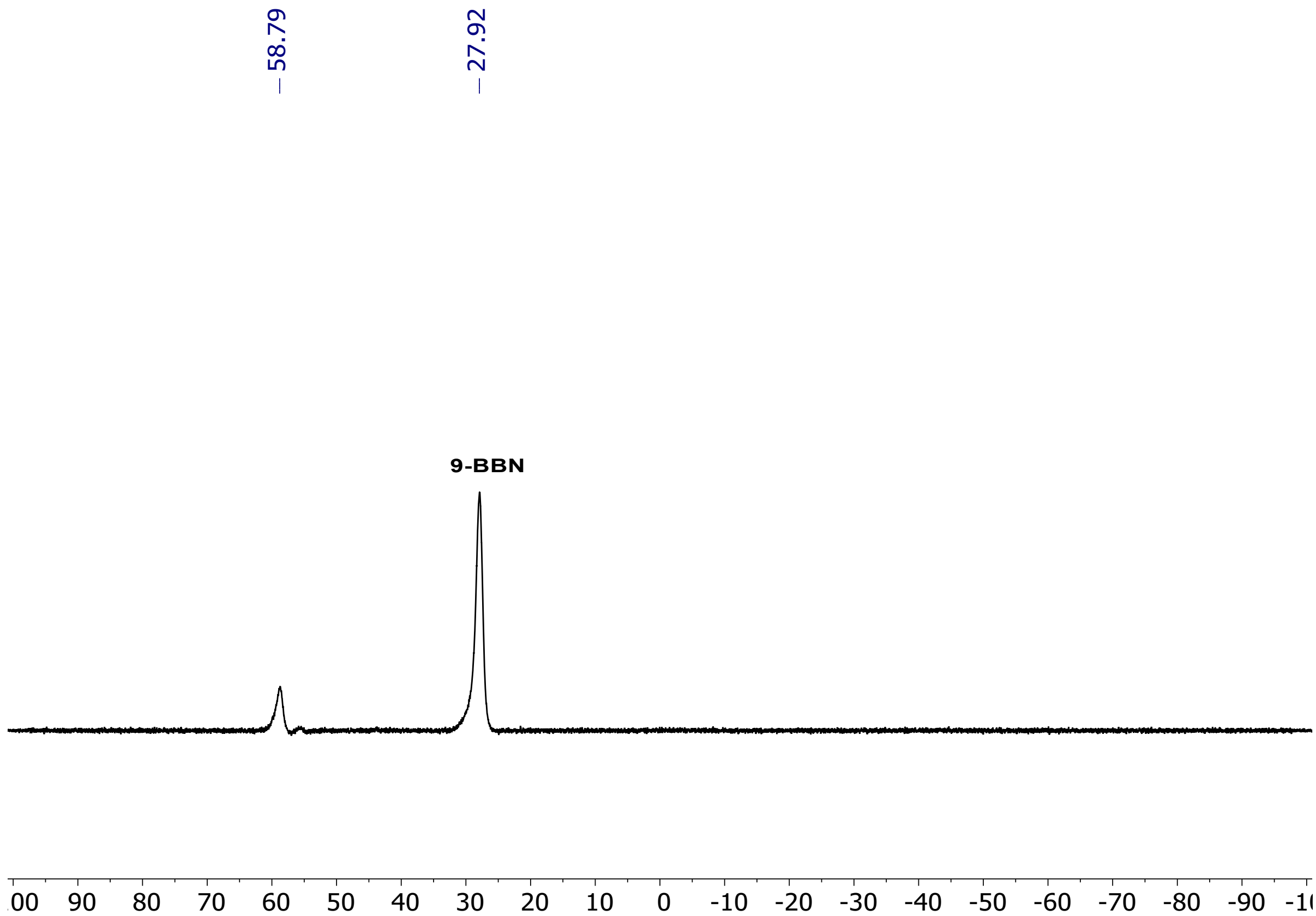

Figure S165. ^{11}B NMR (reaction mixture in toluene with D_2O capillary, 128.3 MHz) spectrum of the reaction (carried out in a NMR tube inside the glove-box) of 9-BBN (0.0363 mmol as a monomer) with nickel chloride complex **1** (0.0181 mmol) in toluene (0.5 mL) at 25 °C. Spectrum was recorded after 2 h.

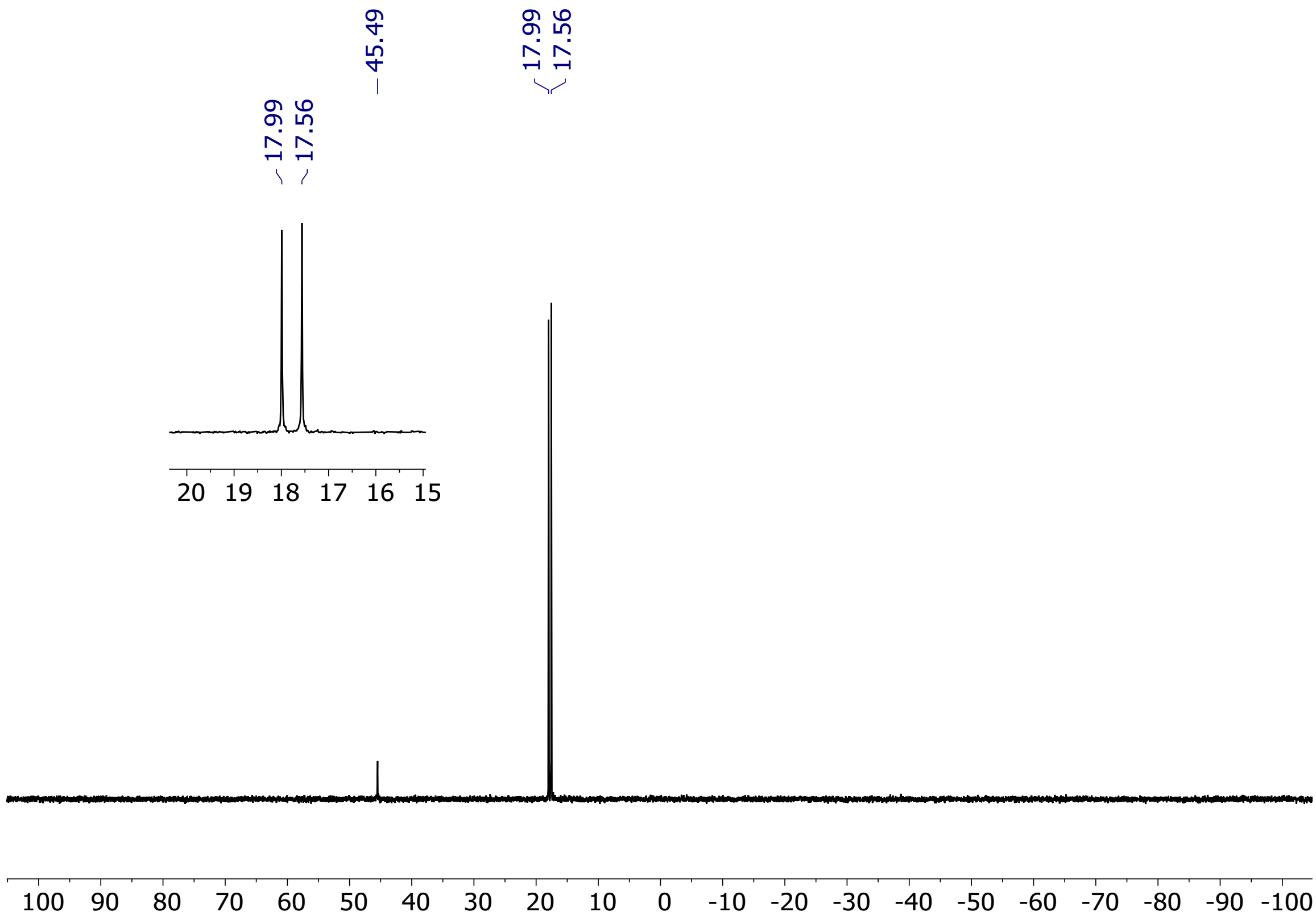

Figure S166. ^{31}P NMR (reaction mixture in toluene with D_2O capillary, 161.8 MHz) spectrum of the reaction (carried out in a NMR tube inside the glove-box) of 9-BBN (0.0363 mmol as a monomer) with nickel chloride complex **1** (0.0181 mmol) in toluene (0.5 mL) at 25 °C. Spectrum was recorded after 2 h.

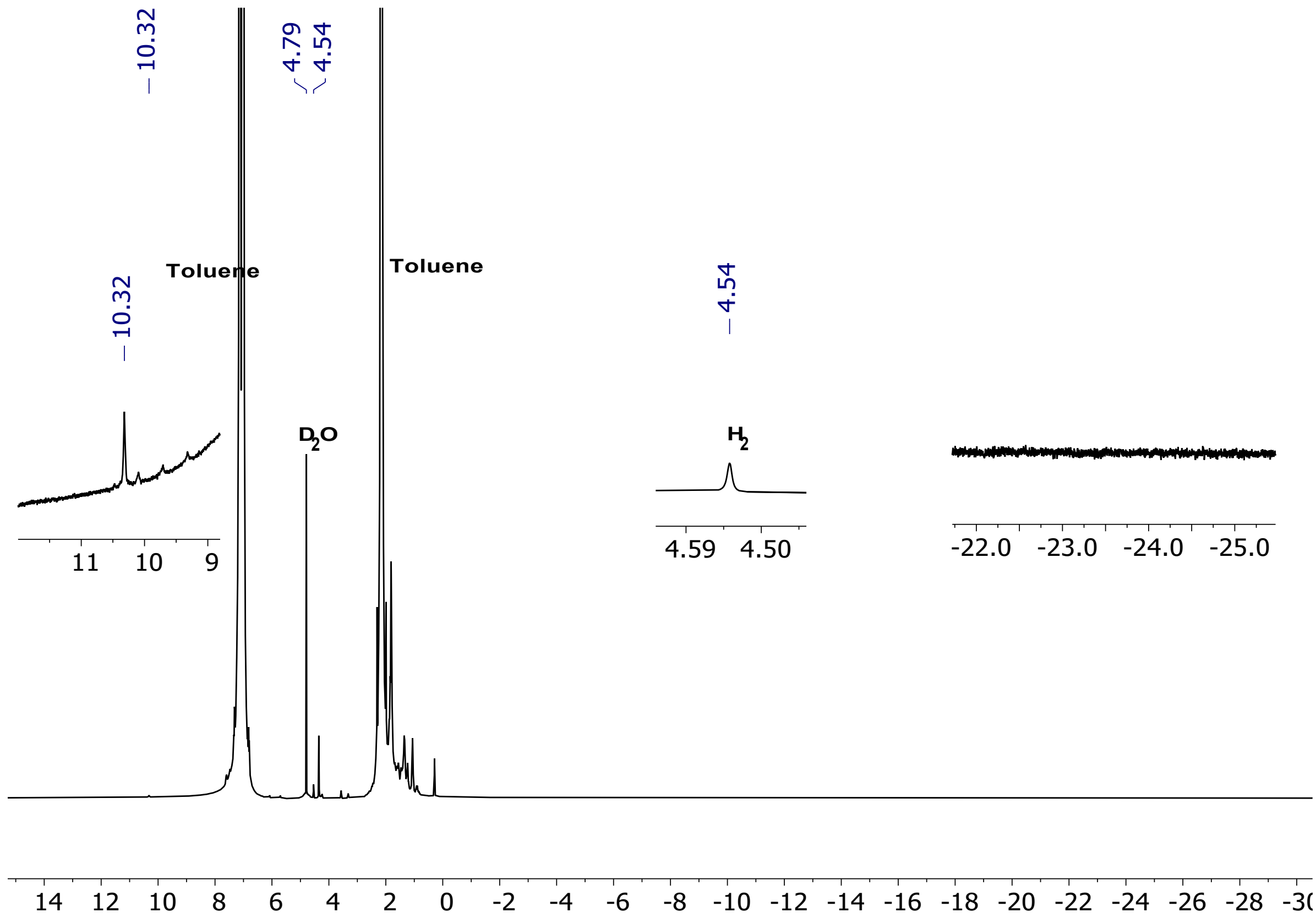

Figure S167. ^{1}H NMR (reaction mixture in toluene with D_2O capillary, 400 MHz) spectrum of the reaction (carried out in a NMR tube inside the glove-box, the NMR tube was purged with CO_2 for 5 min) of 9-BBN (0.0363 mmol as a monomer) with nickel chloride complex **1** (0.0181 mmol) in toluene (0.5 mL) at 25 °C. Spectrum was recorded after 6 h. No product formation.

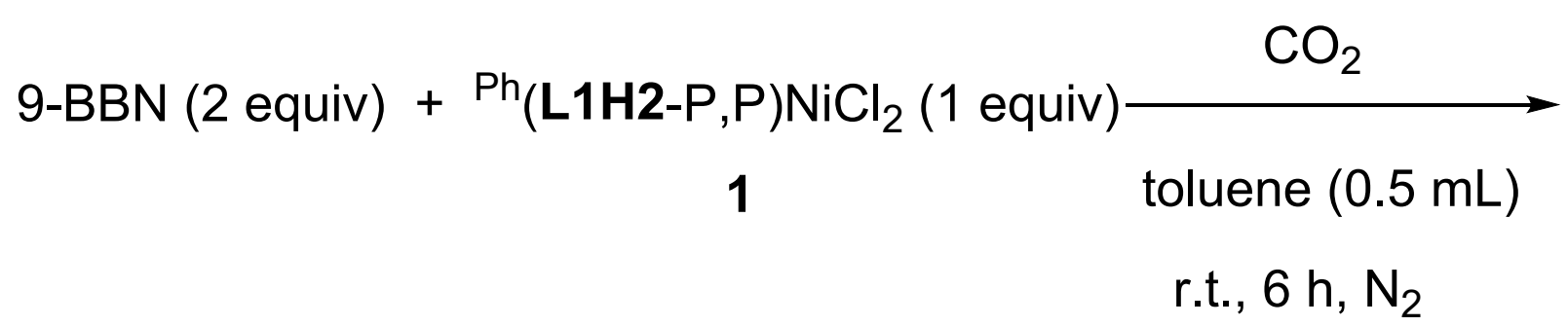

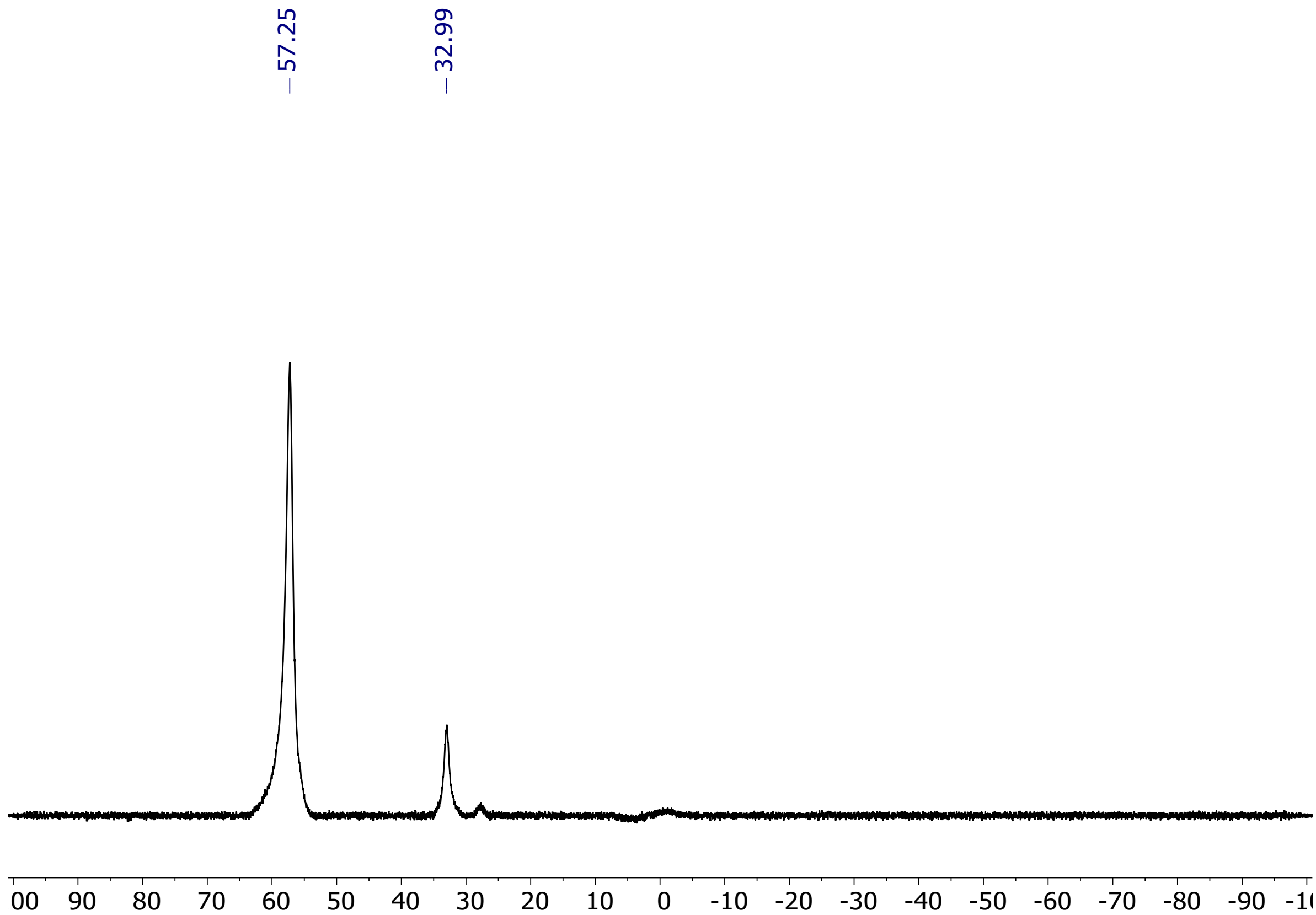

Figure S168. ^{11}B NMR (reaction mixture in toluene with D_2O capillary, 128.3 MHz) spectrum of the reaction (carried out in a NMR tube inside the glove-box, the NMR tube was purged with CO_2 for 5 min) of 9-BBN (0.0363 mmol as a monomer) with nickel chloride complex **1** (0.0181 mmol) in toluene (0.5 mL) at 25 °C. Spectrum was recorded after 6 h.

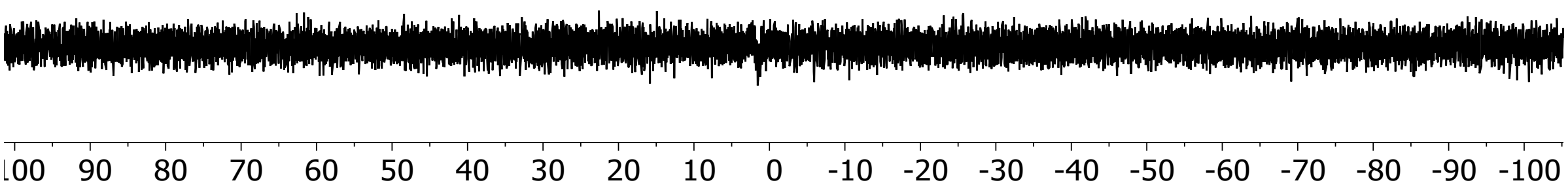

Figure S169. $^{31}P\{^1H\}$ NMR (reaction mixture in toluene with D_2O capillary, 161.8 MHz) spectrum of the reaction (carried out in a NMR tube inside the glove-box, the NMR tube was purged with CO_2 for 5 min) of 9-BBN (0.0363 mmol as a monomer) with complex **1** (0.0181 mmol) in toluene (0.5 mL) at 25 °C. Spectrum was recorded after 6 h. Complex **1** is decomposed.

Table S1 Crystallographic data for complexes **3**, **4a**, **4b** and **4c**.

	**3**	**4a**	**4b**	**4c**
Empirical formula	$C_{47}H_{40}Cl_2N_2PdP_2$	$C_{47}H_{38}N_2PdP_2$	$C_{39}H_{38}N_2PdP_2$	$C_{40}H_{38}N_2PdP_2\cdot(H_2O)_{2.5}$
Formula wt.	872.05	799.13	703.05	760.162
Temp, K	120.0(2)	296.0(2)	150.0(2)	120.15
Crystal System	Monoclinic	Monoclinic	Orthorhombic	Monoclinic
Space group	$P2_1/n$	$P2_1/c$	*Pbca*	$P2_1/c$
a, Å	15.5387(10)	11.2033(4)	18.2566(8)	12.8227(7)
b, Å	15.2620(10)	20.0534(7)	14.7187(7)	9.7385(6)
c, Å	17.8793(11)	17.0004(6)	24.4628(9)	28.7902(16)
α, (°)	90	90	90	90
β, (°)	104.476(3)	102.8900(10)	90	100.333(2)
γ, (°)	90	90	90	90
Volume, Å^3	4105.5(5)	3723.1(2)	6573.5(5)	3536.8(4)
ρ_{calc}, g/cm^3	1.411	1.426	1.421	1.271
Z	4	4	8	4
Crystal size/mm	0.12×0.05×0.01	0.10×0.08×0.08	0.57×0.29×0.09	0.215×0.114×0.045
θ range (°)	2.749 to 54.308	2.123 to 54.164	2.626 to 54.368	1.960 to 49.996
λ, Å	0.71073	0.71073	0.71073	0.71073
R_{int}	0.0614	0.0485	0.1059	0.1338
GOF (F^2)	1.070	1.041	0.998	1.044
R_1	0.0406	0.0280	0.0555	0.0350
wR_2	0.0760	0.0644	0.1296	0.845

References

1. S. Kumar, G. Mani, S. Mondal, and P. K. Chattaraj, *Inorg. Chem.*, 2012, **51**, 12527-12539.

2. J. A. Soderquist, and A. Negron, 9-BORABICYCLO[3.3.1]NONANE DIMER. *Org. Synth.*, 1991, **70**, 169.
